# Neuromotor functions across the lifespan: percentiles from 6 to 80 years

**DOI:** 10.3389/fnagi.2025.1543408

**Published:** 2025-07-29

**Authors:** Tanja H. Kakebeeke, Jon Caflisch, Aziz Chaouch, Valentin Rousson, Flavia M. Wehrle, Oskar G. Jenni

**Affiliations:** ^1^Child Development Center, University Children’s Hospital Zurich, Zurich, Switzerland; ^2^Children’s Research Center, University Children’s Hospital Zurich, Zurich, Switzerland; ^3^Department of Epidemiology and Health Systems, Center for Primary Care and Public Health (Unisanté), University of Lausanne, Lausanne, Switzerland; ^4^University of Zurich, Zurich, Switzerland

**Keywords:** motor development, aging, lifespan, Zurich Neuromotor Assessment (ZNA), body mass index (BMI), percentiles of neuromotor performance

## Abstract

**Aim:**

To investigate the dynamics of neuromotor functions from 6 to 80 years with the Zurich Neuromotor Assessment (ZNA) and to provide reference curves for clinical and research use.

**Materials and methods:**

Neuromotor data on 1620 individuals (828 females) measured with the ZNA and ZNA-2 between 1983 and 2023 were extracted from 11 studies, all performed at the same center in the region of Zurich, Switzerland. Performance on 14 motor tasks was modeled as a function of age and sex while controlling for differences in testing procedures that occurred over the period spanned by the studies. The age of peak performance was identified for each task. Motor performance was converted into standard deviation scores (SDSs) at the task level and combined into the five motor components of the ZNA-2: fine motor, pure motor, balance, gross motor, and contralateral associated movements. The effect of body mass index (BMI) on motor component SDSs was also investigated.

**Results:**

The data showed a rapid increase in motor functions during the first years, particularly before age 10 years, followed by a leveling in performance between the ages of 20 and 40 years. Afterward, a decrease in motor functions was observed in most tasks. However, the age of peak performance and the rate of decline varied greatly between the tasks: motor functions in tasks requiring muscle force deteriorated faster than those involving isolated movements, which showed only mild declines at older ages. Males reached peak performance on average 1 year later than females. High BMI (SDS > 1) was associated with lower balance and poorer gross motor functions.

**Discussion:**

Neuromotor functions undergo dynamic changes throughout the lifespan from early childhood to older adulthood, with peak performances and declines depending on type of motor task and sex. High BMI negatively impacts balance and gross motor functions but not other neuromotor domains. Our findings may inform clinical practice and interventions aimed at optimizing motor functions across the lifespan.

## Introduction

Motor performance plays a crucial role during childhood and adolescence: it supports children’s social interactions and academic success (Stodden et al., 2008). However, it remains essential across the entire lifespan for a healthy, active, and independent lifestyle (Haywood and Getchell, 2014). Thus, the comprehensive study of the evolution of motor functions from infancy to old age is important for providing normative data for diagnostic purposes at all ages, tracking changes over time, describing individual trajectories, predicting future motor performance, and informing treatment and interventions (Burton and Miller, 1998; Burton and Rodgerson, 2001).

It is generally accepted that motor functions improve during childhood, stabilize during adulthood, and then gradually decline in older age (Haywood and Getchell, 2014; JafariNasabian et al., 2017). However, the data on motor performance are largely limited to specific age ranges [e.g., childhood (Kakebeeke et al., 2018), or old age (Galhardas et al., 2022; Lanzino et al., 2012; Perera et al., 2014)] or to selected motor tasks (Koerte et al., 2010; Springer et al., 2007). Furthermore, studies often focus on children and adolescents with abnormal or delayed motor development (Folio and Fewell, 2000; Henderson et al., 2007; Kakebeeke et al., 2018) or on the decline of functions in old age (Samuel et al., 2012; Arking, 2006).

What is required is a coherent description of the normal course and variability of motor functions across the entire lifespan. For example, McKay et al. (2017) provided data for 12 fine and gross motor (GM) functions from 3 to 101 years, whereas Springer et al. (2007) focused on static balance between 18 and 99 years. However, the tracking of motor functions for clinical and research purposes would be facilitated by age- and sex-specific percentile reference curves, and these are still lacking. A key requirement for developing such percentiles is the reliable assessment of the same motor functions across the lifespan without variations in test design, motor tasks, or study procedures.

The Zurich Neuromotor Assessment (ZNA) meets these criteria. The test is widely used in clinical practice and research to assess motor skills across various domains, including fine and gross motor functions, balance, and associated movements (Kakebeeke et al., 2018). The ZNA is designed for a broad age range and provides objective measurements of neuromotor performance and quality of movements. Normative data for the ZNA have been published for individuals aged 3–18 years (Kakebeeke et al., 2018), and additional data are available for adults around ages 45 and 65 years (Kakebeeke et al., 2023). However, the ZNA has not been applied beyond age 65, and a comprehensive cross-sectional evaluation from early childhood to old age with percentile-based norms for motor functions is still lacking.

In this study, we provide normative data on neuromotor functions covering a variety of motor tasks over the lifespan in continuous, sex-specific percentile curves. Additionally, we examine the impact of body mass index (BMI) on neuromotor functions.

## Materials and methods

This study combines cross-sectional measurements on motor functions collected on 1,620 individuals (828 females) aged between 6.0 and 81.5 years. The motor measurements were selected from 11 studies conducted by our group and at the same center between 1983 and 2023 (Wehrle et al., 2021; Largo et al., 2001b; Largo et al., 2001a; Rousson et al., 2008; Wilhelm et al., 2014; Wehrle et al., 2016; Kakebeeke et al., 2018; Knaier et al., 2023; Kakebeeke et al., 2023). For the studies after 2015, the ZNA-2 was used (Kakebeeke et al., 2023; Kakebeeke et al., 2018; Knaier et al., 2023; see Table 1). BMI measurements were available for 1,040.

**TABLE 1:** Pooled sample characteristics.

Study	Origin see	*n* (% females)	Age range (in years)	Used test	BMI (*n*)
ZLS-1	Wehrle et al., 2021	86 (60.5)	29.6–56.8	ZNA	56
ZLS-2	Wehrle et al., 2021	79 (44.3)	7.0–33.0	ZNA	23
ZLS-3	Wehrle et al., 2021	262 (51.1)	6.0–34.0	ZNA	251
ZNA norms	Largo et al., 2001b; Largo et al., 2001a	385 (51.4)	6.0–14.1	ZNA	0
ZNA reliability	Rousson et al., 2008	22 (54.5)	7.0–8.2	ZNA	0
Controls 2014	Wilhelm et al., 2014	42 (28.6)	8.7–25.8	ZNA	0
Controls 2016	Wehrle et al., 2016	32 (53.1)	10.0–17.0	ZNA	0
ZNA-2 norms	Kakebeeke et al., 2018	442 (50.0)	6.0–22.5	ZNA-2	442
Controls 2023	Knaier et al., 2023	17 (35.3)	8.0–14.9	ZNA-2	17
ZLS 1 and 2 as adults	Kakebeeke et al., 2023	111 (49.5)	40.8–67.6	ZNA-2	109
ZNA-p	Presented here	142 (60.6)	60.5–81.5	ZNA-2	142
Total		1,620 (51.1)	6.0–81.5		1,040

ZLS: Zurich Longitudinal Studies; ZNA: Zurich Neuromotor Assessment, first version; controls, normal controls of clinical studies; ZNA-2: Zurich Neuromotor Assessment, second version; ZNA-p: pensionaries 60–82 years who performed the ZNA-2 adapted for this age.

For details on the study cohorts, please refer to the previous publications (Wehrle et al., 2021; Largo et al., 2001b; Largo et al., 2001a; Rousson et al., 2008; Wilhelm et al., 2014; Wehrle et al., 2016; Kakebeeke et al., 2018; Knaier et al., 2023; Kakebeeke et al., 2023). Because these studies only covered the ages from 6 to 67.6 years, an additional cohort was recruited to specifically capture data on the elderly. For this purpose, retired employees of the University Children’s Hospital Zurich and their partners were recruited (ZNA-p), with the only inclusion criteria being that they were physically mobile to come to the hospital for testing without walking aids and were free of known neurological diseases. For more details on the ZNA-p, please refer to Supplementary material 1. A few adaptations of the ZNA-2 test protocol were introduced in the ZNA-p, such as a reduction in the number of repetitions in the GM tasks of chair-rise and jumping sideways and the omission of the standing long jump to prevent injuries.

The general inclusion criteria were the absence of any known neurological disease, chronical physical illness, handicap or prematurity. In addition, for children and adolescents the inclusion criterion was attending regular schooling. For the cohort of 65–80 years old subjects: cognitive decline was an exclusion criterion as measured by mini-mental-state-assessment (Folstein et al., 1975). All studies from which data were extracted were approved by the Ethical Commission of the Canton Zurich and were performed according to the Declaration of Helsinki. For studies involving children, all families received a study description, and the primary parent or guardian provided written consent for the participation of the children. Additionally, children from 6 to 14 years gave verbal consent. Adolescents older than 14 years gave written informed consent. All participants older than 18 years provided consent.

Individuals were tested with either the original ZNA (Largo et al., 2001b; Largo et al., 2001a) or the ZNA-2 (Kakebeeke et al., 2018; Kakebeeke et al., 2013). The ZNA (the original form as well as its update ZNA-2) is a standardized procedure to describe neuromotor development in children and adolescents. It measures neuromotor performance for five components: fine motor (FM), pure motor (PM), balance (BA), gross motor (GM), and movement quality in contralateral associated movements (CAMs). An overview of the structure of the test in presented in Table 2. Original ZNA data were accommodated to the ZNA-2 testing procedures. For a comparison between the two versions, see Supplementary material 2. Recently, the ZNA-2 has also been used with adults (Kakebeeke et al., 2023).

**TABLE 2:** Overview of the tasks of the ZNA-2 (Kakebeeke et al., 2019).

Components	Tasks		Performance	Quality	Abbreviations
Fine motor (FM)	Pegboard		10 Pegs	CAMs	PGB d/nd
	Bolts		Bolts: out-in	CAMs	BLT d/nd
	Beads		5 Beads		BDS
Pure motor (PM)	Repetitive movements	Foot	20×		RFT d/nd
		Hand	20×		RHD d/nd
		Fingers	20×		RFG d/nd
	Alternating movements	Foot	10×	CAMs	AFT d/nd
		Hand	10×	CAMs	AHD d/nd
	Sequential movements	Fingers	5×	CAMs	SFG d/nd
Balance (BA)	Standing on one leg	Eyes open	Best of 2 trials, max 30 s		SBO d/nd
		Eyes closed	Best of 2 trials, max 30 s		SBC d/nd
Gross motor (GM)	Jumping sideways		20×*		JSW
	Chair-rise		10×		CHR
	Standing long jump		Average of 2 trials**		SLJ

d: dominant side; nd: non-dominant side. *ZNA-p only performs half of the amount of jumps. **Not performed in the ZNA-p.

### Data analysis

Outcomes of interest were timed performance (seconds), jumping distance (centimeters), and an index measuring movement quality called the intensity of CAM (see Kakebeeke et al., 2018 for more details). The intensity of CAM can take 24 possible values between 0 and 5 and is approximately normally distributed over that range in healthy individuals (Kakebeeke et al., 2018).

Three of the 11 studies from which data were extracted featured data from the Zurich Longitudinal Studies (ZLS) (Wehrle et al., 2021). To maintain a cross-sectional design well suited to modeling developmental norms, we randomly selected one data point per individual from the longitudinal measurements by employing unequal sampling probabilities. The sampling probabilities were designed to favor the selection of a single data point among longitudinal measurements (a) in age ranges that were less frequently represented in the cross-sectional studies and (b) at ages where measurements on many other tasks were also available.

Generalized additive models for location, scale, and shape implemented in the gamlss R package (Rigby and Stasinopoulos, 2005) were used to model the evolution of timed performance, jumping distance, and intensity of CAMs as a function of age and sex. This framework provides flexibility in modeling developmental tasks by employing simple models (e.g., linear regression, albeit possibly on some transformed scale) when appropriate, while also accommodating more complex features—such as skewness and heteroscedasticity—when supported by the data. Model selection at each stage was guided by the Bayesian information criterion (BIC) (Schwarz, 1978), which imposes a stronger penalty for model complexity than alternatives like the Akaike information criterion (AIC) (Akaike, 1974). As a result, BIC tends to favor more parsimonious models, leading to smooth percentile curves. When applicable, the models were adjusted for changes in testing procedures (see Supplementary material 2 for a description of differences between ZNA and ZNA-2). Consequently, predicted percentile curves were derived as if all measurements had been taken using the ZNA-2, with the same procedure over the whole lifespan, as defined in Table 2. Timed performance and jumping distance were modeled with a Box-Cox Cole and Green distribution (also called LMS method) (Cole and Green, 1992), which captures changes in median, variability, and skewness and reduces to a lognormal distribution when the shape parameter controlling skewness is fixed to zero. A right-censored lognormal model addressed ceiling effects in SB tasks. CAM intensity was modeled with an interval-censored Gaussian distribution using censoring intervals defined by the mid points between the 24 possible values of intensity. This approach has already been used to model CAM intensity in the ZNA and ZNA-2 (Largo et al., 2001b; Kakebeeke et al., 2018), and is justified both from a clinical and statistical point of view (Gasser and Rousson, 2004; Gasser et al., 2007). The age effect was modeled using penalized splines (Eilers and Marx, 1996) while optimizing the BIC for smoothness. The splines were fitted to the logarithm of age because this resulted in a lower BIC and thus improved fit in nearly all tasks. The model included interactions between splines and sex to provide separate norms for males and females. A pragmatic model selection refined the structure of scale and shape parameters, starting with a simple intercept model and adding age and sex effects only if they improved BIC. The shape parameter was modeled with either the same or a simpler structure than the scale parameter. Outliers were identified by calculating task standard deviation scores (SDSs) and flagging those observations exceeding ±3 SDS. The fitting procedure was then repeated after excluding the previously flagged data until no additional outliers were detected.

A bootstrap procedure was used to establish 95% confidence intervals (CIs) for the 50th percentile curve by resampling individuals within sex (200 replications) and refitting the norms while fixing the degrees of freedom in the penalized splines. This method also allowed us to generate pointwise 95% CIs for the sex difference at each age to highlight where typical motor functions in males differed significantly from those in females. Additionally, a likelihood ratio test was conducted to assess the sex difference globally across all ages.

For each task, we examined the age at which motor functions began to decline by plotting the first derivative, or slope, of the 50% centile curve against age for males and females. Pointwise 95% CIs for the first derivative were also obtained through the bootstrap procedure. We identified the age at which the first derivative reached zero, indicating peak performance, for each sex and reported the oldest age when it occurred multiple times. Finally, we calculated the rank correlation between the ages of peak performance for males and females across the tasks separately for timed performance or jumping distance and CAMs.

Task SDSs were combined into the five ZNA-2 components after replacing missing task SDSs by the average of SDS available for tasks loading on the same component. In later analyses, each component SDS was weighted inversely to the proportion of imputed task SDS. BMI centiles, adjusted for age and sex, were derived from the subset of data with available BMI measurements using the same approach employed to model motor functions, thus allowing for the calculation of age- and sex-adjusted BMI SDS. Motor component SDSs were modeled as a function of smooth effects of BMI SDS using generalized additive models with the mgcv R package (Wood, 2004) while using weights defined by the number of imputed task SDSs. The impact of BMI on each ZNA-2 component was visualized by plotting predicted motor SDS and its 95% CI against BMI SDS.

Median curves (with 95% bootstrap CI) for males and females obtained in this study were compared with results from McKay et al. (2017) on standing long jump, including 1,000 healthy individuals from Sydney, Australia and with the findings from Springer et al. (2007) for standing on one leg with eyes open on 549 healthy participants from Washington DC. In the latter case, our results were adapted to conform to the procedure and censoring time used in the Springer et al. (2007) study. All statistical analyses were conducted in R software version 4.4.1 (R Core Team, 2024).

## Results

The highest proportion of data points excluded during model fitting due to |SDS| > 3 was 1.1%, observed in the repetitive hand movement task with the non-dominant hand. All other tasks had less than 1% of outliers (see Supplementary material 5).

### Effects of age and sex

**FIGURE 1:**
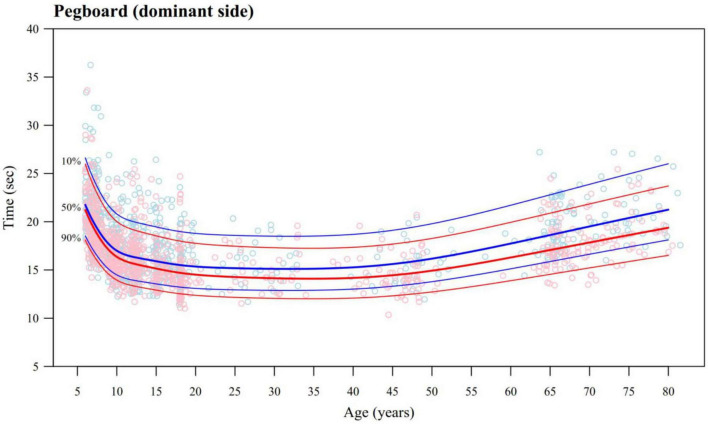
Pegboard (dominant side) timed performance (seconds) as a function of age, with estimated percentile curves (10%, 50%, and 90%) as a fine motor component task. Male data and percentiles are represented with blue dots and curves, and female data and percentiles are represented with red dots and curves. Observed (dots) and predicted timed performance (percentile curves) are normalized to the exercise with 10 pegs, as in the ZNA-2.

**FIGURE 2:**
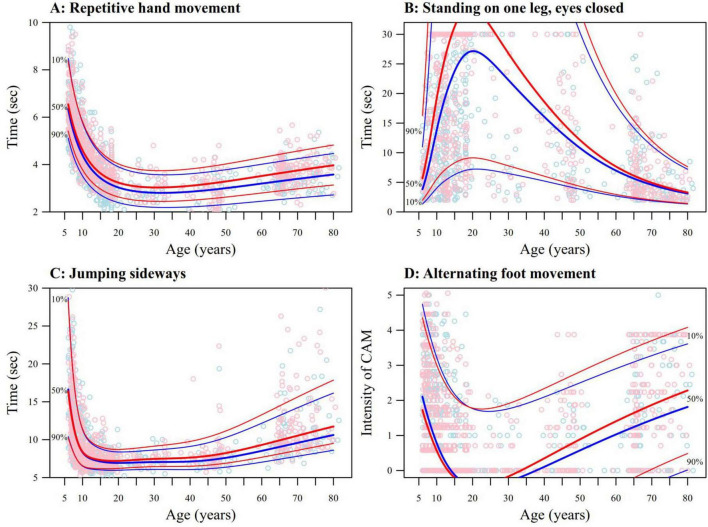
Percentile curves (10%, 50%, and 90%) and data points displaying the changes over age for males (blue) and females (red) in **(A)** pure motor – repetitive hand movements, dominant hand; **(B)** balance – standing on one leg, eyes closed, non-dominant leg; **(C)** gross motor – jumping sideways; the amount of jumps in the 65–80 year olds was multiplied with two; and **(D)** quality of movement – contralateral associated movements (CAMs) during alternating foot movements, non-dominant side active.

In Figure 1, FM functions are illustrated through the pegboard task (dominant side). A fast increase in motor performance can be seen in both sexes, especially below 10 years. During adolescence, the functional improvement slows down, reaches a maximum in the early thirties followed by a slow but continuous deterioration from the early forties onward. Figure 2 displays the evolution of motor functions for selected tasks loading on several motor components (PM: repetitive hand movement, dominant hand; BA: standing on one leg with eyes closed, non-dominant leg; GM: jumping sideways; and CAM: during alternating foot movements, non-dominant side active). A fast increase in motor performance is also seen in these tasks. However, the onset of aging and the speed of the deterioration in motor function is quite variable from one task to the other. Detailed figures with sex specific percentile curves for all tasks are shown in Supplementary materials 3, 4. Overall, it seems that once peak performance is reached, PM functions remain relatively stable and only suffer a mild decline with increasing age, while FM functions decrease faster. In contrast, the deterioration in motor function following peak performance appears larger in BA and GM, with motor functions in the elderly reaching comparable levels to those of the 6 years olds. The intensity of CAM also seems to increase in some tasks (e.g., alternating foot movements) while others remain relatively unaffected (e.g., pegboard).

Figures illustrating the gap between the 50th centile of males and females at each age are provided in Supplementary material 6. They also report the *p*-value of the likelihood ratio test comparing models with and without sex differences. A table summarizing important findings from these figures is available in Supplementary material 7. Virtually all tasks featured a statistically significant sex difference, especially at younger ages. With the exception of standing long jump where males did perform much better than females, the magnitude of the sex difference in other tasks was small when compared to the (sex-adjusted) between-subject variability depicted on the norms. In FM, females performed consistently better than males in pegboard and beads, while males usually performed better in the bolt task (though only after 50 years on the dominant side). In PM, males were performing better on all repetitive tasks while females performed better on alternating and sequential tasks. The only exception was alternating hand movements, where no significant sex difference could be observed on either side (*p* ≥ 0.181). Females performed better on all BA tasks, although the difference was only statistically significant during childhood. Furthermore, males were consistently better on GM tasks. As children, the boys generally had more CAMs than girls of the same age. However, as adults the differences became largely insignificant for most items, persisting only on sequential finger movements (dominant hand), a task that females also performed faster than males. For further details on the differences in sex on the tasks, refer to Supplementary materials 4, 6, 7.

### Age of peak performance

**FIGURE 3:**
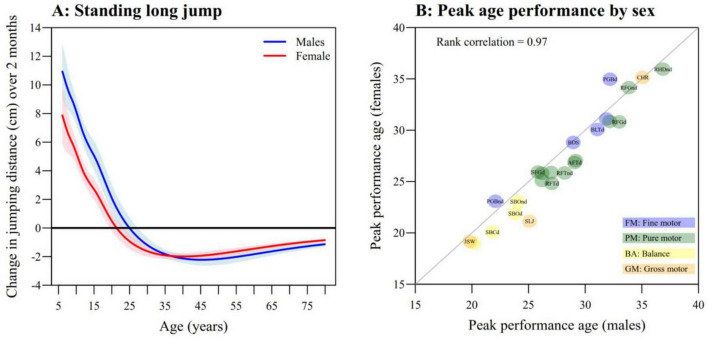
Age of peak performance. **(A)** First derivative or slope of 50% centile with 95% confidence interval for standing long jump as a function of age, for males (blue) and females (red). The *y*-axis value refers to the expected change in jumping distance that occurs over a 2 months’ period (i.e., ±1 month around each age). The age of peak performance is reached when the curve crosses 0. **(B)** Peak performance age for males (*x*-axis) and females (*y*-axis) for all tasks. Rank correlation between ages of peak performance in males and females for timed performance/jumping distance. For abbreviations see Table 2; task labels BLTnd, RHDd, AFTnd, AHDd/nd, SFGnd, and SBCnd are not shown for readability.

Because motor performance increases rapidly during childhood and decreases in old age, it is of particular interest to identify the age at which motor performance reaches its peak. This peak age is defined as the point where the first derivative of the 50% centile curve equals zero, and lies between 20 and 25 years for standing long jump (Figure 3A). Plots of the first derivative of the 50th centile curve for all tasks are provided in Supplementary material 8. Overall, the age of peak performance lies between the early twenties and the mid-thirties. However, maximum performance in most BA and GM tasks appears to be reached first, approximately between 20 and 25 years, whereas performance in other motor dimensions still improves for a few more years. Excepting pegboard, males appeared to reach peak performance at the same age or slightly older than did females on all other tasks. The median difference in peak performance age between males and females was +1 year (interquartile range 0–2 years). Additionally, the rank correlation between the peak performance ages in males and females across the tasks was remarkably high at 0.97 and 0.96 for timed performance or jumping distances and for CAMs, respectively (Figure 3B). For further details about the timing of peak performance and associated sex differences, see Supplementary material 8.

### Effect of BMI on ZNA-2 components

**FIGURE 4:**
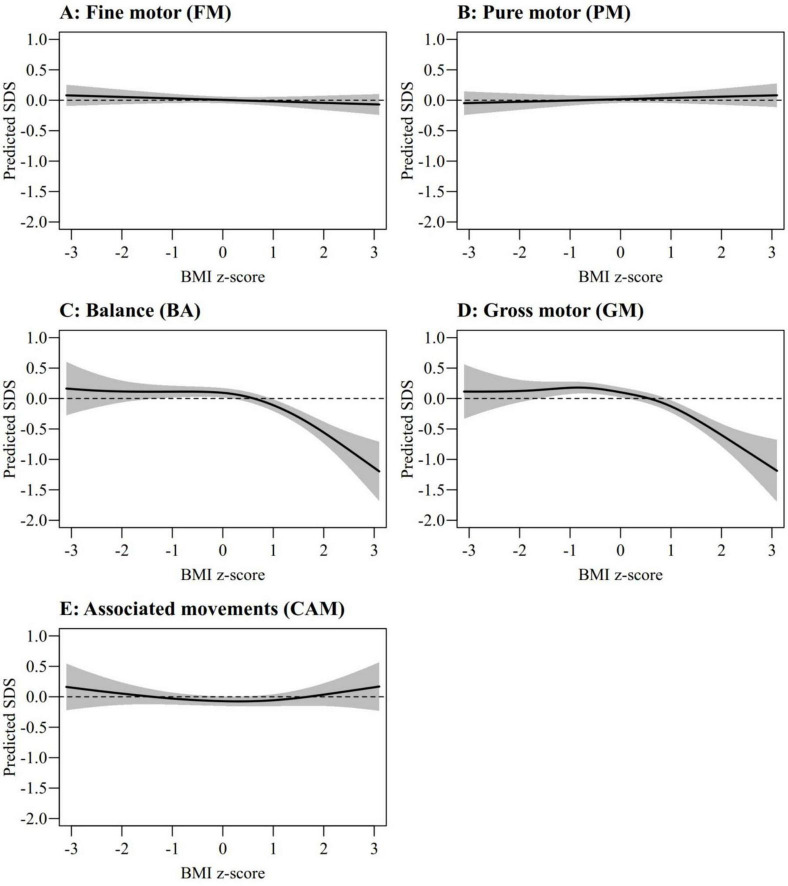
Predicted effects of BMI SDS on fine motor (FM, **A**), pure motor (PM, **B**), balance (BA, **C**), gross motor (GM, **D**), and contralateral associated movements (CAMs, **E**). The shaded areas correspond to the 95% confidence intervals.

The BMI of our sample increases during youth and adolescence conforming to the Swiss and WHO norms on weight and height. After 45 years, the 50th percentile for BMI remained stable for females at 23.3 kg/m^2^, but it still increased slightly in males, from 24.8 kg/m^2^ at 45 years to 25.5 kg/m^2^ at 80 years. At 45 years, percentiles 84.1% and 97.7% of BMI (corresponding to +1 and +2 SDSs) were equal to 29.2 and 36.7 for males, and 27.5 and 34.6 for females, respectively. As shown in Figure 4, motor performance in FM, PM, and CAM was not significantly associated with BMI. BA and GM showed no BMI effect on performance up to a BMI of approximately +1 SDS (equivalent to percentile 84.1%). However, a higher BMI had a deleterious impact on both BA and GM performance.

### Comparison with other studies

**FIGURE 5:**
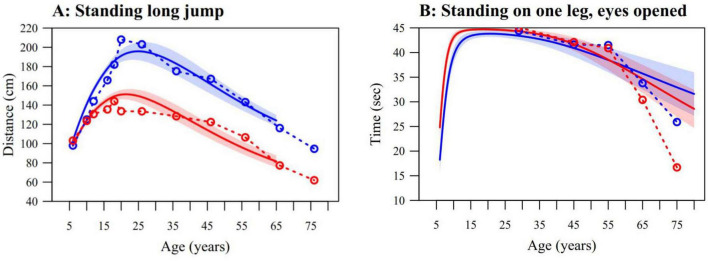
**(A)** Median curves of the standing long jump from this study (solid lines, with pointwise 95% confidence interval) compared to the data from the online calculator of the McKay et al. (2017) study (dashed lines). Note that our norms stop at 65 years because participants in the ZNM-p did not perform the standing long jump. **(B)** Average time standing on one leg with eyes open (dominant side) from this study (solid lines, with pointwise 95% confidence interval) compared to the data from the Springer et al. (2007) study (dashed lines). Our results were obtained by simulating timed performance from the balance model and then censoring the simulated values at 45 s to ensure comparability with the Springer et al. (2007) study. Males in blue, females in red.

For the standing long jump, the data presented here are nearly identical with the data gathered by McKay et al. (2017), which are integrated and displayed in Figure 5A. Small differences can be detected, but these differences are mostly due to how the data were handled: McKay et al. (2017) calculated the average jumping distance within distinct age groups, whereas our modeling approach considered age as a continuous variable. Although Springer et al. (2007) did not assess balance during childhood, their adult balance data show similar results to ours between 25 and 60 years, as seen on Figure 5B. Some differences can be seen in the elderly, where the US sample performed worse than the Swiss but still showed the same sex effect; the performance in females dropped earlier. Their mean BMI (18–99 years) was 27.07 compared to a mean BMI (18–80 years) of 23.54 in this study.

## Discussion

This study is the first to present percentile curves for several neuromotor functions between 6 and 80 years of age for both sexes. The changes across the lifespan were investigated in tasks measuring FM, PM, BA, and GM functions and CAMs.

The findings confirm significant functional improvements in young children, a slowdown during adolescence and adulthood, and a decline in older ages (Haywood and Getchell, 2014; Arking, 2006). Notably, PM and FM tasks show pronounced improvements during youth, with peak performances occurring between 25 and 35 years of age. BA and GM tasks also exhibit a marked increase during childhood and adolescence mostly reaching peak performance in the early twenties followed by a decline. These changes are particularly evident in balance tasks, which depend on multiple body systems that evolve with age. Beside muscular strength and coordination, proprioceptive, visual, vestibular, and auditory perceptual systems all contribute to balance function (Haywood and Getchell, 2014). Each of these systems undergoes specific aging processes. A decrease in muscle mass causes a deterioration in the capacity to stand upright (Haywood and Getchell, 2014; Springer et al., 2007). At greater ages, a decline in force variability amplitude hinders rapid adaptations to balance corrections when standing on one leg (Brunner et al., 2007). And finally, older adults become increasingly dependent on their visual system for balance, shown by the fact that BA with eyes closed deteriorates more rapidly than BA with eyes open, as was also shown by Springer et al. (2007).

For the GM tasks of jumping sideways, chair rising, and standing long jump, the increase in performance during childhood and adolescence is attributed to the increase in muscle mass in adolescents, especially in males. Peak performances in jumping tasks are attained at early adult age and earlier by females than by males: Peak performance in jumping sideways occurs at 19 years for females and 20 years for males, and in standing long jump at 21 years for females and 25 years for males (see Figure 3B). The subsequent decline in performance can be attributed to a reduction in the size of the fast muscle fibers (Hepple and Rice, 2016; JafariNasabian et al., 2017; Deschenes, 2004; Brunner et al., 2007). In contrast, the chair rise test starts to deteriorate only after 35 years because the slower muscle fibers start to deteriorate later (Hepple and Rice, 2016).

The late decline in performance of PM and FM in comparison to BA and GM was striking. Additionally, the 50th percentile of PM and FM at old age (around 80) was functionally always better, i.e., faster than at the starting point of 6 years of age. It looks as if – once learnt – performance is preserved on a high level. Presumably the high maintenance of PM and FM is accredited to the lifelong commitment and daily use of the hands and feet. The change of CAMs over age follows a similar course: at the beginning the children (between 6 and 10 years) have a lot of CAMs, they disappear during adolescence and during aging they reappear, but are less abundant than in the young children (Kakebeeke et al., 2023).

Tasks on which females are always better than males are the pegboard, beads, and sequential finger movements. Tasks on which males are always better than females are repetitive movements of hands, fingers, and feet. The consistency in the sex difference observed between repetitive PM tasks and sequential PM and FM tasks suggests that these functions are probably linked to biological sex. This also applies to movement quality; females always have fewer CAMs than do males except on the bolts task. In BA, females always perform better than males until adolescence. At older ages, sex differences could not be reliably estimated due to ceiling effects and/or a sparsity of data. In the GM tasks, males always perform better than the females, which can be attributed to the males having more muscle mass (10%) than the females (Bredella, 2017). However, the differences between the sexes are generally small in comparison to the high variability within the sexes, yet they remain highly statistically significant, which is why we present sex-specific percentiles.

The study also examines age and sex differences by reporting the age of peak performance for various tasks. Males mature on average 1 year later than females in all the functions, as shown by the high rank correlation (*r* = 0.97) between the ages of peak performance in males and females. Males also exhibit more CAMs than females of the same age, thus confirming that females mature earlier (Tanner, 1978; Tanner et al., 1983; Thomas and French, 1985; Haywood and Getchell, 2014).

Although higher BMI did not significantly affect PM, FM, or CAM performances, it negatively impacted BA and GM at BMI > 1 SDS. The deleterious effect of higher BMI on motor performance in antigravity tasks emphasizes the importance of maintaining a healthy weight from an early age to support motor performance (Chivers et al., 2013; Haywood and Getchell, 2014).

Some limitations need to be considered when interpreting these curves. Despite pooling data from 11 studies, resulting in the analysis of motor measurements from 1,620 individuals, the density of data is not uniform. Many data points are available for the developmentally dynamic phases, whereas for ages from 25 to 65 years, the data density is lower (Figures 1, 2). Further, we focused on obtaining smooth reference curves for motor functions over the whole lifespan and used a strong smoothness penalty (BIC) to model the age trend. This resulted in smoothed percentiles that provide an acceptable good fit globally, but may occasionally lack precise fit within some age ranges. Also, although the age of peak performance is an interesting metric with which to compare the speed of maturation between males and females, it is not well defined on tasks in which the degradation in motor performance due to aging is not pronounced. Consequently, its estimation may be imprecise.

The representativeness of data for neuromuscular function across the lifespan is often discussed, and several recruitment biases have been acknowledged, especially in elderly populations (Banack et al., 2019; Enzenbach et al., 2019). The motor measurements performed on children and adolescents from the Zurich area were obtained from diverse samples stratified by socioeconomic status (SES) (Kakebeeke et al., 2018; Largo et al., 2001b; Largo et al., 2001a). Adults around 45 years and around 65 years were characterized in detail and shown to be representative of the Swiss population (Kakebeeke et al., 2023). However, recruiting elderly participants poses challenges because healthier individuals are substantially more likely to volunteer, leading to a selection bias. In particular, the ZNA-p subsample included former employees of the University Children’s Hospital Zurich who were physically mobile. Due to the inclusion criteria this subpopulation is not completely representative of the general Swiss population at the same age. Nevertheless, the ZNA-p subsample aligned with the findings from the Swiss Health Survey (Bundesamt für Statistik, 2022), especially on BMI distribution (see Supplementary material 1). In contrast, representativity with respect to SES is more difficult to assess because the number of years of education, a proxy for SES in the elderly, shows large disparities between males and females. In fact, no single definition of SES applies from childhood to old age. Therefore, it was not possible to investigate any SES effect on motor performance across the wide age range covered in this study.

Despite these limitations, the neuromotor data presented in this study are suitable to serve as normative standards. In fact, comparisons with the reference values reveal similarities in key motor domains such as BA and GM (e.g., McKay et al., 2017). Some differences are likely due to varying protocols. The difference in BA in the elderly may be attributed to the higher BMI in the US sample (Springer et al., 2007), which is in line with our findings about high BMI effects. Thus, our findings reflect typical motor development across the lifespan, and the curves may be used beyond Switzerland.

The percentiles for neuromotor functions across the lifespan presented here may serve several of the purposes suggested by Burton and Miller (1998) and Burton and Rodgerson (2001). For example, the differing changes over the lifespan exhibited by GM and FM functions indicate which functions are more prone to aging than others. For clinicians, the norm data and percentile curves support norm-based diagnostics that allow individual motor performance in one or more domains to be interpreted relative to age-specific reference values. In fact, significantly below-average values (<10th percentile) are of clinical importance, as they may indicate functionally meaningful impairments. For example, in children and adolescents, one of the key diagnostic criteria for the diagnosis of developmental coordination disorder (DCD) is performance below a cut-off on a norm-based assessment (Kakebeeke et al., 2018), for which the ZNA-2 is used. We also assume that the norms could be clinically valuable in older adults, especially in the age group over 65 years, for estimating the risk of falling or determining the indication for therapeutic interventions and assistive devices in the case of FM impairments. Moreover, tracking a decline in motor performance over time may provide diagnostic insights into emerging neurological conditions. To distinguish such disease-related decline from the normal age-related changes in performance, sex- and age-specific percentile curves, as shown in Supplementary material 3, are essential.

## Conclusion

This article presents percentiles for 14 neuromotor tasks of the ZNA-2 performed by participants from 6 to 80 years. Performance on most tasks featured an increase in motor functions during childhood followed by a decrease in performance during adulthood. The performance decline started in the early twenties; it was more pronounced in balance and GM functions but began later and was less pronounced in FM and PM functions. Particular precautions should be taken in judging the neuromotor performance of individuals with high BMI (>percentile 84.1% or >1 SDS) because the impact of gravity on weight-sensitive functions overrides motor function.

## Data Availability

The original contributions presented in this study are included in this article/Supplementary materials. Further inquiries can be directed to the corresponding author.
