# Supplementary material for: Neuromotor functions across the lifespan: percentiles from 6 to 80 years
Source: Front Aging Neurosci. 2025 Jul 29;17:1543408. doi: 10.3389/fnagi.2025.1543408 (PMC12340781; doi:10.3389/fnagi.2025.1543408)
Supplement: Supplementary file 1 [file Data_Sheet_1.pdf]

Supplement e1

ZNMp cohort: description of the 65- to 80-year-old participants

For the cohort 65- to 80-year-old 142 (86 females) retired employees of the University Children's Hospital Zurich and their partners were recruited. In this age band a certain selection bias cannot be avoided. Some people of the same age have already died during this period, and volunteers do not register to participate if they suffer from serious chronic illnesses (Banack et al., 2019). Moreover, participants with higher socioeconomic status (SES) and healthier lifestyles are known to be overrepresented (Enzenbach et al., 2019). Special attention was given to the body mass index (BMI), level of activity and training, health status, SES, and mini-mental state both to address the question of representativeness and to analyze the potential effects of these parameters on the measurements. These findings were compared to those of the Swiss Household Survey (SHS) of 2022 (Bundesamt für Statistik, 2022). The SHS is a systematic randomized interview-based survey performed every 5 years since 1992 by the *Bundesamt für Statistik* of the Swiss Confederation. The SHS addresses multiple domains of society with special emphasis on health and well-being; in 2022, it included more than 21,000 interviewees: more than 3,000 per age group of 10 years (relevant age groups 65–74 and >75 years).

Confounding parameter

Body Mass Index (BMI)

To gather the BMI, each participant's weight measured to 0.1 kilogram was divided by the square of their height measured to 1.0 mm. The BMI in both sexes of our participants was constant over the age period; in the participants below 75, it was slightly lower than in the SHS (males -5.1%, females -4.7%), and the BMI of participants older than 75 years was nearly equal (-1.1/-1.3%).

Level of activity/training

According to the definition used in the SHS, the sporting activity was divided into 3 levels (1 = active, 2 = partially active, 3 = inactive), training was recorded in a 4-level classification (1 = trained, 2 = sufficiently active, 3 = partially active, 4 = no training). In our group, sporting activity was approximately constant across the entire age range and was nearly the same for women and men (mean value 1.91). When training, men showed a slight increase in intensity over age (mean value falling from 2.9 to 2.6). Women showed a reverse trend (increasing from 2.5 to 2.9). Compared to the SHS data, our participants between 65 and 74 years were about equally active, and those over 75 were more active. This can be explained by the proportion of frail people with limited mobility, which was higher among the SHS respondents.

Health Status

Similar to the SHS, questions were asked about the presence of chronic illnesses: illnesses that have been present for longer than 6 months and are expected to persist for longer than 6 months. In the 65–74-year-old age group, the frequency of chronic diseases in men and women was very similar to that in the

SHS; in the older group, our men were slightly more affected by chronic diseases and the women were slightly healthier.

Socioeconomic status (SES)

Because our participants had already retired from professional life, the SES was classified by the highest vocational training qualification and was therefore comparable to the SHS (Bundesamt et al., 2022). This uses the following 5 categories: 1 = compulsory school, no vocational training, 2 = vocational apprenticeship, vocational school, apprenticeship, 3 = middle school, vocational baccalaureate, teacher training college, 4 = higher technical school, 5 = university, technical college. Due to the recruitment location, the University Children's Hospital Zurich, the SES distribution, particularly among men, differed from that of the normal population. The mean SES of the ZNA-p compared to the SHS was for males 4.43 compared to 2.90 in the SHS; for females it was 3.16 compared to 2.23 in the SHS.

Mini-mental state assessment

Mini-mental state assessment (Folstein et al., 1975) was performed just before testing began on the cohort of 65 to 80 year olds to exclude any mental problems with the understanding of our test. None of the subjects had to be excluded because of an abnormal score on this test.

Summary ZNM-p

Our sample was comparable to their peers in the SHS in BMI and level of activity and training. The health status of men in older age was somewhat lower, and that of the older women was slightly higher than in the SHS. The greatest deviation from the SHS was seen in the SES of men, which was much higher. None of subjects failed the mini-mental status test.

References

Banack, H. R., Kaufman, J. S., Wactawski-Wende, J., Troen, B. R. & Stovitz, S. D. 2019. Investigating and Remediating Selection Bias in Geriatrics Research: The Selection Bias Toolkit. *Journal of the American Geriatrics Society,* 67**,** 1970-1976.

Bundesamt für Statistik 2022. Schweizerische Gesundheitsbefragung.

Enzenbach, C., Wicklein, B., Wirkner, K. & Loeffler, M. 2019. Evaluating selection bias in a population-based cohort study with low baseline participation: the LIFE-Adult-Study. *BMC Med Res Methodol,* 19**,** 135.

Folstein, M. F., Folstein, S. E. & Mchugh, P. R. 1975. "Mini-mental state". A practical method for grading the cognitive state of patients for the clinician. *J Psychiatr Res,* 12**,** 189-98.
