# Supplementary material for: Neuromotor functions across the lifespan: percentiles from 6 to 80 years
Source: Front Aging Neurosci. 2025 Jul 29;17:1543408. doi: 10.3389/fnagi.2025.1543408 (PMC12340781; doi:10.3389/fnagi.2025.1543408)
Supplement: Supplementary file 2 [file Data_Sheet_2.pdf]

Supplement e2: Difference between ZNA 2 and ZNA testing procedures. Greyed cells indicate tasks that were not performed.

		ZNA 2		ZNA		
**COMPONENTS**	**Items / Age group**	**6 to 65 years**	**65 to 80 y**	**5 to >6.5 y**	**6.5 to <10.5 y**	**10.5 to 60 y**
**Fine motor**	Pegboard	10 pegs		12 pegs		12 pins
	Bolts	1				
	Beads	5				
**Pure motor**	repetitive foot movements	20×		20×		
	alternating foot movements	10×		10×		
	repetitive hand movements	20×		20×		
	alternating hand movements	10×		10×		
	repetitive finger movements	20×		20×		
	sequential finger movements	5×		3×	5×	
**Static balance**	with eyes open	max 30 sec (best of 2 trials)		max 60 sec	max 60 sec*	
	with eyes closed	max 30 sec (best of 2 trials)				max 60 sec
**Gross motor**	jumping sideways over line	20×	10×		10×**	15×**
	chair rise test	10×				
	standing long jump	average of 2 trials				

*holding stick

**rope 20cm above floor

Controlling for changes in testing procedures.
Some adjustments had to be made to some tasks e.g. to accommodate changes between the ZNA and ZNA-2 testing procedures. For example, in the pegboard task, the ZNA used 12 pegs, but only 10 are used in the ZNA-2. Furthermore, the ZNA used plastic pegs for children up to 10.5 years old and thereafter switched to metal pins that had to be turned around, but the ZNA-2 consistently uses plastic pegs with all ages. In the original ZNA, the sideways jumping task involved jumping over a rope set 20 cm above the ground, with participants completing 10 jumps between the ages of 6.5 and 10.5 years and 15 jumps thereafter. In contrast, the ZNA-2 assesses this task over 20 jumps on flat ground without a rope. The resulting norms refer exclusively to the ZNA-2 procedure, with the same number of repetitions at all ages. For example, normative data for the pegboard task are provided as if 10 pegs were used at all ages.
