# Supplementary material for: Neuromotor functions across the lifespan: percentiles from 6 to 80 years
Source: Front Aging Neurosci. 2025 Jul 29;17:1543408. doi: 10.3389/fnagi.2025.1543408 (PMC12340781; doi:10.3389/fnagi.2025.1543408)

Supplement e3

Percentile curves for the different neuromotor functions of the Zurich Neuromotor Assessment-2 (ZNA-2).

The x-axis shows the age from 6 to 80 years; the y-axis shows the time in seconds needed to perform the exercise or the distance achieved in centimeters, the male percentiles at the top, the female percentiles at the bottom of the page, dominant side first, nondominant side second. In consecutive order the following tasks are presented: pegboard, bolts, beads, repetitive foot movements, repetitive hand movements, repetitive finger movements, alternating foot movements, alternating hand movements, sequential finger movements, standing on one leg with eyes open, standing on one leg with eyes closed, jumping sideways, chair rise and standing long jump.

Pegboard (dominant side)

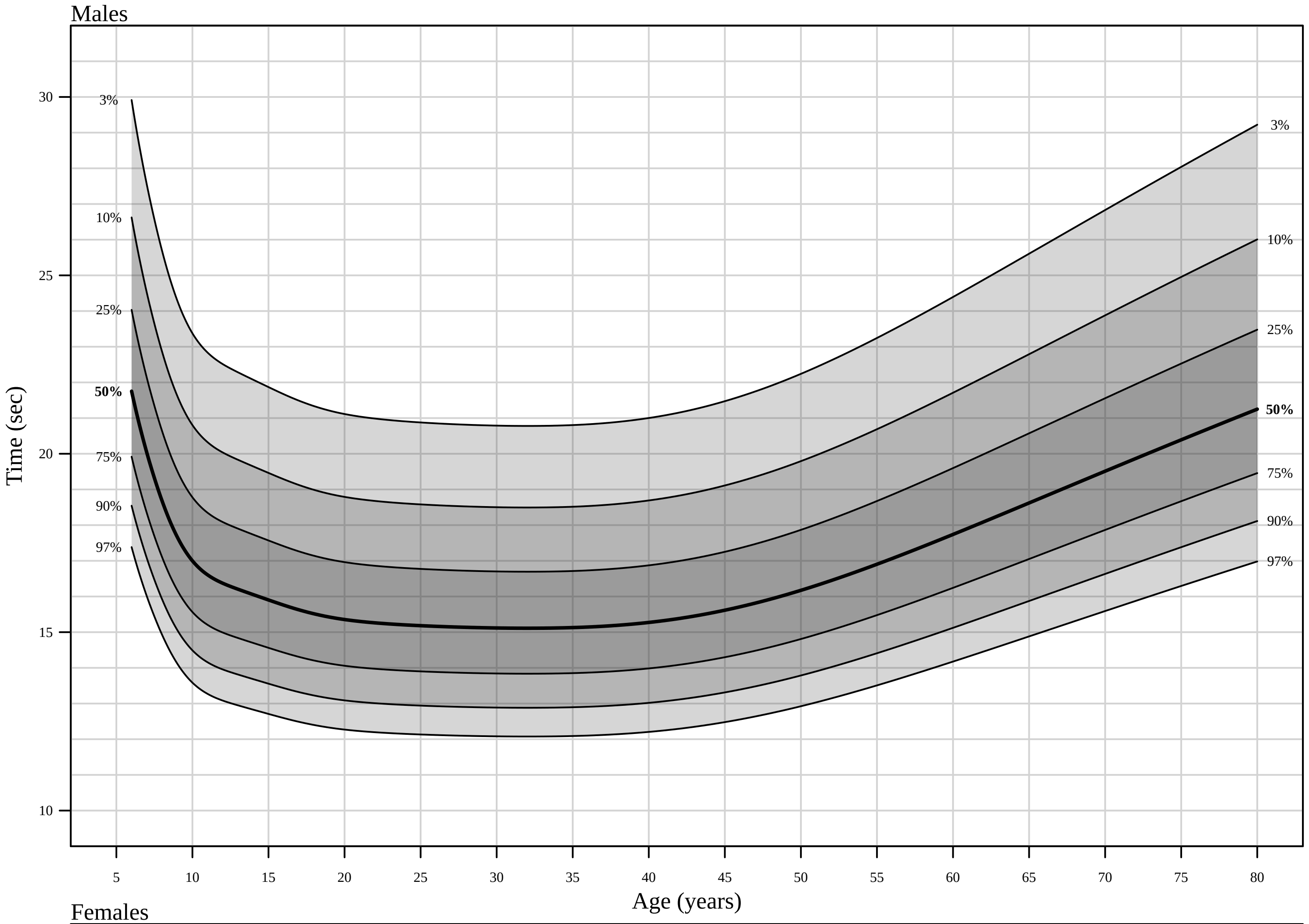

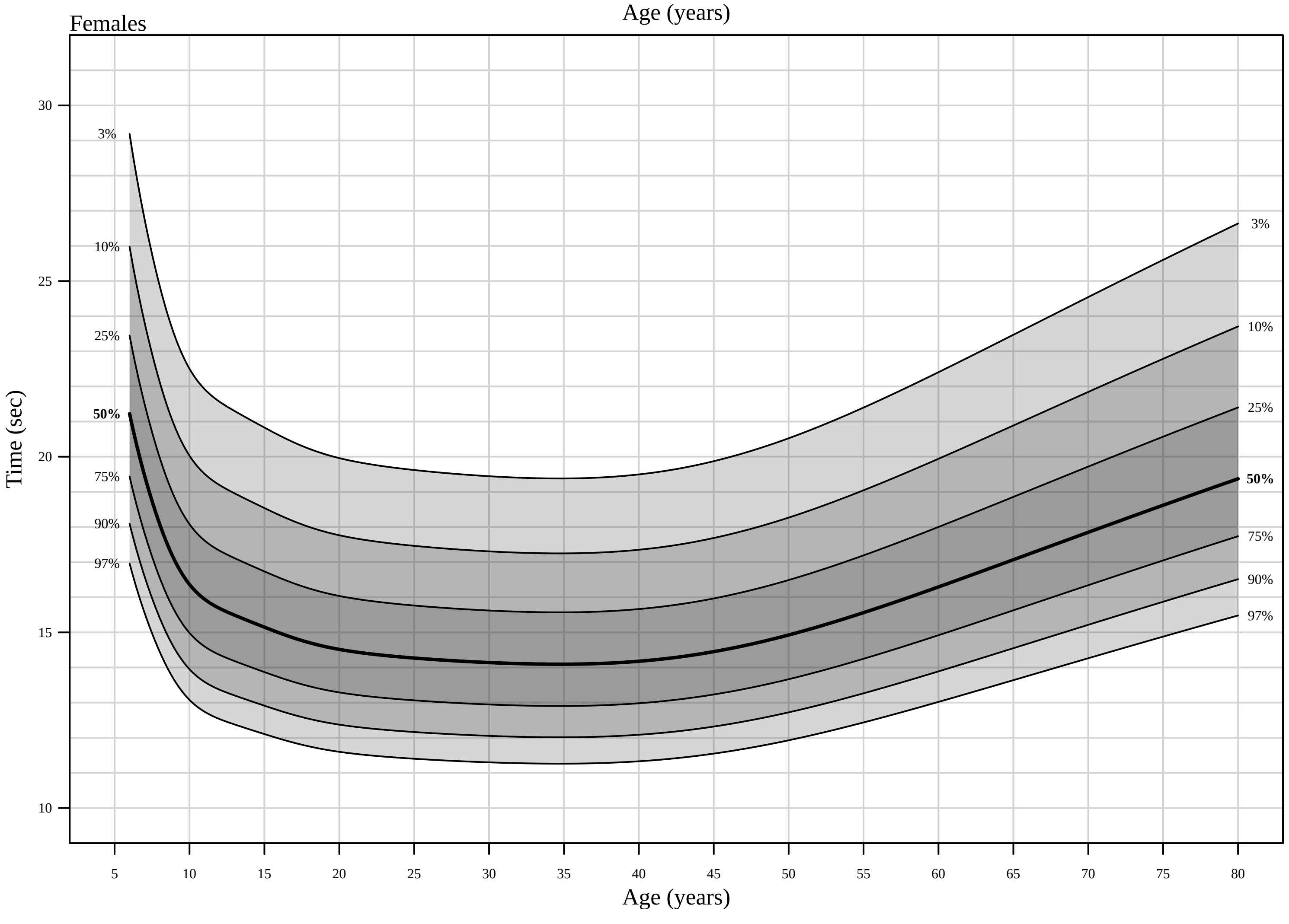

Pegboard (nondominant side)

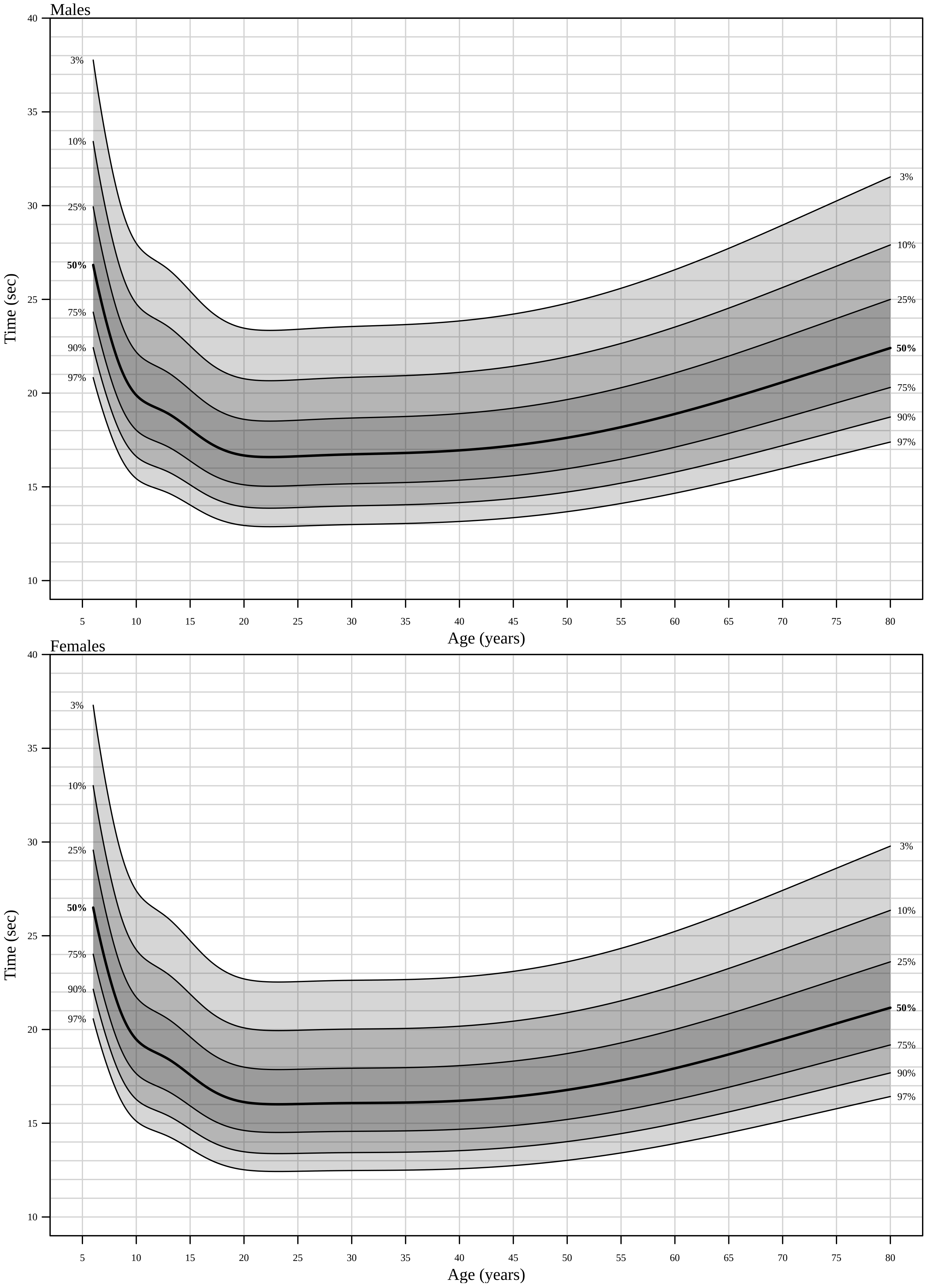

Bolts (dominant side)

Males

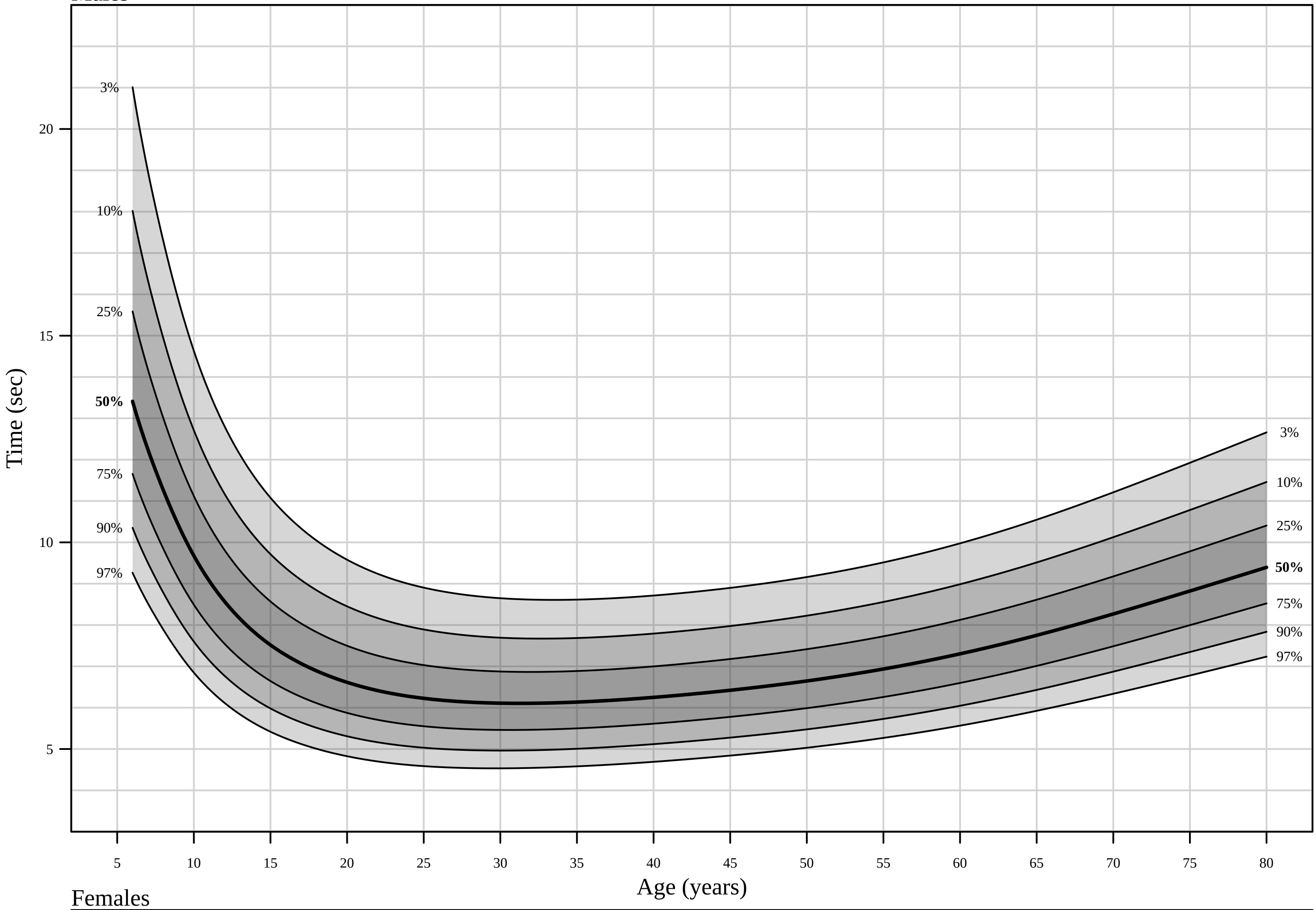

Females

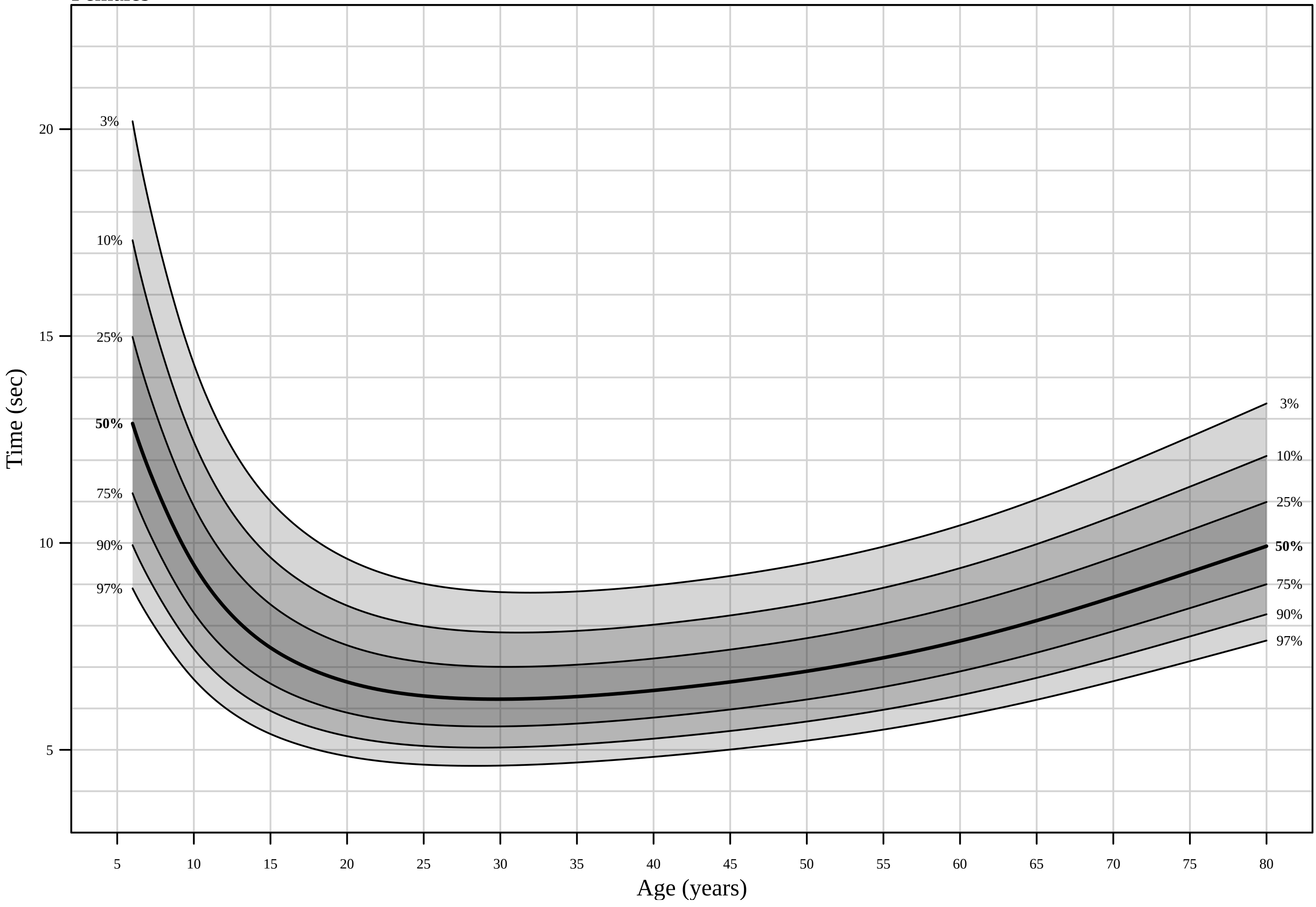

Bolts (nondominant side)

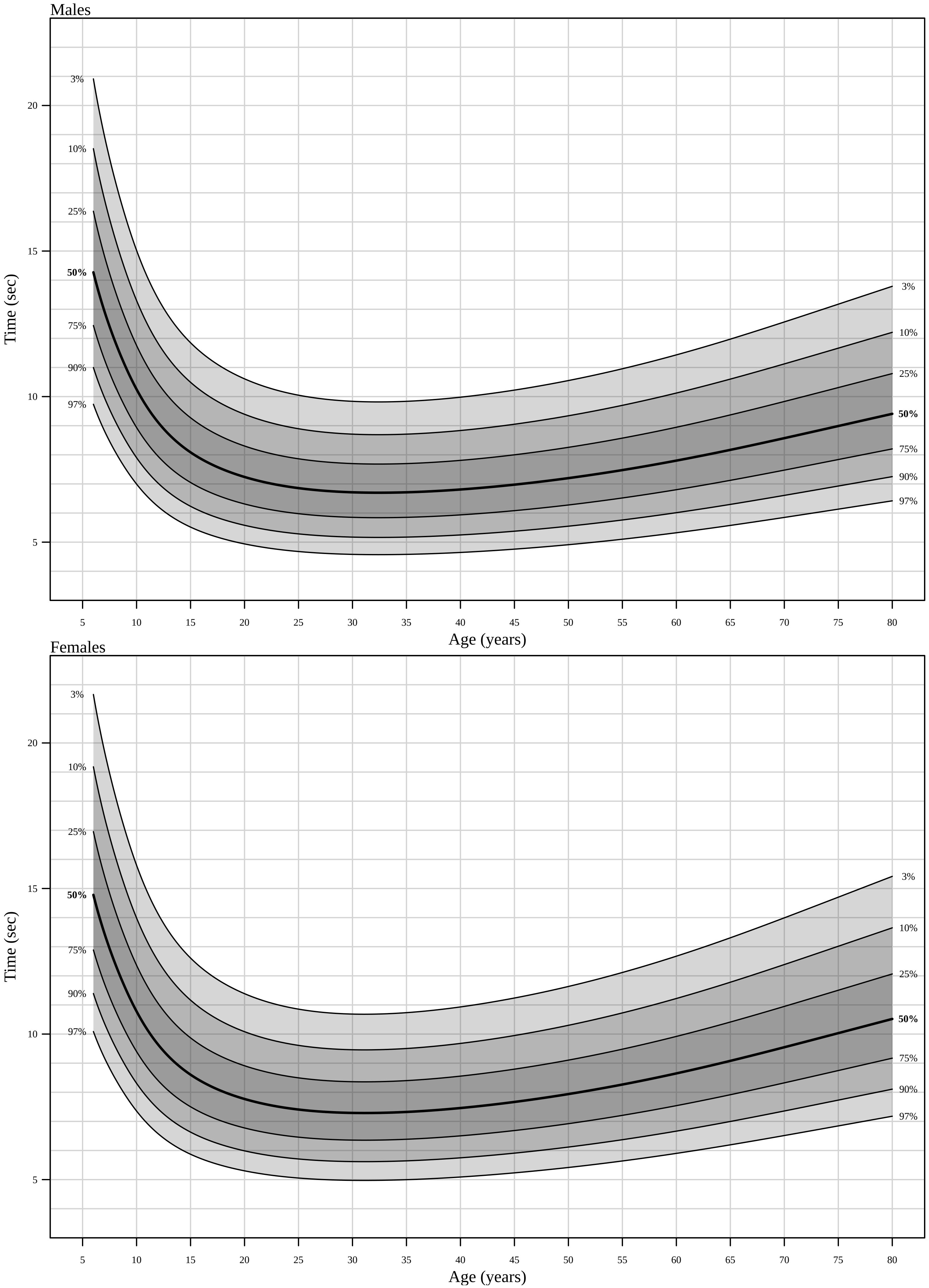

Beads

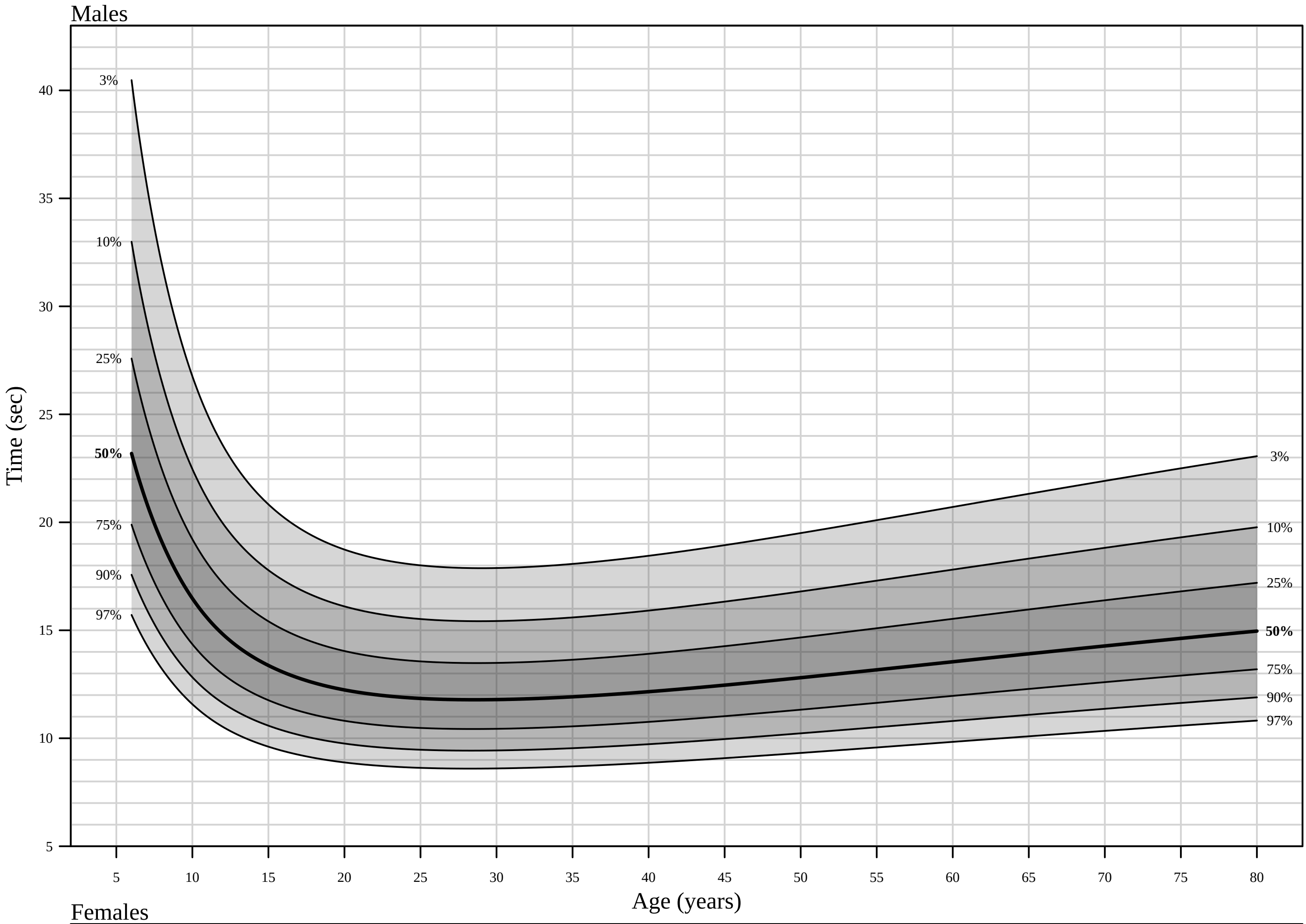
Males
Time (sec)
Age (years)
3%
10%
25%
50%
75%
90%
97%
5
10
15
20
25
30
35
40
45
50
55
60
65
70
75
80

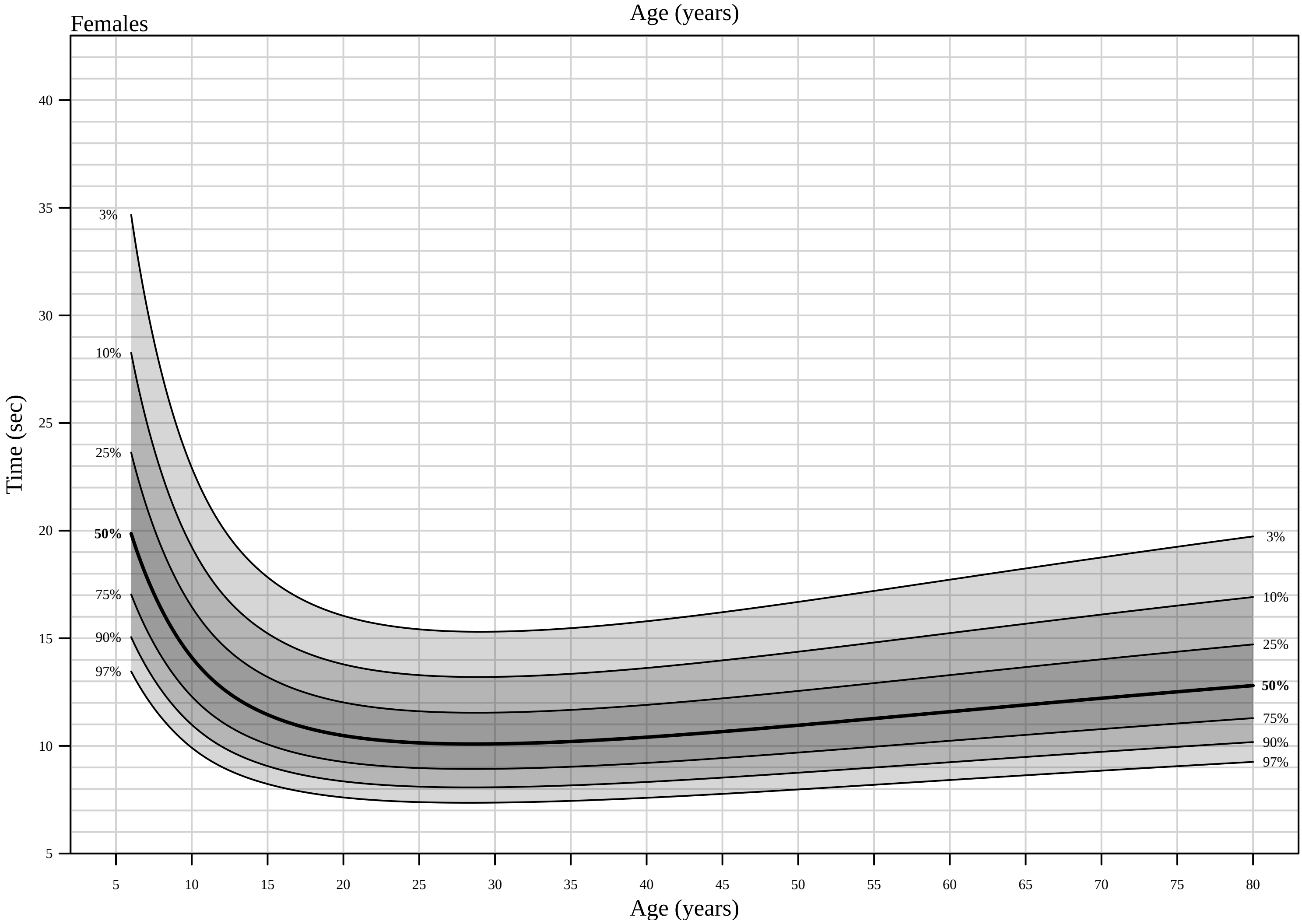
Females
Time (sec)
Age (years)
3%
10%
25%
50%
75%
90%
97%
5
10
15
20
25
30
35
40
45
50
55
60
65
70
75
80

Repetitive foot movements (dominant side)

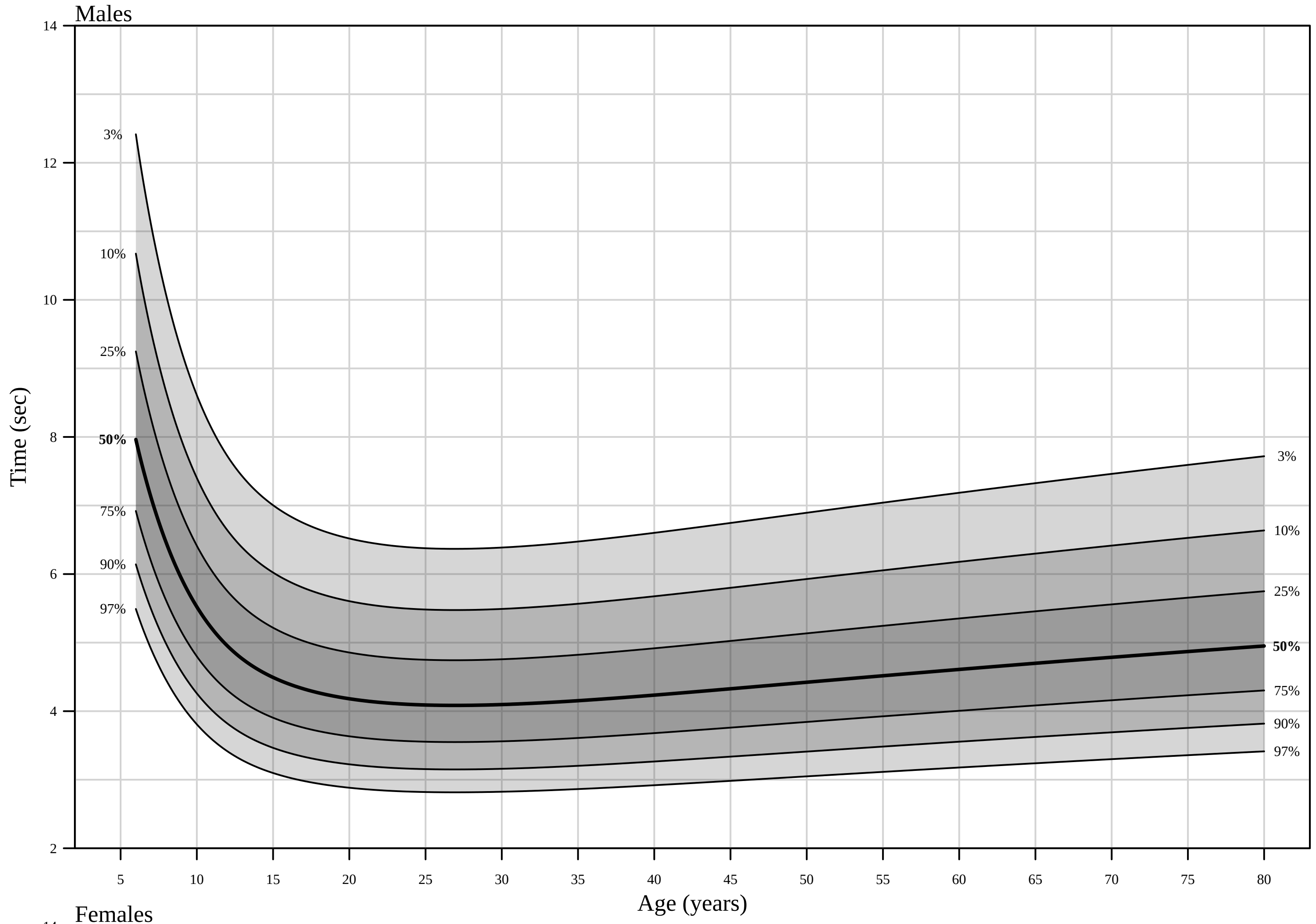

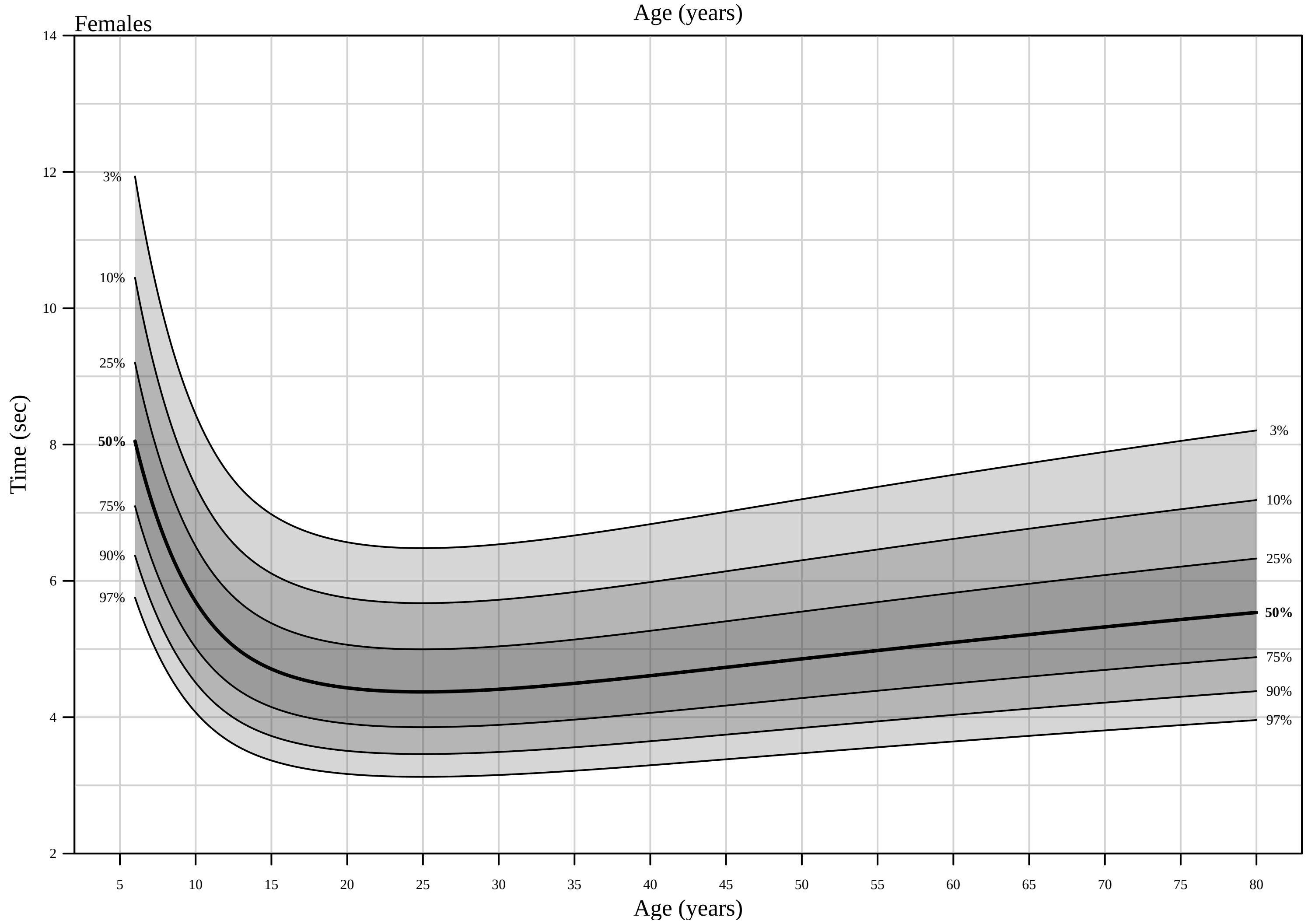

Repetitive foot movements (nondominant side)

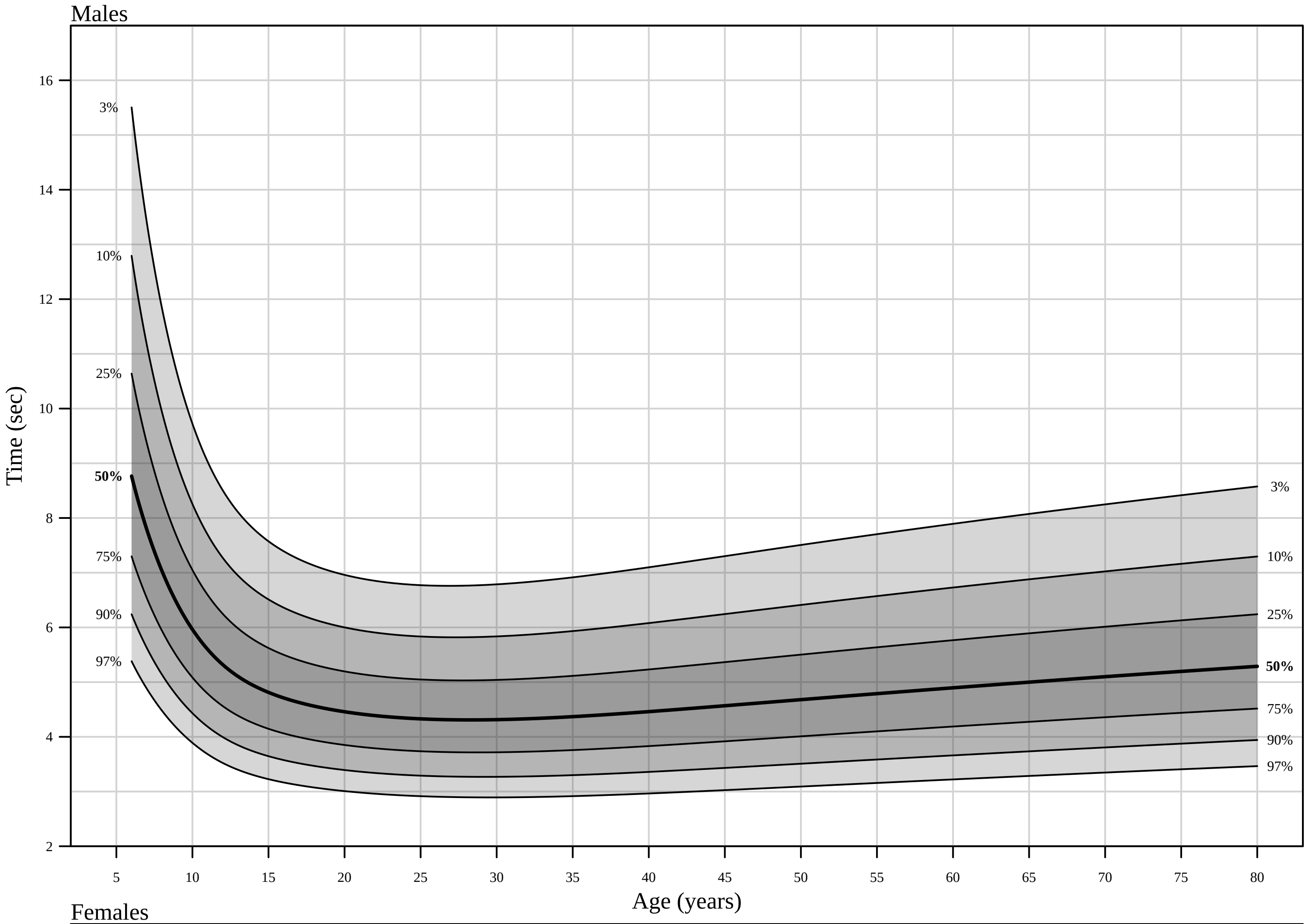

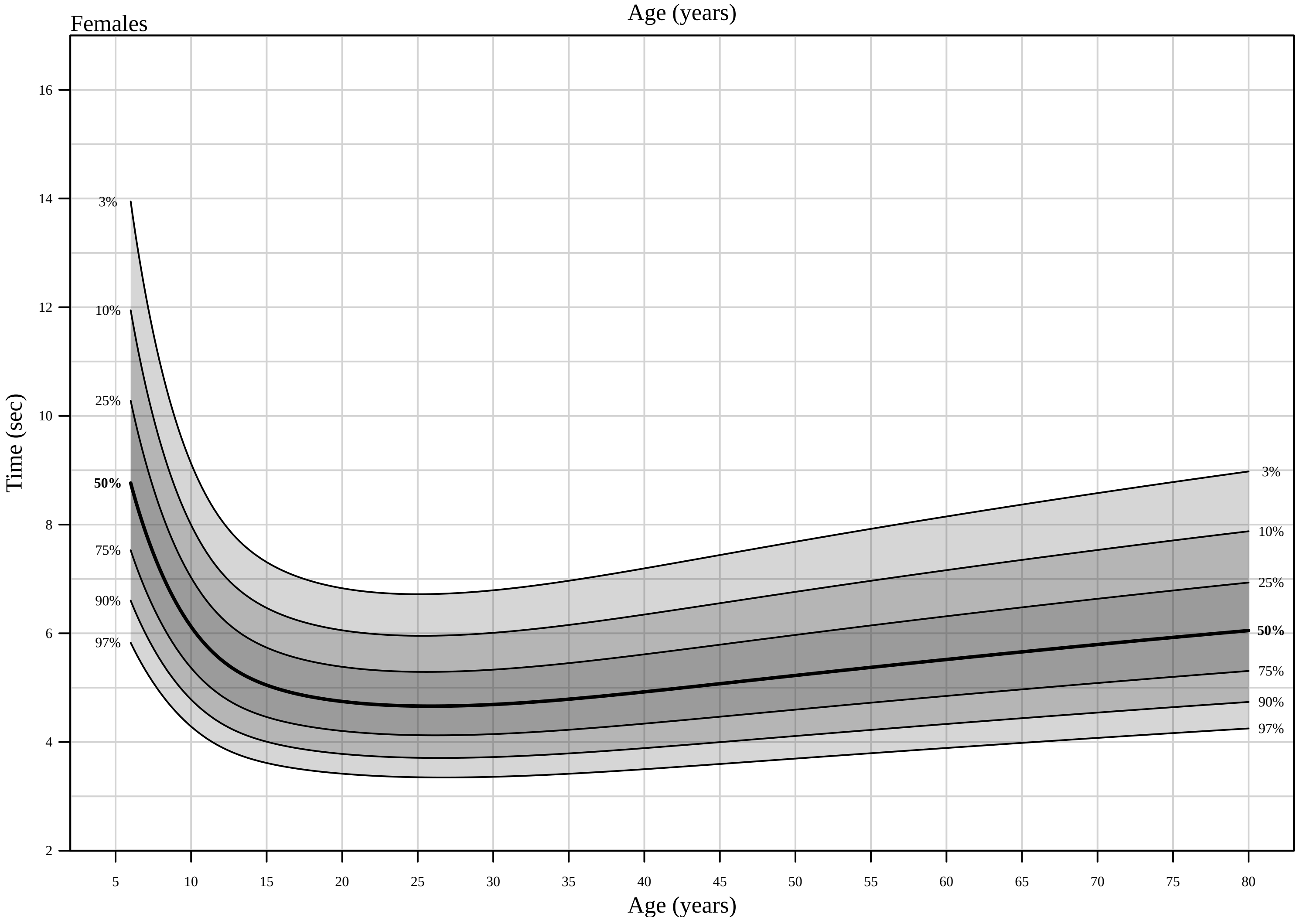

Alternating foot movements (dominant side)

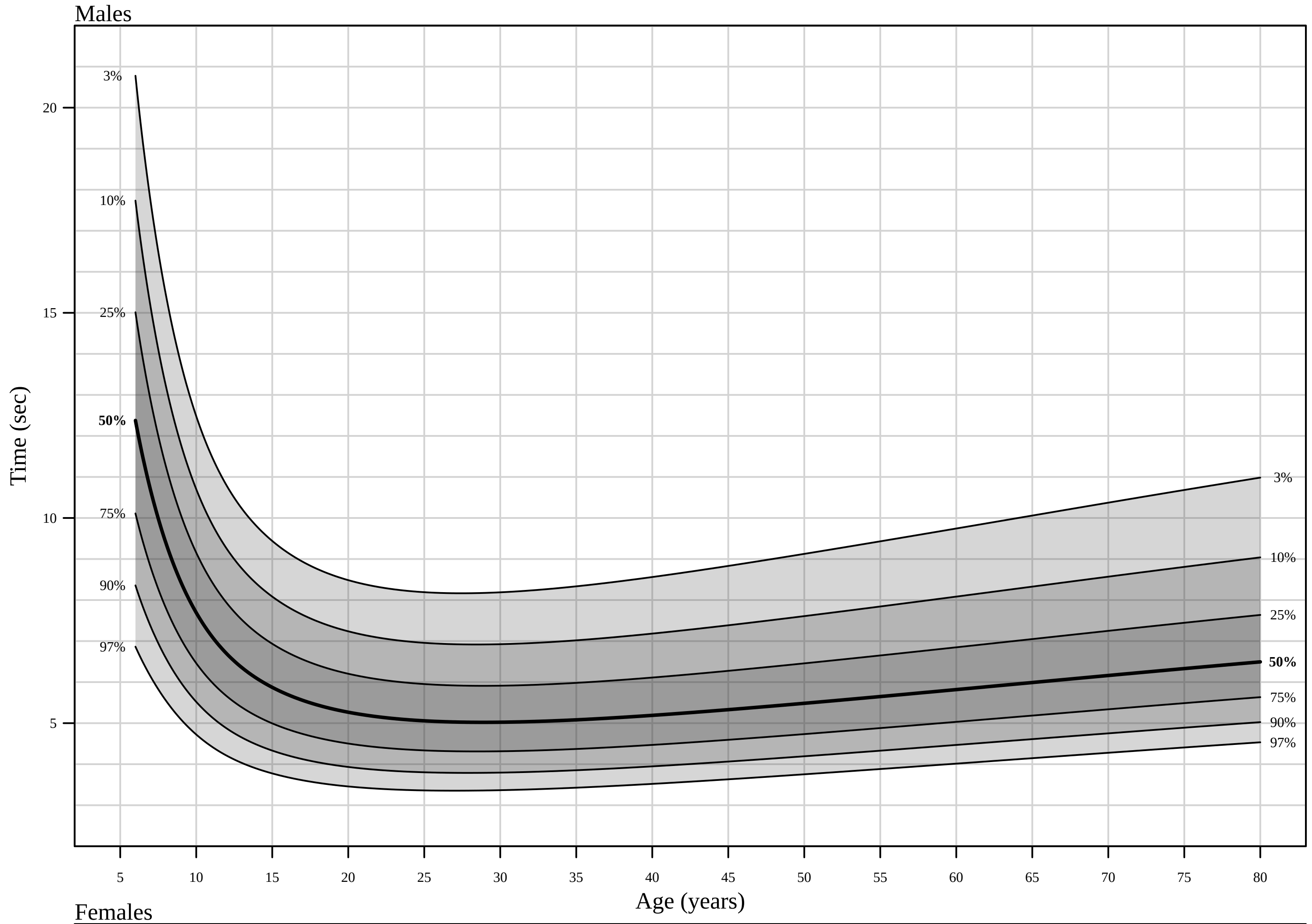

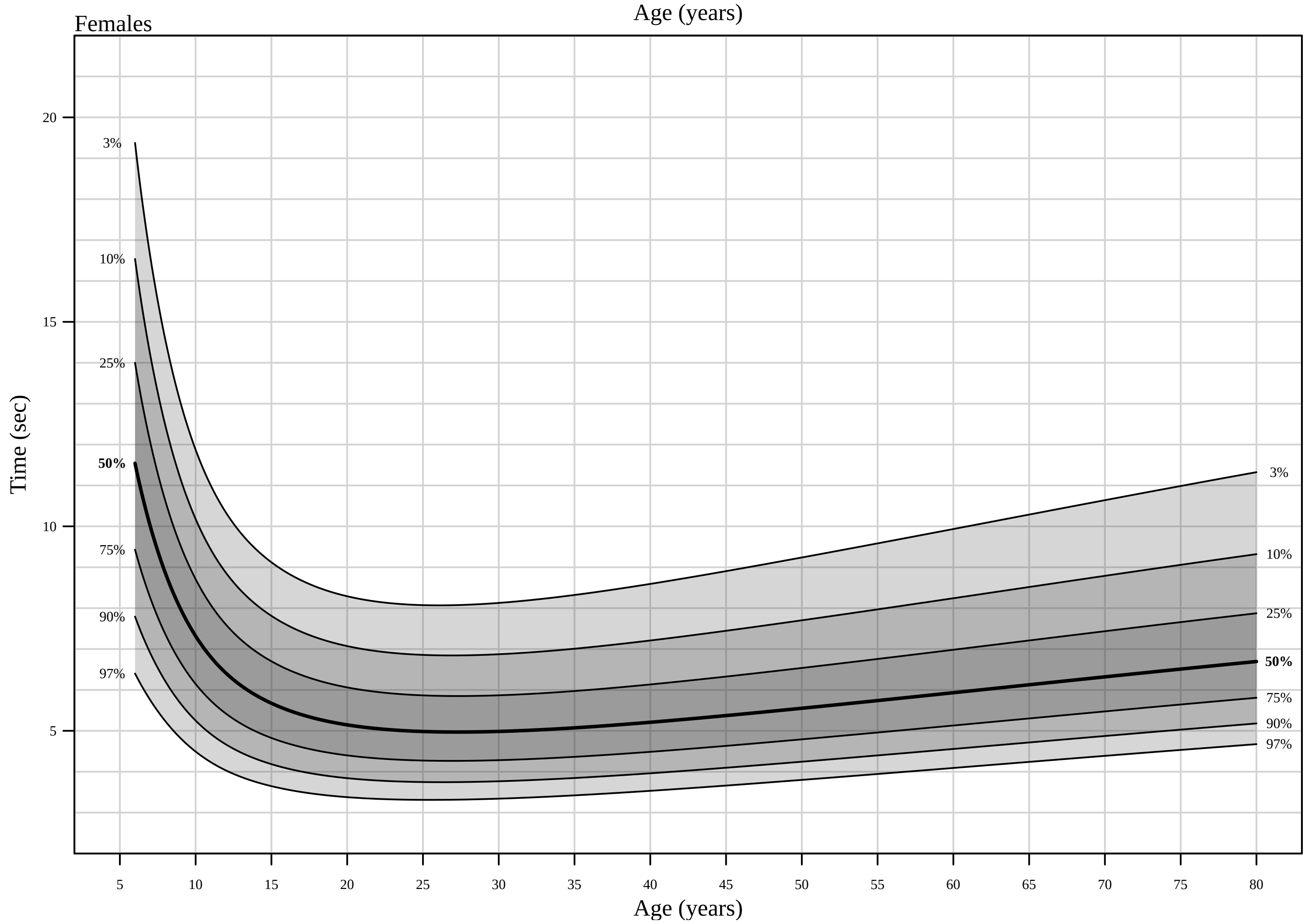

Alternating foot movements (nondominant side)

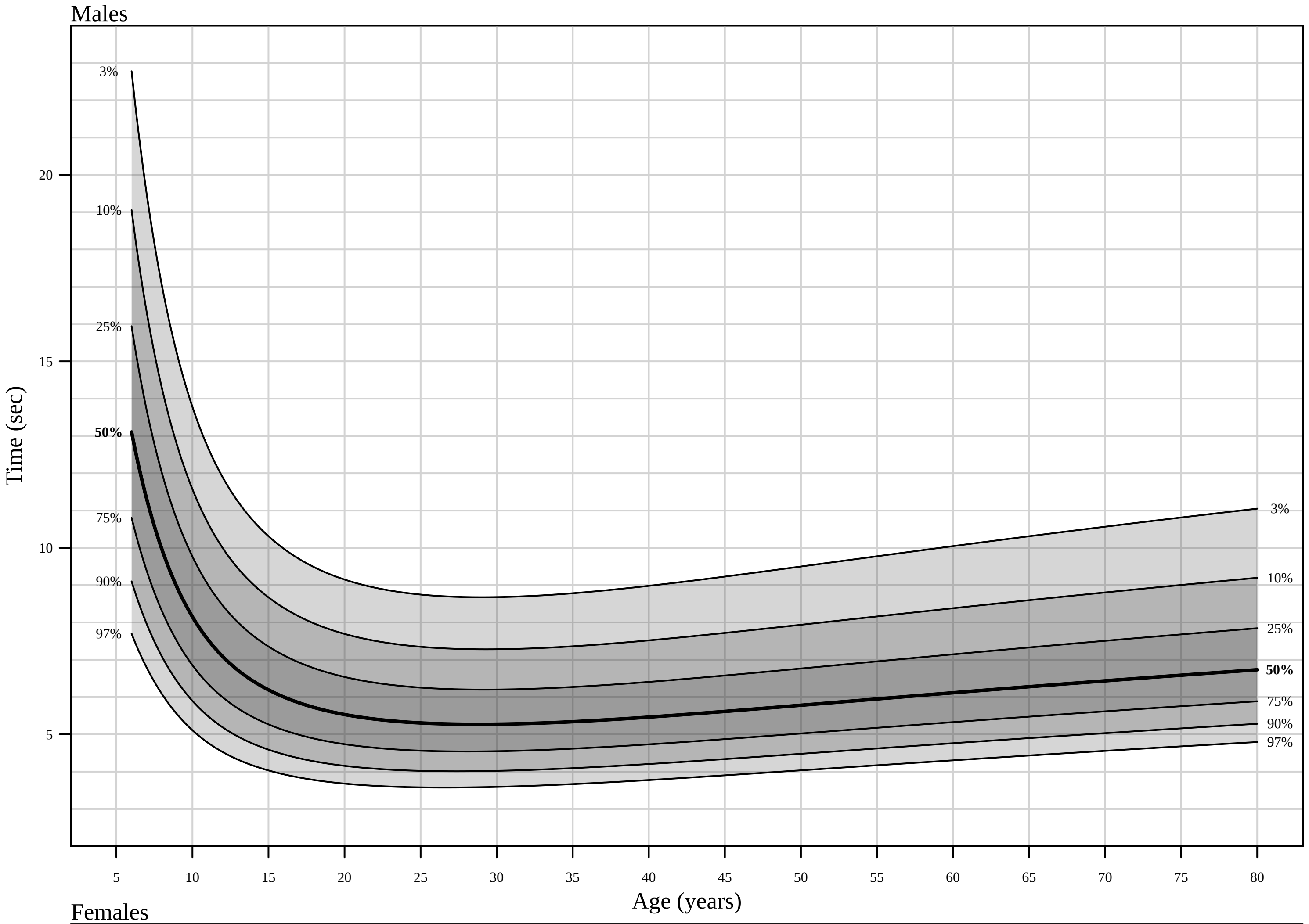

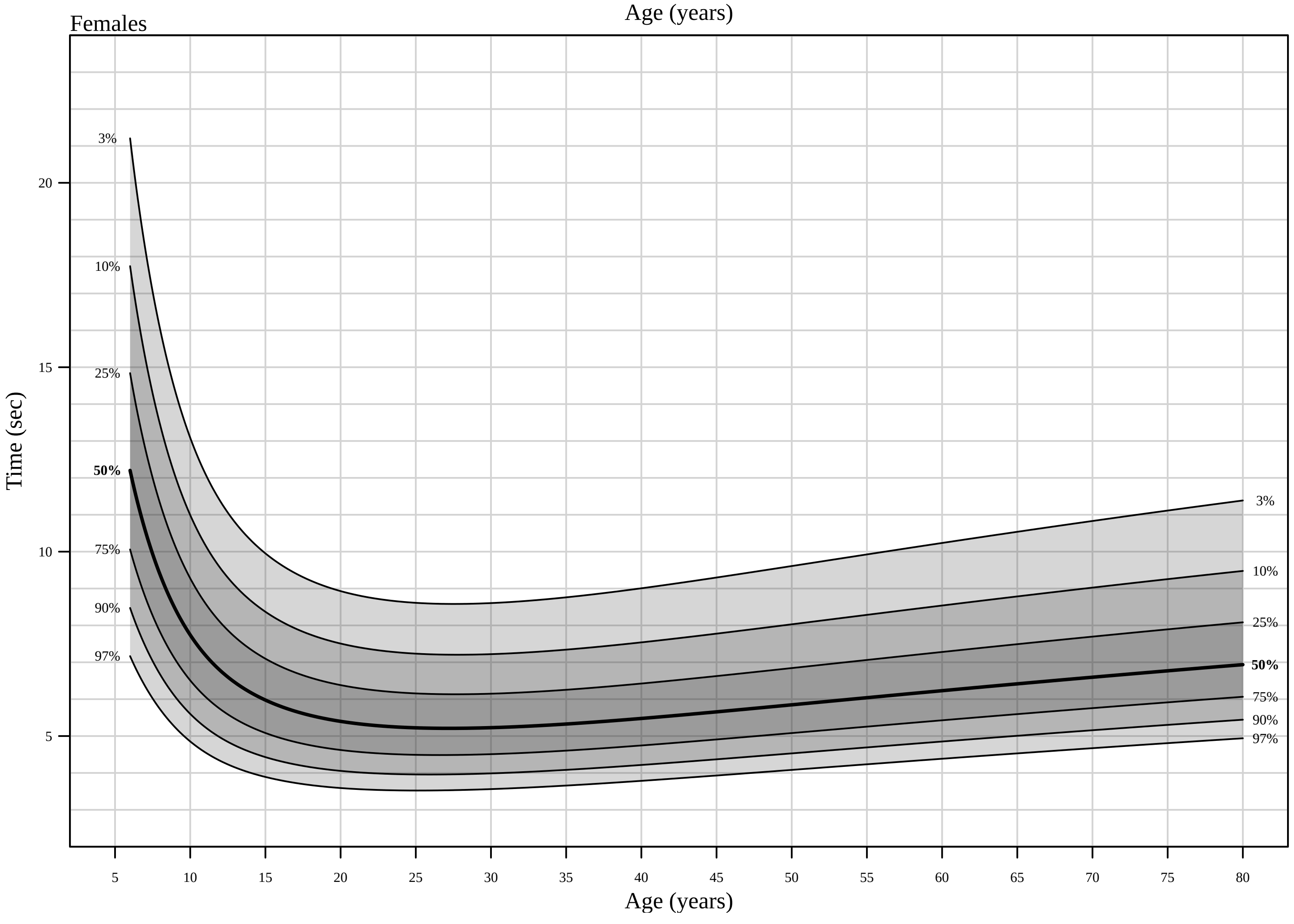

Repetitive hand movements (dominant side)

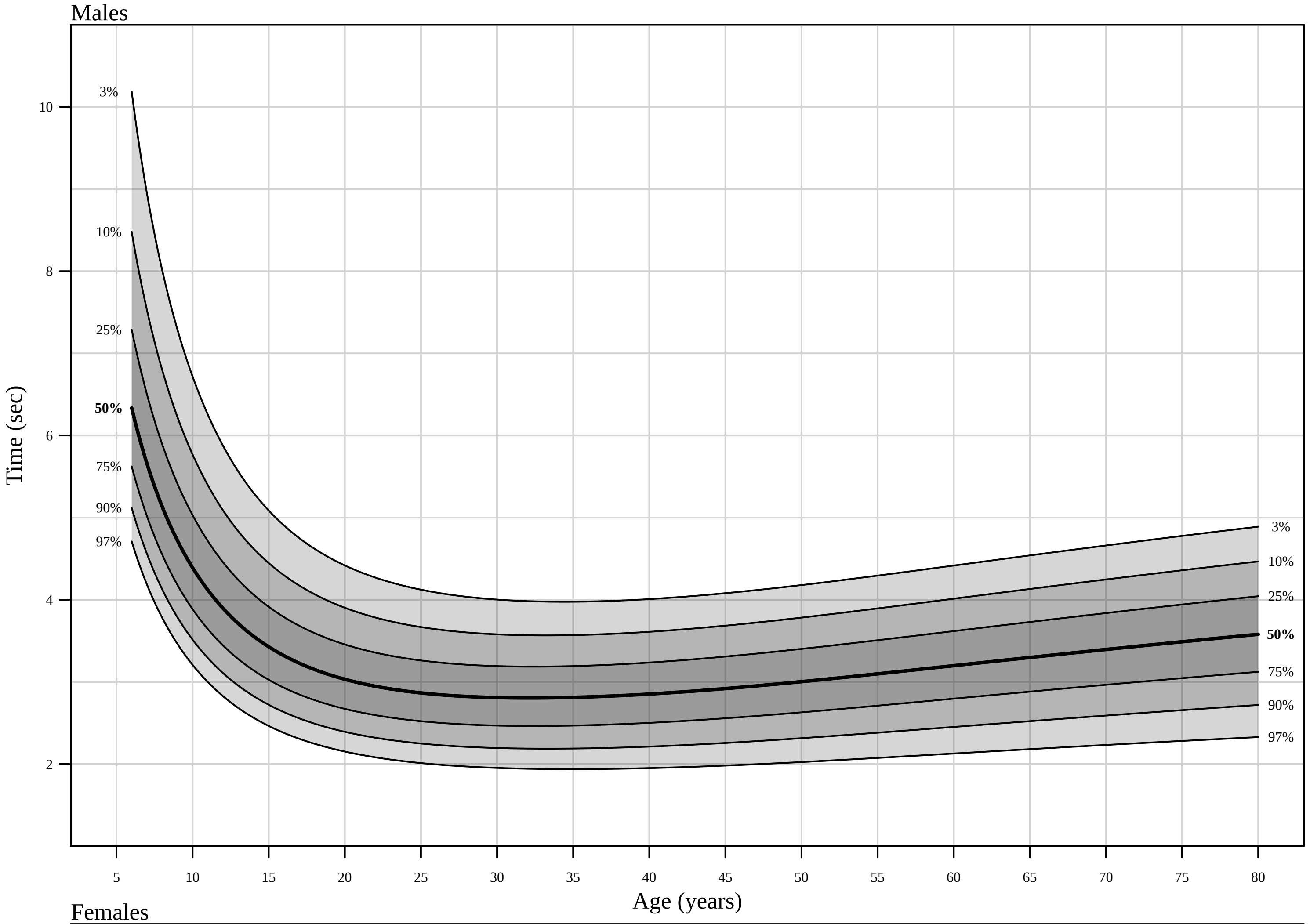

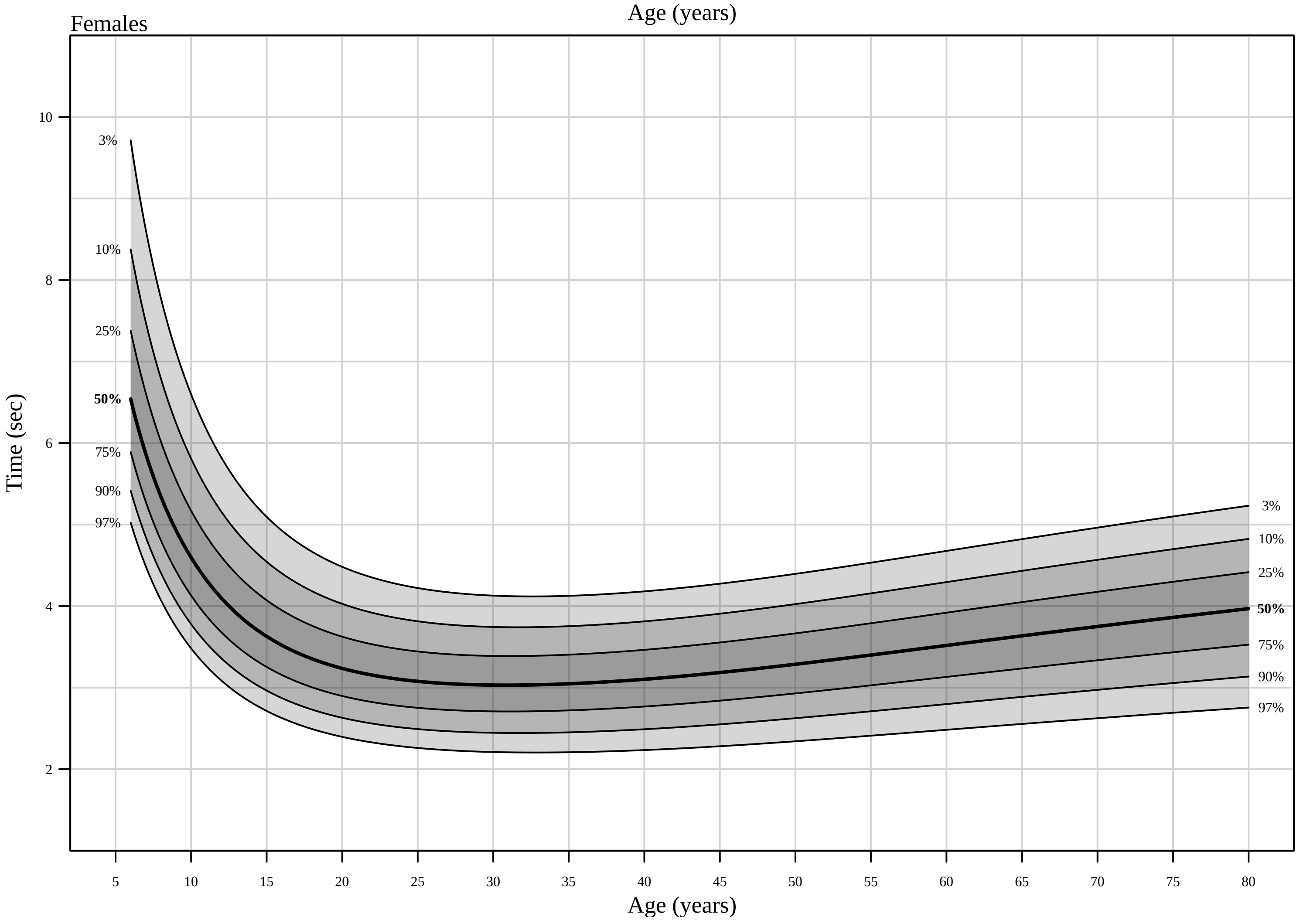

Repetitive hand movements (nondominant side)

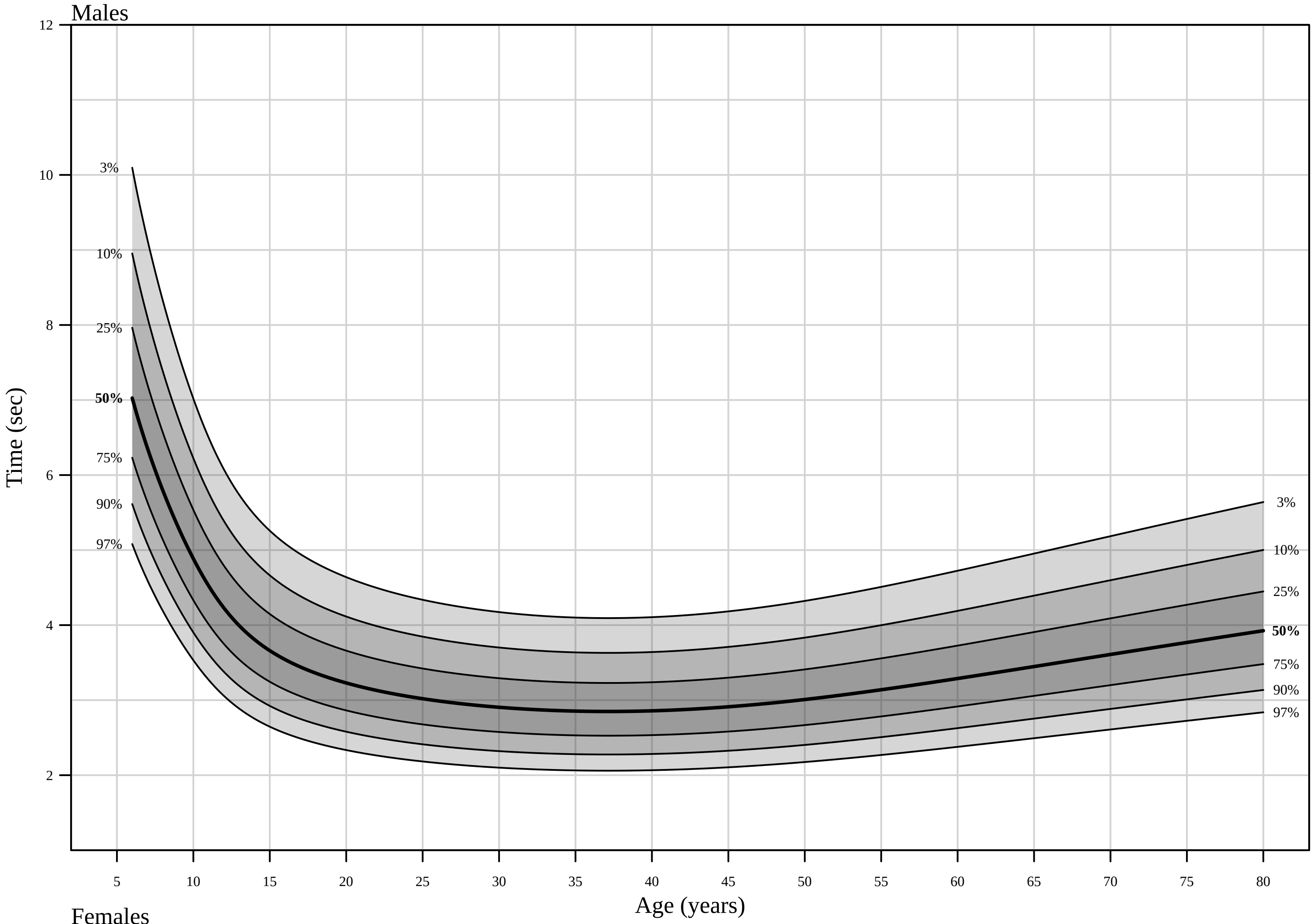

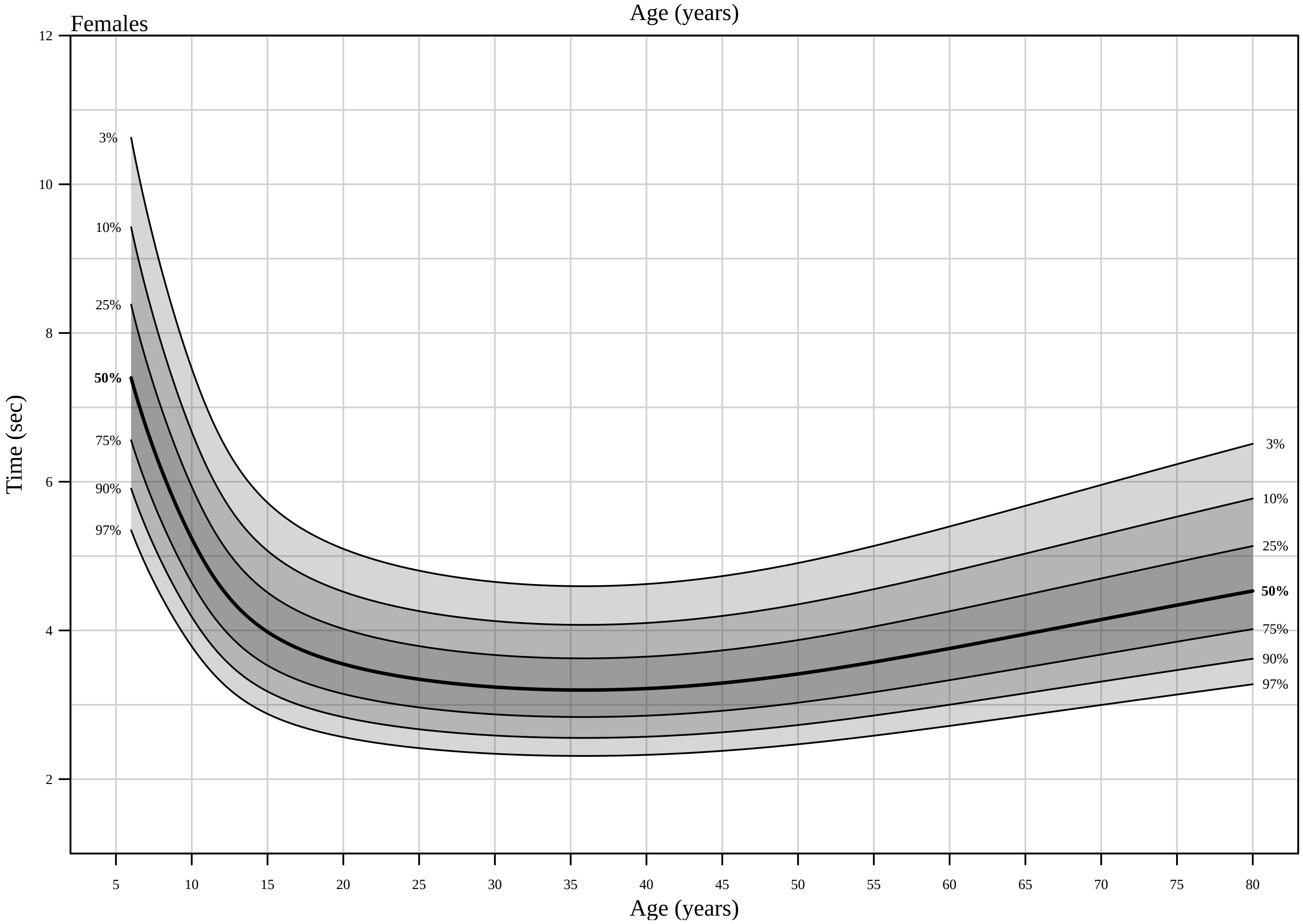

Alternating hand movements (dominant side)

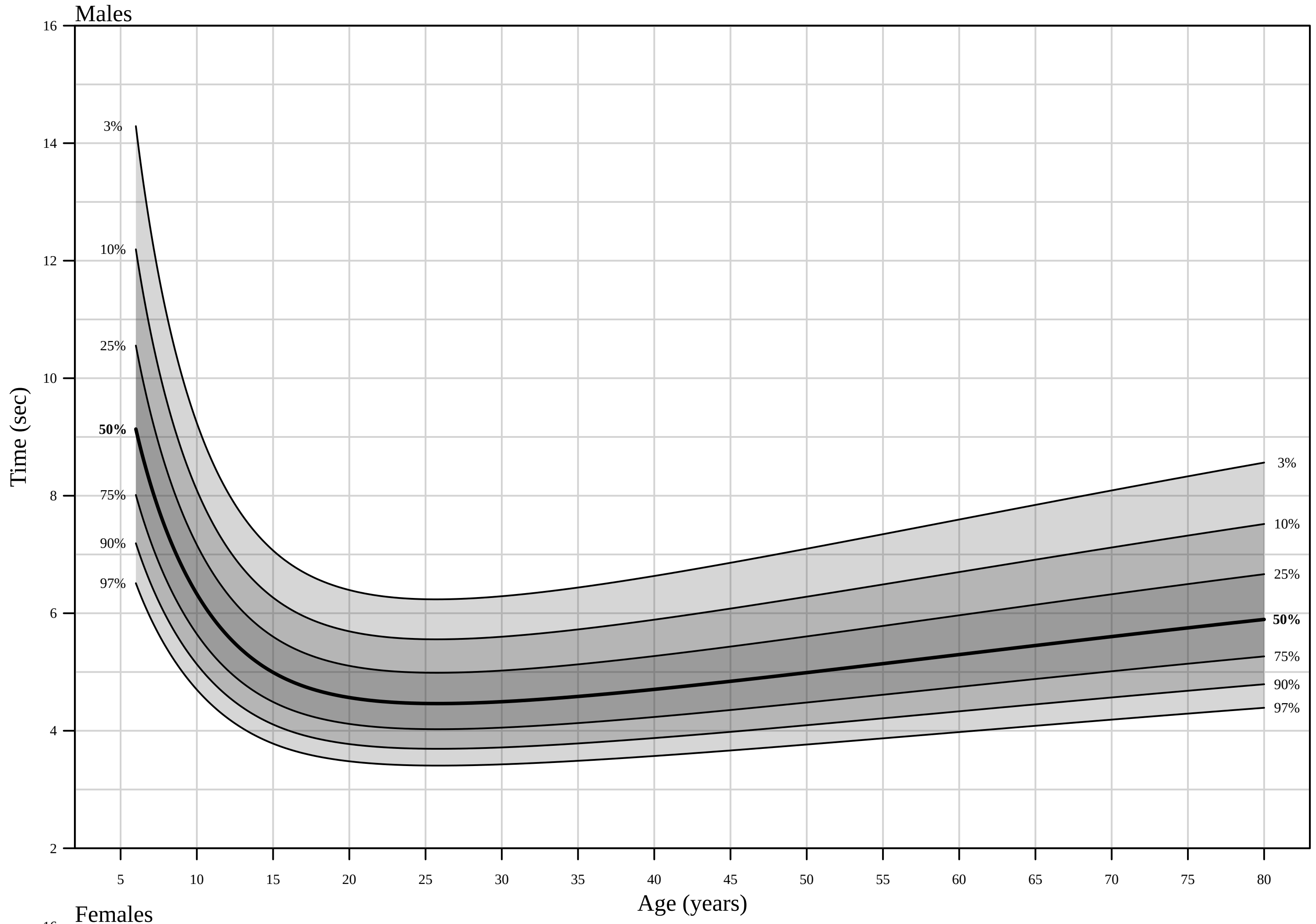

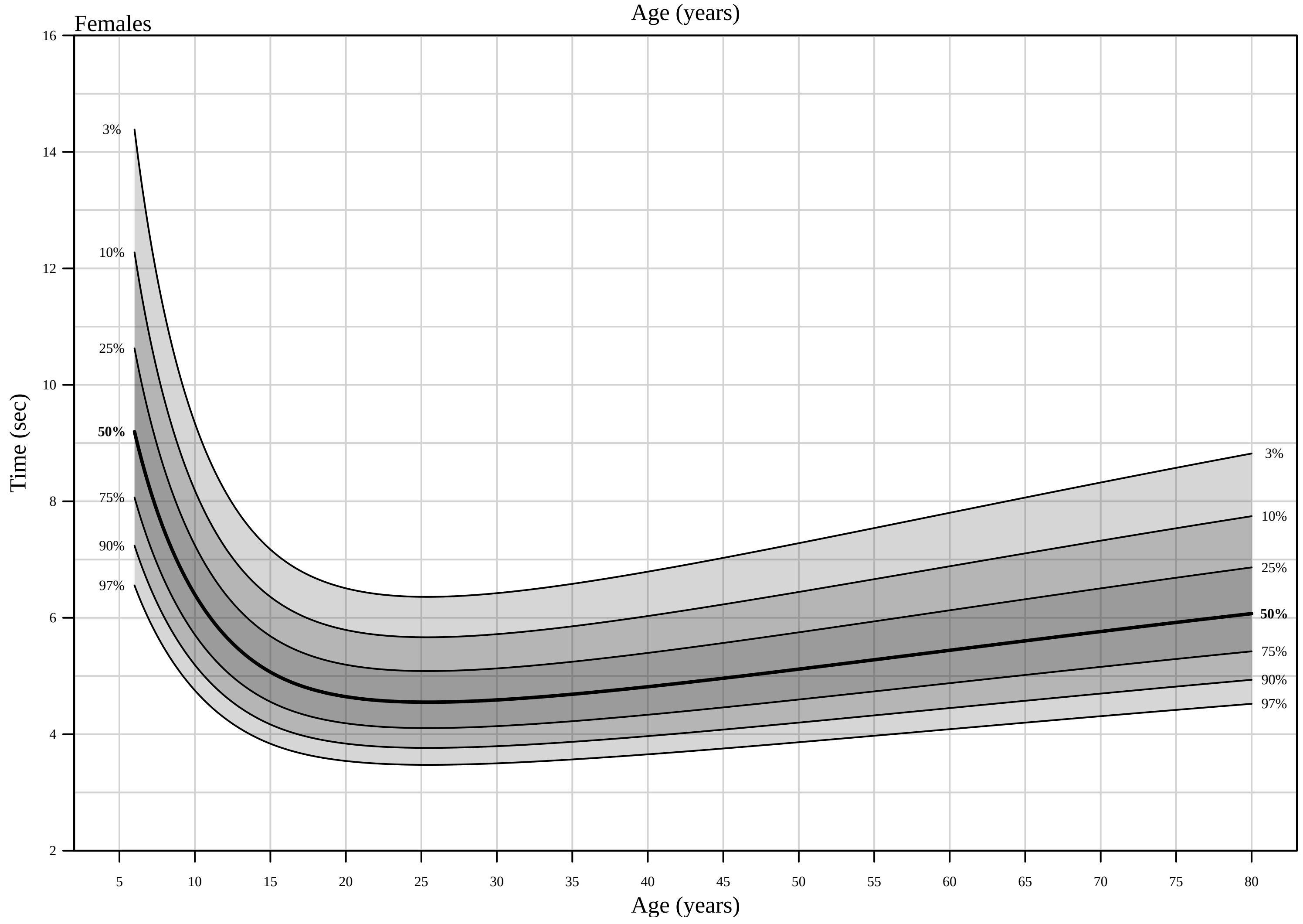

Alternating hand movements (nondominant side)

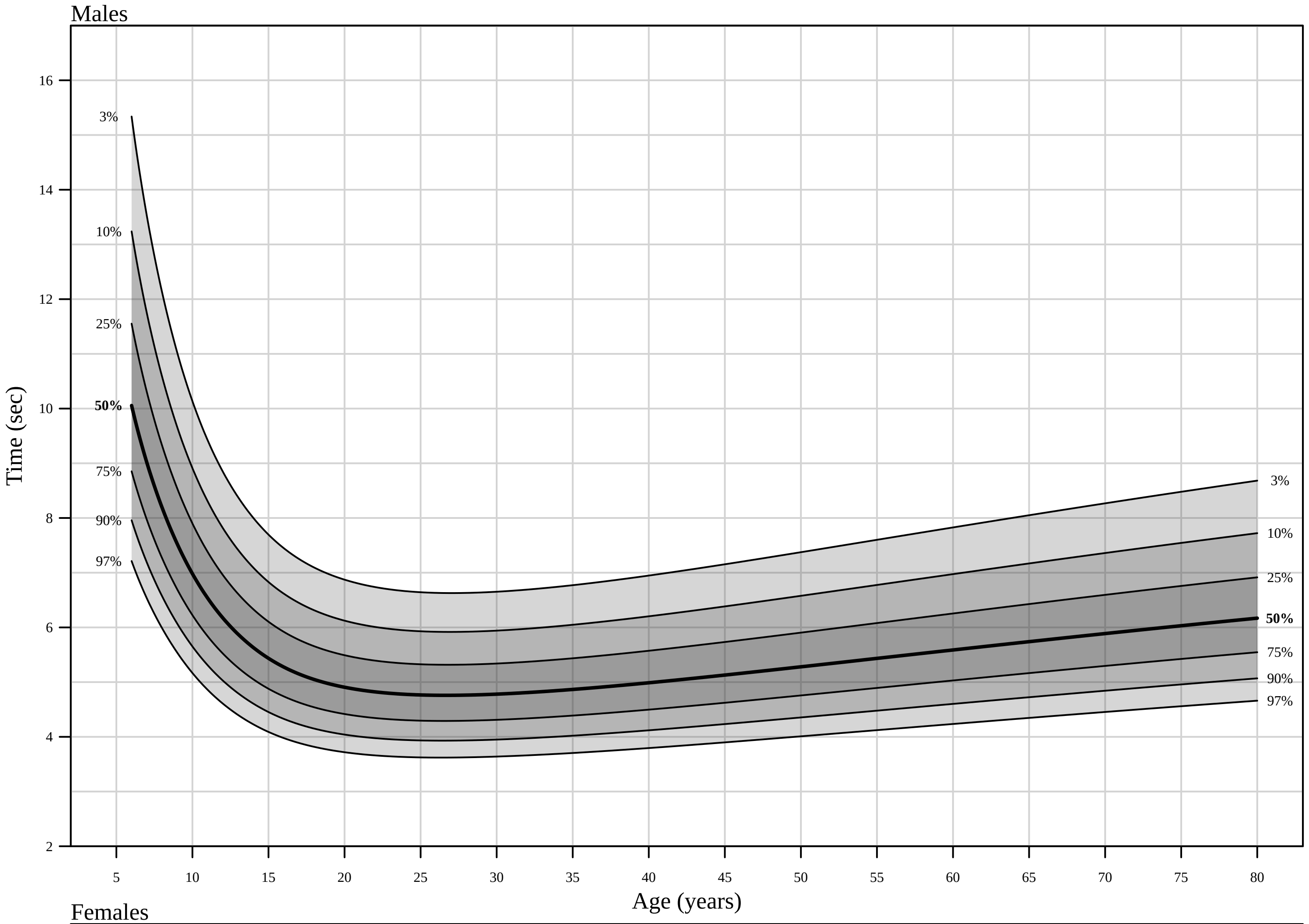

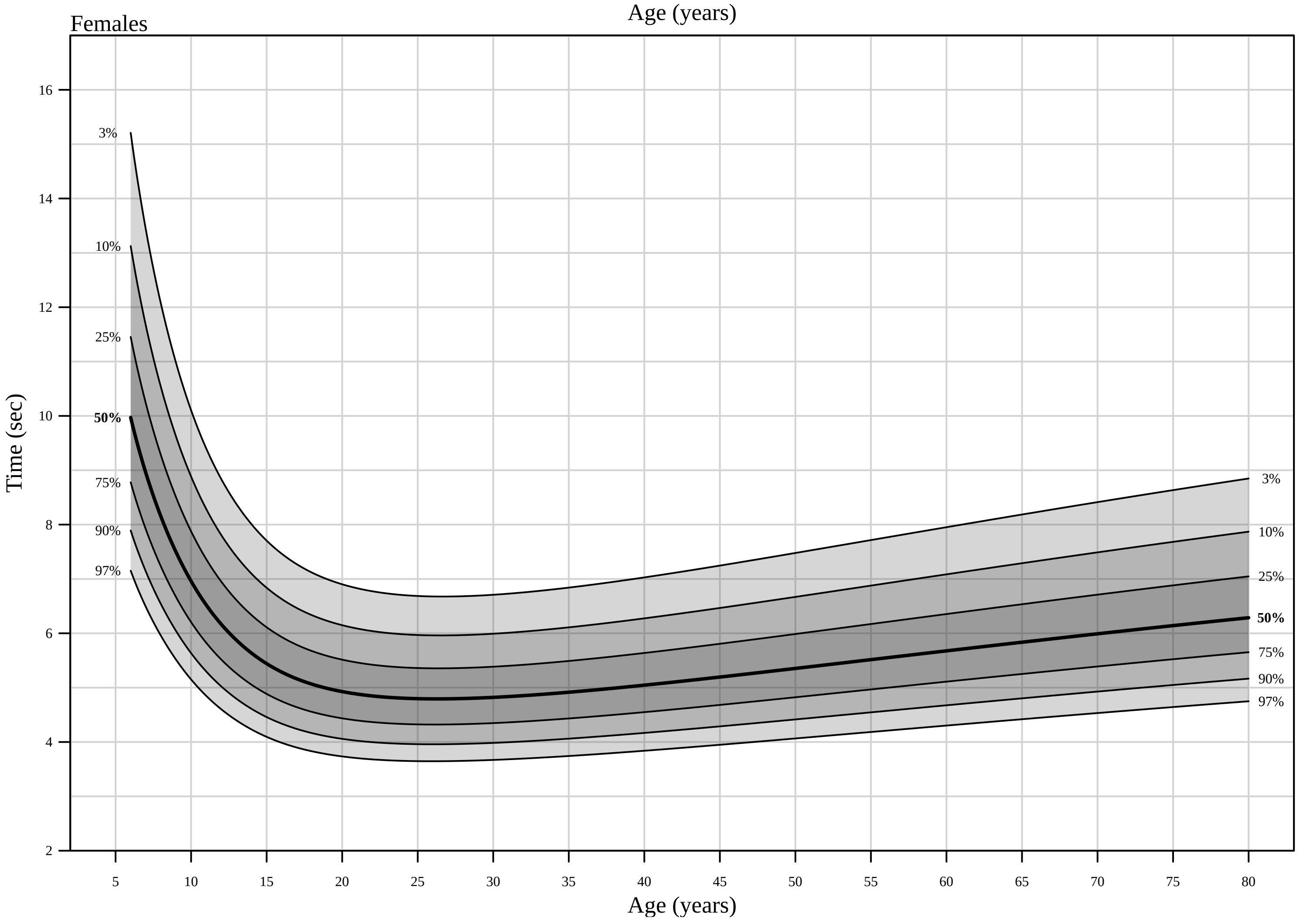

Repetitive finger movements (dominant side)

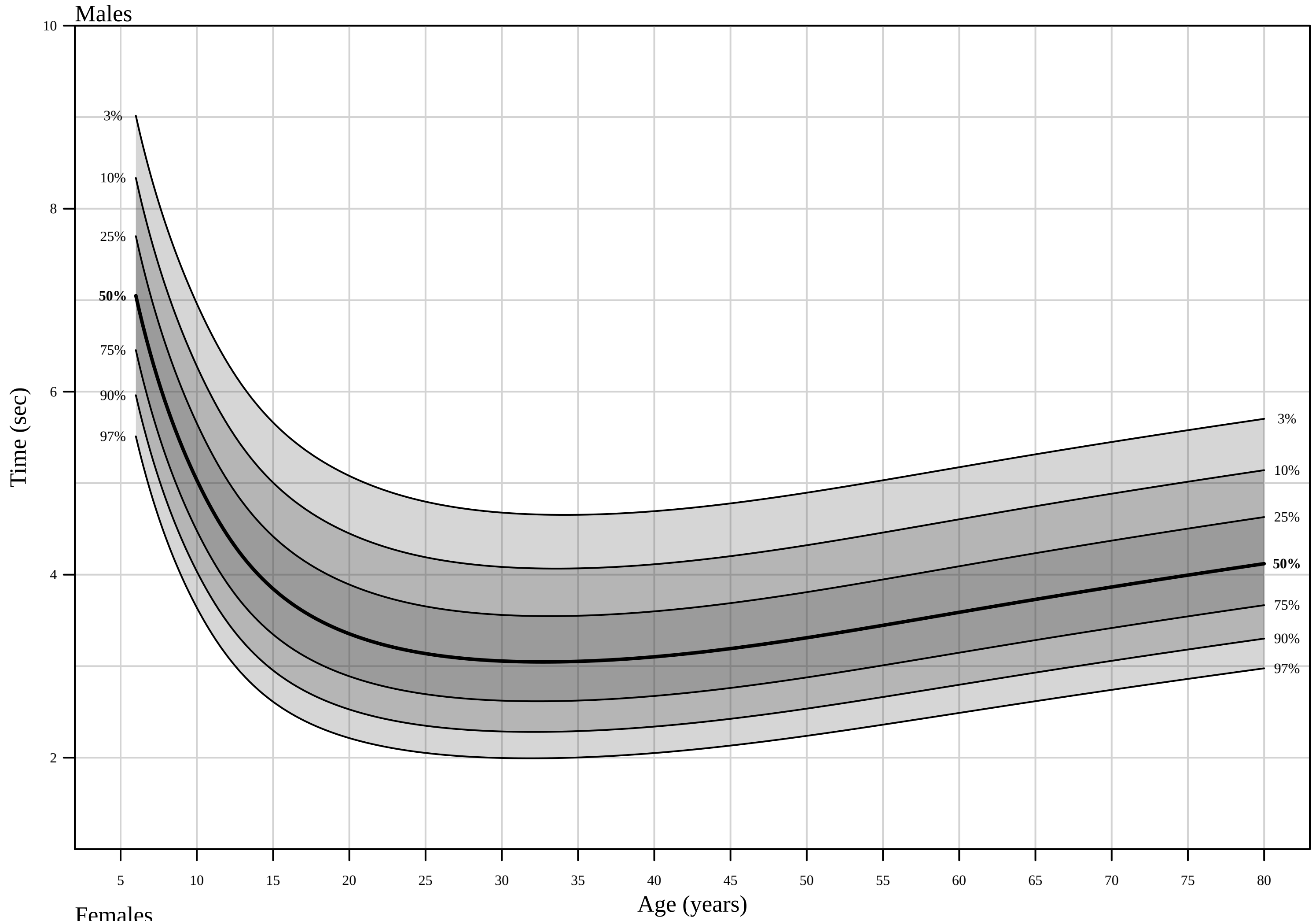

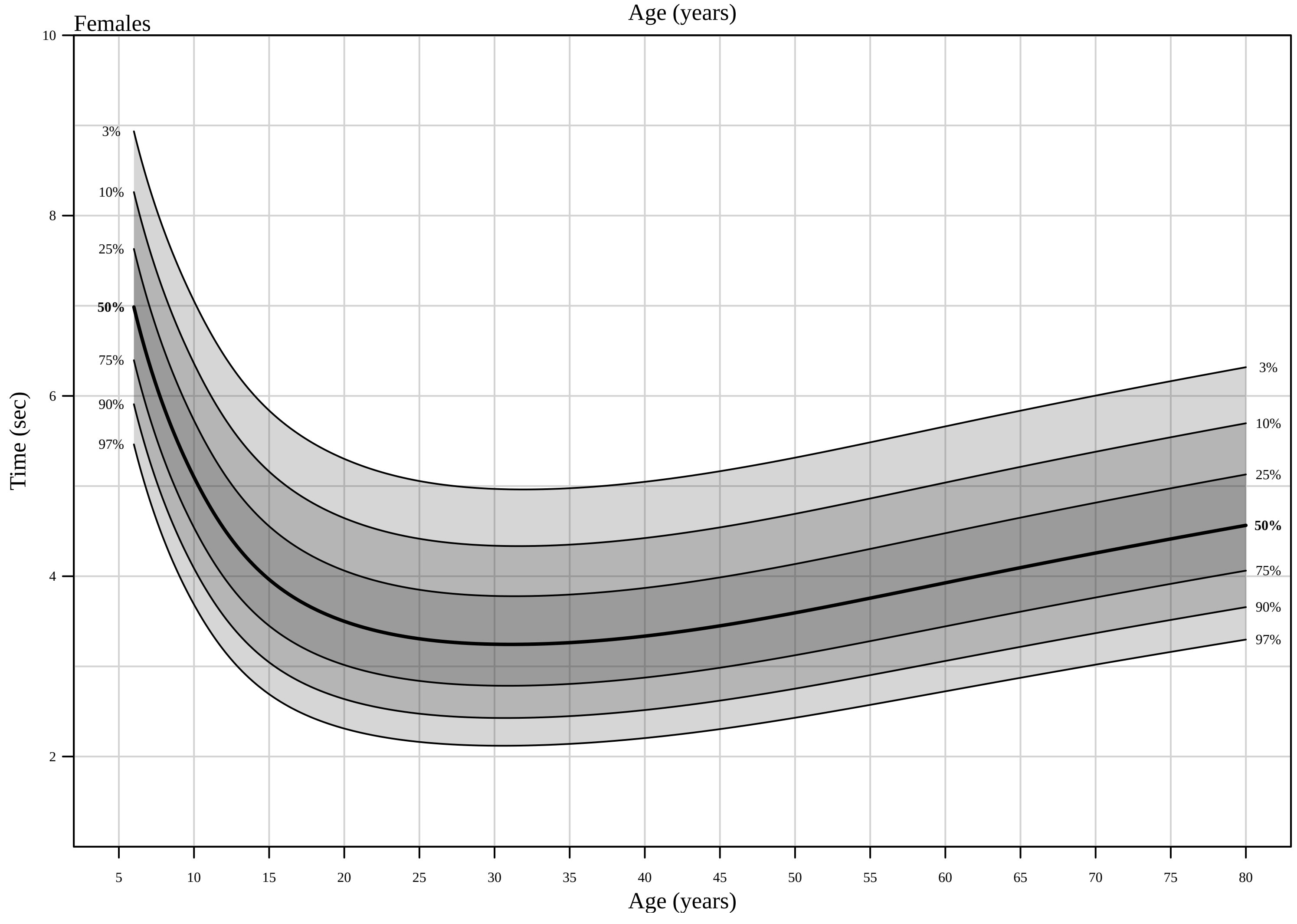

Repetitive finger movements (nondominant side)

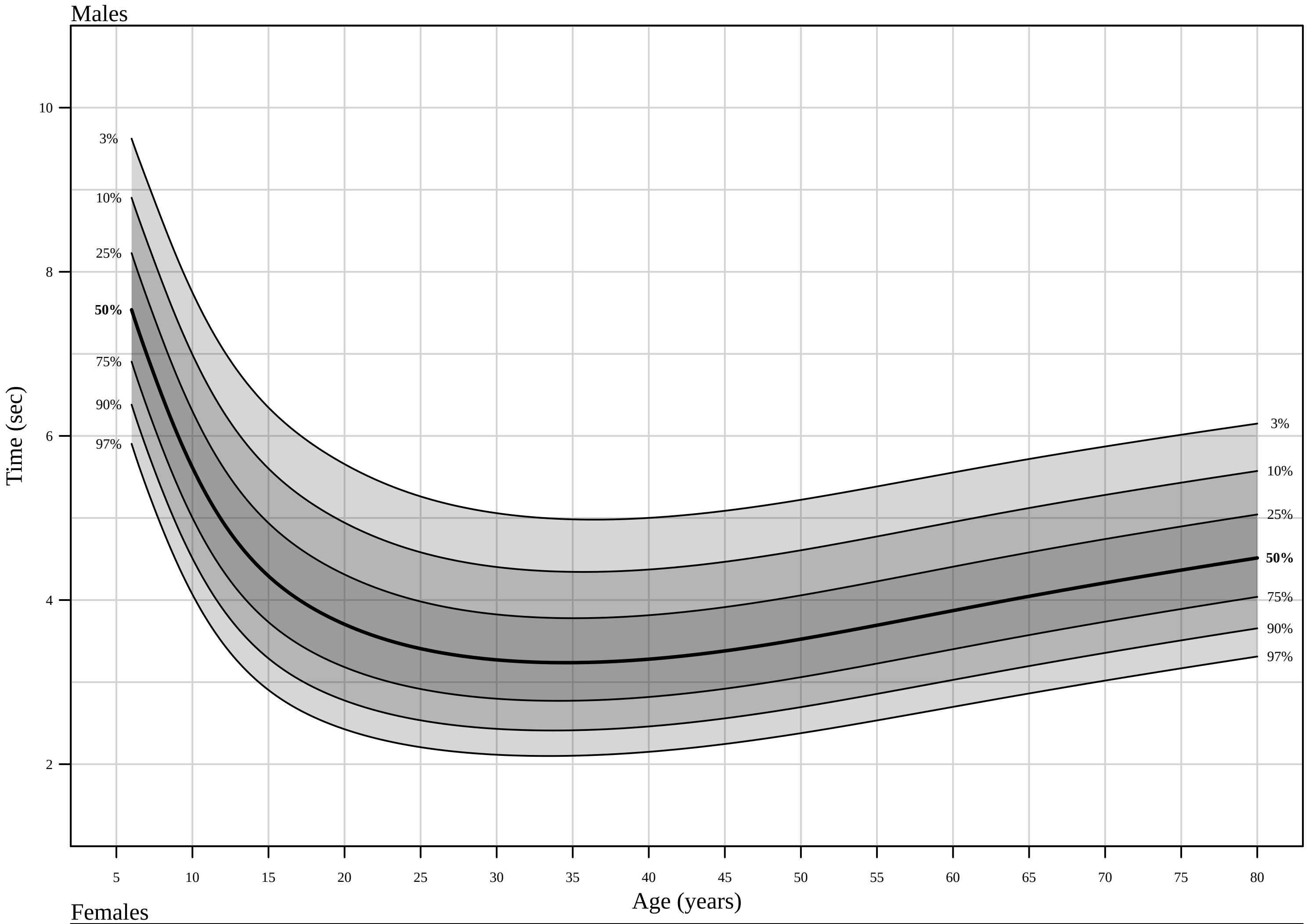

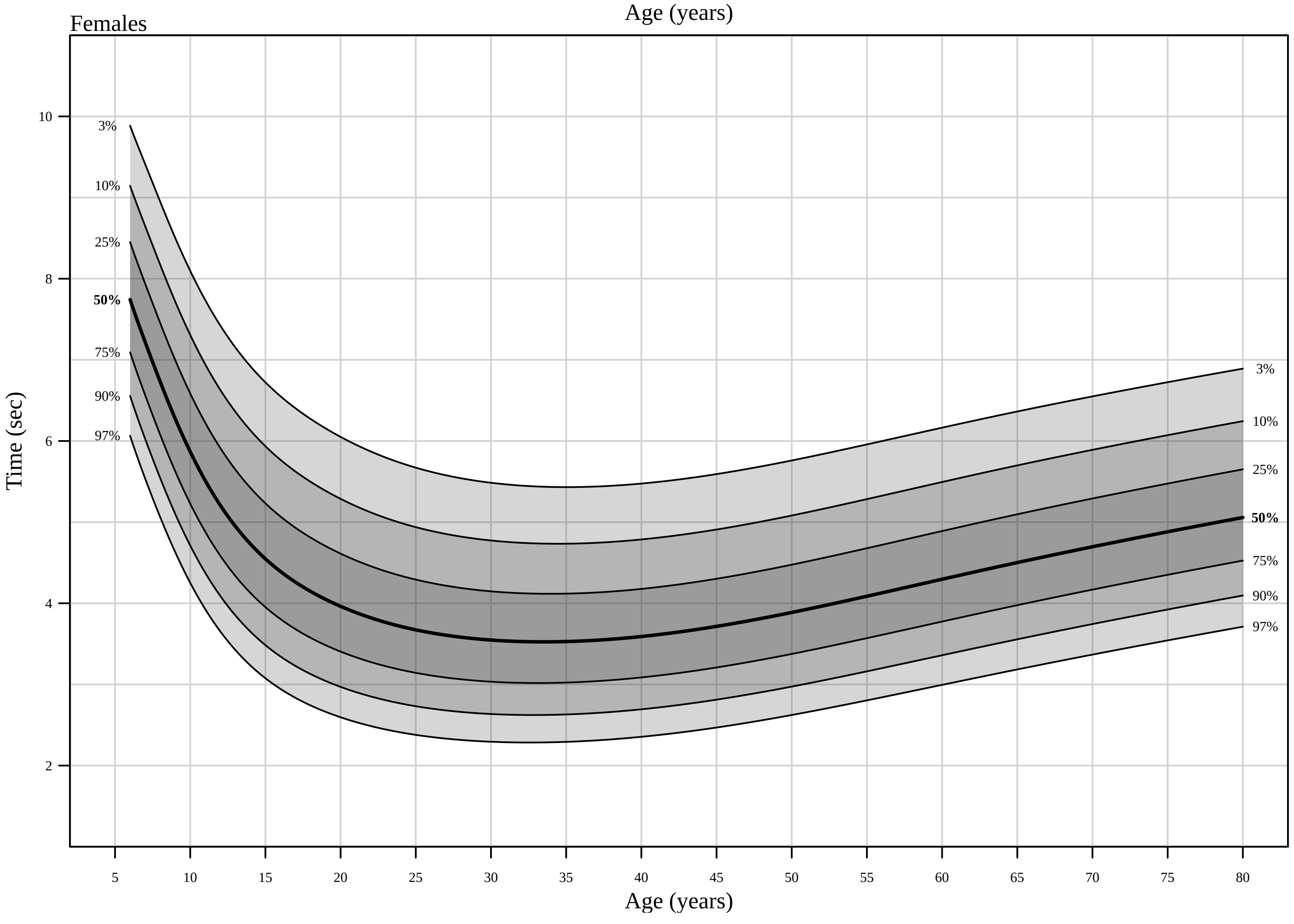

Sequential finger movements (dominant side)

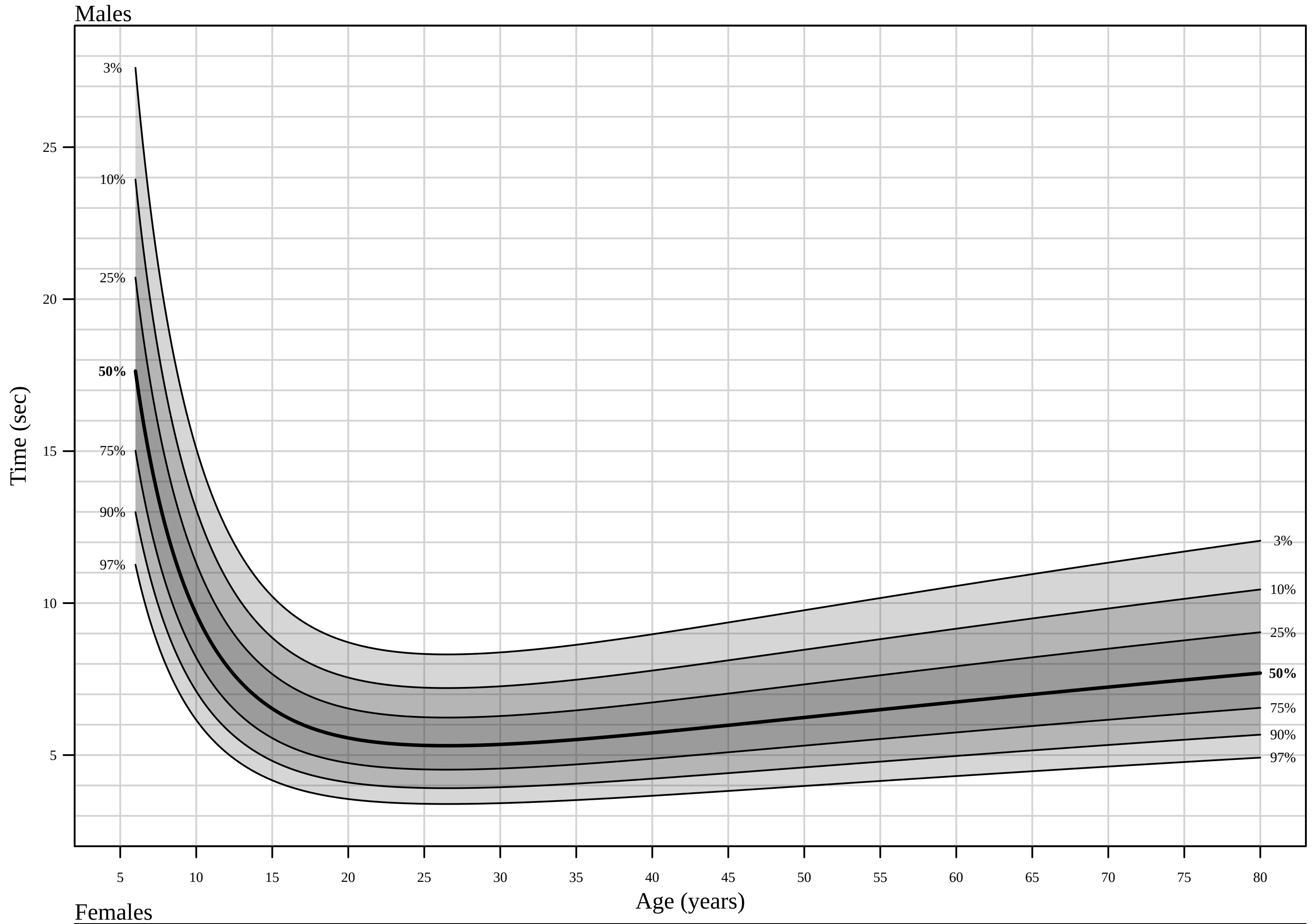

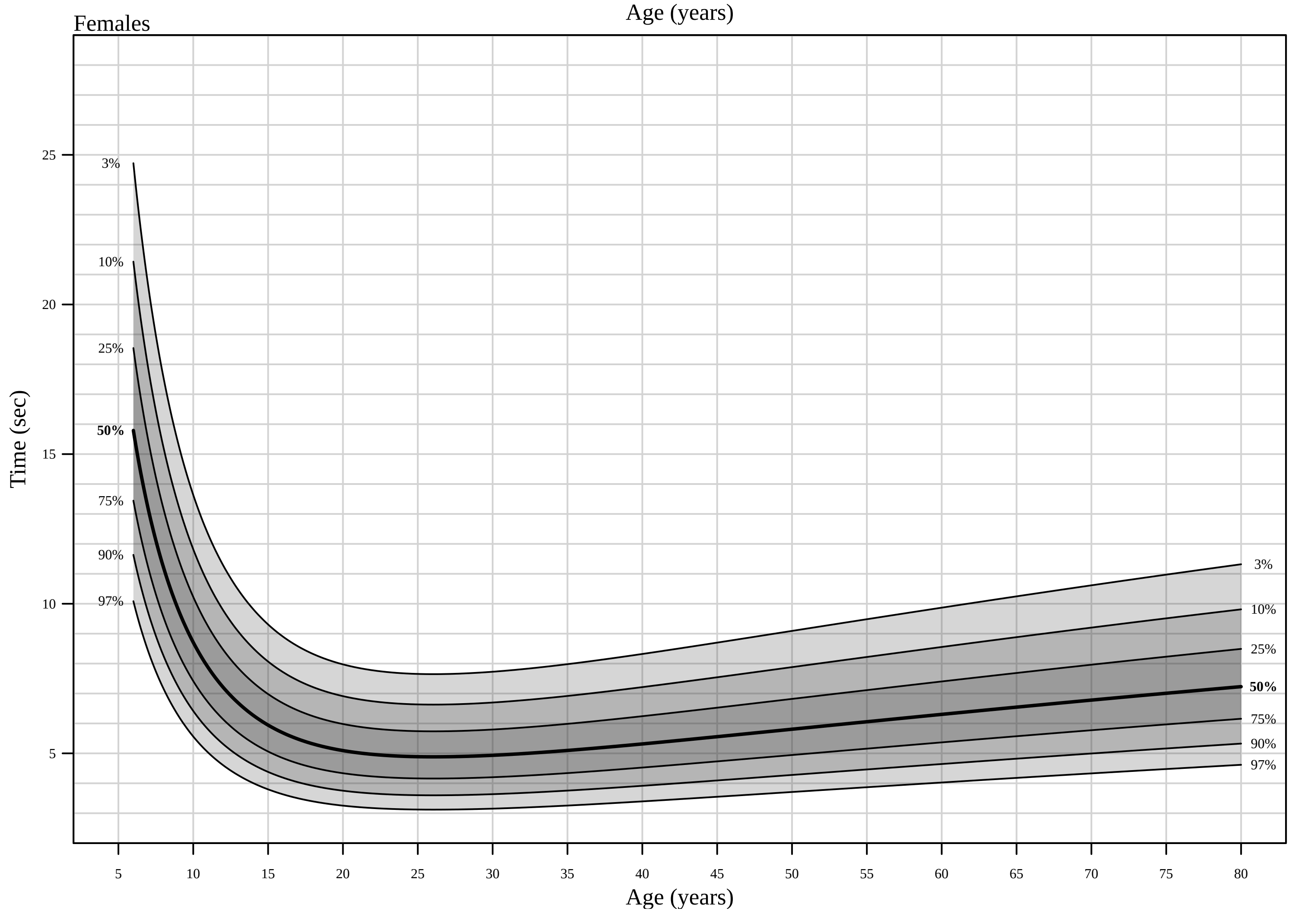

Sequential finger movements (nondominant side)

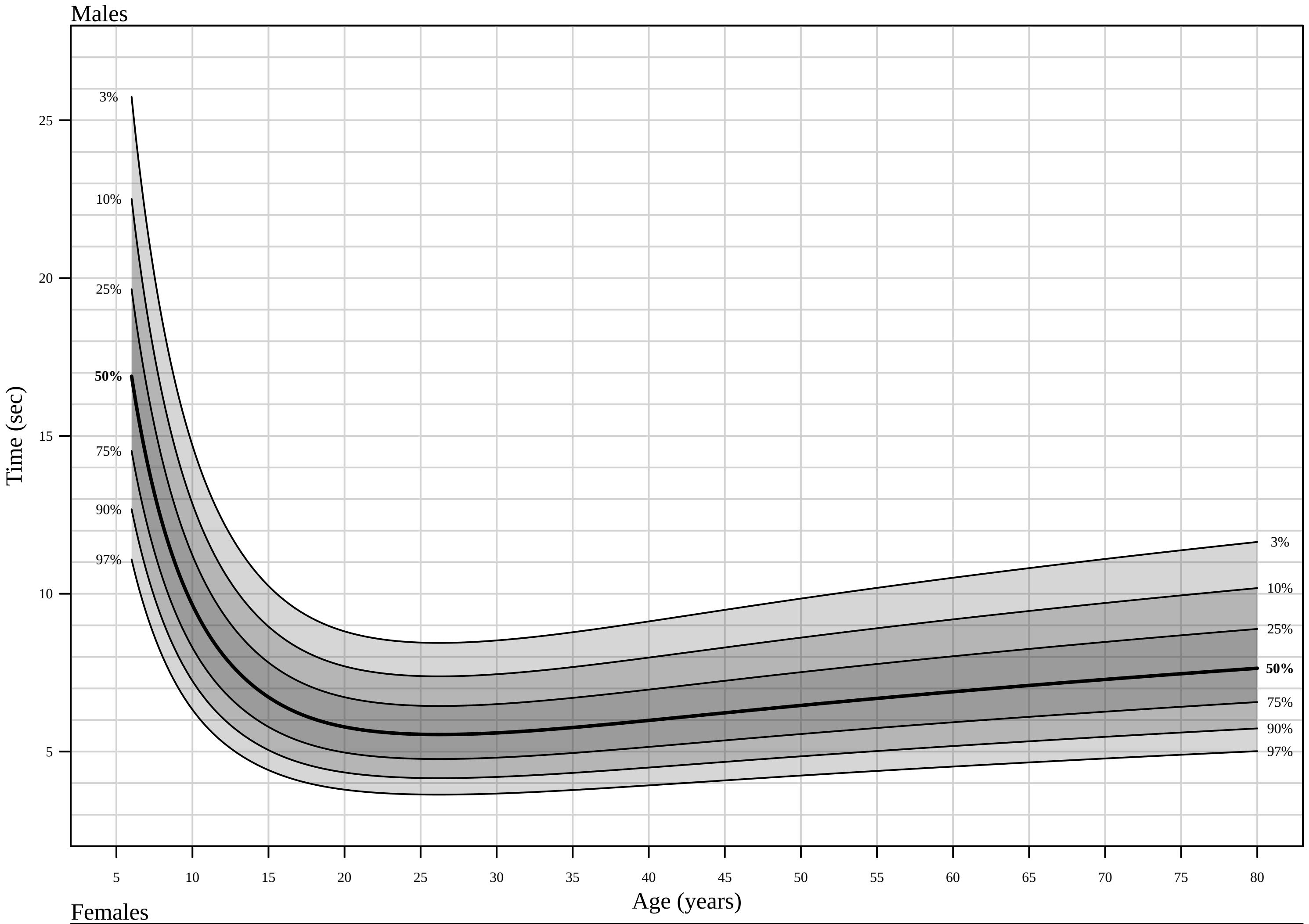

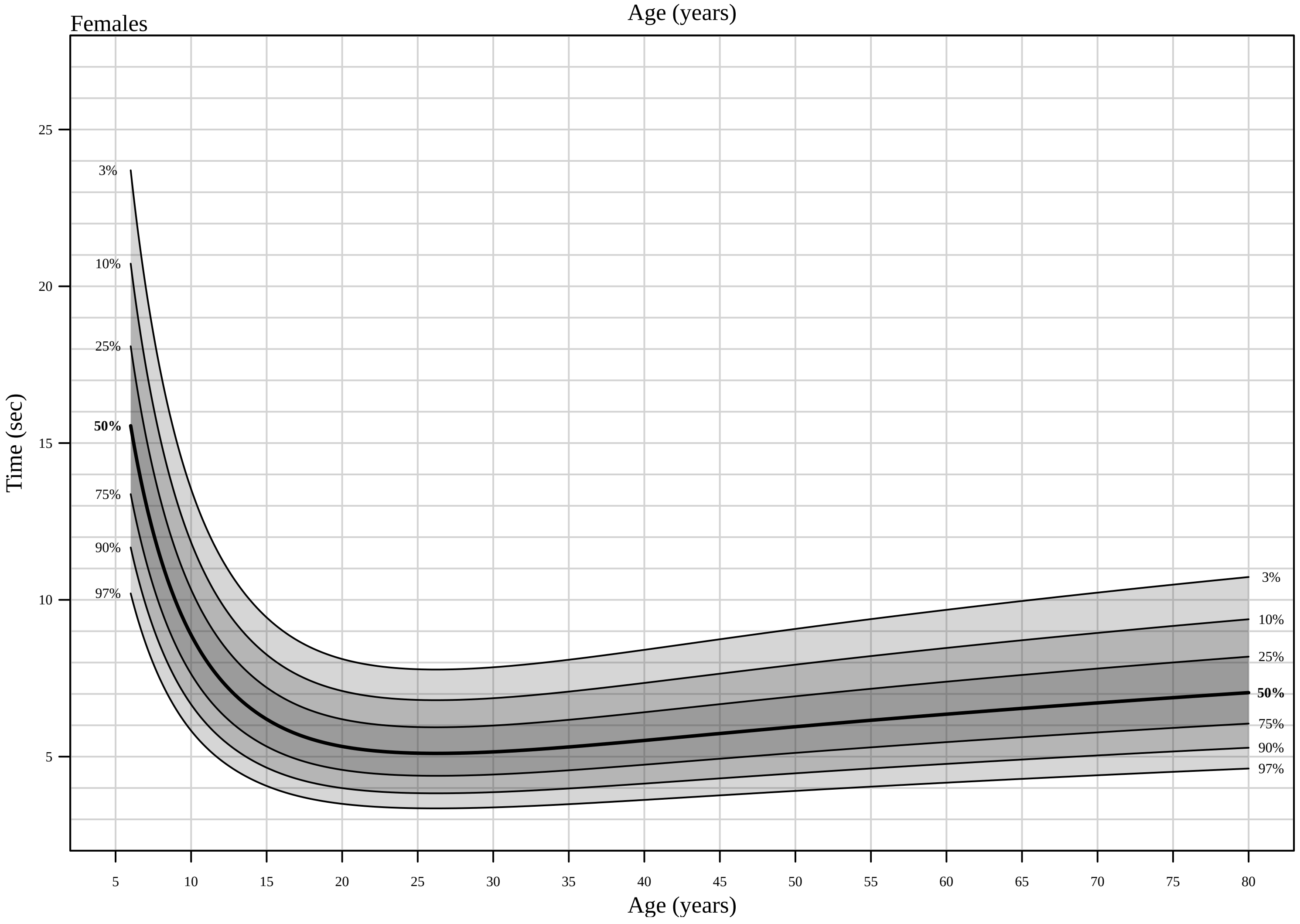

Standing on one leg with eyes open (dominant side)

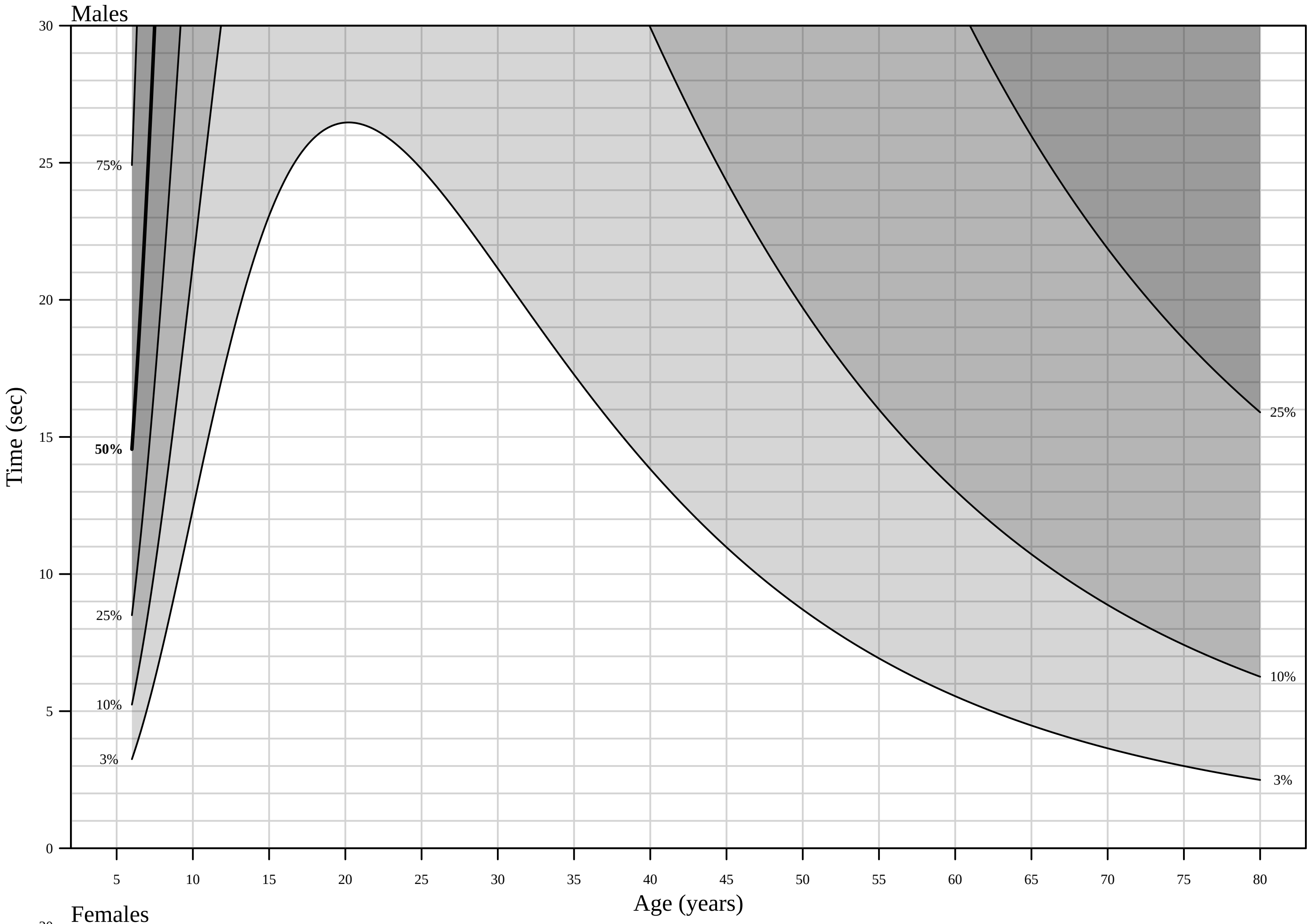

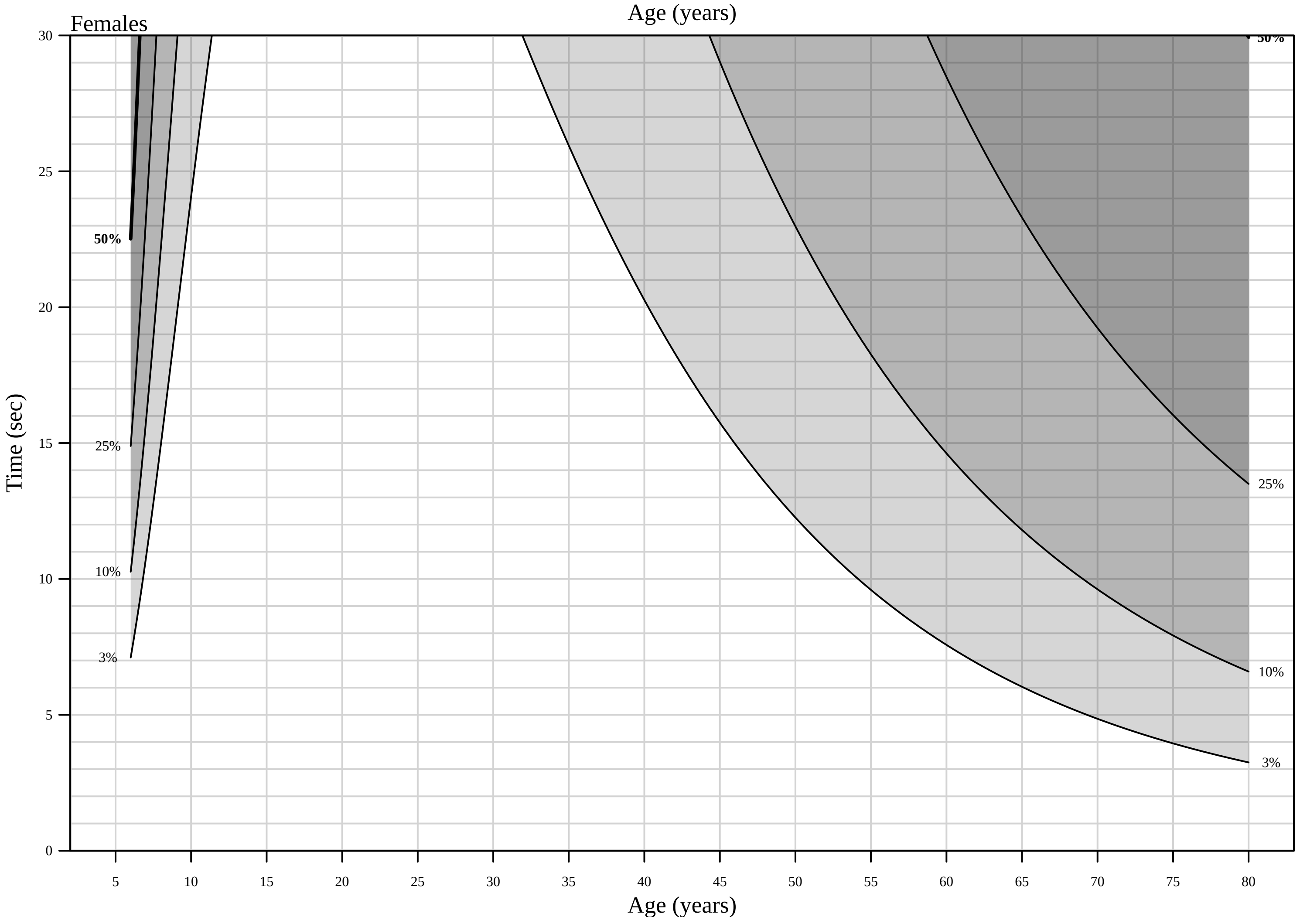

Standing on one leg with eyes open (nondominant side)

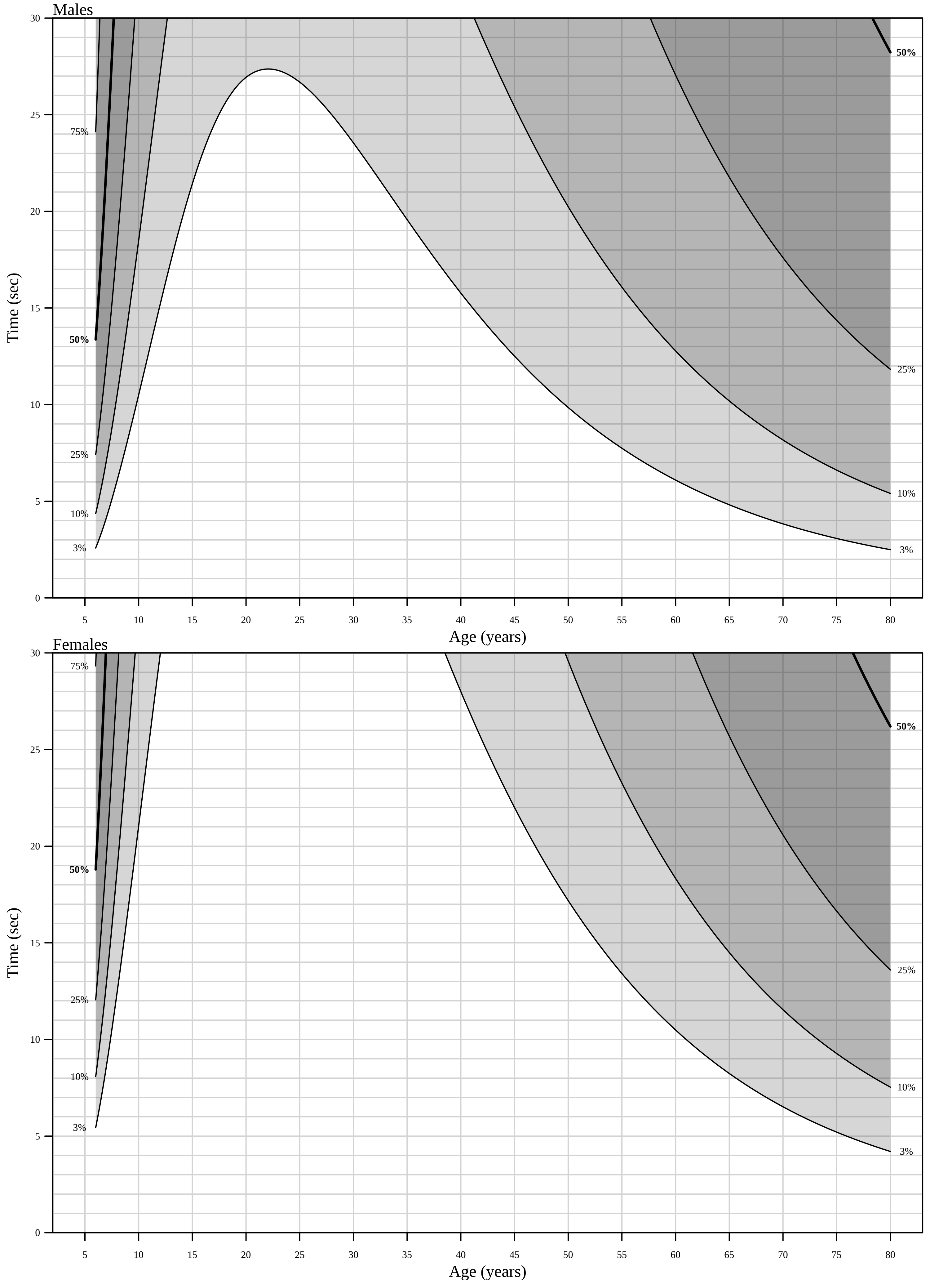

Standing on one leg with eyes closed (dominant side)

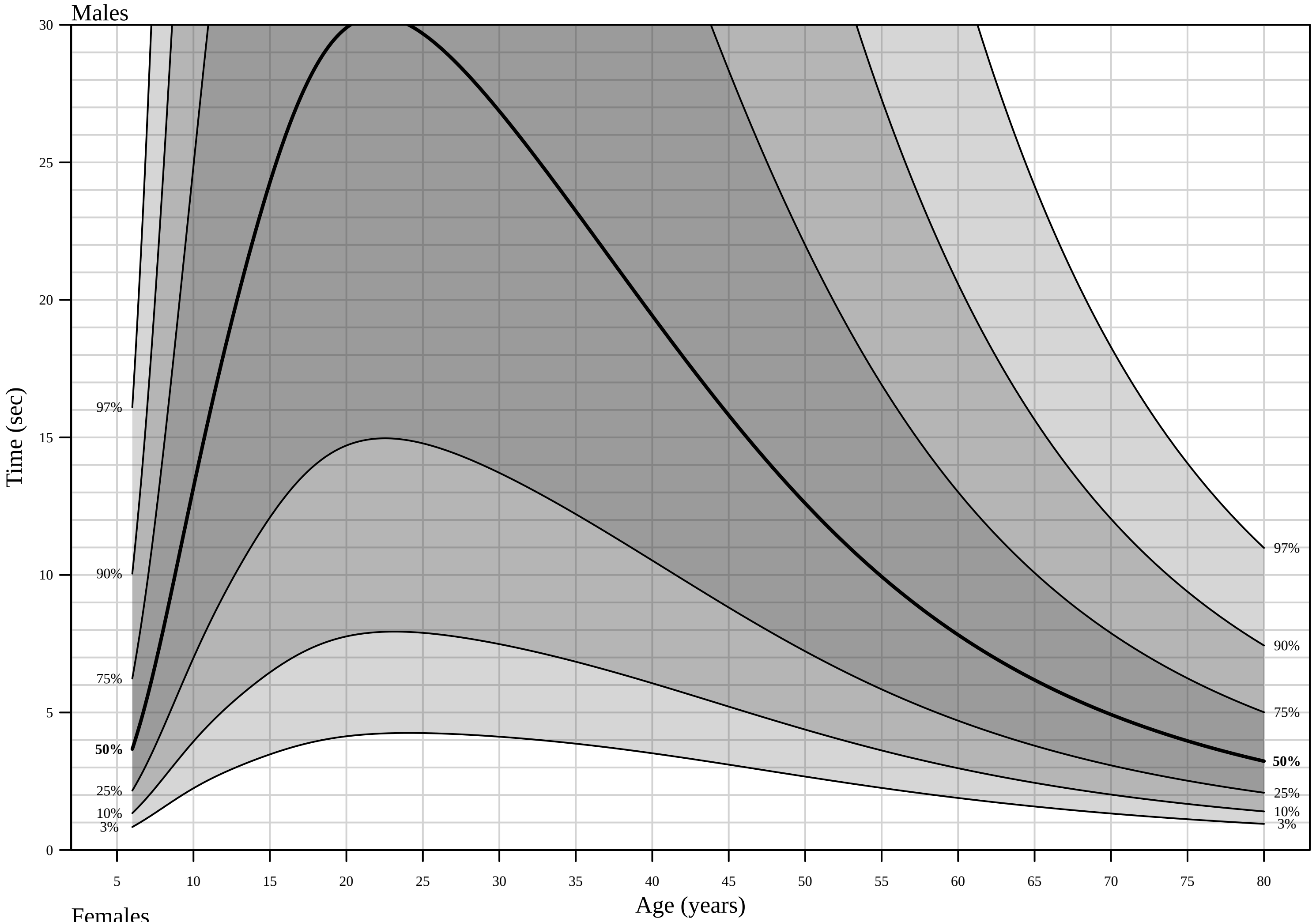

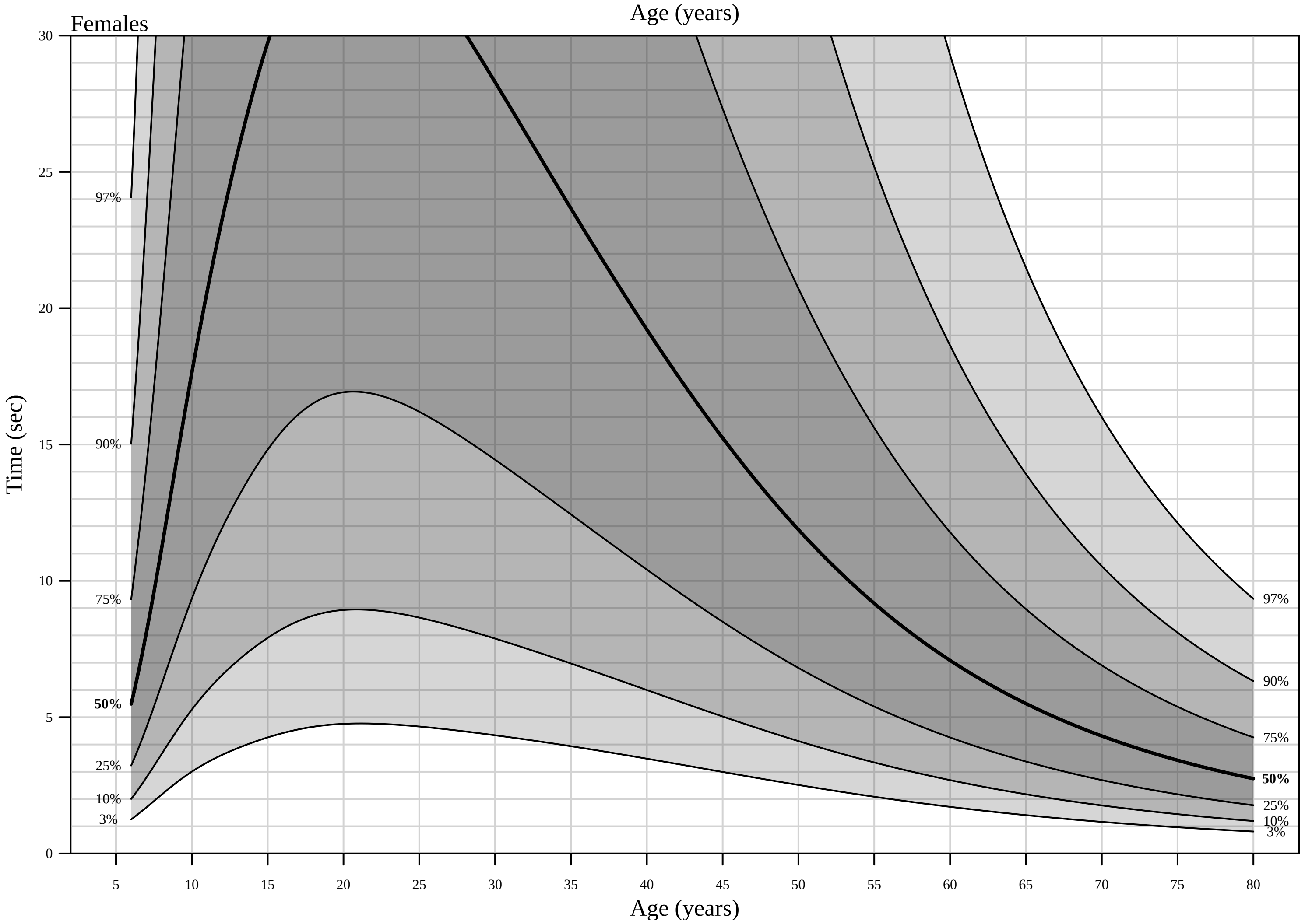

Standing on one leg with eyes closed (nondominant side)

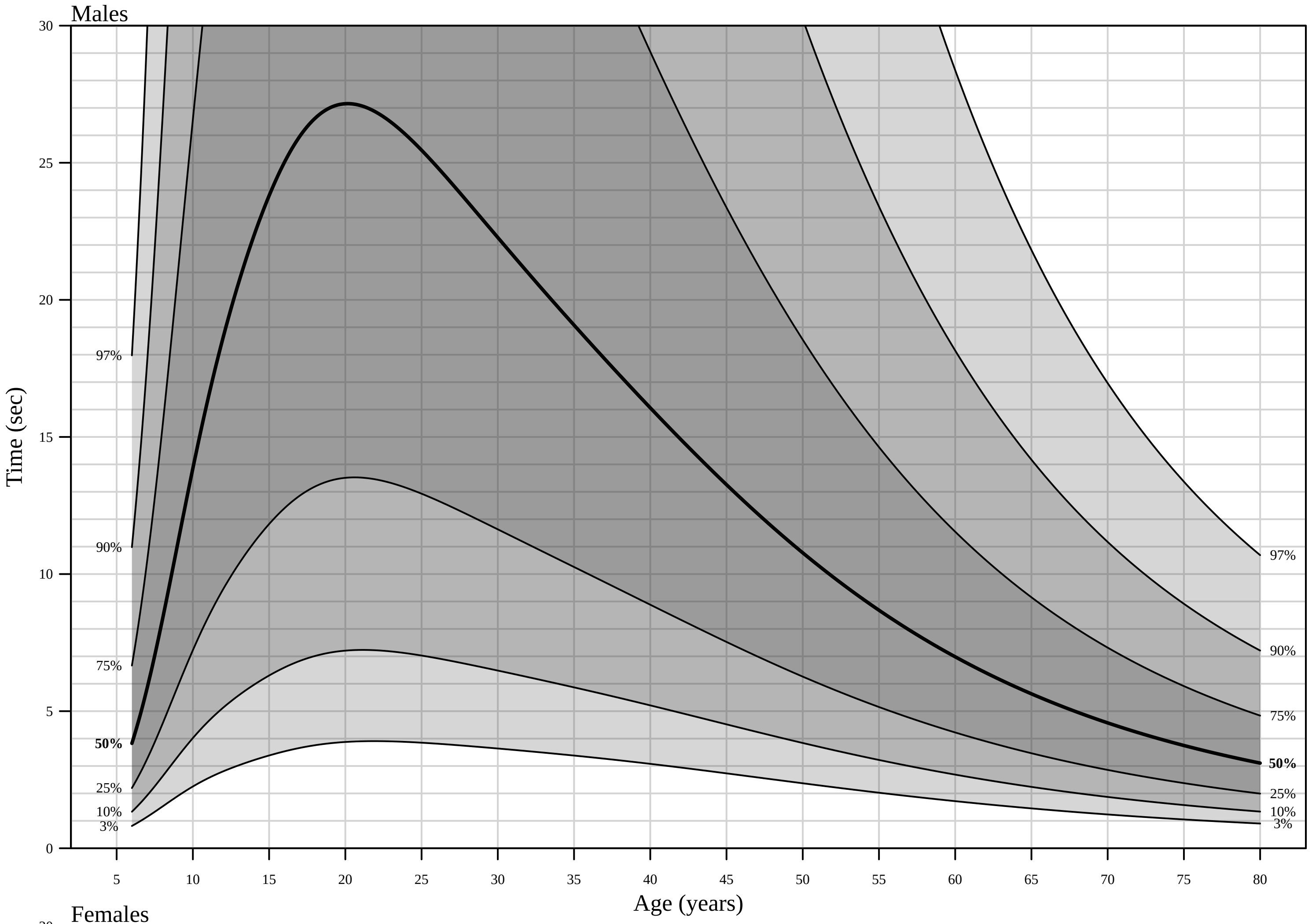

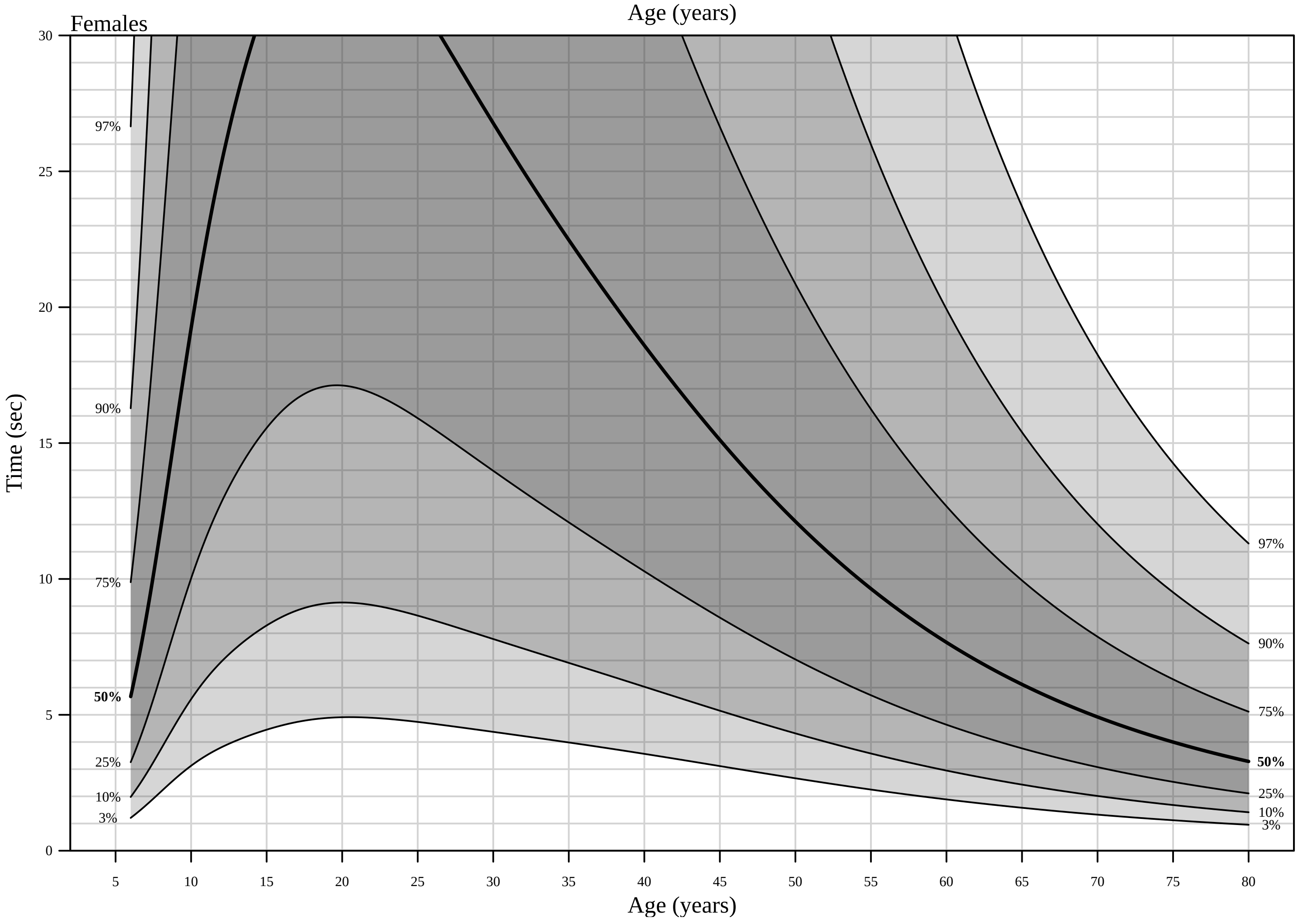

Jumping sideways

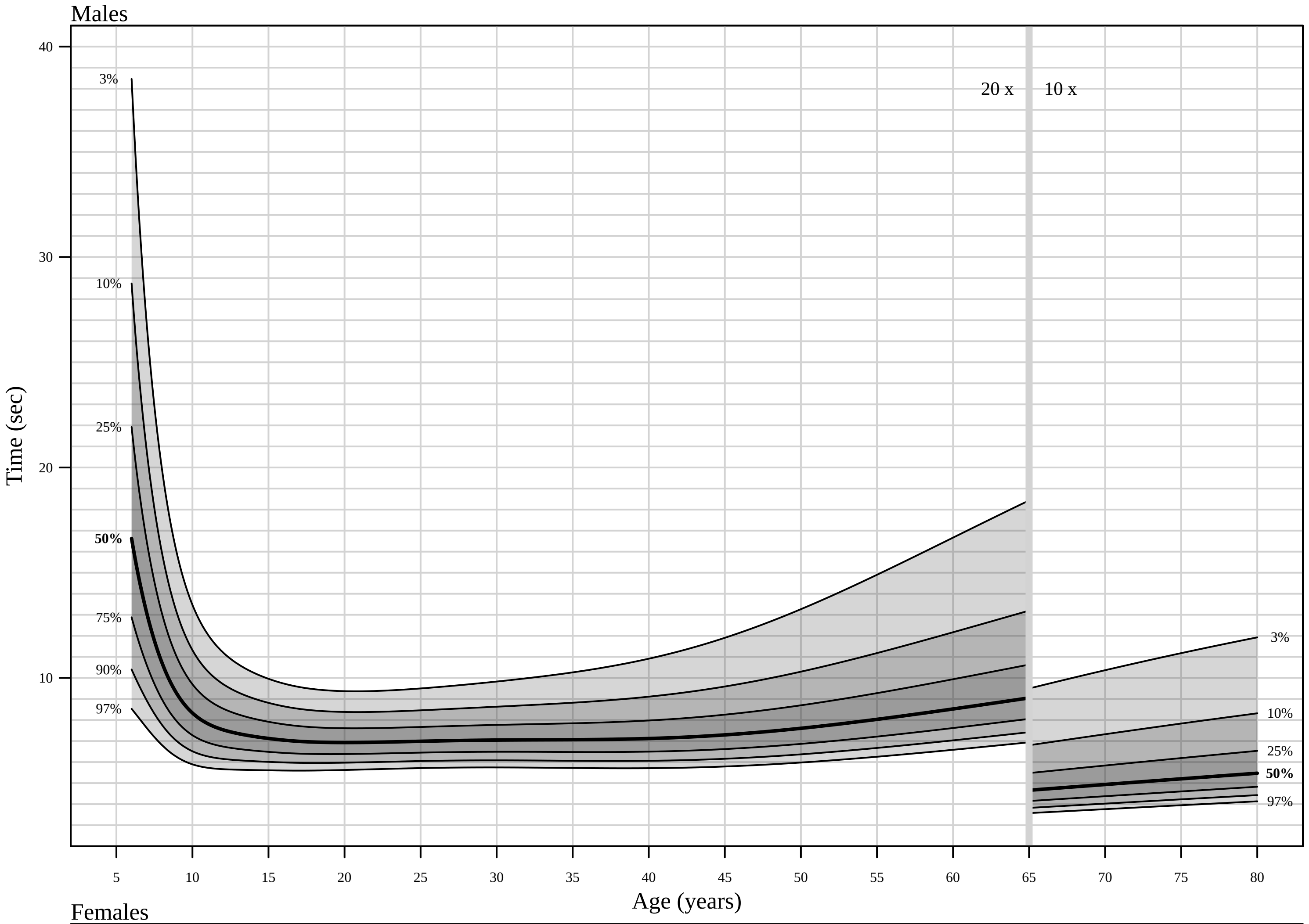

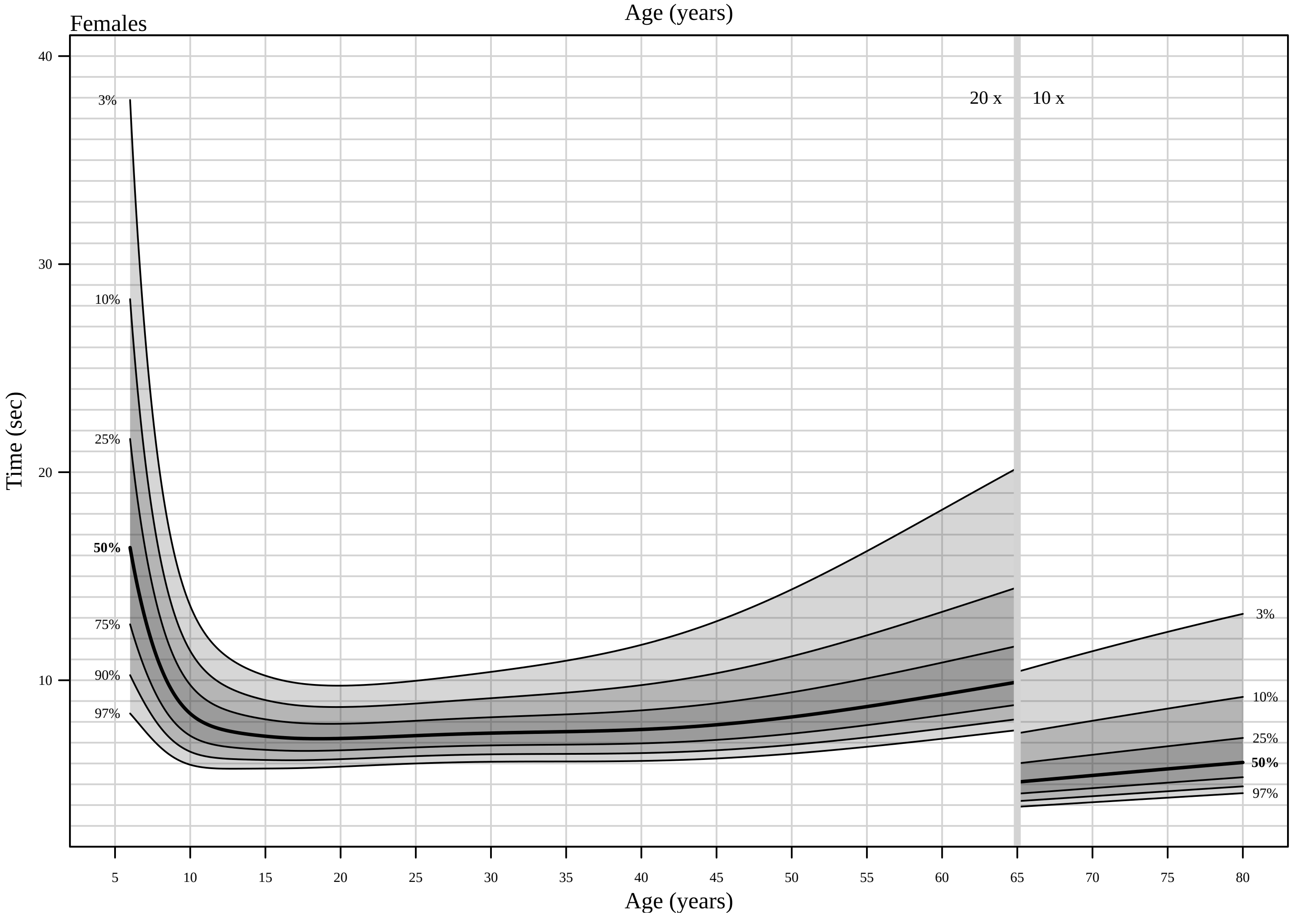

Chair rise

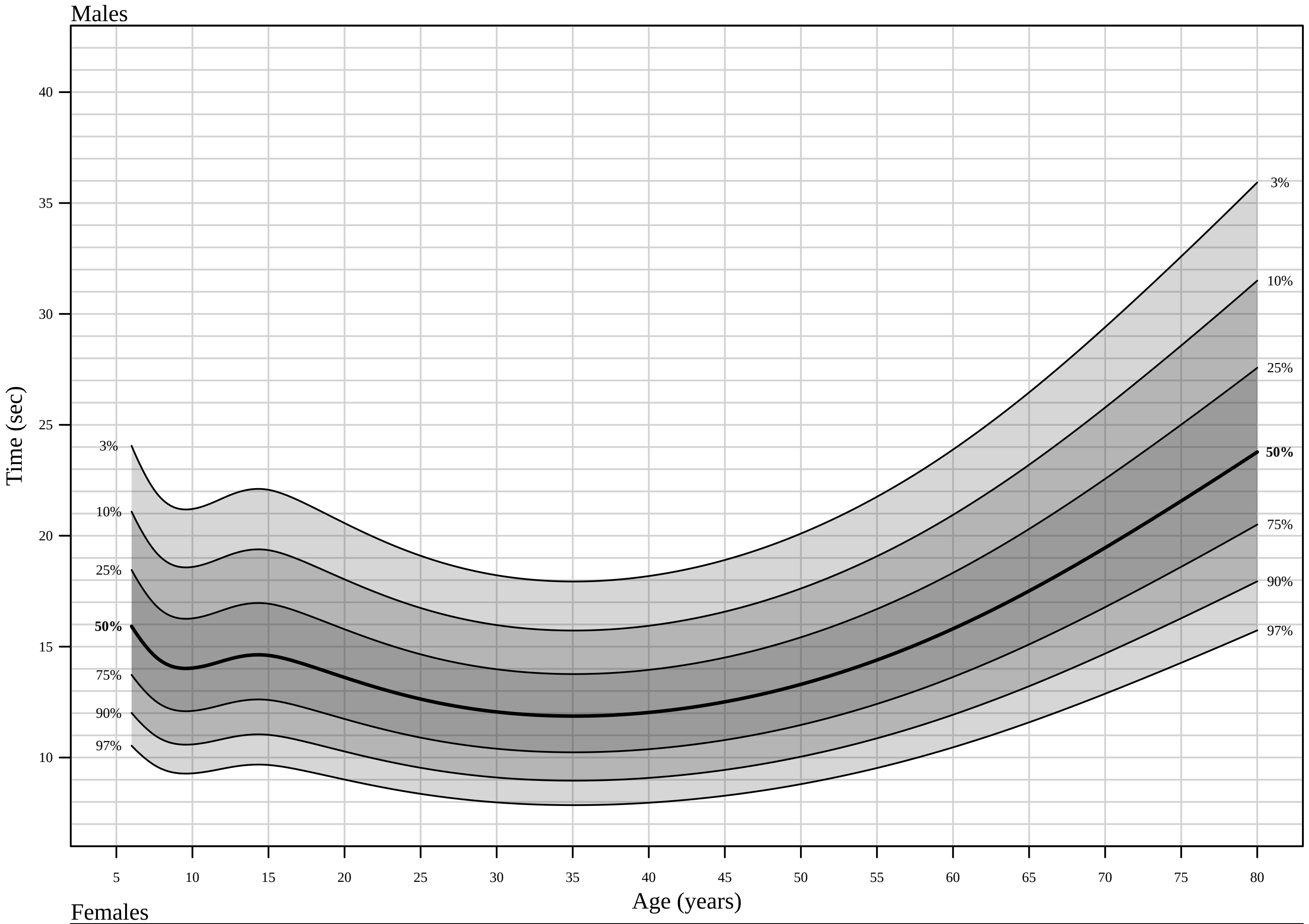
Males
Time (sec)
Age (years)
3%
10%
25%
50%
75%
90%
97%
5
10
15
20
25
30
35
40
45
50
55
60
65
70
75
80

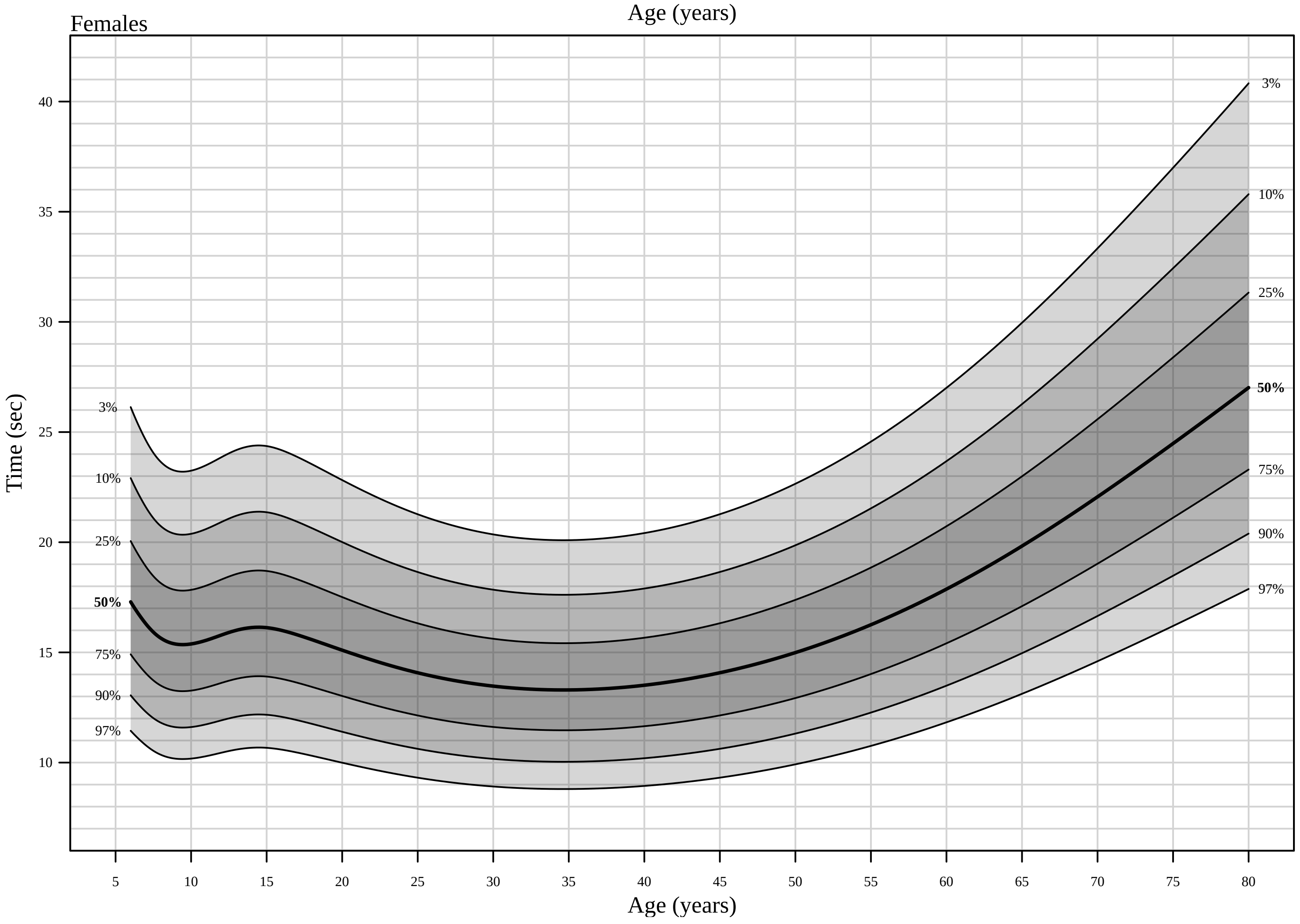
Females
Time (sec)
Age (years)
3%
10%
25%
50%
75%
90%
97%
5
10
15
20
25
30
35
40
45
50
55
60
65
70
75
80

Standing long jump

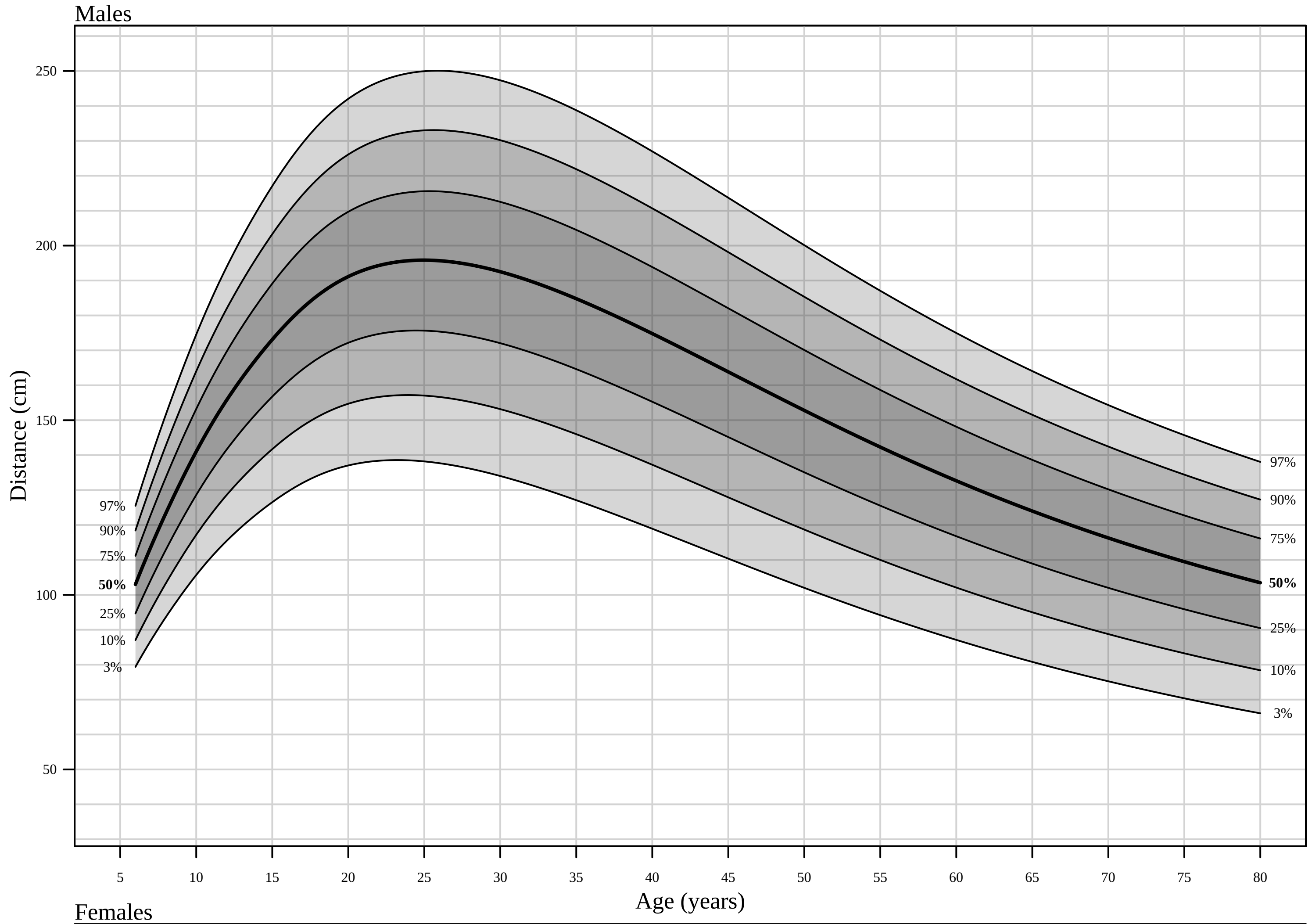

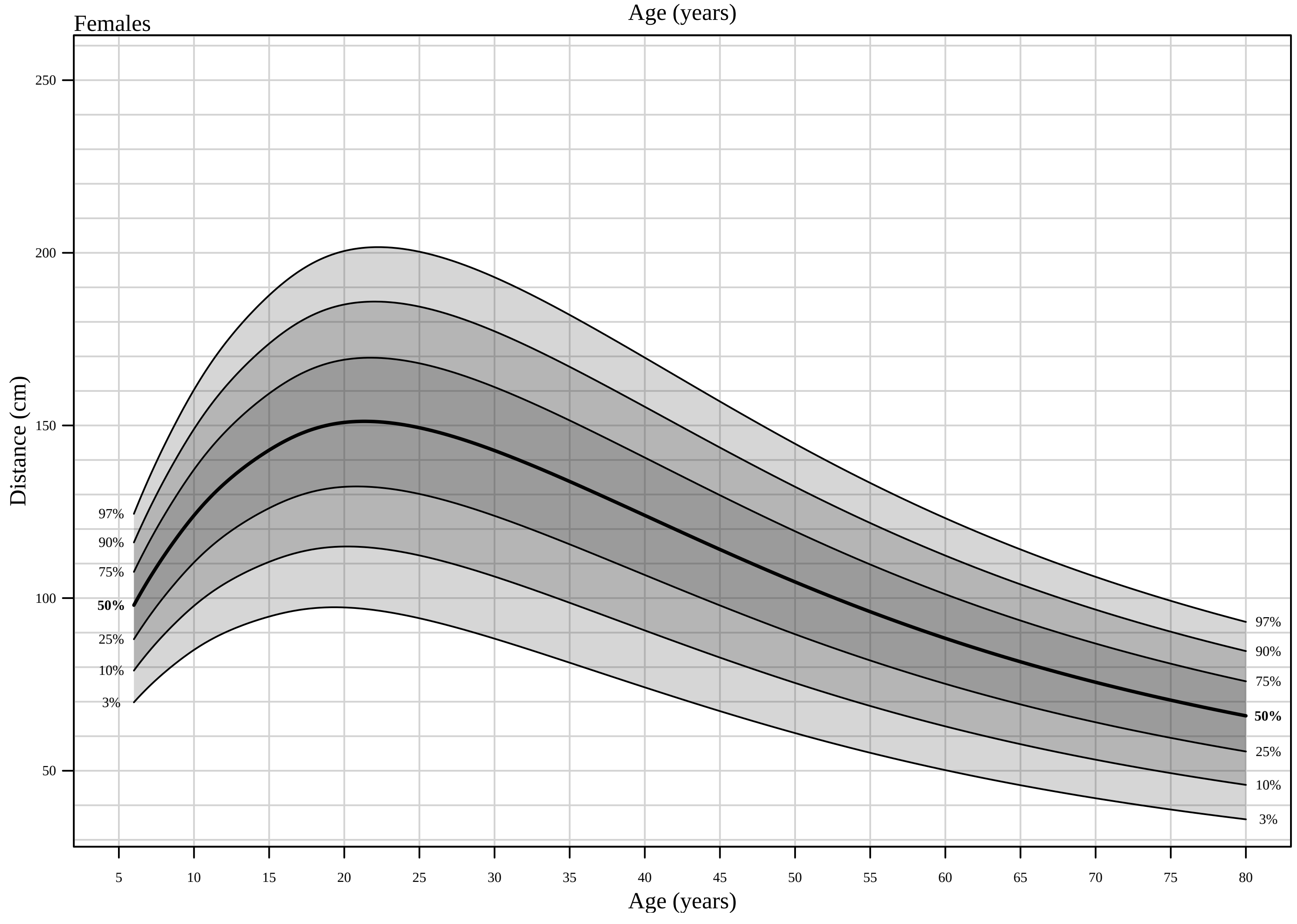

Supplement: Supplementary file 3 [file Data_Sheet_3.pdf]
