# Supplementary material for: Neuromotor functions across the lifespan: percentiles from 6 to 80 years
Source: Front Aging Neurosci. 2025 Jul 29;17:1543408. doi: 10.3389/fnagi.2025.1543408 (PMC12340781; doi:10.3389/fnagi.2025.1543408)
Supplement: Supplementary file 4 [file Data_Sheet_4.pdf]

Supplement e4: Percentile curves (10, 50, 90%) displaying the changes over age for males (blue) and females (red), for timed performance and standing long jump.

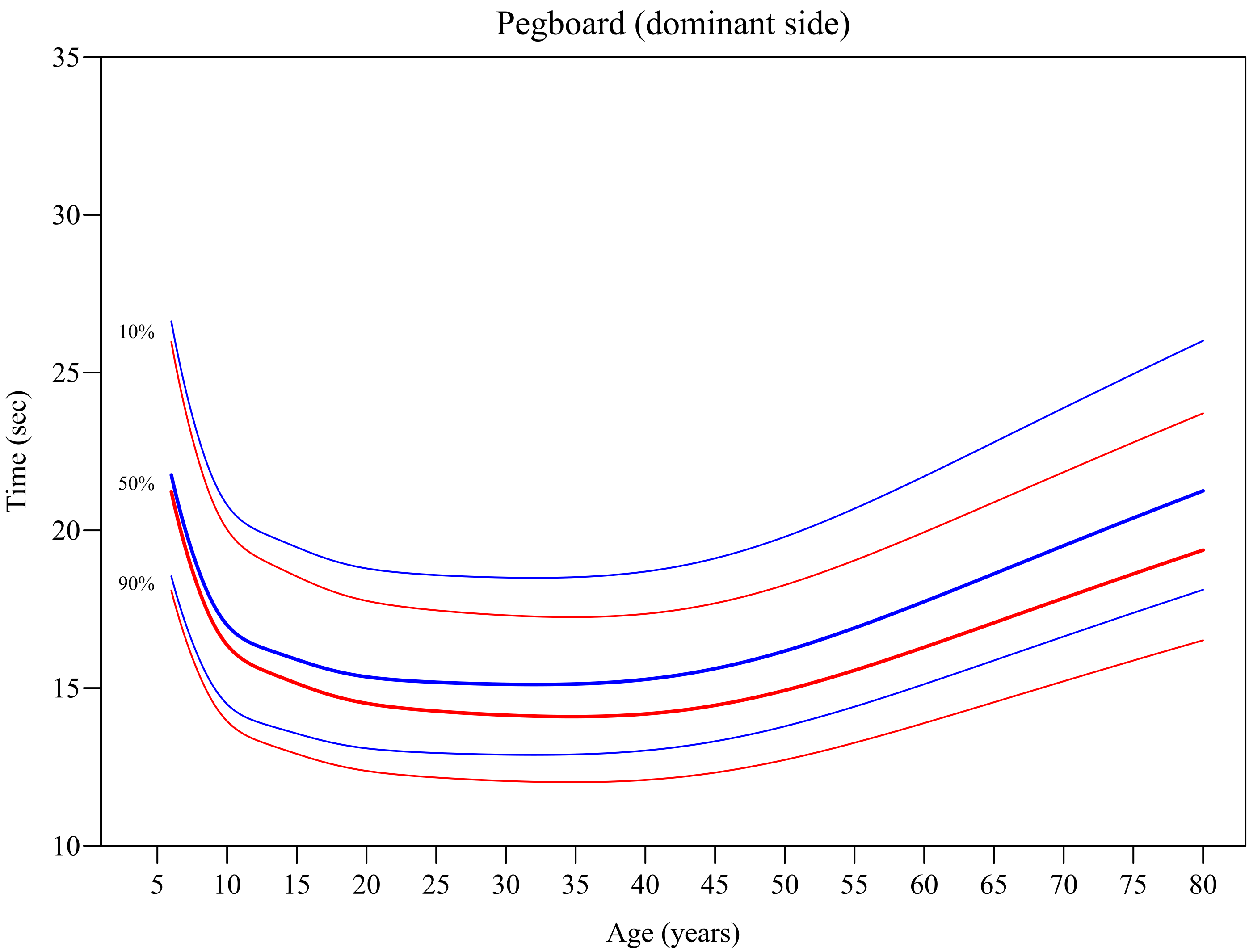

Pegboard (dominant side)
Time (sec)
Age (years)
10%
50%
90%
35
30
25
20
15
10
5
10
15
20
25
30
35
40
45
50
55
60
65
70
75
80

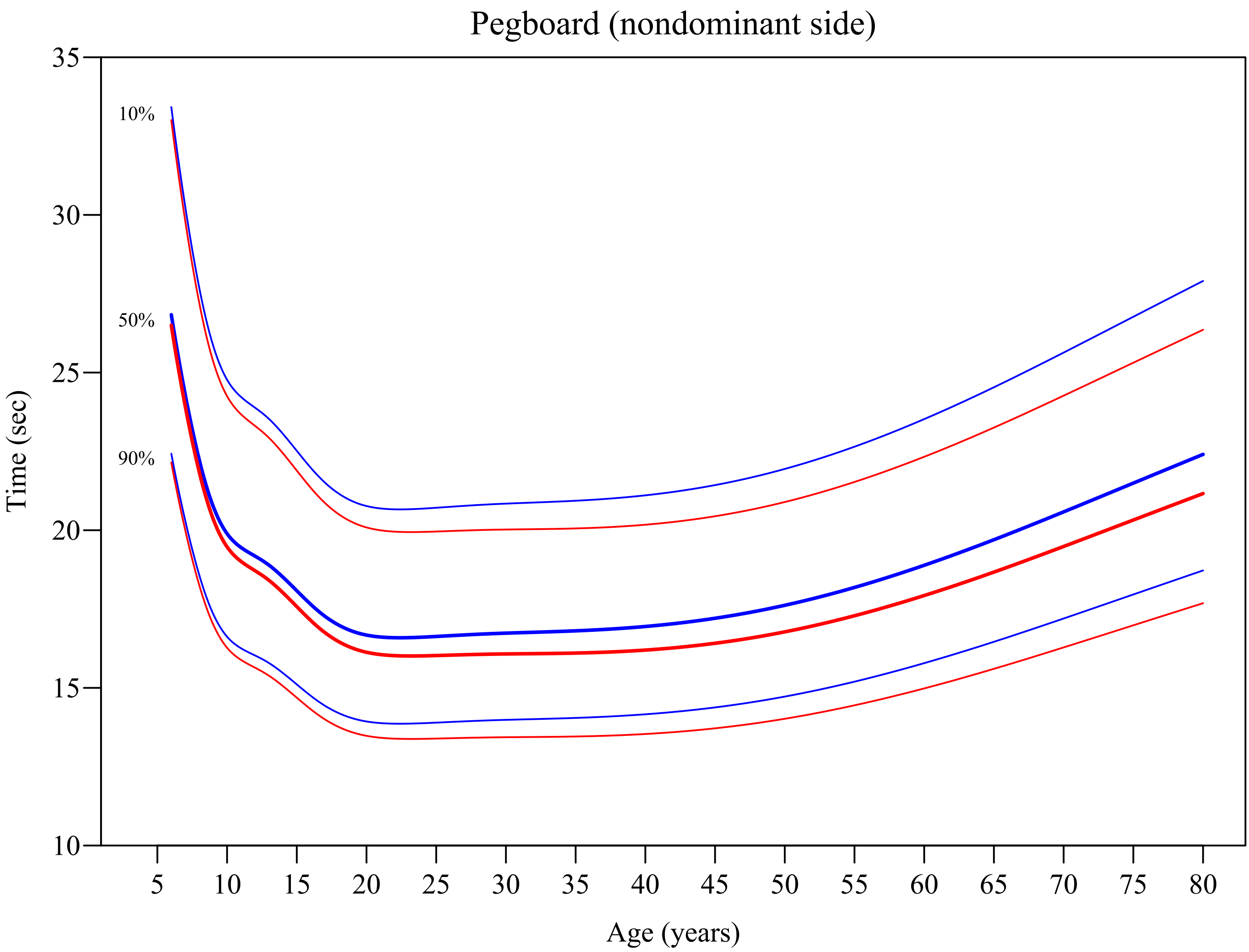

Pegboard (nondominant side)
Time (sec)
Age (years)
10%
50%
90%
35
30
25
20
15
10
5
10
15
20
25
30
35
40
45
50
55
60
65
70
75
80

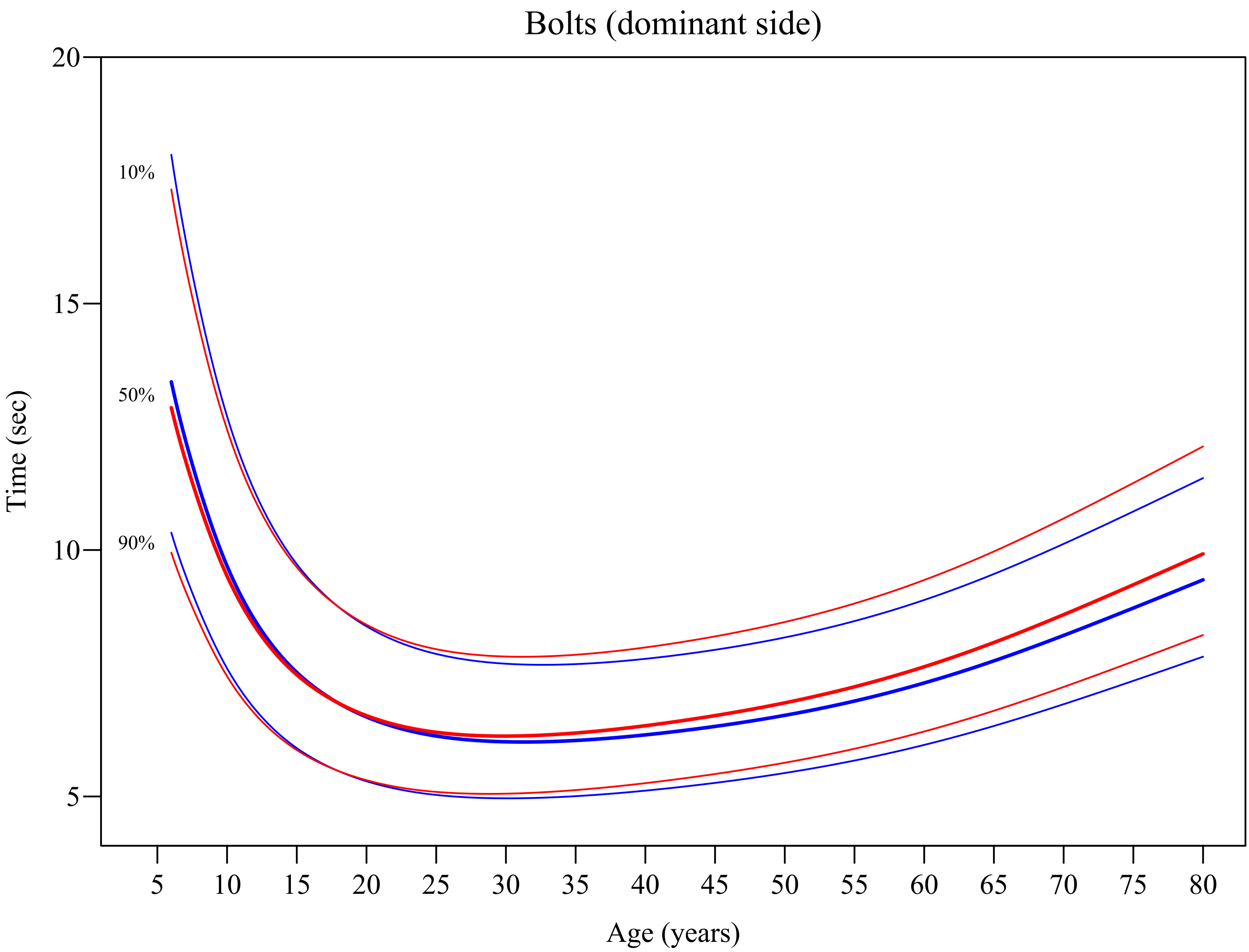
Bolts (dominant side)
Time (sec)
Age (years)
10%
50%
90%
20
15
10
5
5 10 15 20 25 30 35 40 45 50 55 60 65 70 75 80

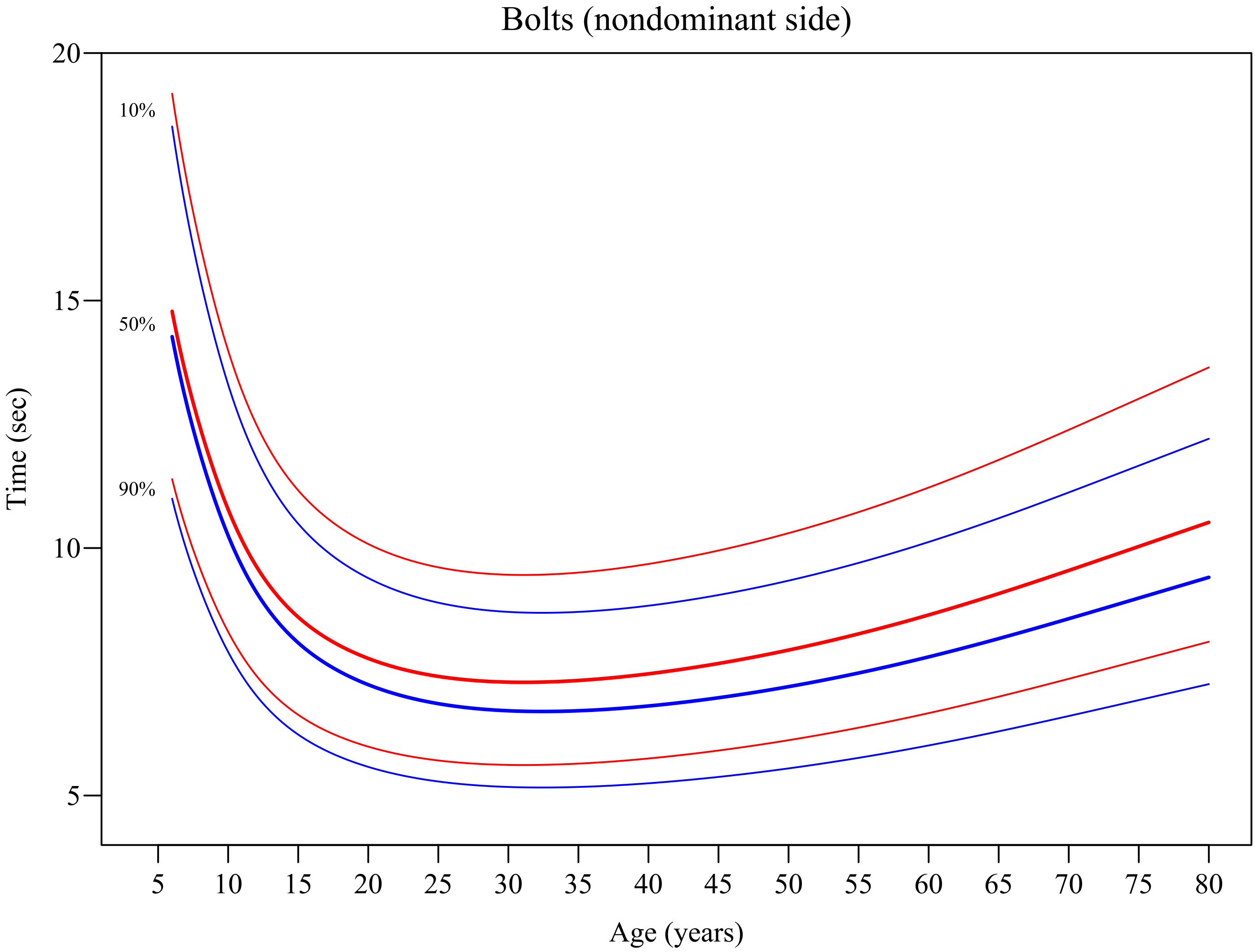
Bolts (nondominant side)
Time (sec)
Age (years)
10%
50%
90%
20
15
10
5
5 10 15 20 25 30 35 40 45 50 55 60 65 70 75 80

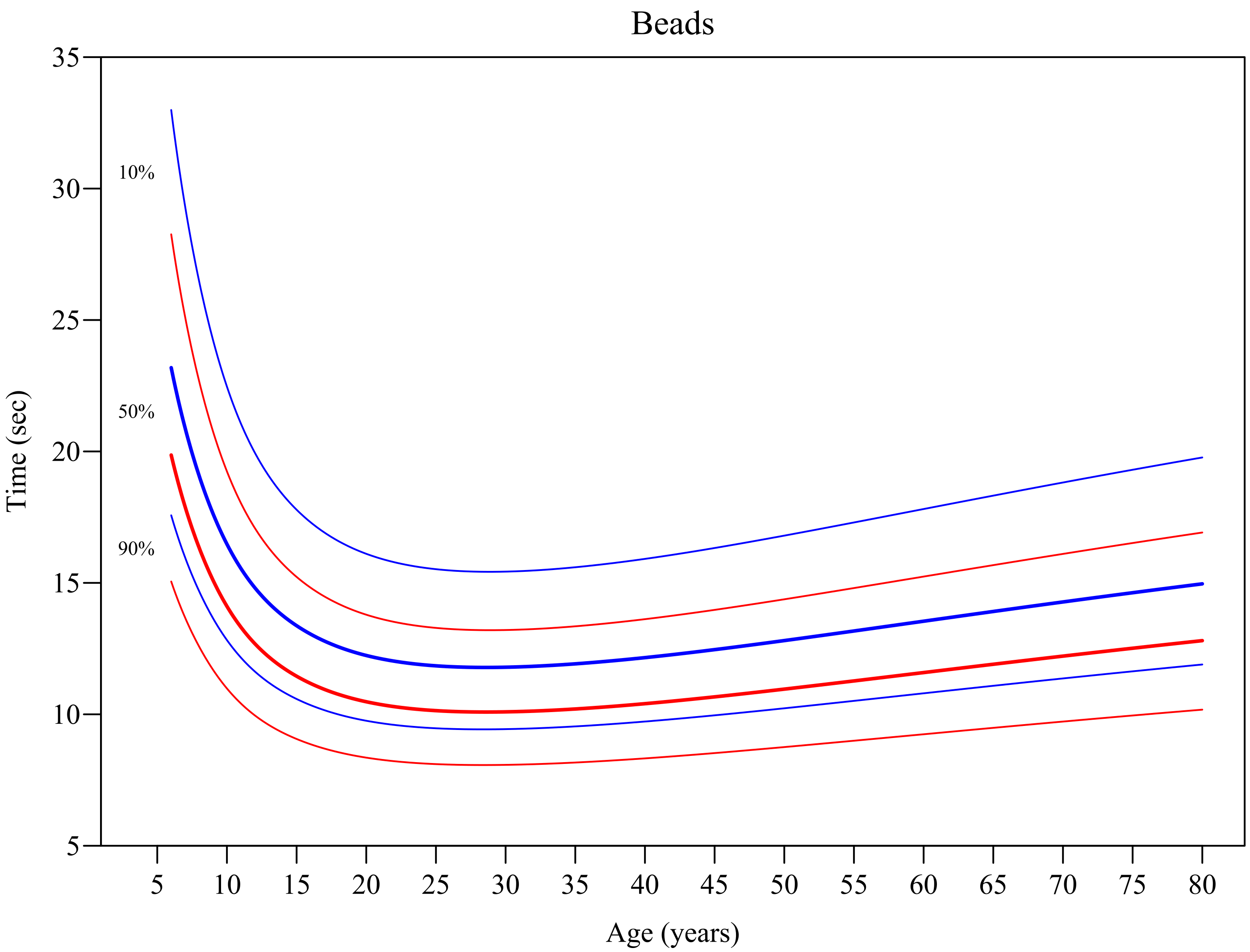

Beads
Time (sec)
Age (years)
10%
50%
90%
35
30
25
20
15
10
5
5
10
15
20
25
30
35
40
45
50
55
60
65
70
75
80

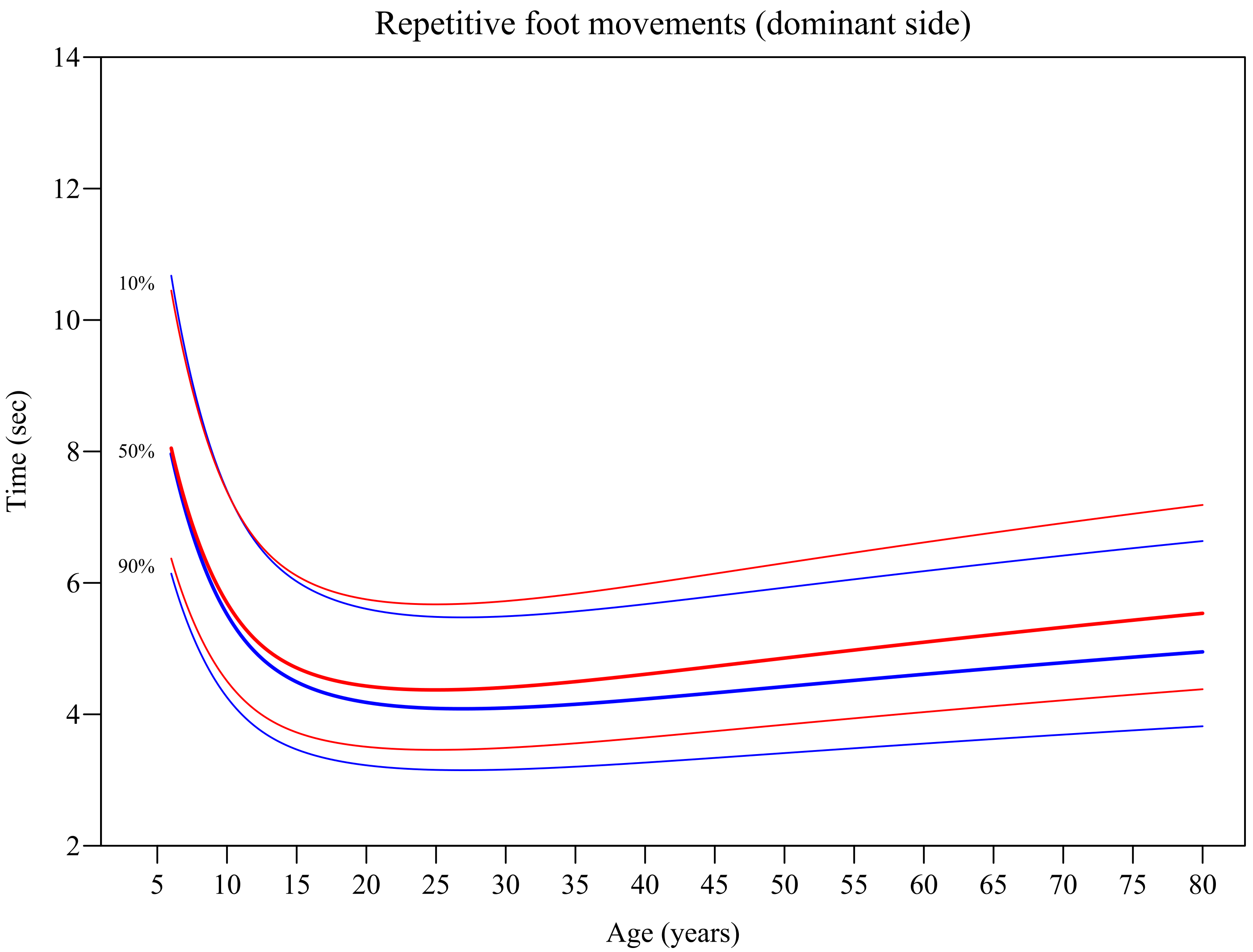

Repetitive foot movements (dominant side)
14
12
10
8
6
4
2
10%
50%
90%
Time (sec)
5
10
15
20
25
30
35
40
45
50
55
60
65
70
75
80
Age (years)

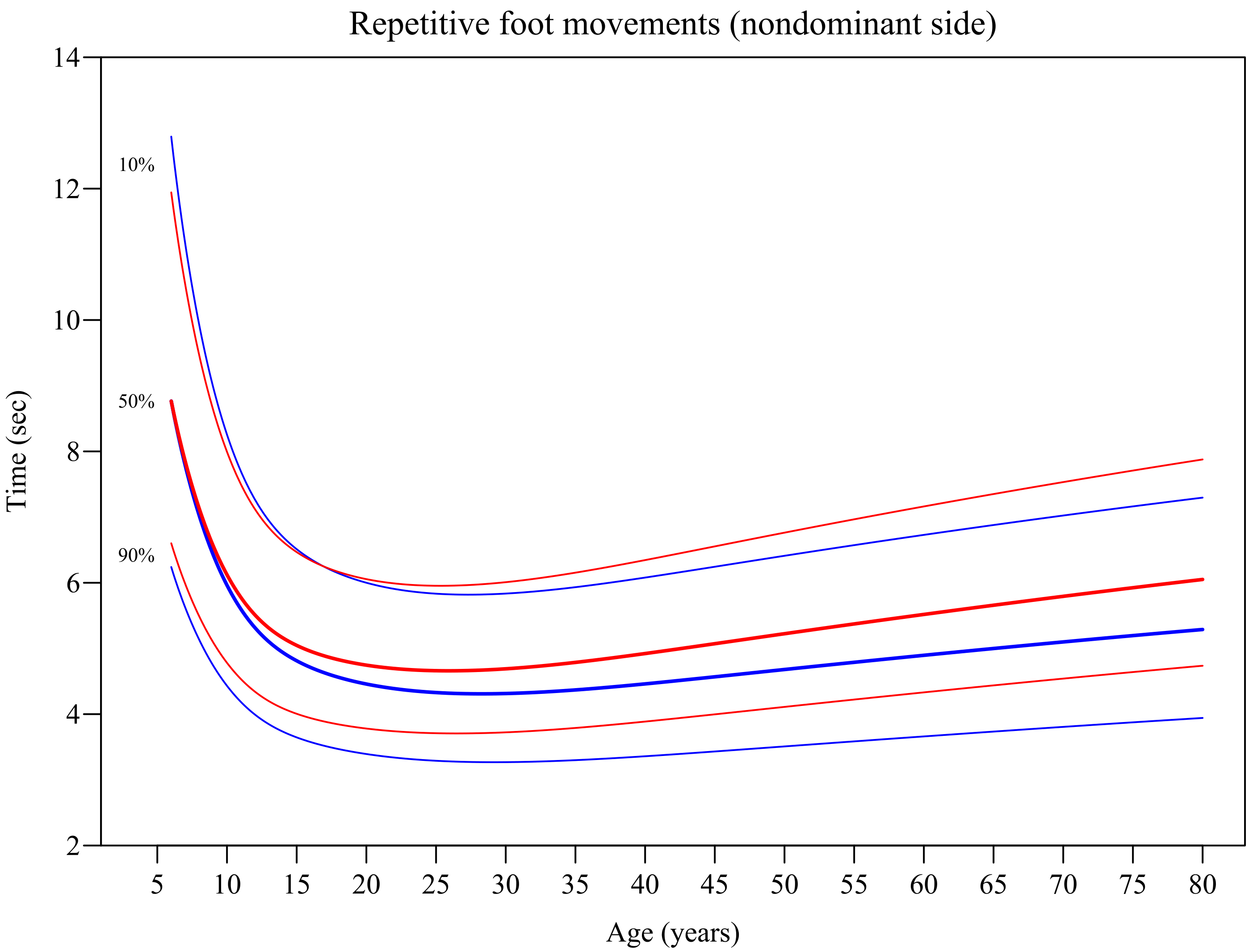

Repetitive foot movements (nondominant side)
14
12
10
8
6
4
2
10%
50%
90%
Time (sec)
5
10
15
20
25
30
35
40
45
50
55
60
65
70
75
80
Age (years)

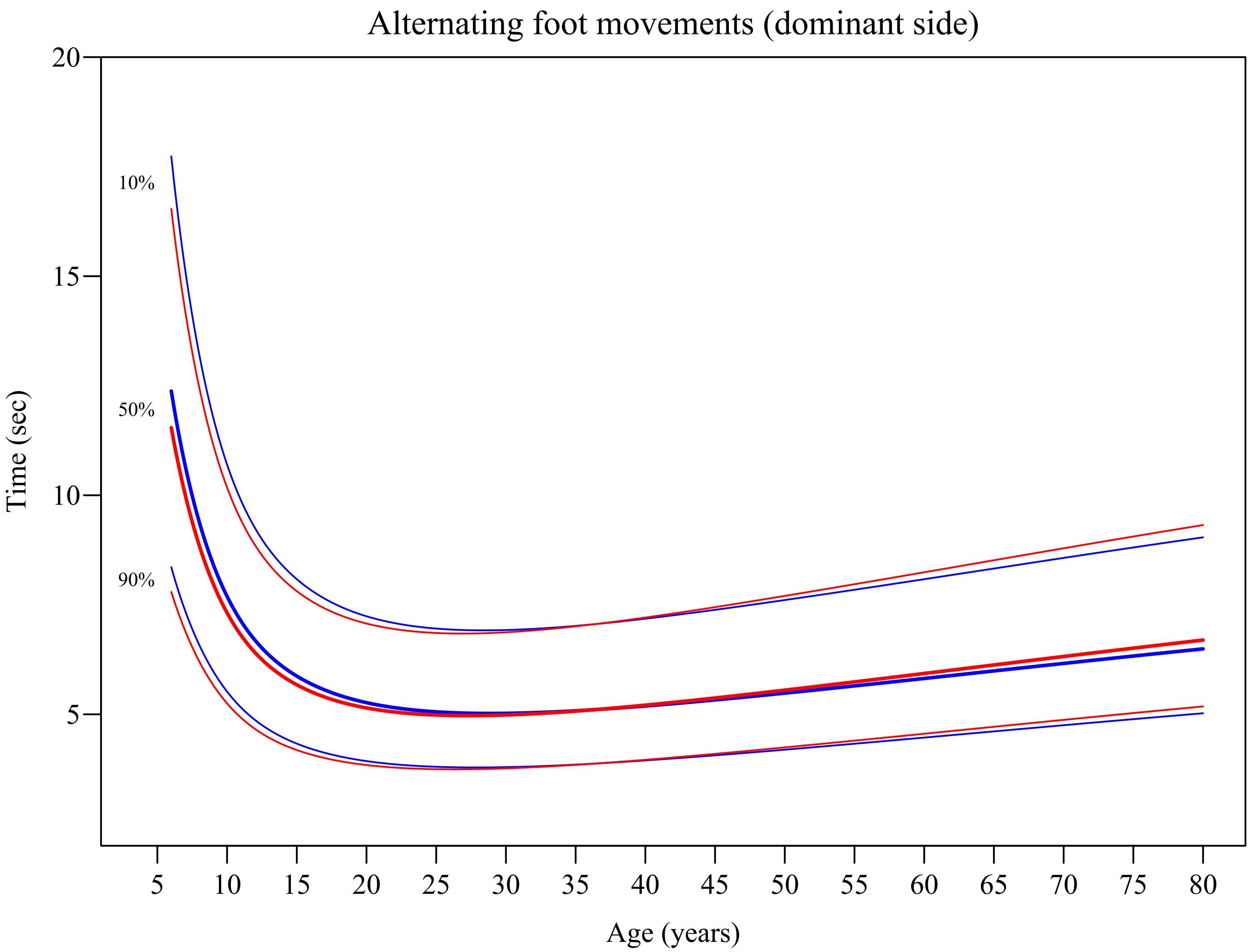

Alternating foot movements (dominant side)
Time (sec)
Age (years)
10%
50%
90%
20
15
10
5
5 10 15 20 25 30 35 40 45 50 55 60 65 70 75 80

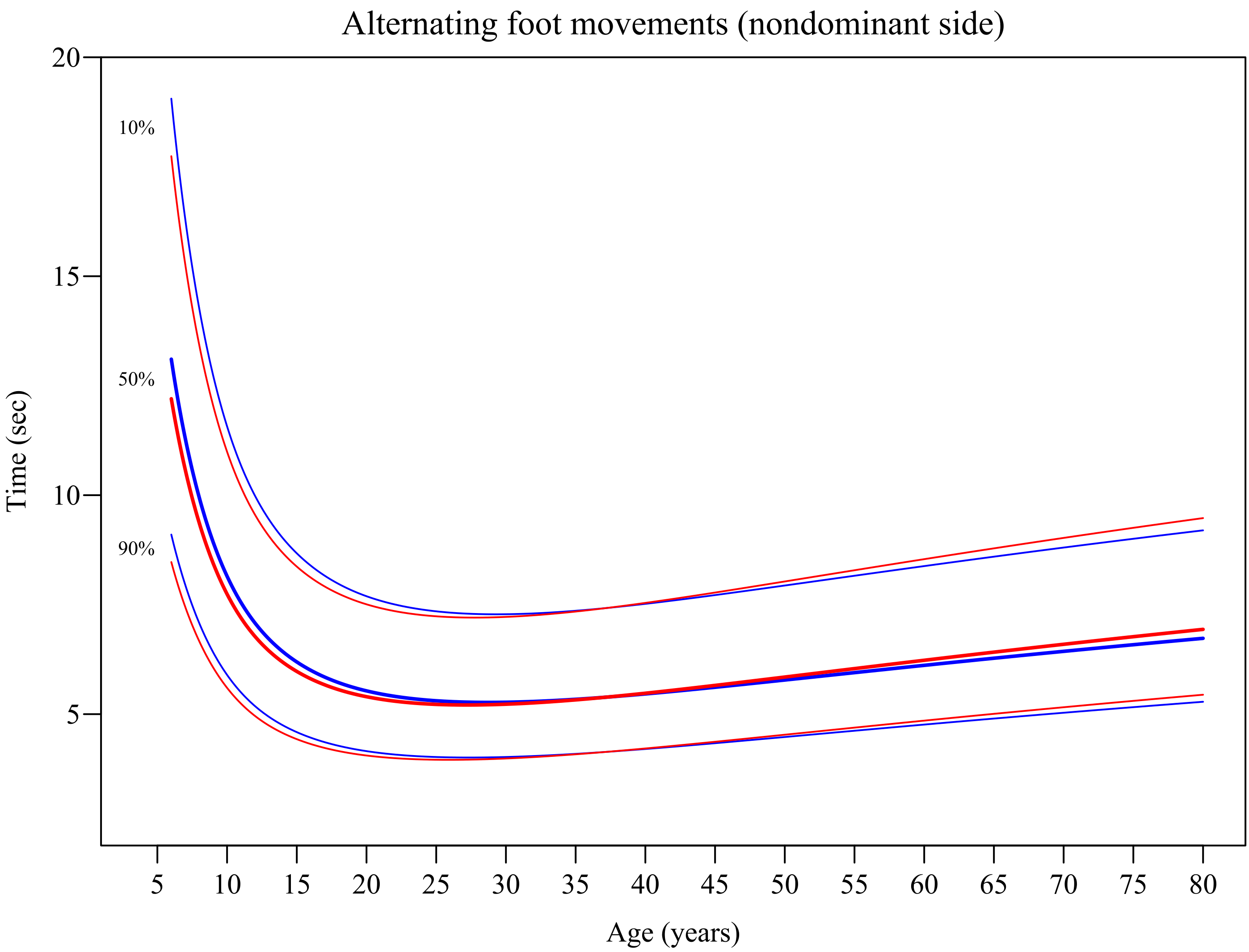

Alternating foot movements (nondominant side)
Time (sec)
Age (years)
10%
50%
90%
20
15
10
5
5 10 15 20 25 30 35 40 45 50 55 60 65 70 75 80

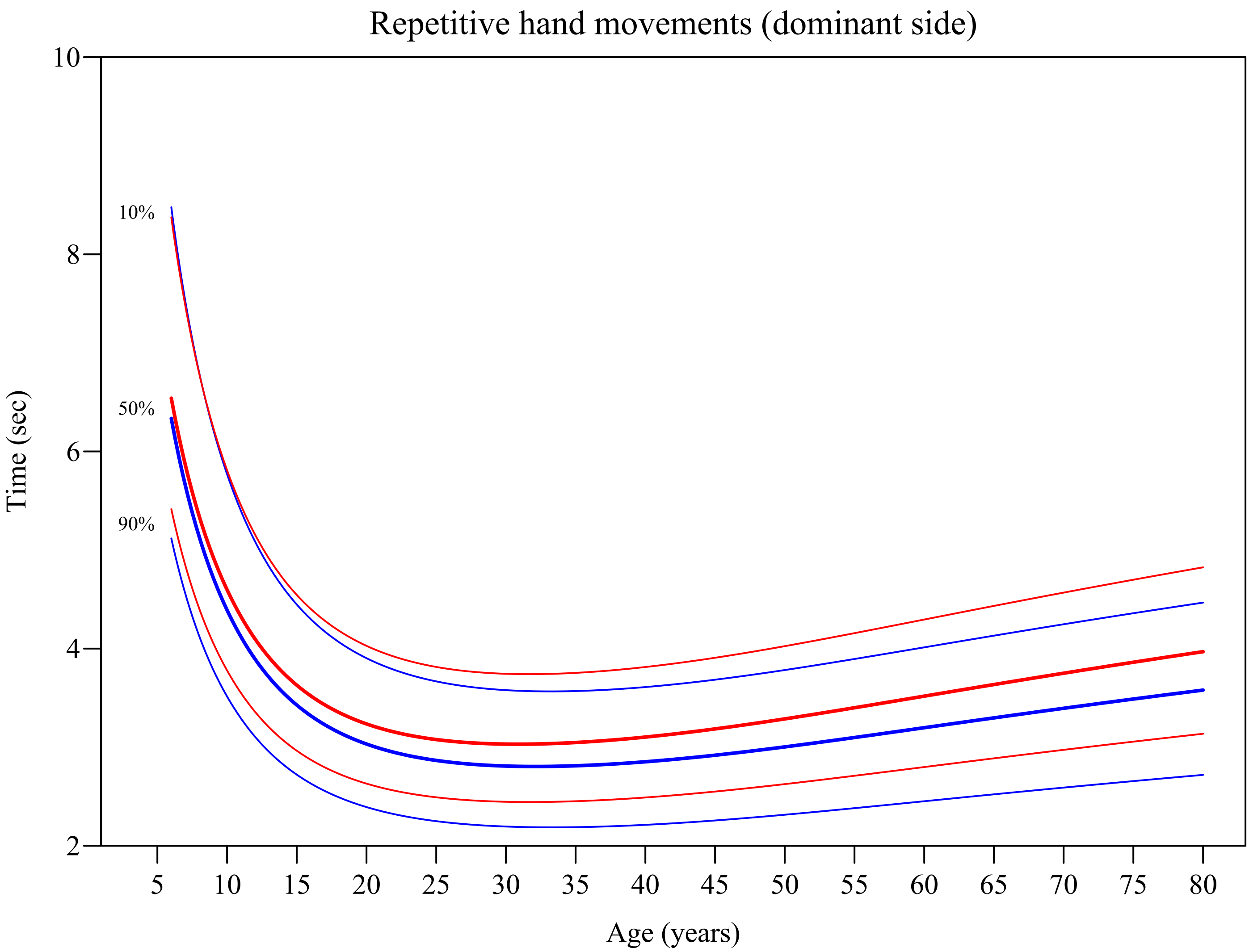
Repetitive hand movements (dominant side)
Time (sec)
Age (years)
10%
50%
90%
2
4
6
8
10
5
10
15
20
25
30
35
40
45
50
55
60
65
70
75
80

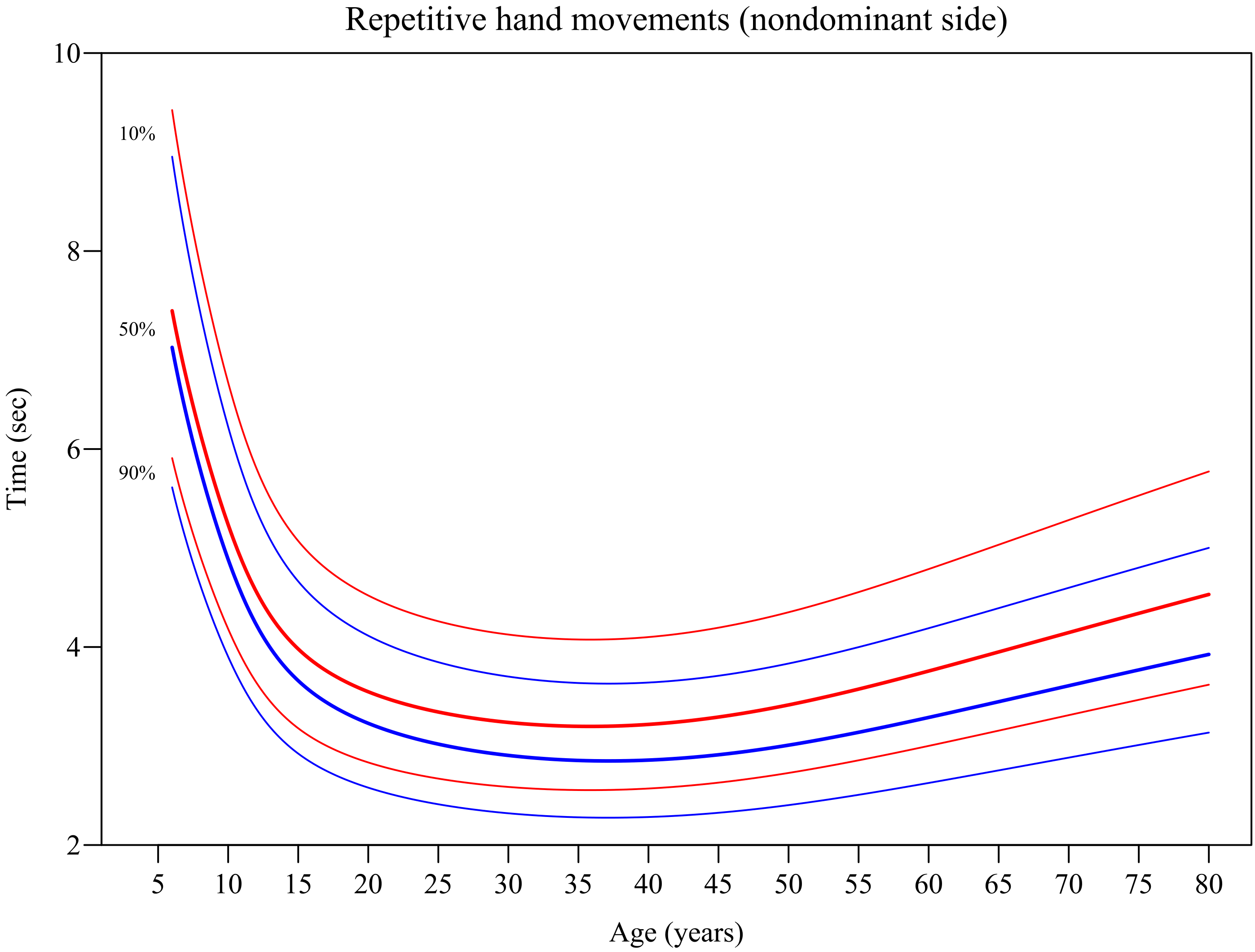
Repetitive hand movements (nondominant side)
Time (sec)
Age (years)
10%
50%
90%
2
4
6
8
10
5
10
15
20
25
30
35
40
45
50
55
60
65
70
75
80

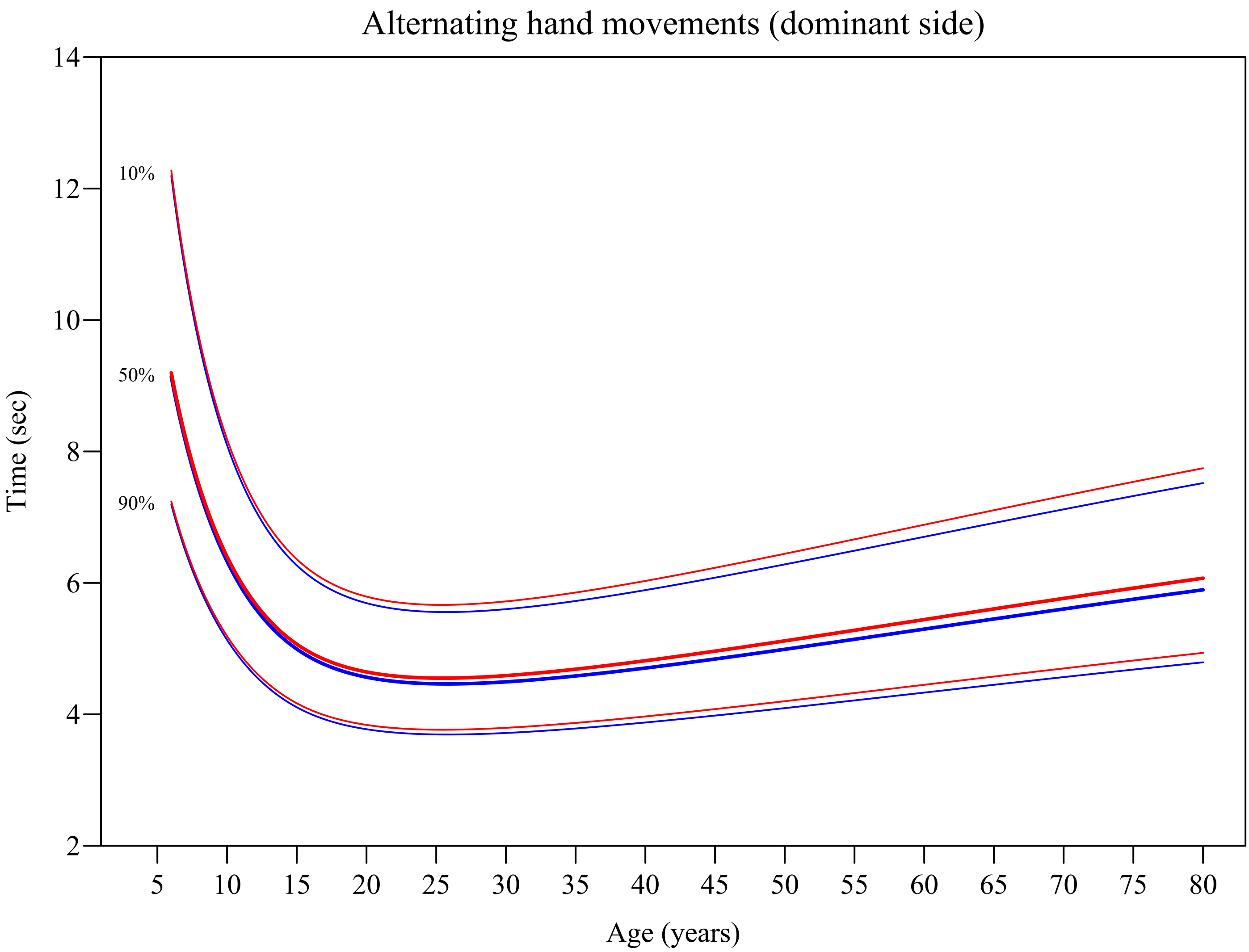
Alternating hand movements (dominant side)
Time (sec)
Age (years)
10%
50%
90%
2
4
6
8
10
12
14
5
10
15
20
25
30
35
40
45
50
55
60
65
70
75
80

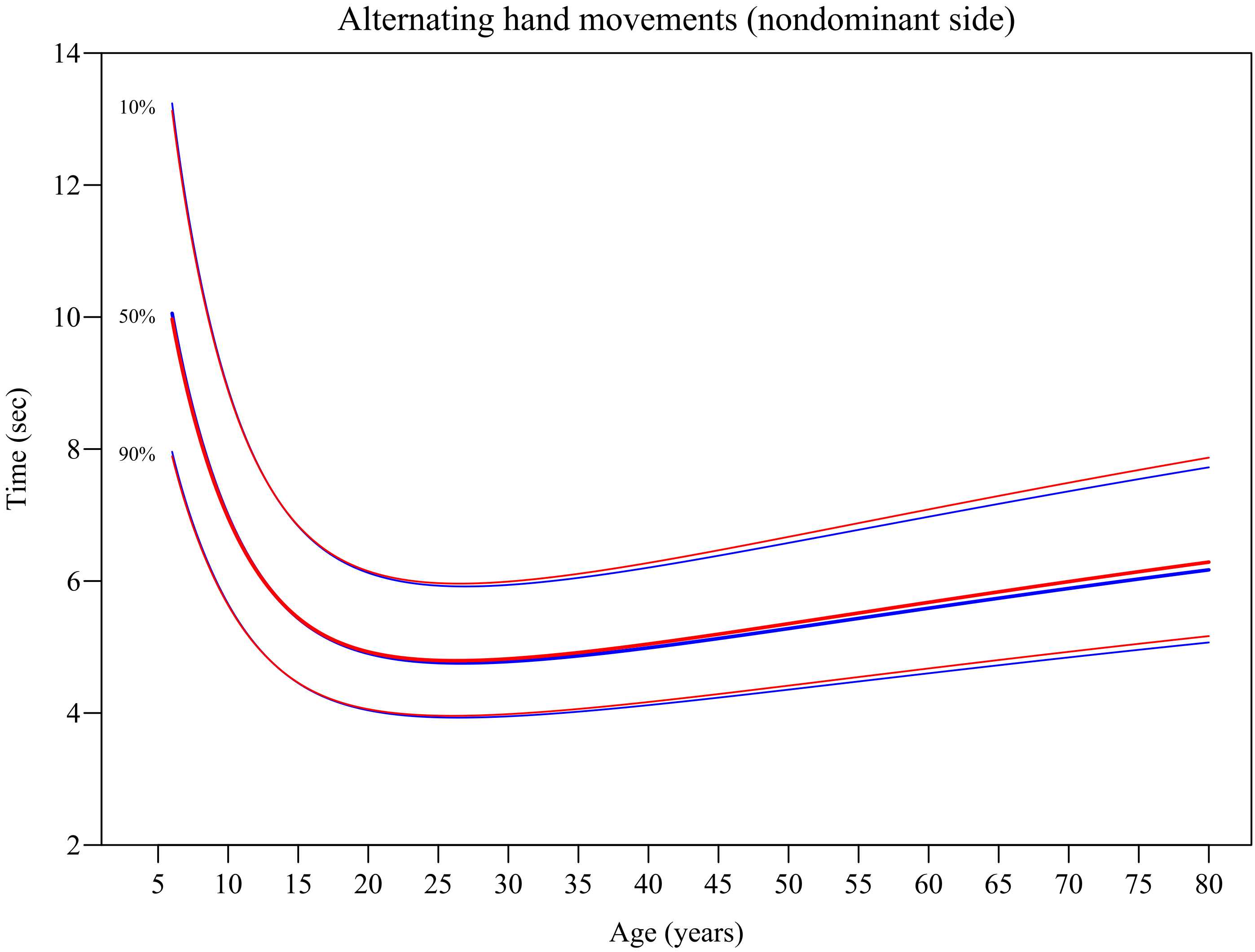
Alternating hand movements (nondominant side)
Time (sec)
Age (years)
10%
50%
90%
2
4
6
8
10
12
14
5
10
15
20
25
30
35
40
45
50
55
60
65
70
75
80

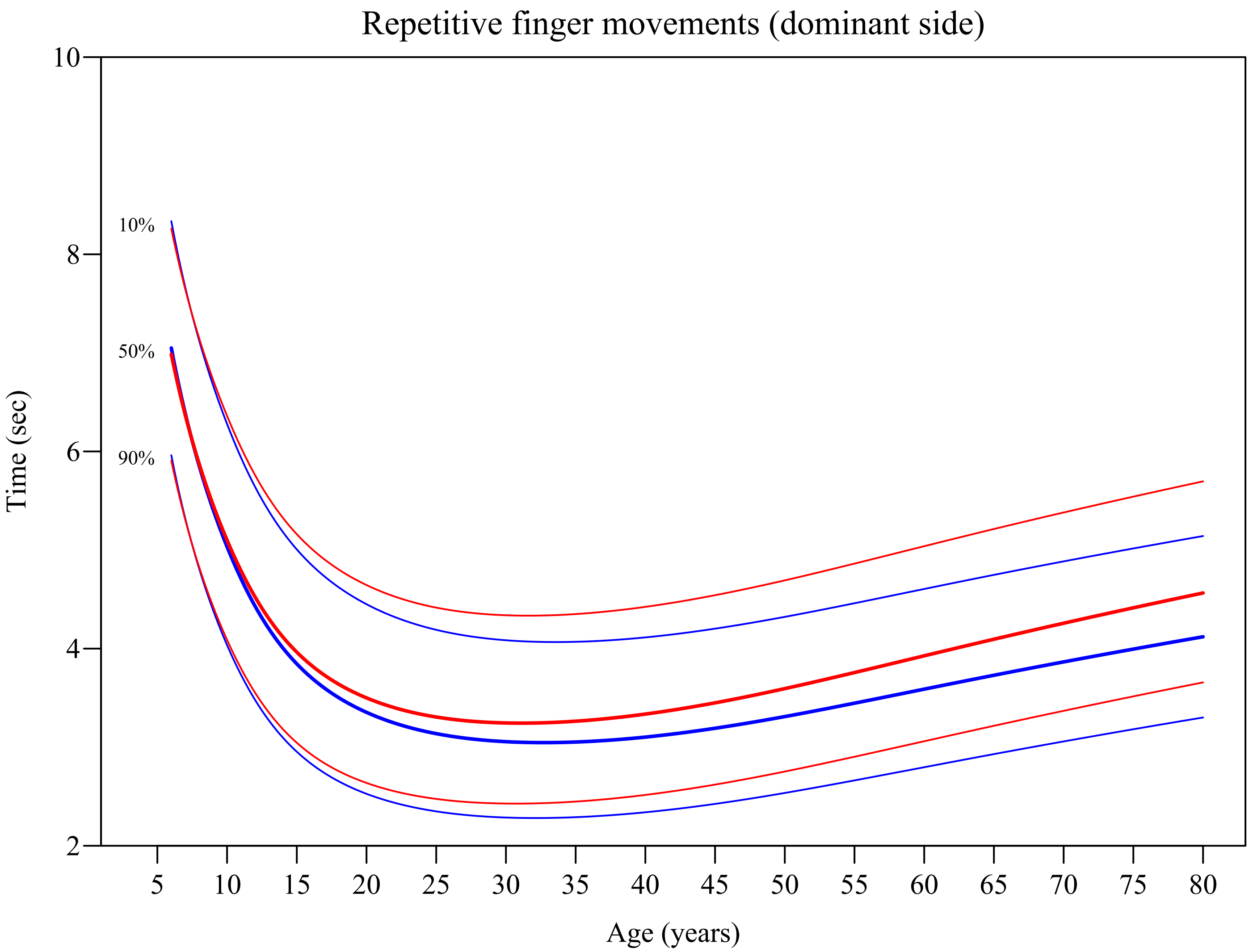
Repetitive finger movements (dominant side)
Time (sec)
Age (years)
10%
50%
90%
2
4
6
8
10
5
10
15
20
25
30
35
40
45
50
55
60
65
70
75
80

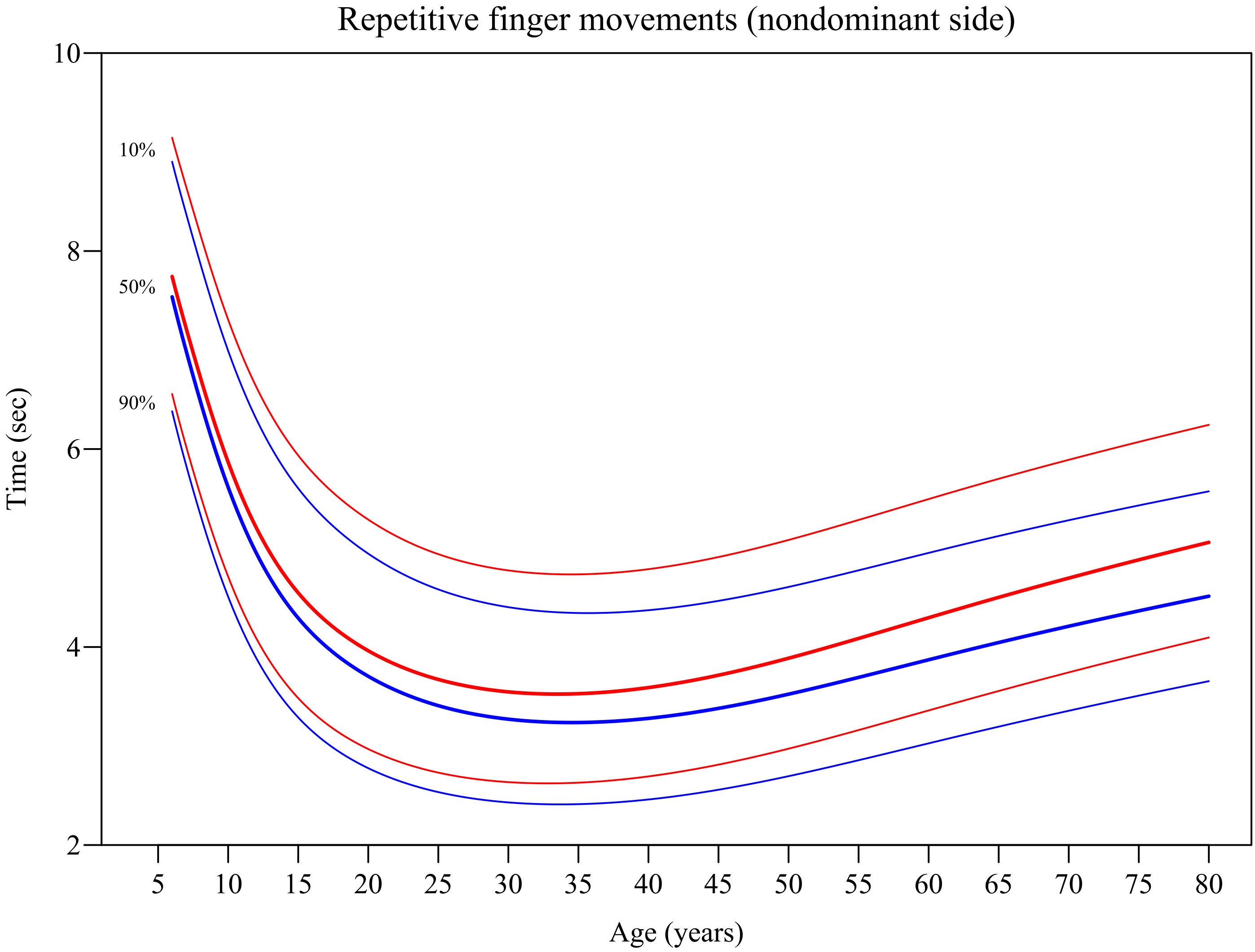
Repetitive finger movements (nondominant side)
Time (sec)
Age (years)
10%
50%
90%
2
4
6
8
10
5
10
15
20
25
30
35
40
45
50
55
60
65
70
75
80

Sequential finger movements (dominant side)

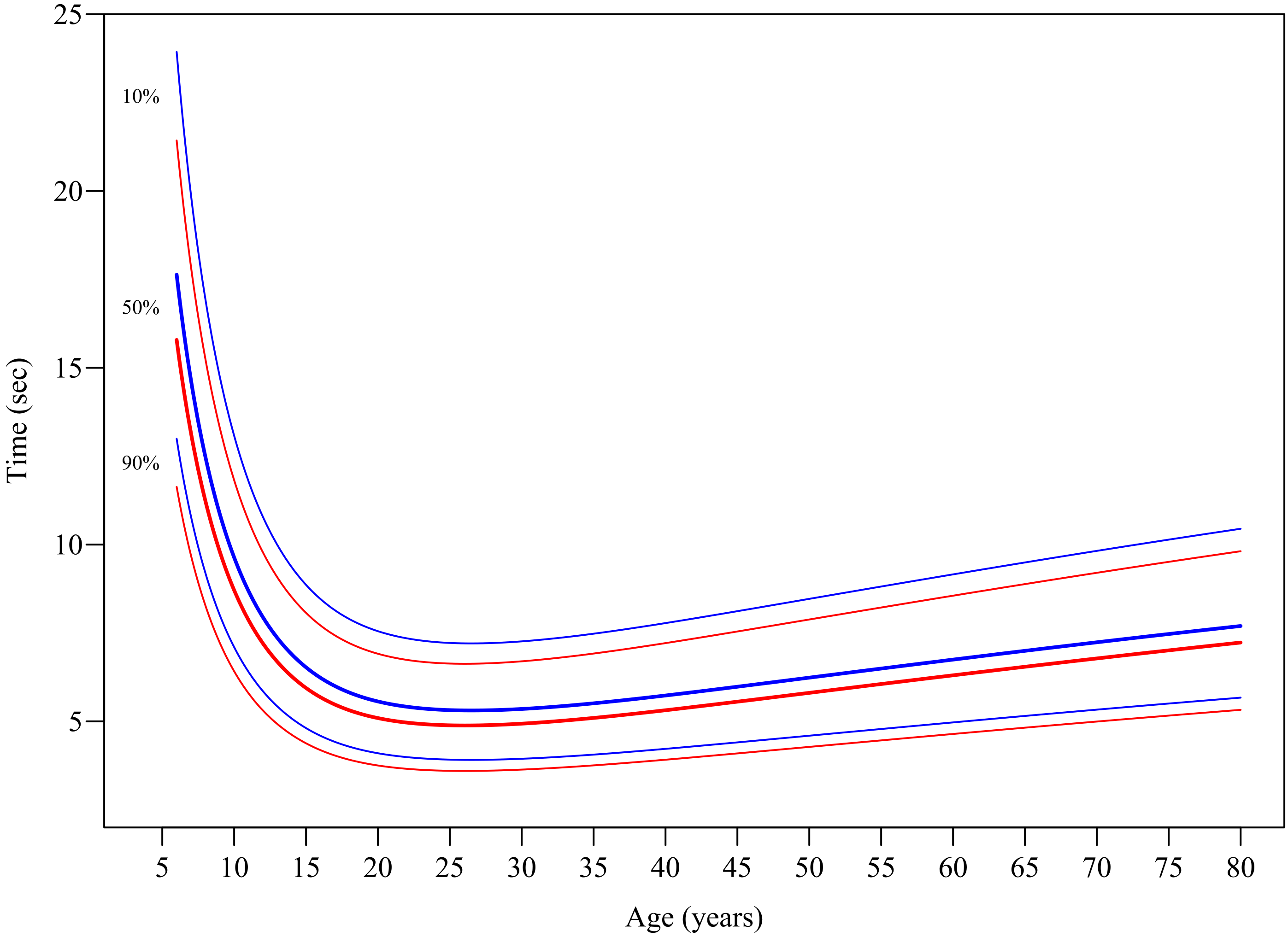

Sequential finger movements (nondominant side)

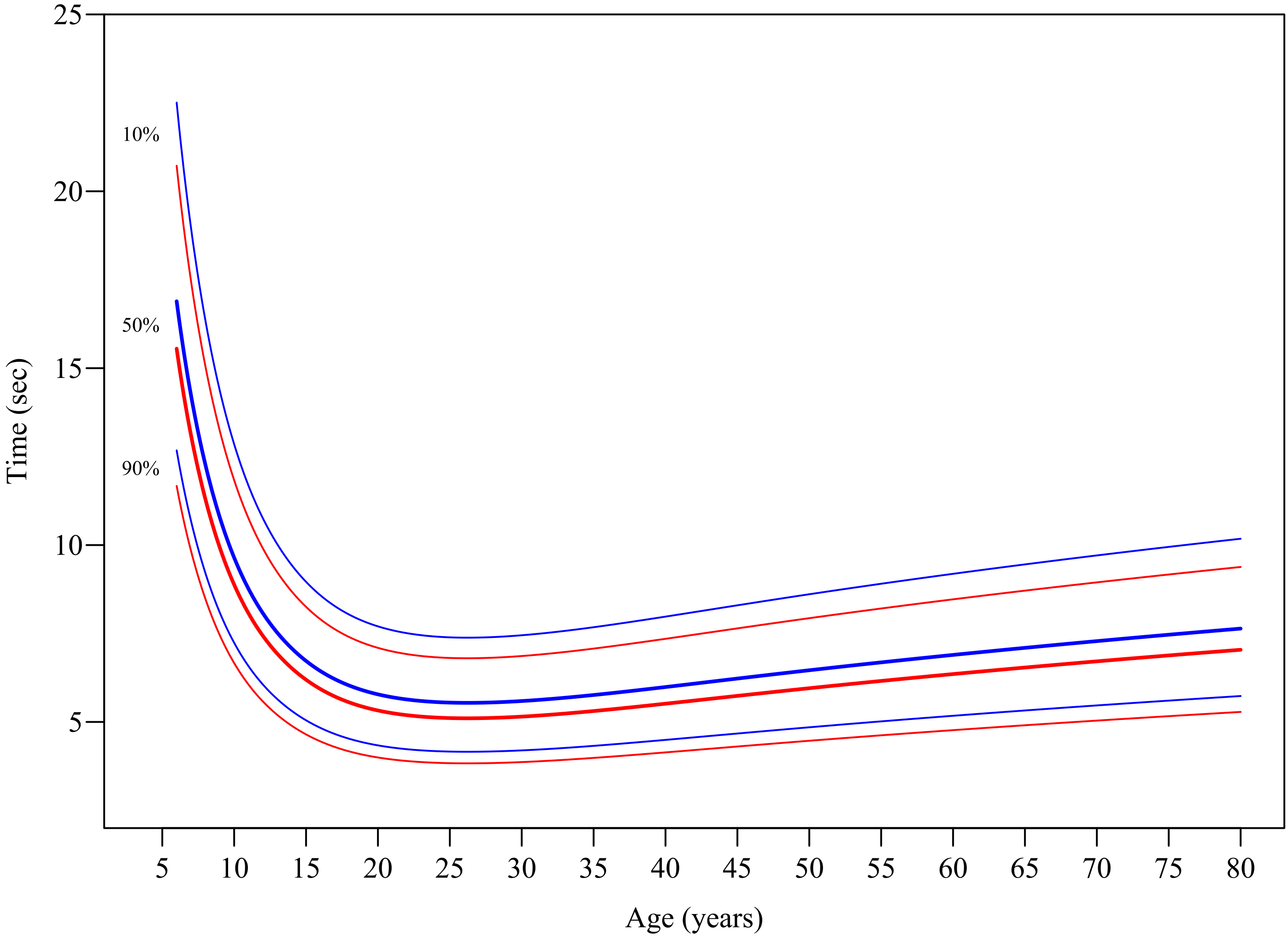

Standing on one leg with eyes open (dominant side)

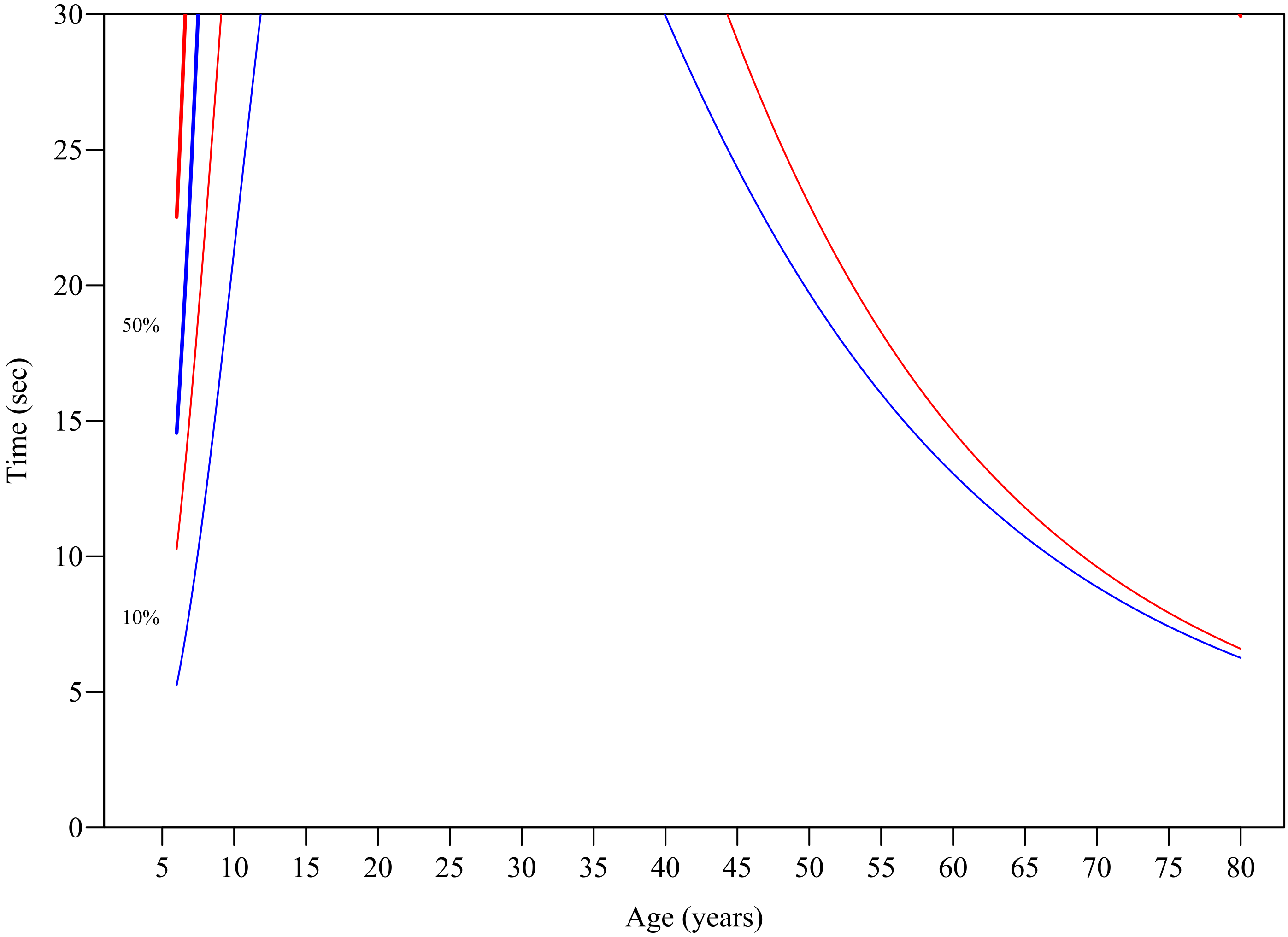

Standing on one leg with eyes open (nondominant side)

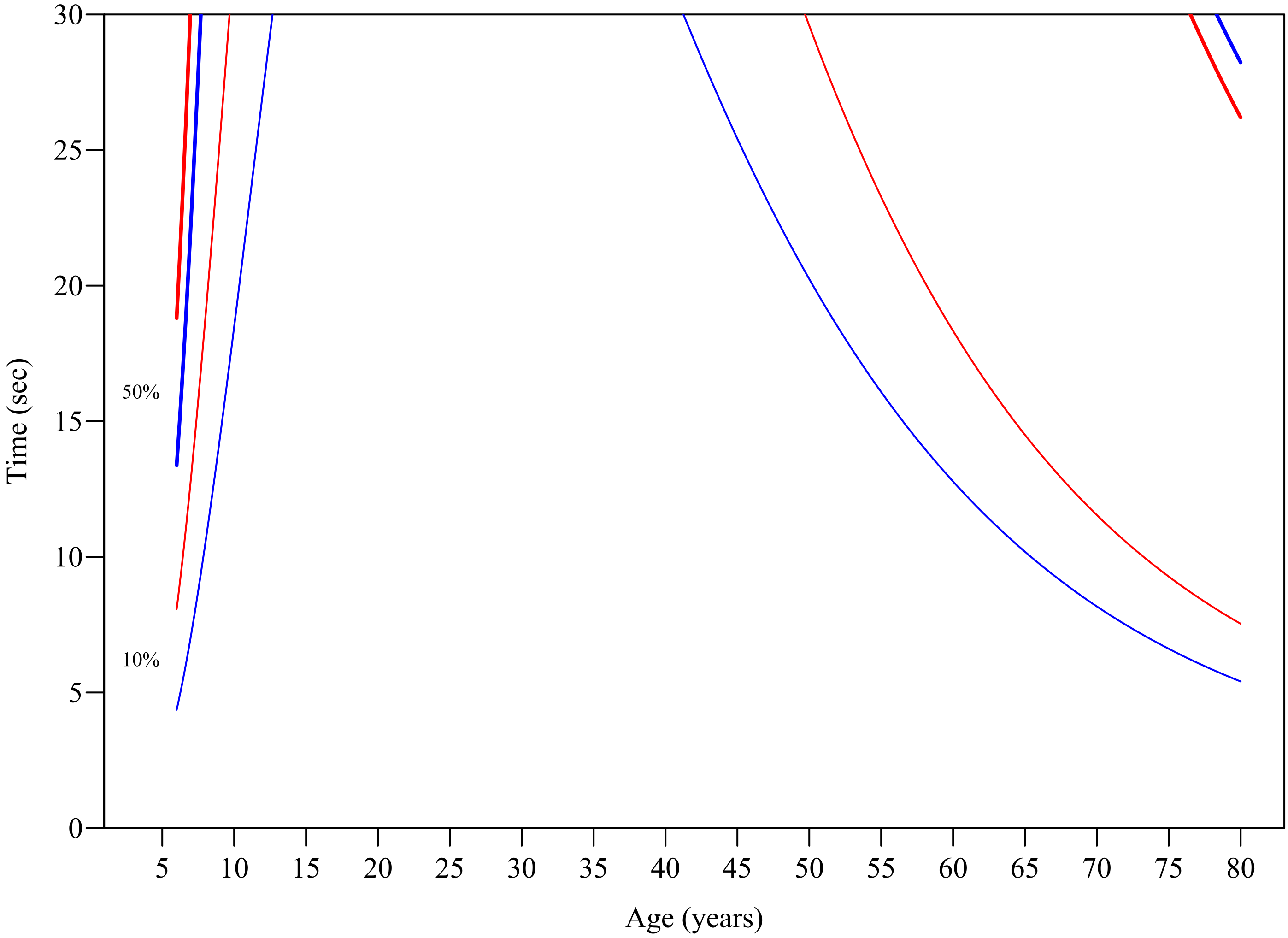

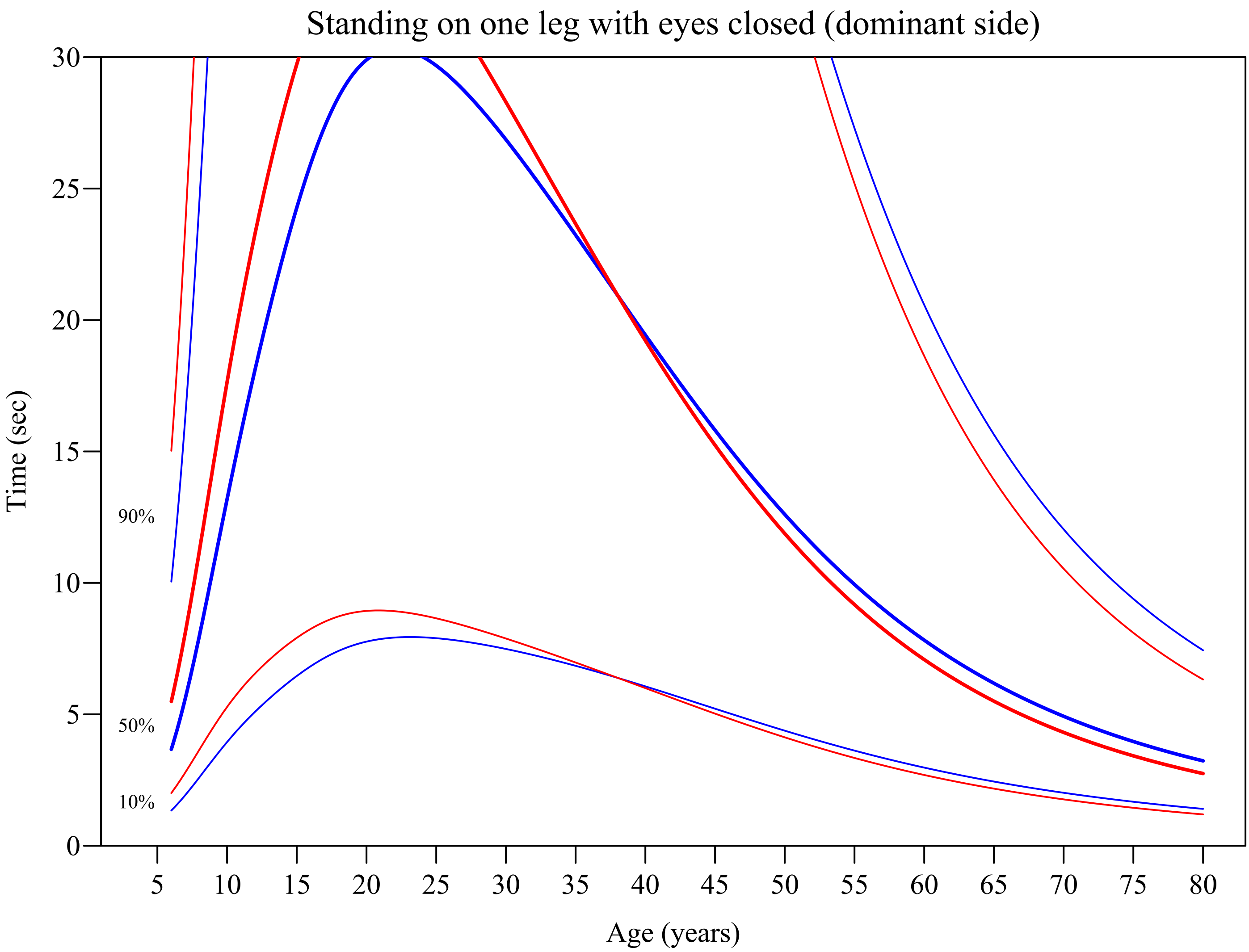

Standing on one leg with eyes closed (dominant side)
Time (sec)
Age (years)
90%
50%
10%
0
5
10
15
20
25
30
5
10
15
20
25
30
35
40
45
50
55
60
65
70
75
80

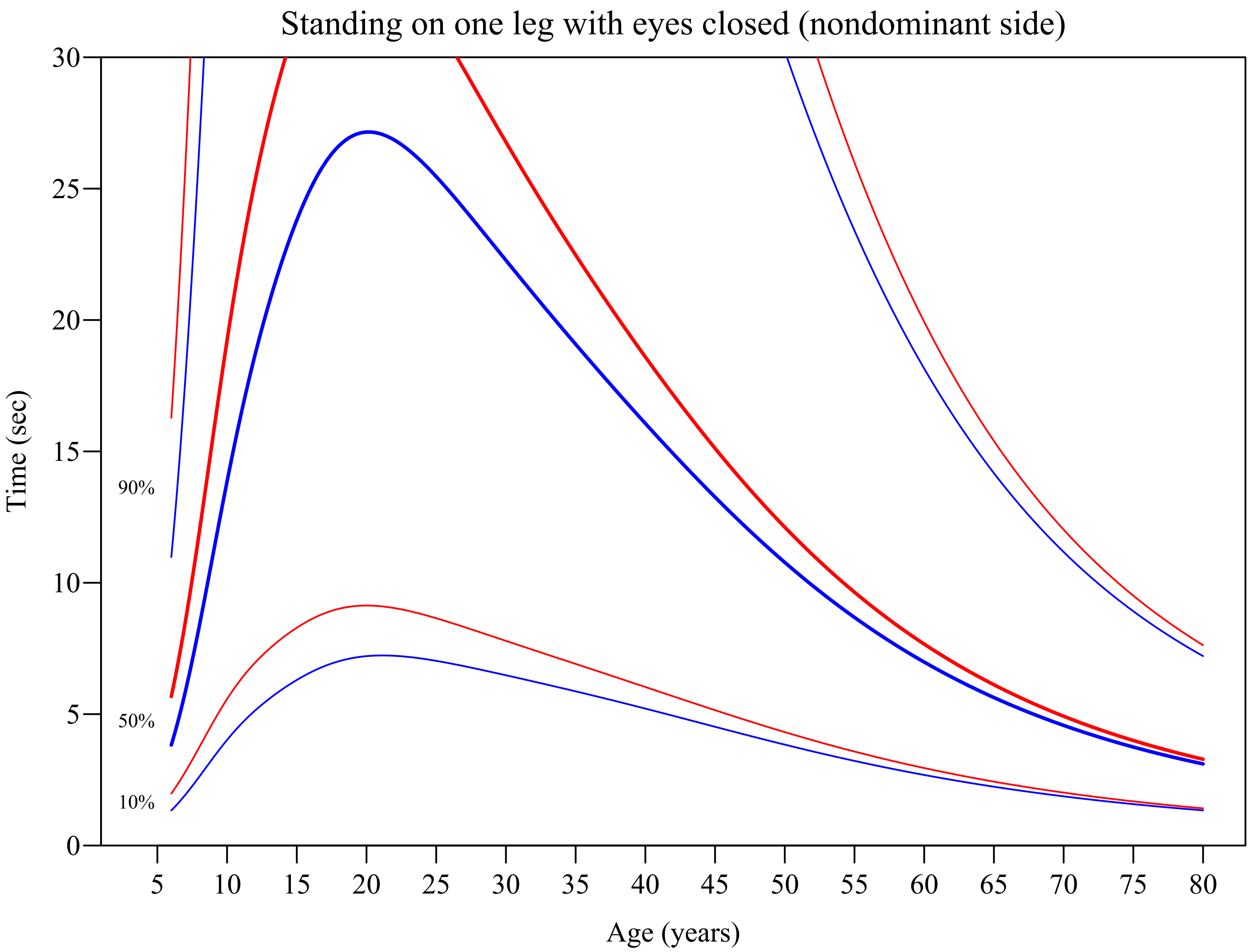

Standing on one leg with eyes closed (nondominant side)
Time (sec)
Age (years)
90%
50%
10%
0
5
10
15
20
25
30
5
10
15
20
25
30
35
40
45
50
55
60
65
70
75
80

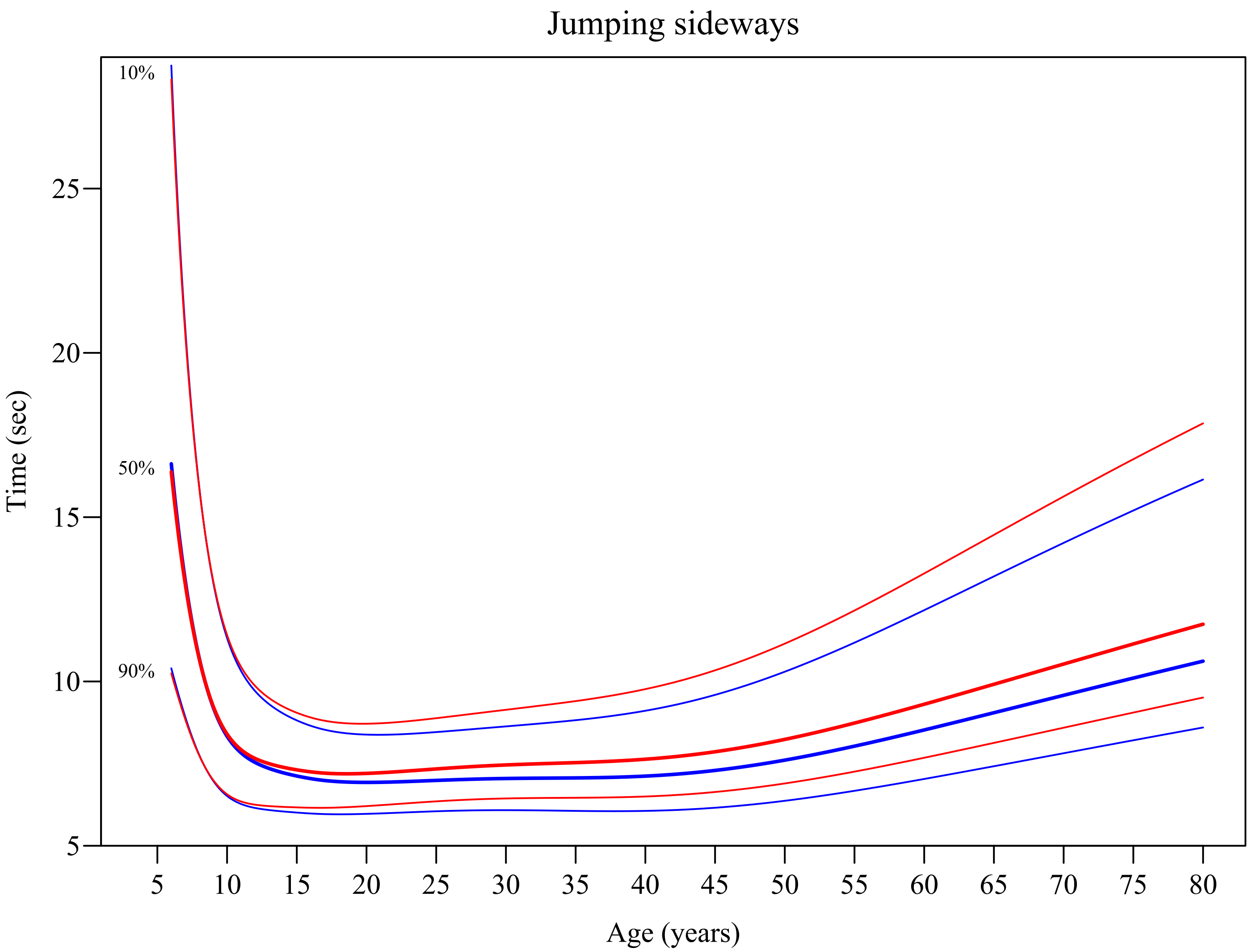
Jumping sideways
Time (sec)
Age (years)
10%
50%
90%
5
10
15
20
25
5
10
15
20
25
30
35
40
45
50
55
60
65
70
75
80

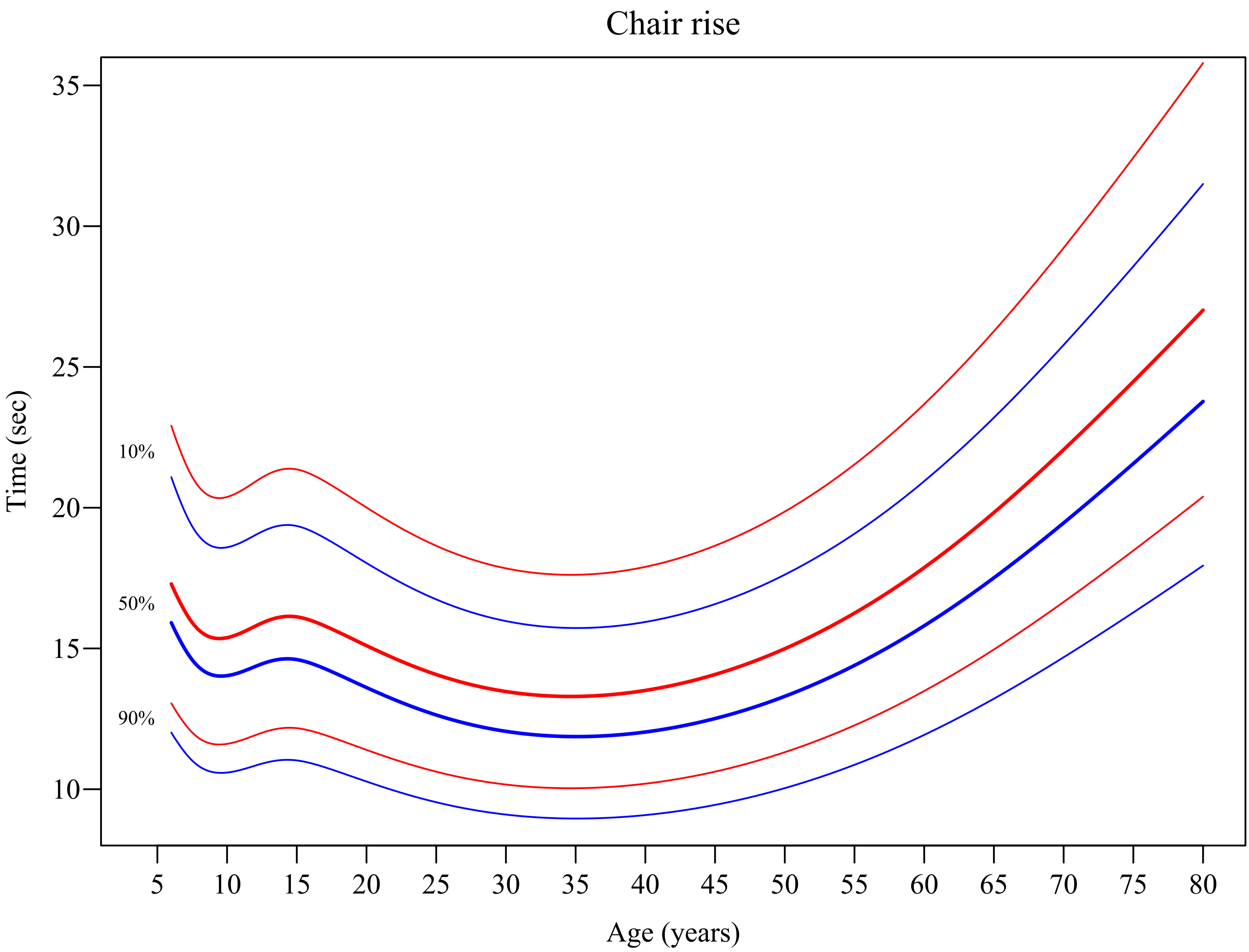
Chair rise
Time (sec)
Age (years)
10%
50%
90%
10
15
20
25
30
35
5
10
15
20
25
30
35
40
45
50
55
60
65
70
75
80

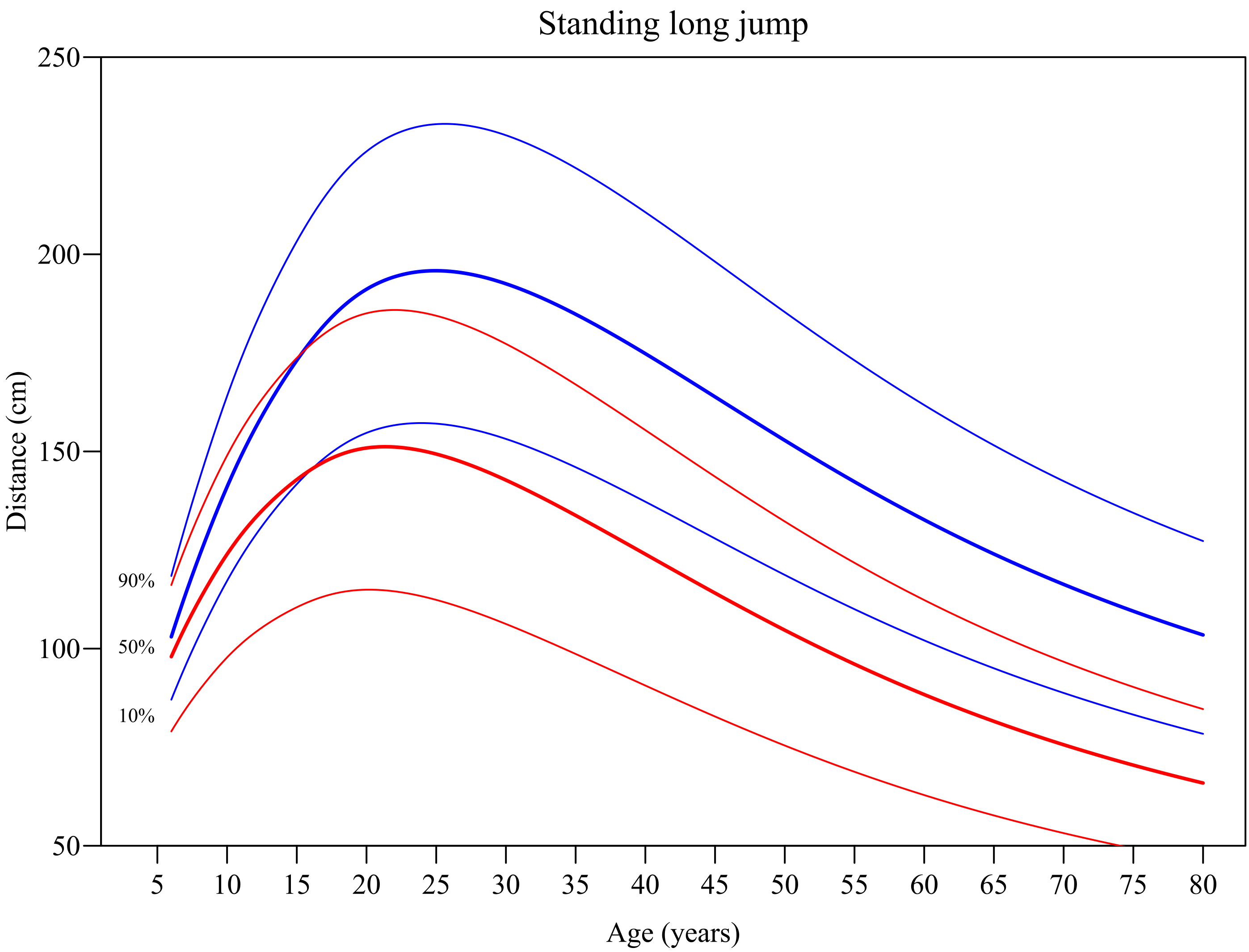

Standing long jump
250
200
150
100
50
Distance (cm)
90%
50%
10%
5
10
15
20
25
30
35
40
45
50
55
60
65
70
75
80
Age (years)
