# Supplementary material for: Neuromotor functions across the lifespan: percentiles from 6 to 80 years
Source: Front Aging Neurosci. 2025 Jul 29;17:1543408. doi: 10.3389/fnagi.2025.1543408 (PMC12340781; doi:10.3389/fnagi.2025.1543408)
Supplement: Supplementary file 5 [file Data_Sheet_5.pdf]

Supplement e5: Details on the number of measurements available in each task, with number of measurements excluded in model fit (i.e. resulting in |SDS| > 3) and the corresponding proportion of exclusions. For task abbreviations, see Table 2.

Task	Measured	Excluded	Proportion excluded (%)
Timed performance (or jumping distance):*			
PGBd	1575	2	0.1
PGBnd	1574	7	0.4
BLTd	703	3	0.4
BLTnd	704	4	0.6
BDS	704	0	0
RFTd	1574	7	0.4
RFTnd	1574	4	0.3
AFTd	1574	9	0.6
AFTnd	1574	5	0.3
RHDd	1609	13	0.8
RHDnd	1607	18	1.1
AHDd	1570	4	0.3
AHDnd	1568	3	0.2
RFGd	1607	6	0.4
RFGnd	1607	8	0.5
SFGd	1601	11	0.7
SFGnd	1599	9	0.6
SBOd	1131	6	0.5
SBOnd	1129	6	0.5
SBCd	1091	0	0
SBCnd	1094	1	0.1
JSW	1479	5	0.3
CHR	700	2	0.3
SLJ*	542	5	0.9
Intensity of contralateral associated movements (CAM):			
PGBd	1461	0	0
PGBnd	1460	1	0.1
BLTd	700	0	0
BLTnd	700	0	0
AFTd	1467	1	0.1
AFTnd	1466	1	0.1
AHDd	1467	0	0
AHDnd	1467	0	0
SFGd	1463	4	0.3
SFGnd	1463	3	0.2
