# Supplementary material for: Neuromotor functions across the lifespan: percentiles from 6 to 80 years
Source: Front Aging Neurosci. 2025 Jul 29;17:1543408. doi: 10.3389/fnagi.2025.1543408 (PMC12340781; doi:10.3389/fnagi.2025.1543408)
Supplement: Supplementary file 6 [file Data_Sheet_6.pdf]

Supplement e6: Estimated difference between 50th centile of males and females as a function of age (with 95% confidence interval), for timed performance and standing long jump. The reported p-value refers to a likelihood ratio test (LRT) comparing a model including interactions between sex and spline coefficients against a model without any sex effect.

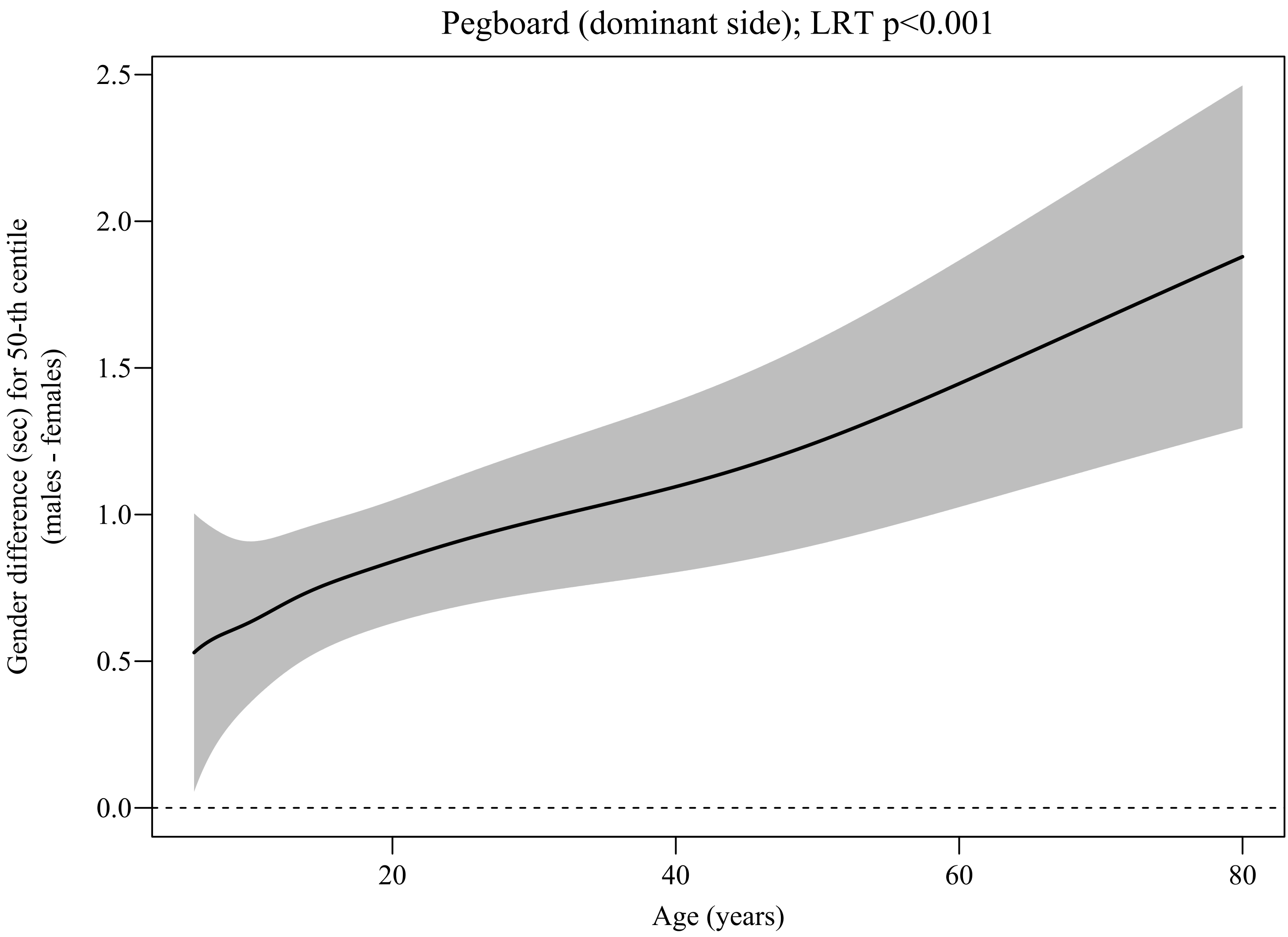

Pegboard (dominant side); LRT p<0.001
Gender difference (sec) for 50-th centile
(males - females)
Age (years)
0.0
0.5
1.0
1.5
2.0
2.5
20
40
60
80

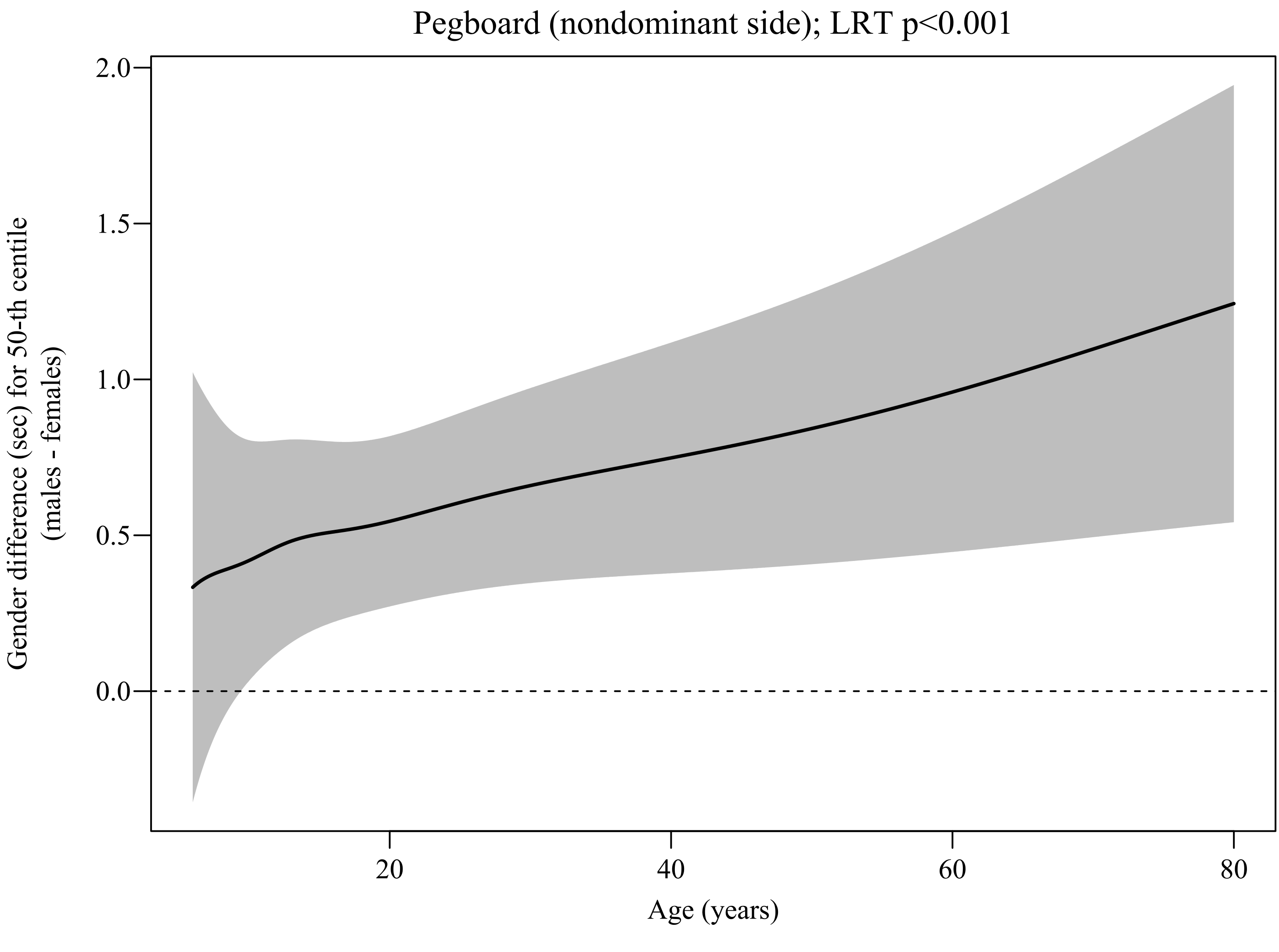

Pegboard (nondominant side); LRT p<0.001
Gender difference (sec) for 50-th centile
(males - females)
Age (years)
0.0
0.5
1.0
1.5
2.0
20
40
60
80

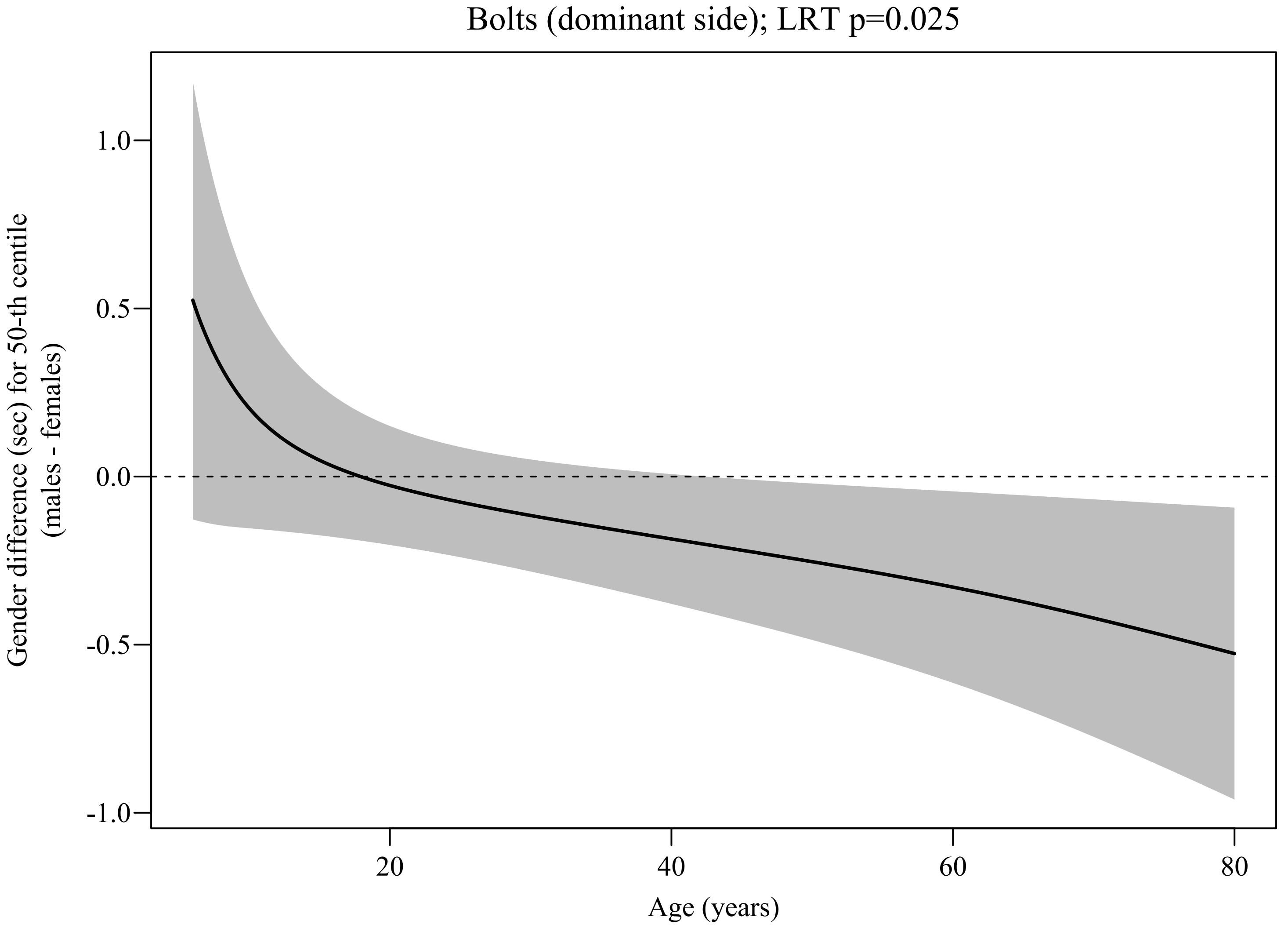

Bolts (dominant side); LRT p=0.025
Gender difference (sec) for 50-th centile
(males - females)
1.0
0.5
0.0
-0.5
-1.0
20
40
60
80
Age (years)

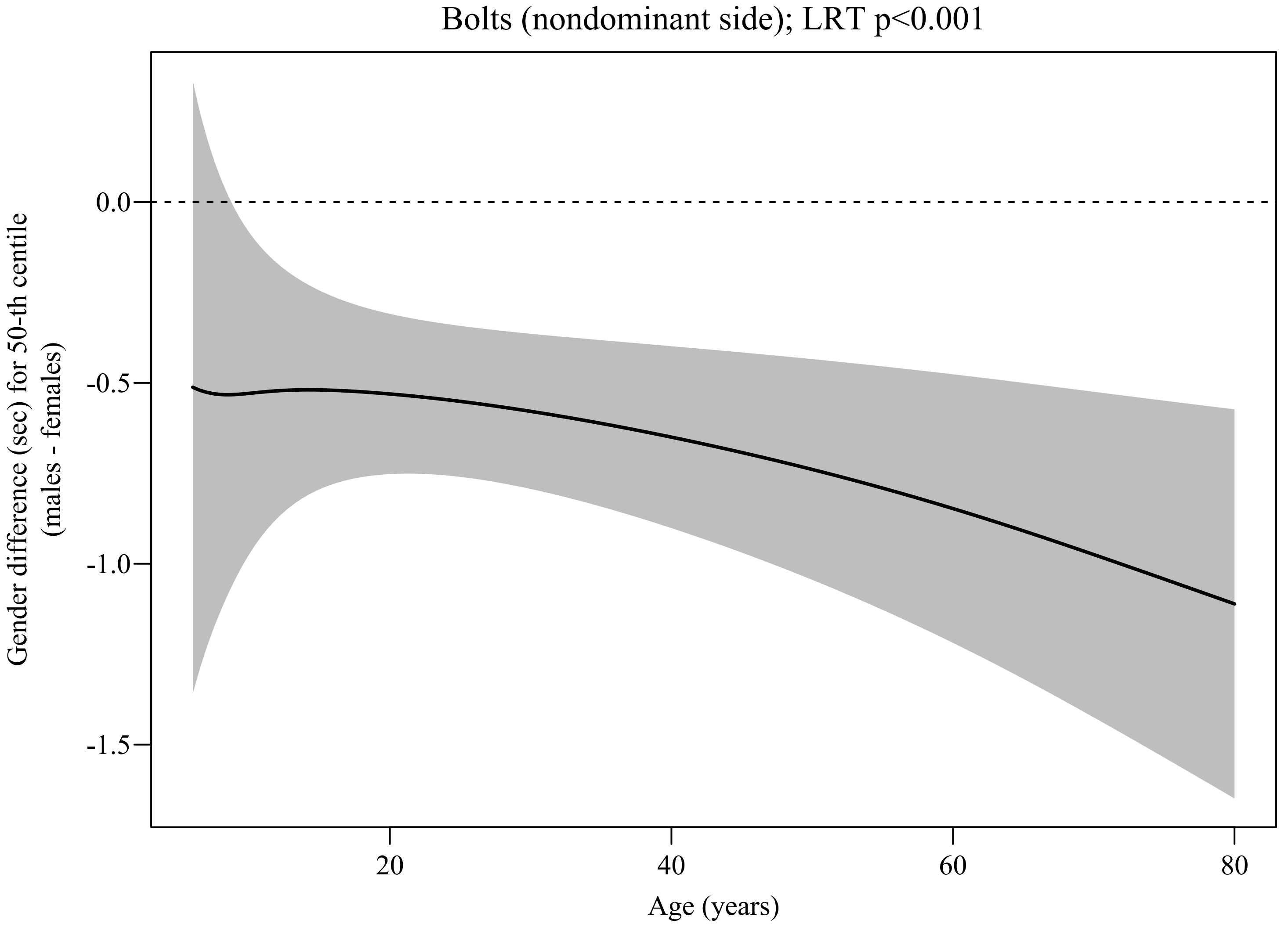

Bolts (nondominant side); LRT p<0.001
Gender difference (sec) for 50-th centile
(males - females)
0.0
-0.5
-1.0
-1.5
20
40
60
80
Age (years)

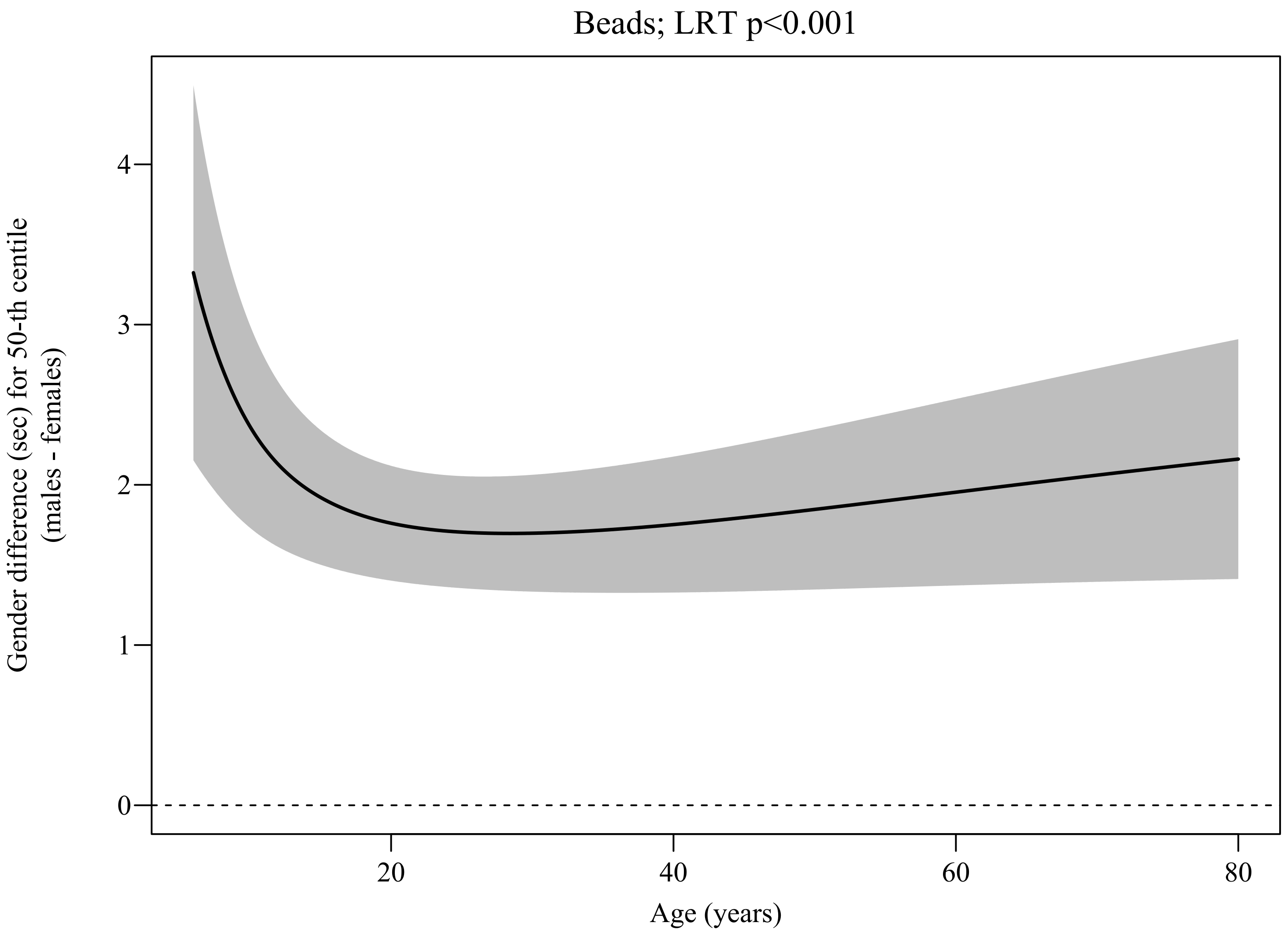
Beads; LRT p<0.001
Gender difference (sec) for 50-th centile
(males - females)
0
1
2
3
4
20
40
60
80
Age (years)

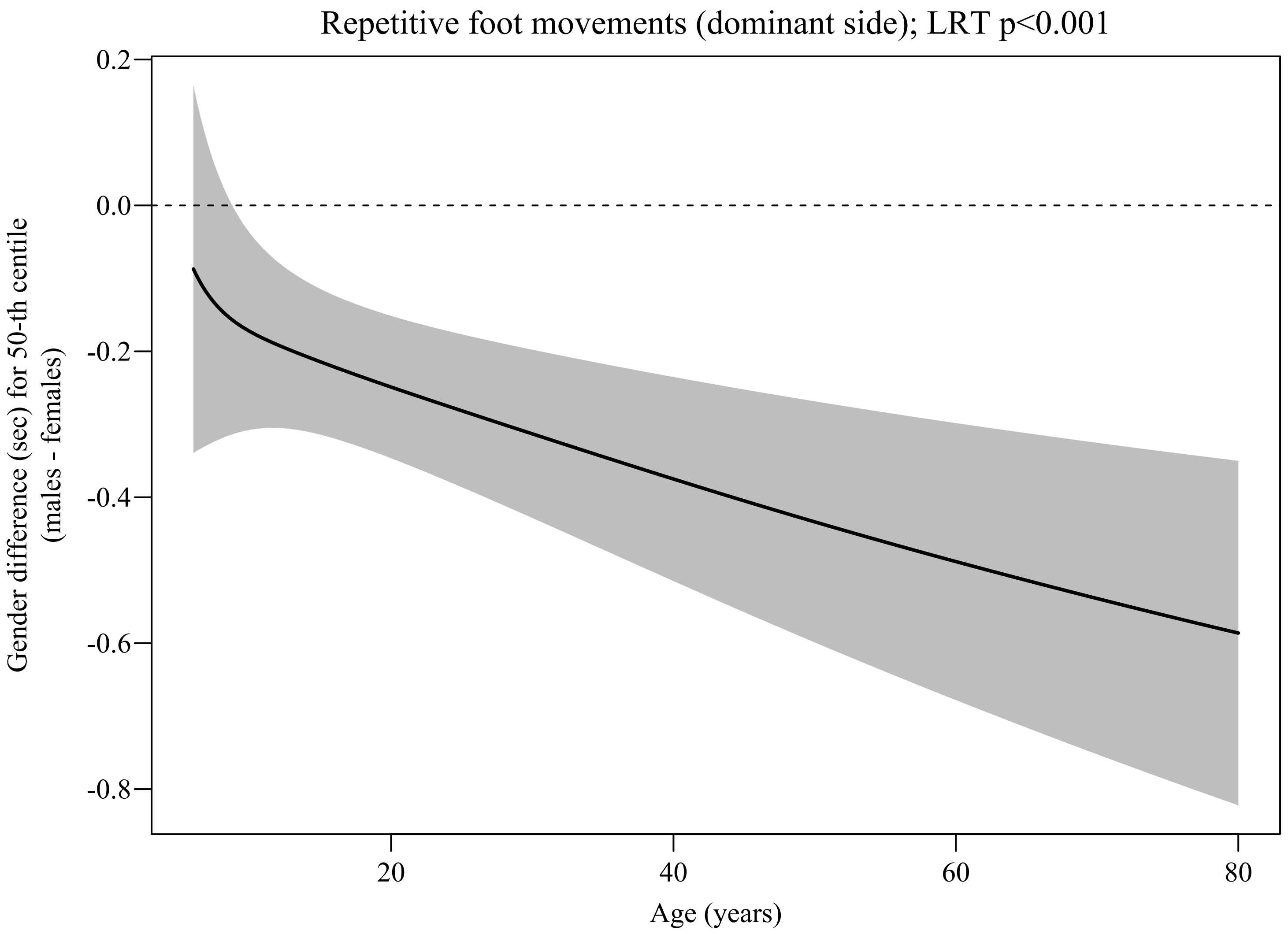
Repetitive foot movements (dominant side); LRT p<0.001
Gender difference (sec) for 50-th centile
(males - females)
0.2
0.0
-0.2
-0.4
-0.6
-0.8
20
40
60
80
Age (years)

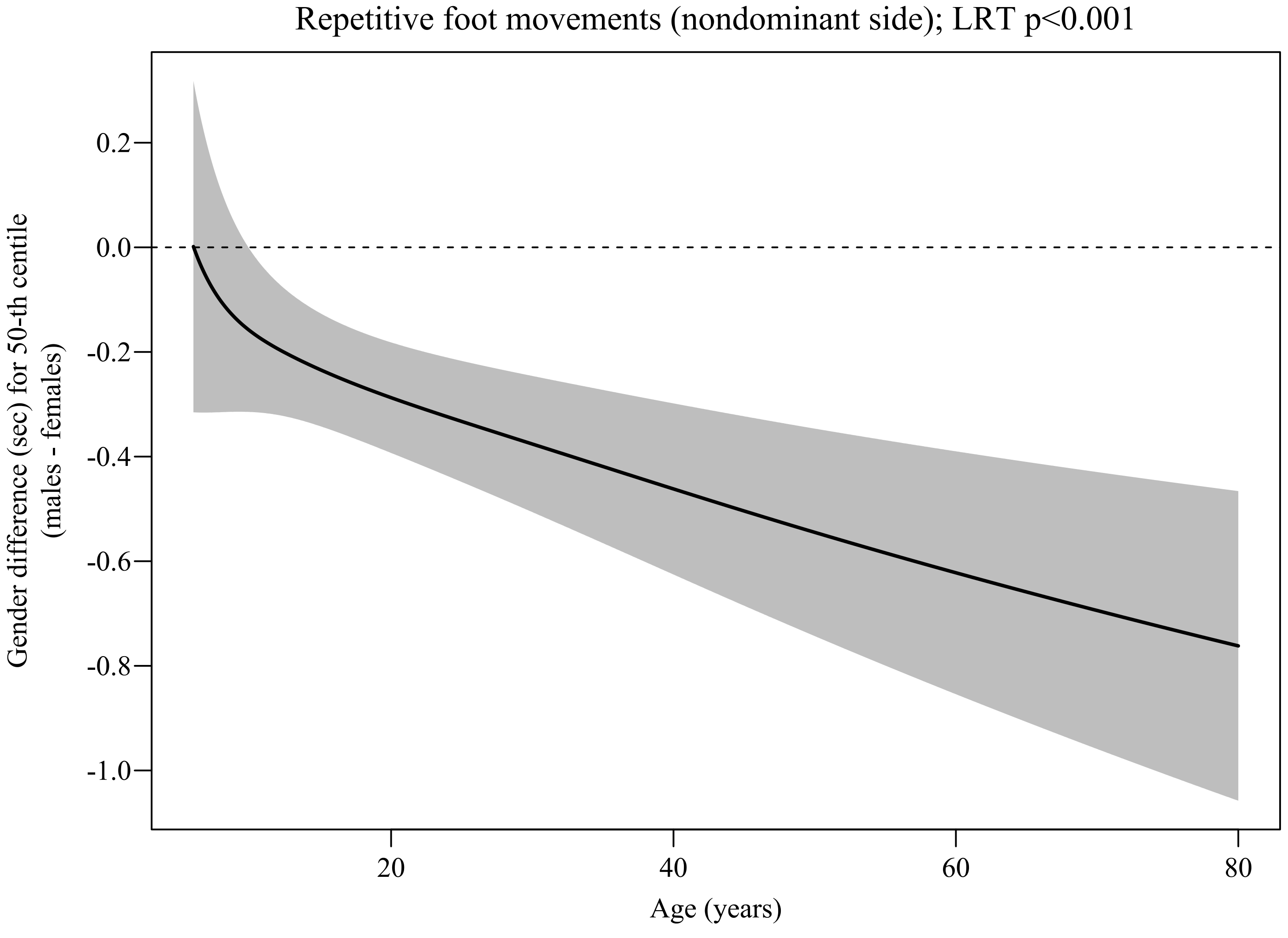
Repetitive foot movements (nondominant side); LRT p<0.001
Gender difference (sec) for 50-th centile
(males - females)
0.2
0.0
-0.2
-0.4
-0.6
-0.8
-1.0
20
40
60
80
Age (years)

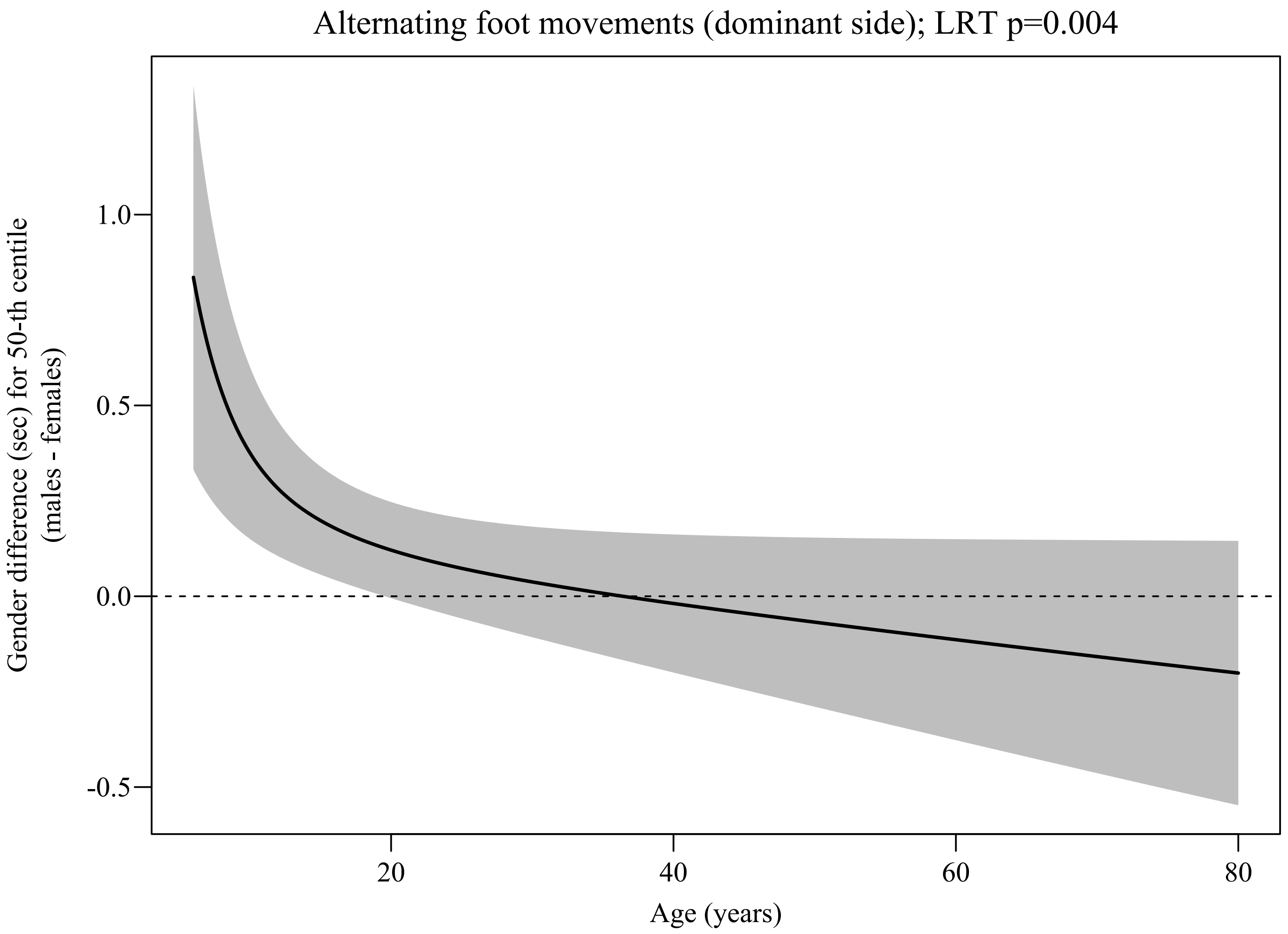

Alternating foot movements (dominant side); LRT p=0.004
Gender difference (sec) for 50-th centile
(males - females)
1.0
0.5
0.0
-0.5
20
40
60
80
Age (years)

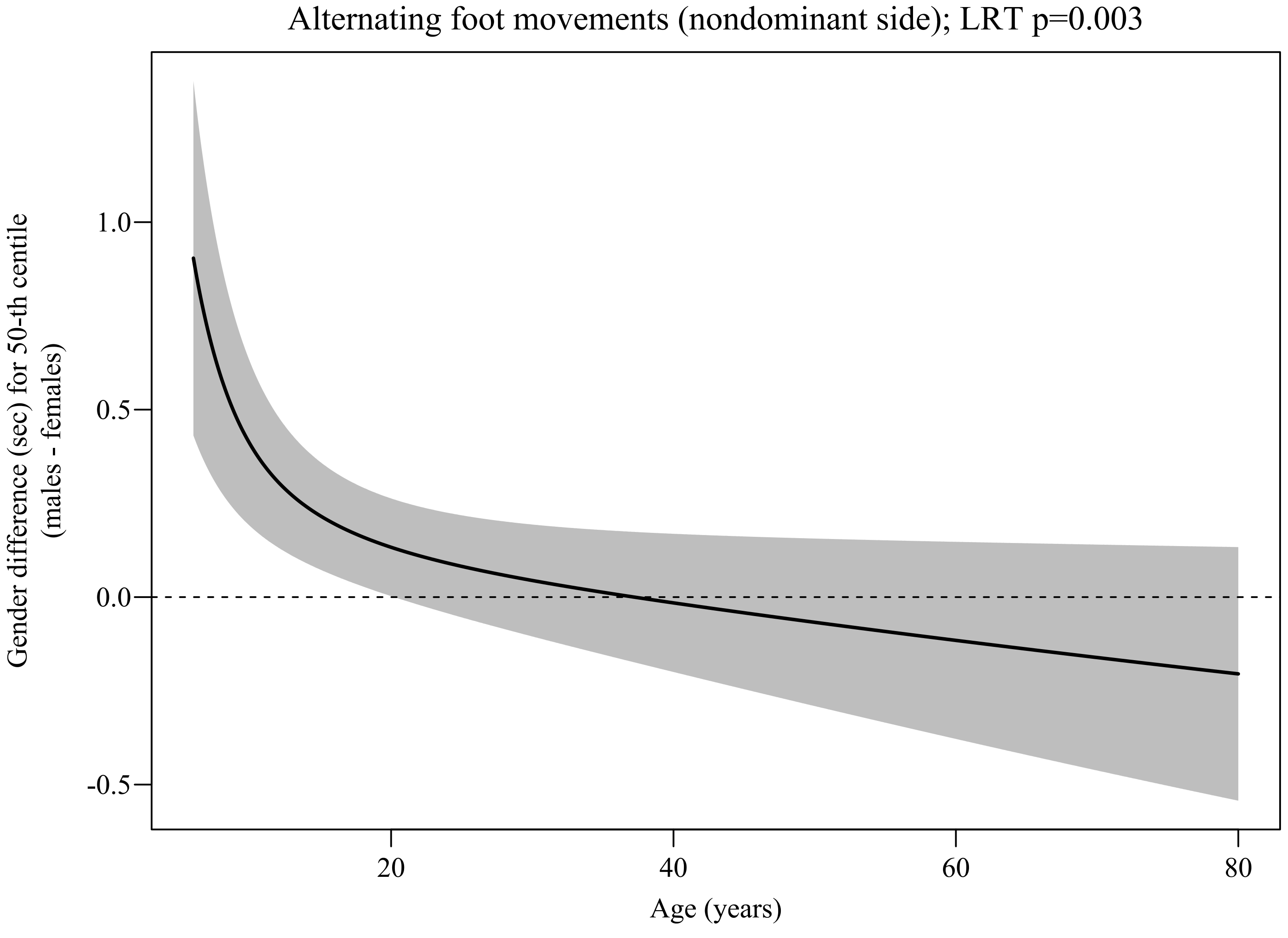

Alternating foot movements (nondominant side); LRT p=0.003
Gender difference (sec) for 50-th centile
(males - females)
1.0
0.5
0.0
-0.5
20
40
60
80
Age (years)

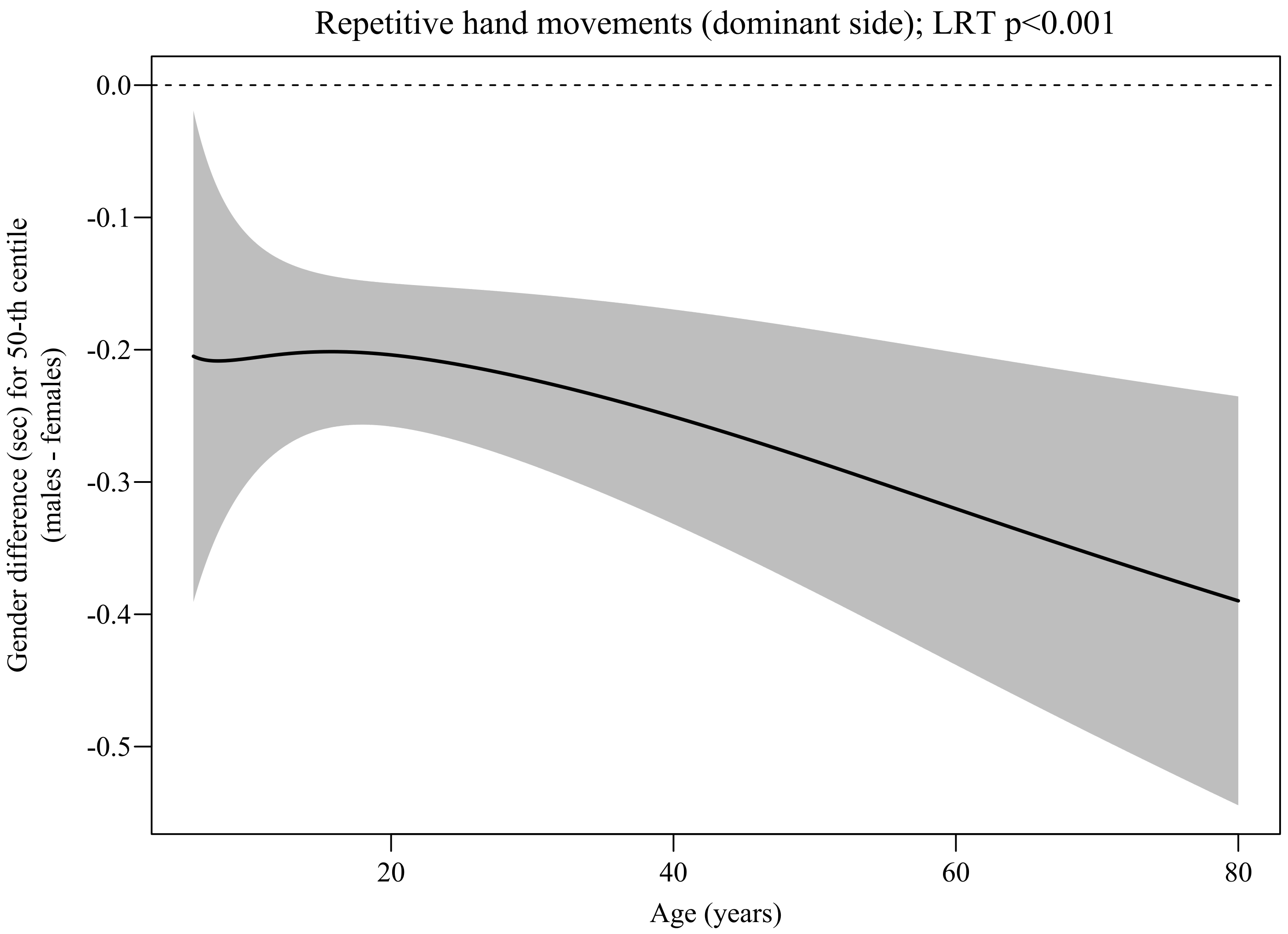
Repetitive hand movements (dominant side); LRT p<0.001
0.0
-0.1
-0.2
-0.3
-0.4
-0.5
Gender difference (sec) for 50-th centile
(males - females)
20
40
60
80
Age (years)

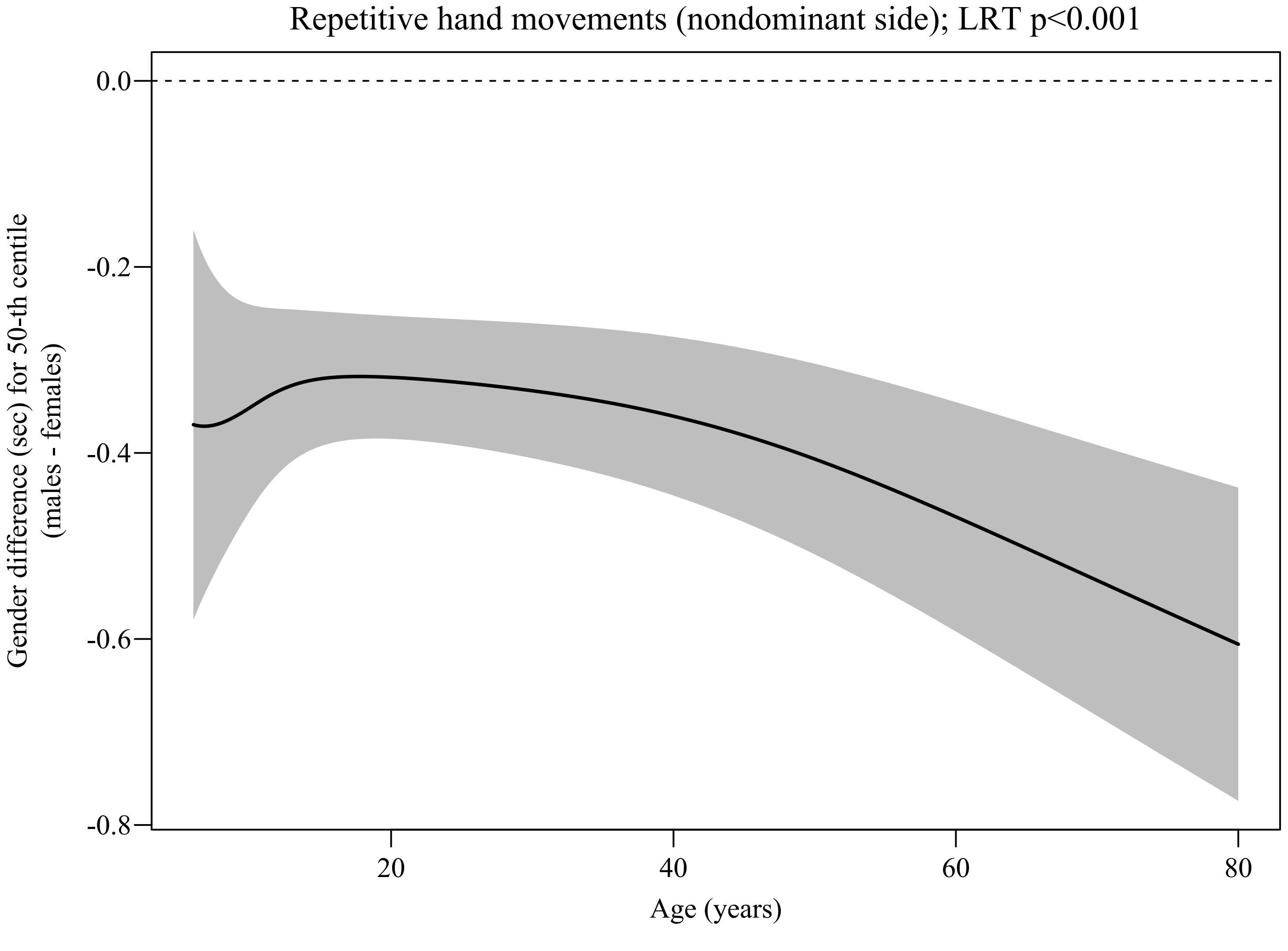
Repetitive hand movements (nondominant side); LRT p<0.001
0.0
-0.2
-0.4
-0.6
-0.8
Gender difference (sec) for 50-th centile
(males - females)
20
40
60
80
Age (years)

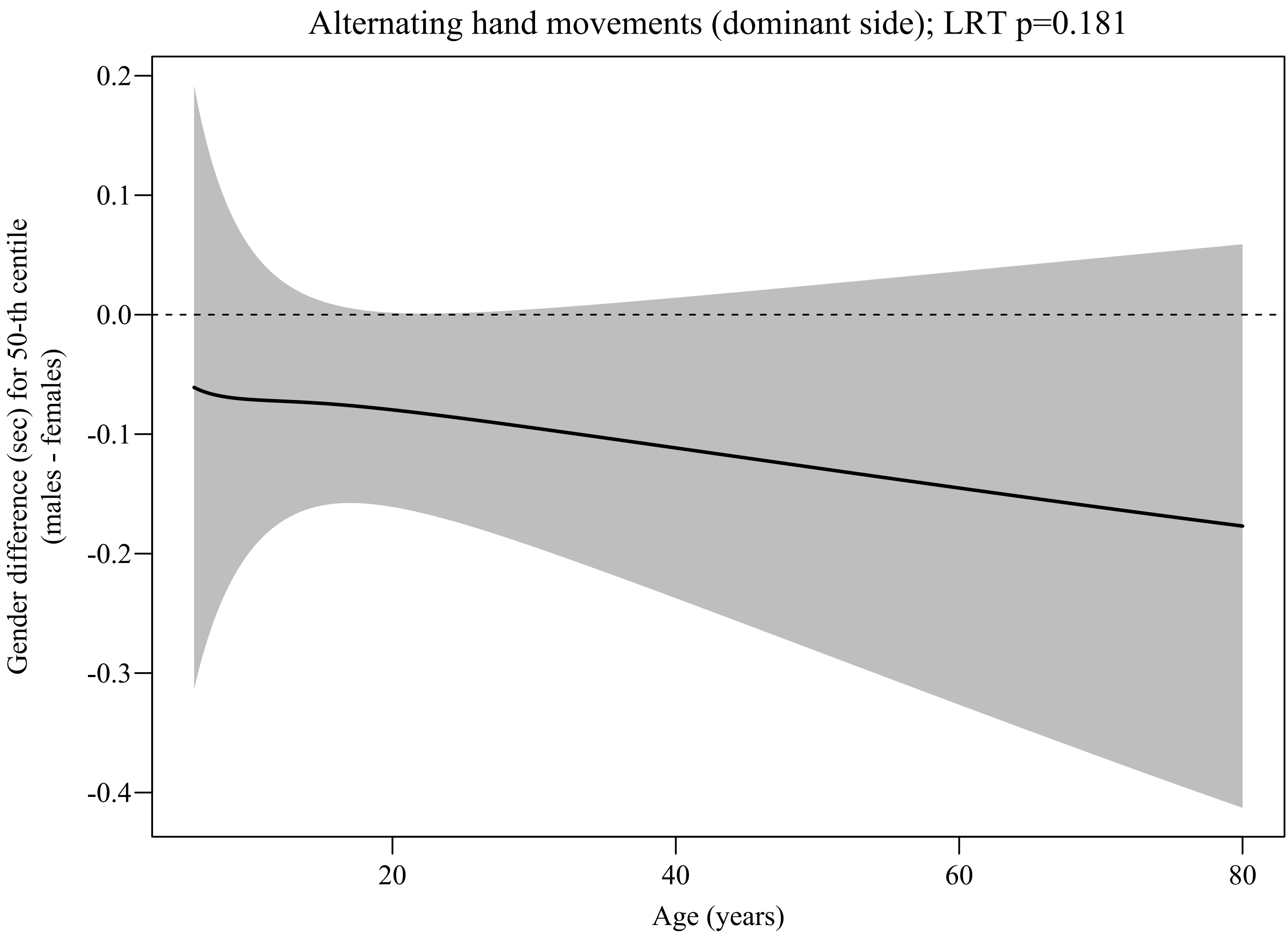

Alternating hand movements (dominant side); LRT p=0.181
Gender difference (sec) for 50-th centile
(males - females)
0.2
0.1
0.0
-0.1
-0.2
-0.3
-0.4
20
40
60
80
Age (years)

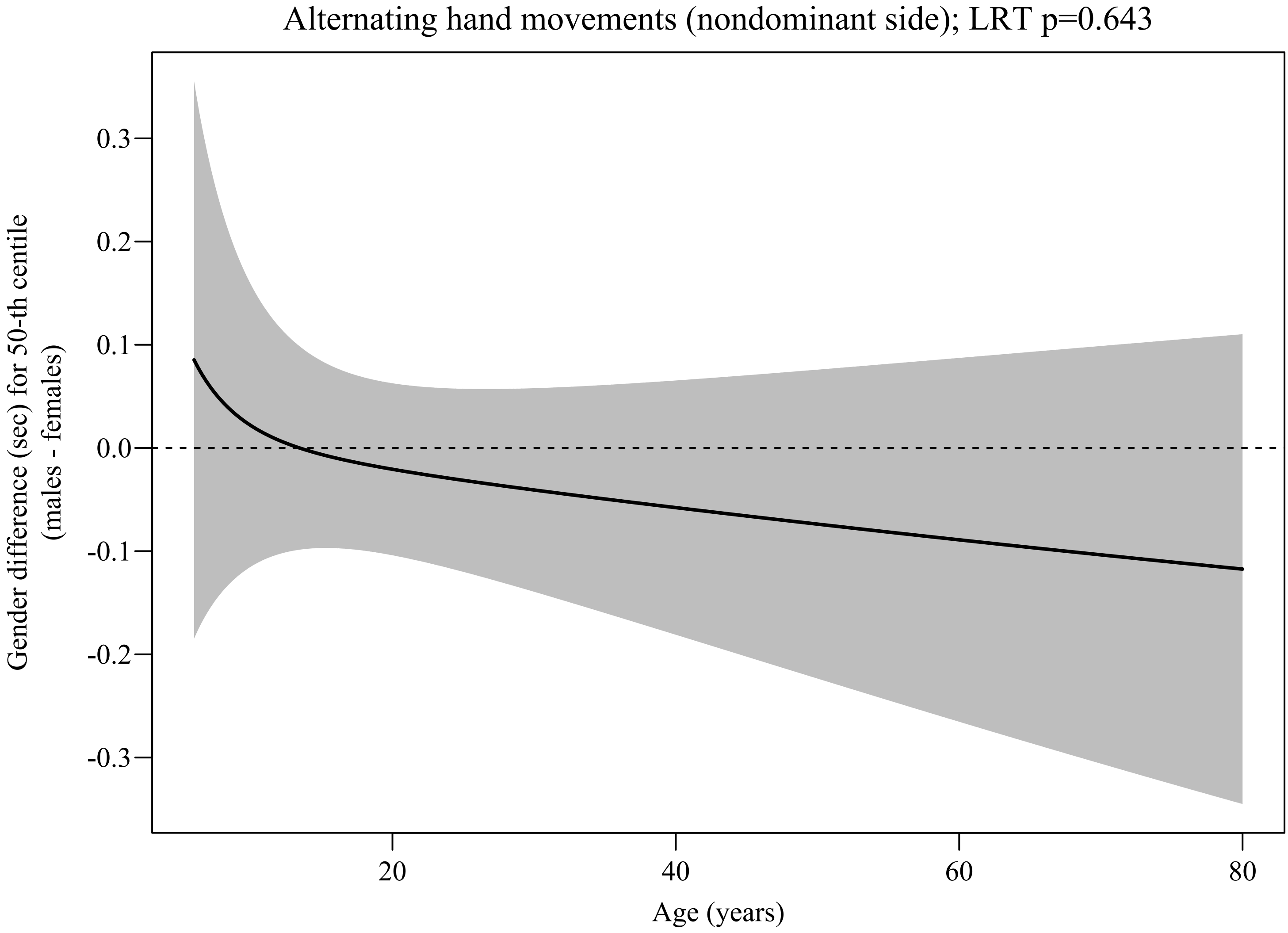

Alternating hand movements (nondominant side); LRT p=0.643
Gender difference (sec) for 50-th centile
(males - females)
0.3
0.2
0.1
0.0
-0.1
-0.2
-0.3
20
40
60
80
Age (years)

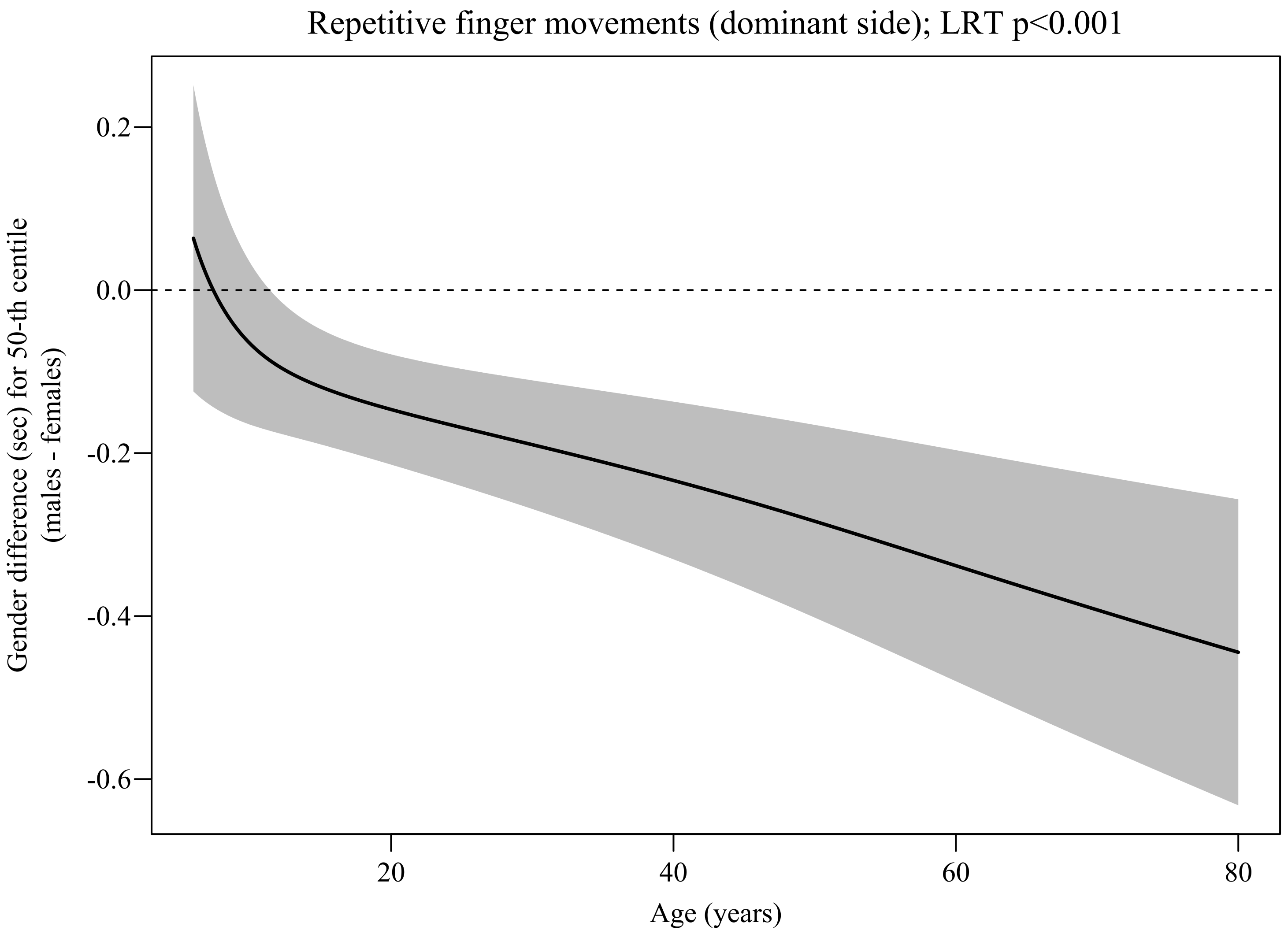
Repetitive finger movements (dominant side); LRT p<0.001
Gender difference (sec) for 50-th centile
(males - females)
0.2
0.0
-0.2
-0.4
-0.6
20
40
60
80
Age (years)

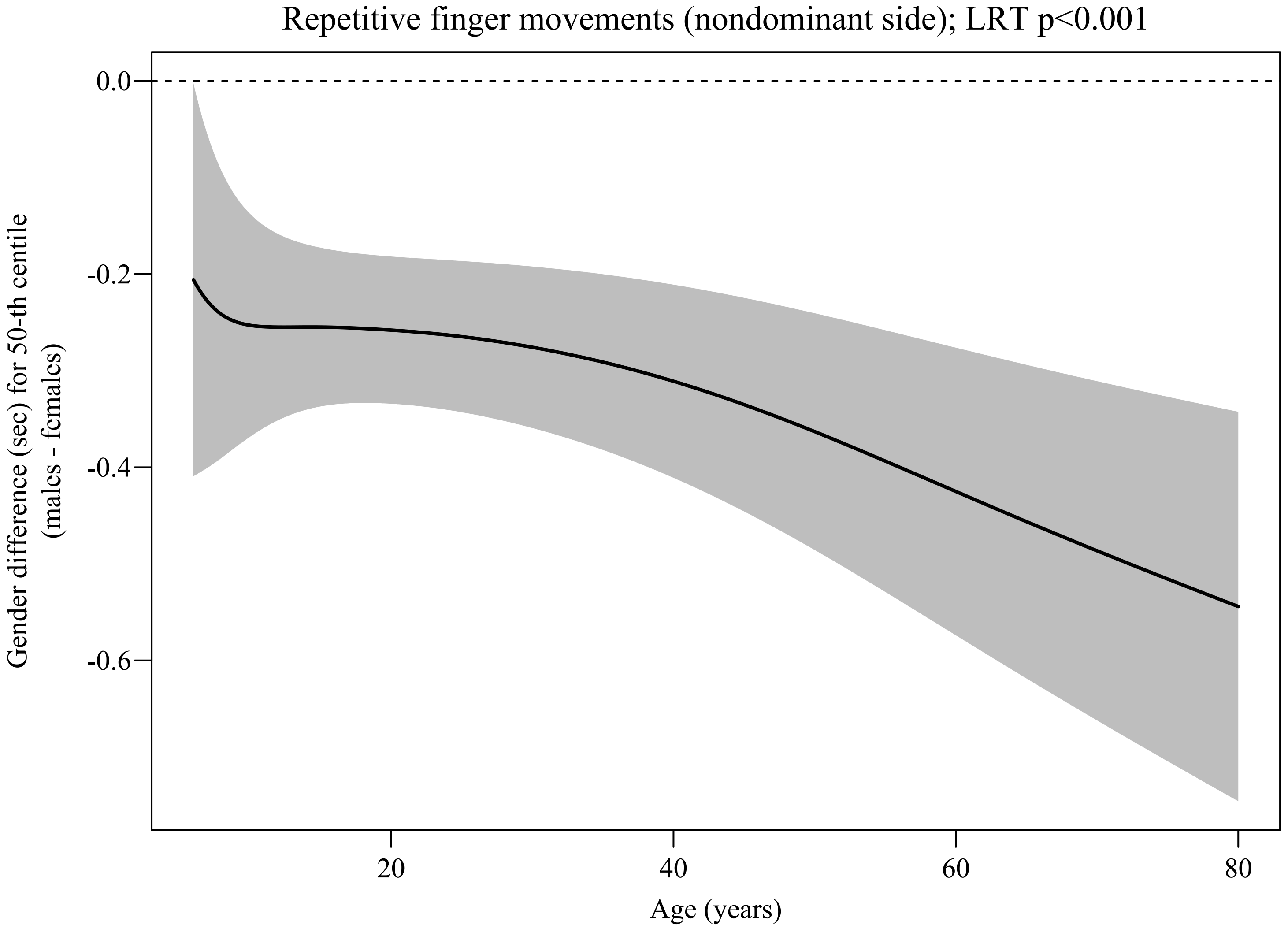
Repetitive finger movements (nondominant side); LRT p<0.001
Gender difference (sec) for 50-th centile
(males - females)
0.0
-0.2
-0.4
-0.6
20
40
60
80
Age (years)

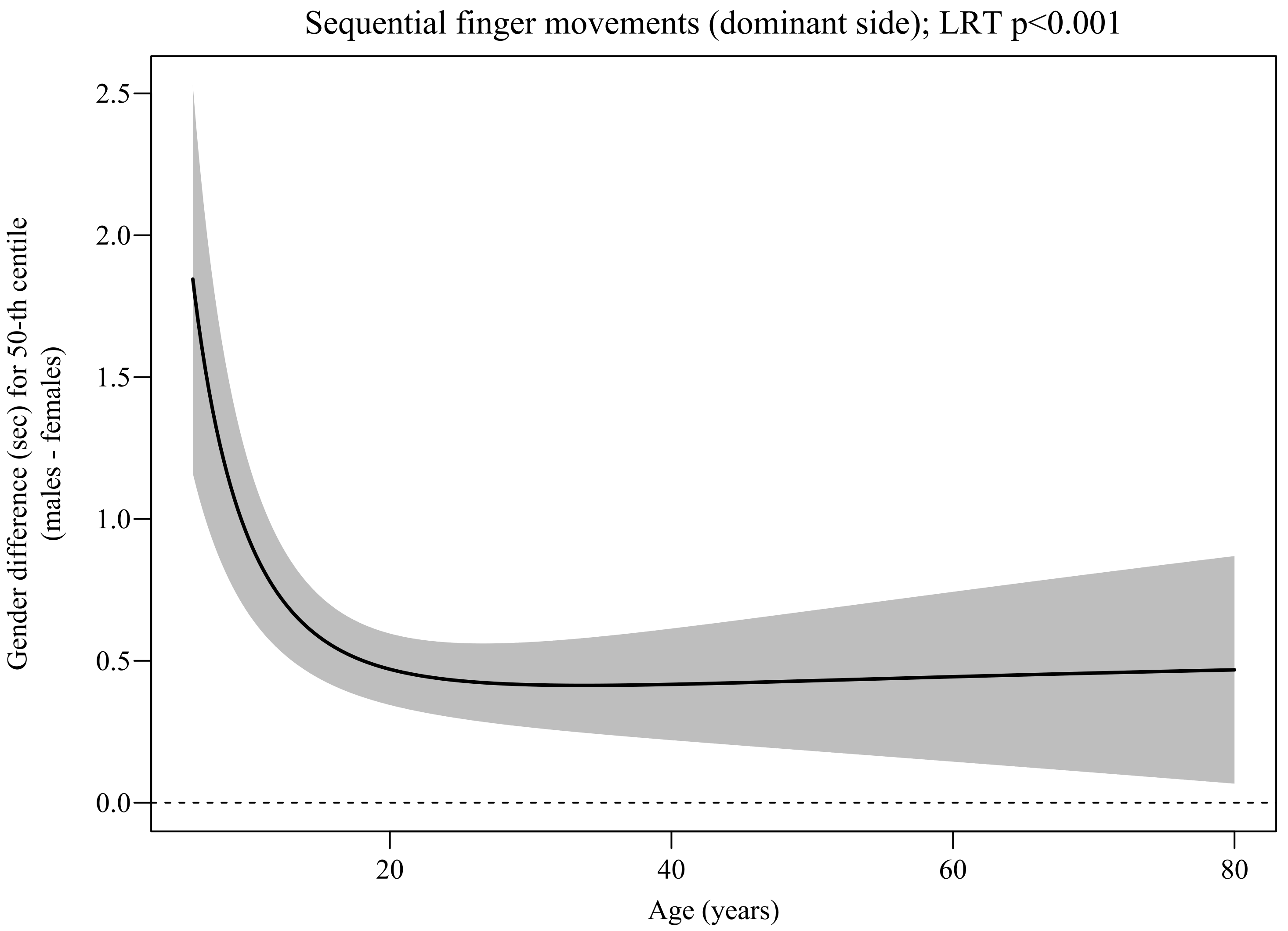
Sequential finger movements (dominant side); LRT p<0.001
Gender difference (sec) for 50-th centile
(males - females)
2.5
2.0
1.5
1.0
0.5
0.0
20
40
60
80
Age (years)

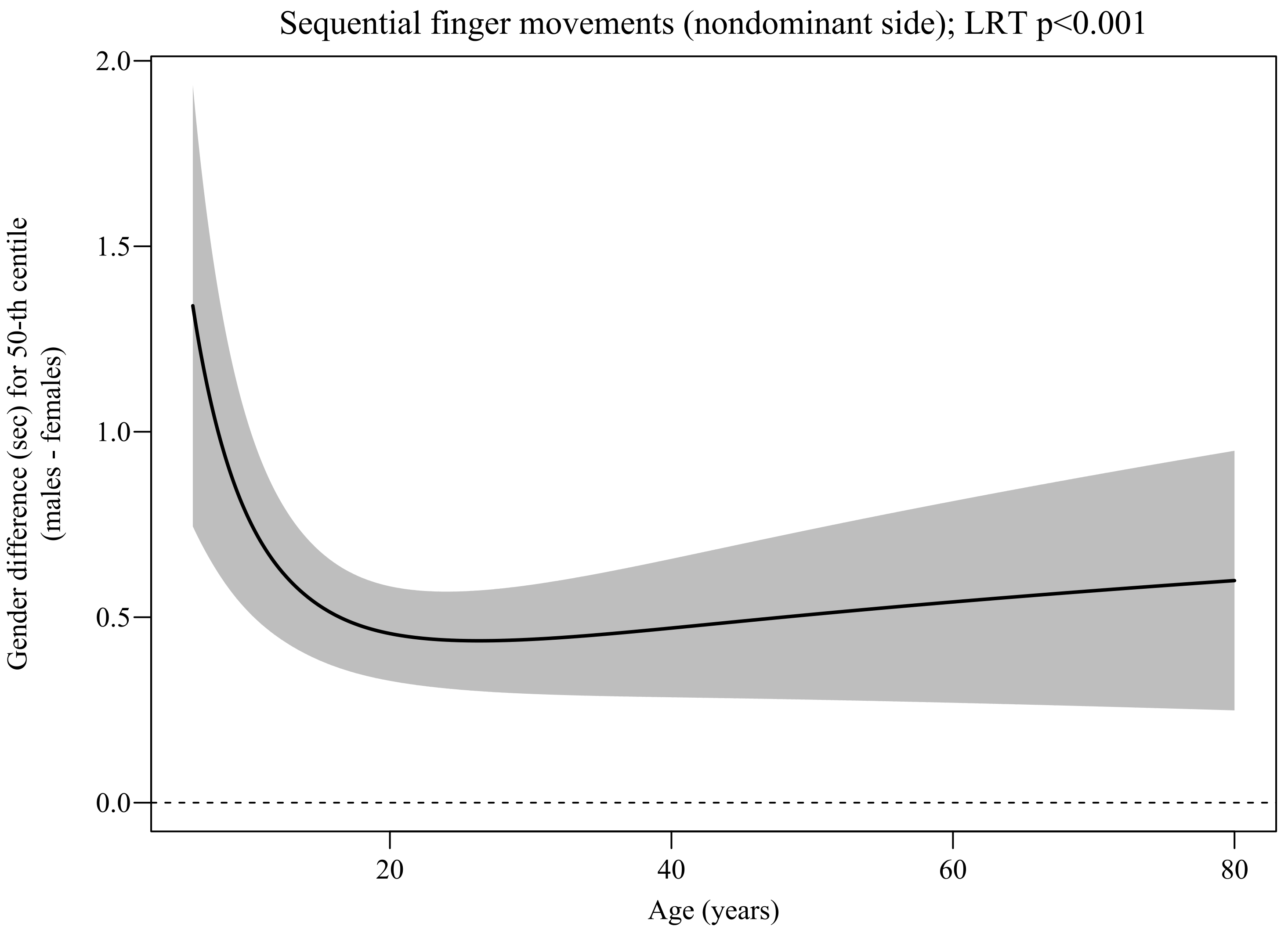
Sequential finger movements (nondominant side); LRT p<0.001
Gender difference (sec) for 50-th centile
(males - females)
2.0
1.5
1.0
0.5
0.0
20
40
60
80
Age (years)

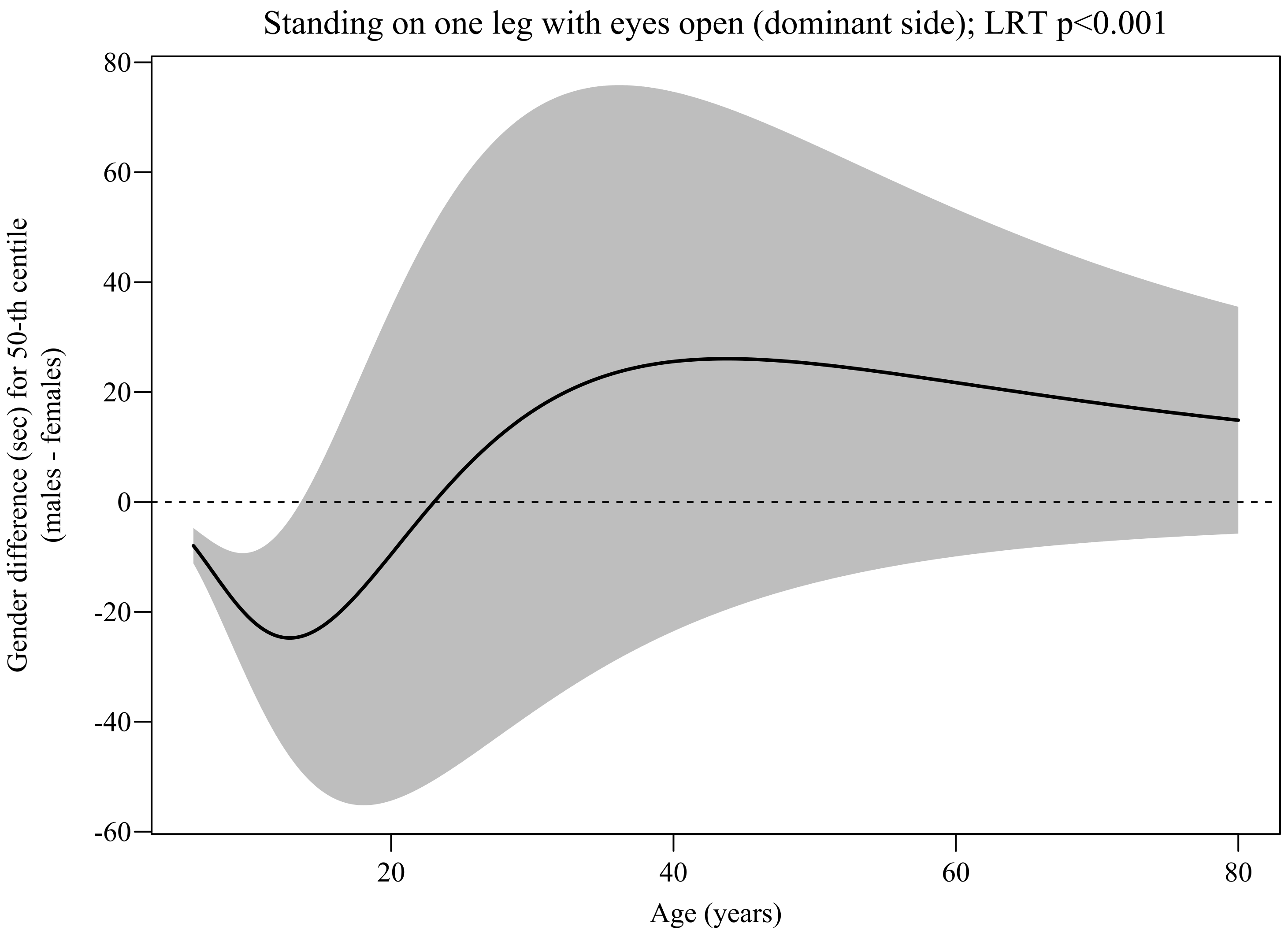
Standing on one leg with eyes open (dominant side); LRT p<0.001
Gender difference (sec) for 50-th centile
(males - females)
80
60
40
20
0
-20
-40
-60
20
40
60
80
Age (years)

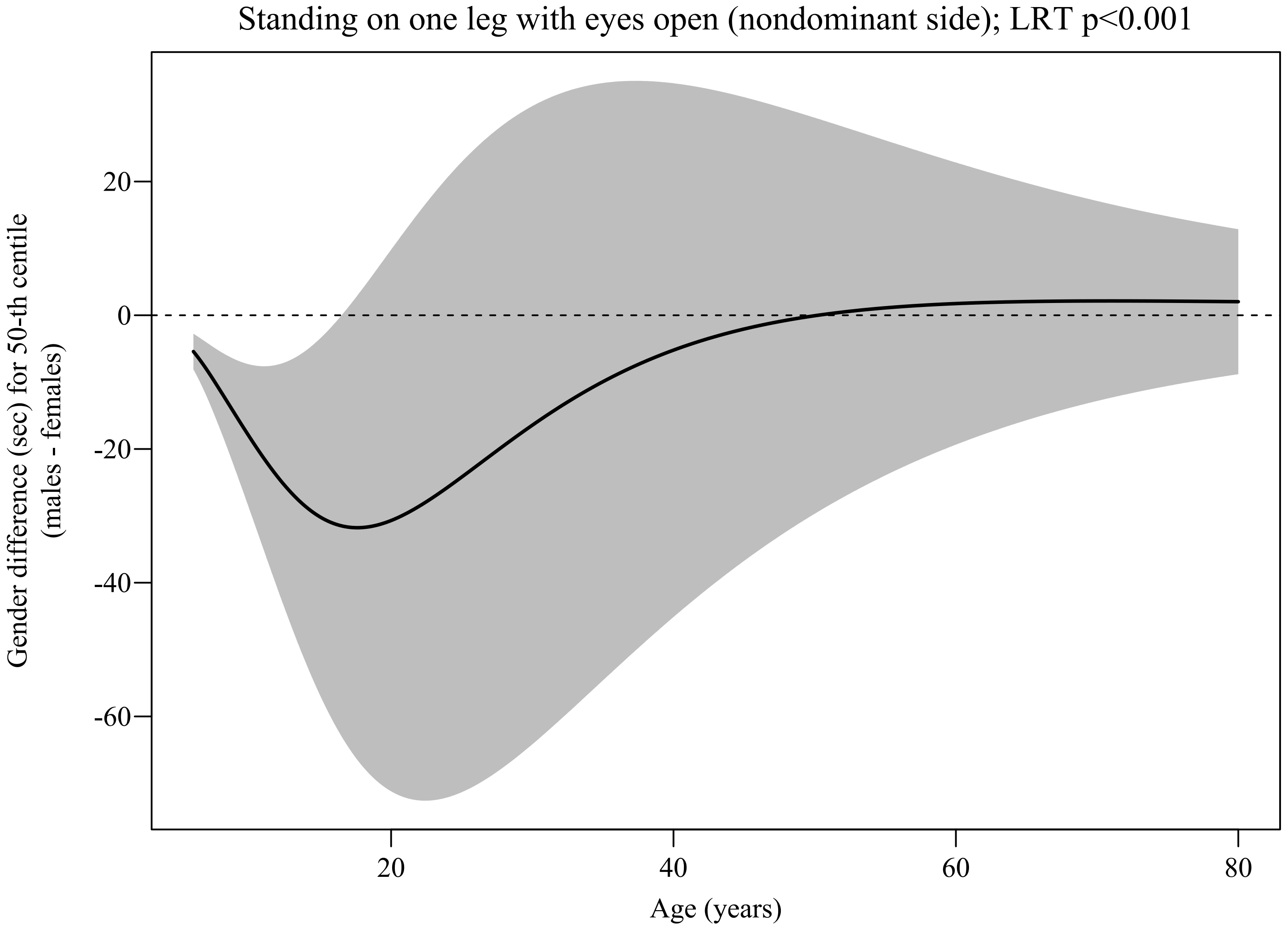
Standing on one leg with eyes open (nondominant side); LRT p<0.001
Gender difference (sec) for 50-th centile
(males - females)
20
0
-20
-40
-60
20
40
60
80
Age (years)

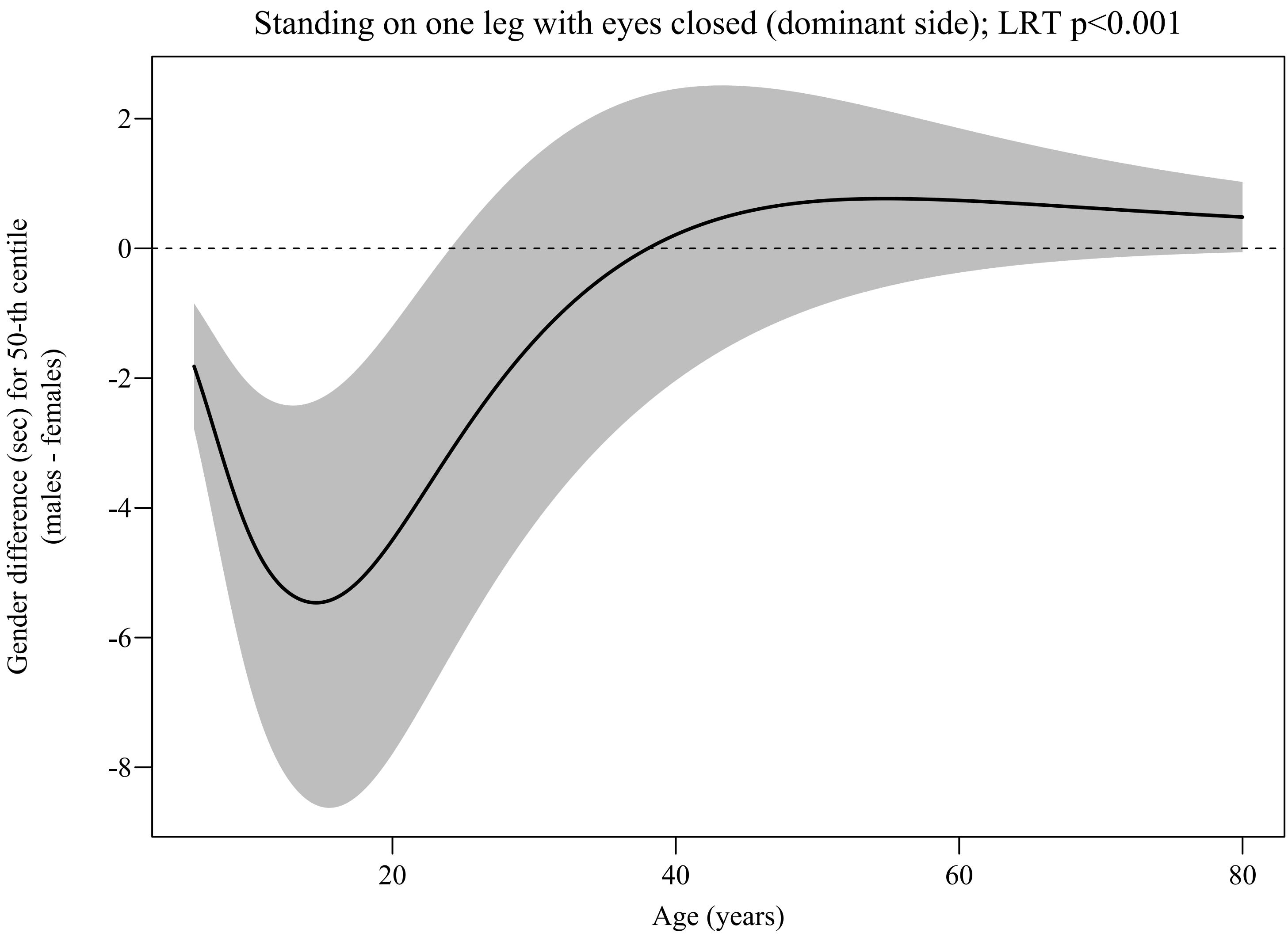

Standing on one leg with eyes closed (dominant side); LRT p<0.001
Gender difference (sec) for 50-th centile
(males - females)
2
0
-2
-4
-6
-8
20
40
60
80
Age (years)

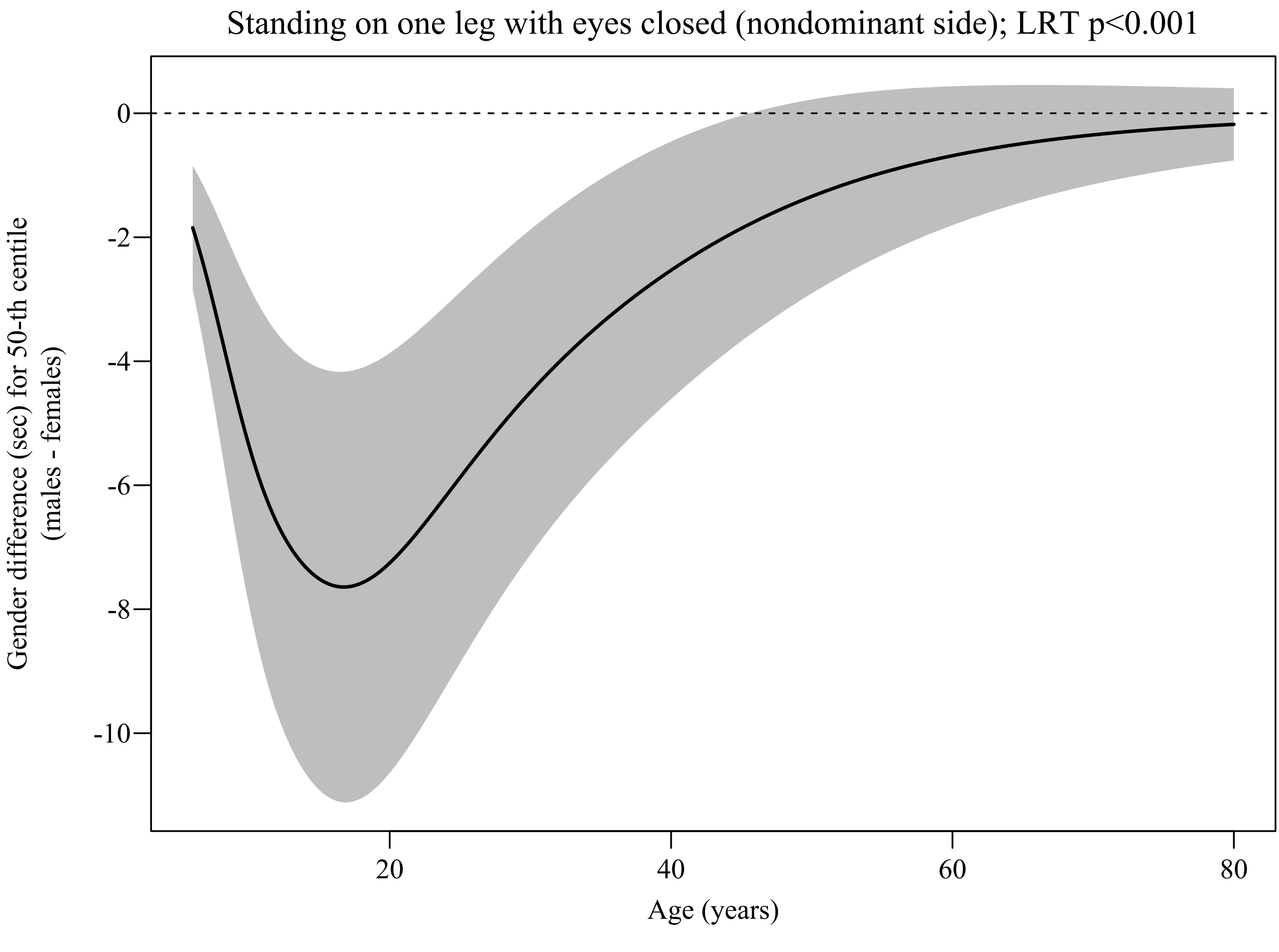

Standing on one leg with eyes closed (nondominant side); LRT p<0.001
Gender difference (sec) for 50-th centile
(males - females)
0
-2
-4
-6
-8
-10
20
40
60
80
Age (years)

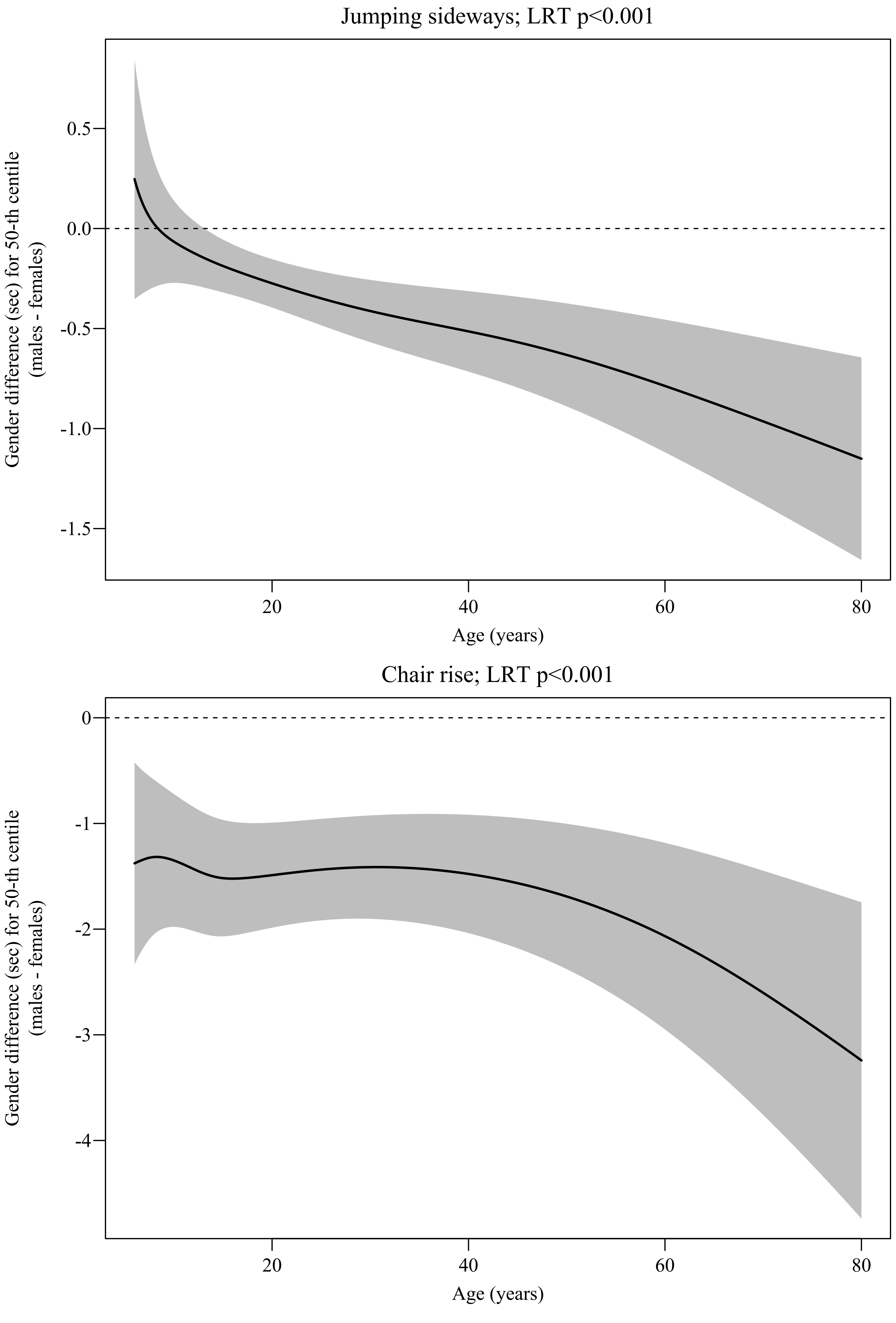
Jumping sideways; LRT p<0.001
Gender difference (sec) for 50-th centile
(males - females)
0.5
0.0
-0.5
-1.0
-1.5
20
40
60
80
Age (years)
Chair rise; LRT p<0.001
Gender difference (sec) for 50-th centile
(males - females)
0
-1
-2
-3
-4
20
40
60
80
Age (years)

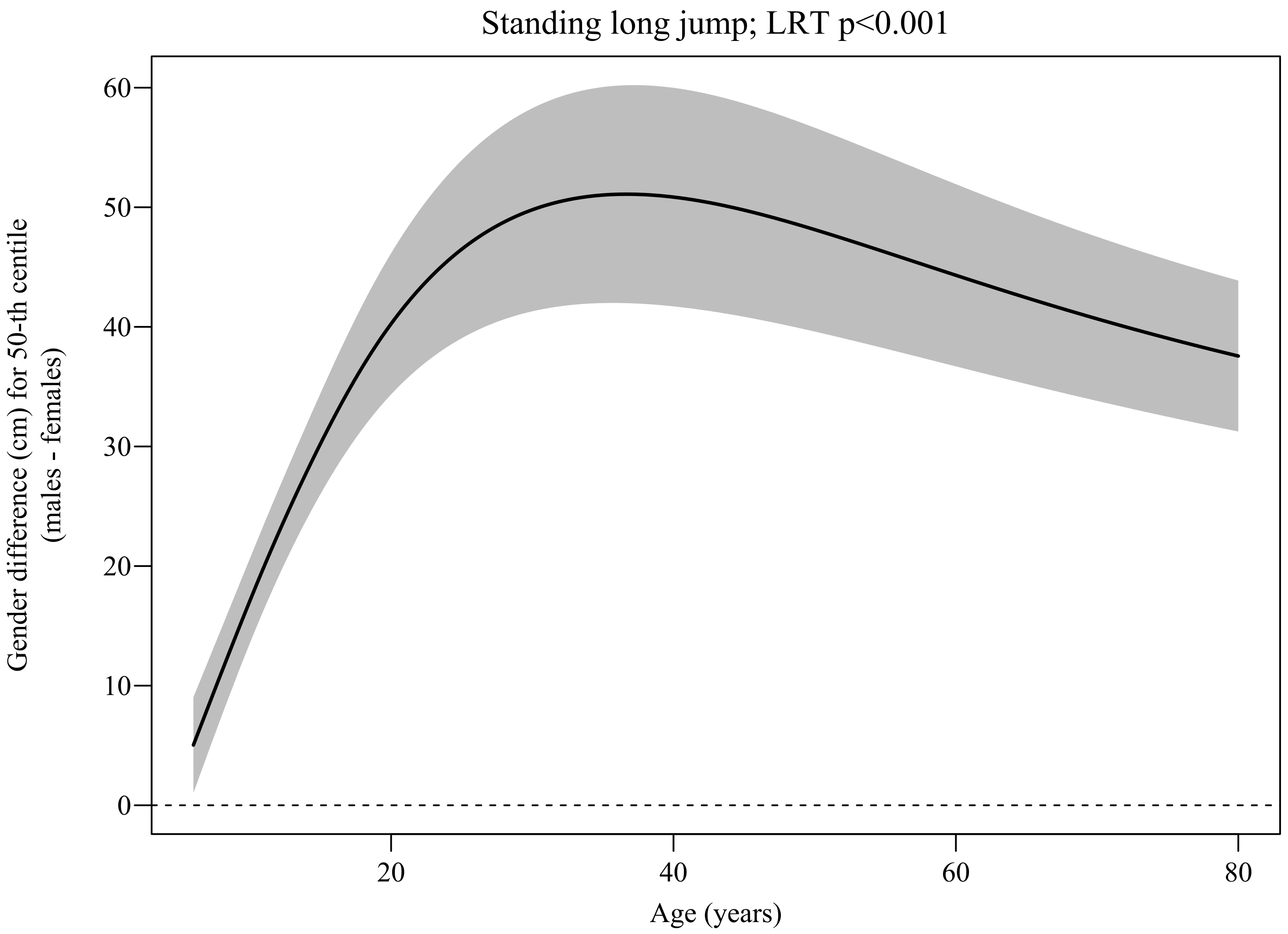

Standing long jump; LRT p<0.001
Gender difference (cm) for 50-th centile
(males - females)
60
50
40
30
20
10
0
20
40
60
80
Age (years)
