# Supplementary material for: Neuromotor functions across the lifespan: percentiles from 6 to 80 years
Source: Front Aging Neurosci. 2025 Jul 29;17:1543408. doi: 10.3389/fnagi.2025.1543408 (PMC12340781; doi:10.3389/fnagi.2025.1543408)
Supplement: Supplementary file 7 [file Data_Sheet_7.pdf]

Supplement e7: Sex differences for all 14 tasks across the lifespan, with age ranges during which one sex performs better than the other. X indicates that the corresponding sex performs best at all ages. *Reported age ranges indicate periods where the sex difference is not statistically significant, but without assessment of clinical relevance. Reported *P*-values refer to likelihood ratio tests comparing models with and without sex effects; y: years; d: dominant side; nd: nondominant side

ZNA-2			PERFORMANCE		
**COMPONENTS**	**TASKS**		**Females better**	**No significant sex difference***	**Males better**
**FINE MOTOR (FM)**	**Pegboard**	d	**X**		
		nd	**X**		
	**Bolts**	d		**<40y**	**>40y**
		nd			**X**
	**Beads**		**X**		
**PURE MOTOR (PM)**	**Repetitive Foot**	d			**X**
		nd			**X**
	**Repetitive Hand**	d			**X**
		nd			**X**
	**Repetitive Finger**	d			**X**
		nd			**X**
	**Alternating Foot**	d	**<20y**	**>20y**	
		nd	**<20y**	**>20y**	
	**Alternating Hand**	d		**X** (P=0.181)	
		nd		**X** (P=0.643)	
	**Sequential Finger**	d	**X**		
		nd	**X**		
**BALANCE (BA)**	**Static Balance eyes open**	d	**<15y**	**>15y**	
		nd	**<15y**	**>15y**	
	**Static Balance eyes closed**	d	**<25y**	**>25y**	
		nd	**<45y**	**>45y**	
**GROSS MOTOR (GM)**	**Jumping Sideways**			**<15y**	**>15y**
	**Chair Rise**				**X**
	**Standing Long Jump**				**X**
**CONTRALATERAL ASSOCIATED MOVEMENTS (CAM)**	**Pegboard**	d	**<35y**	**>35y**	
		nd	**<40y**	**>40y**	
	**Bolts**	d		**X** (P=0.215)	
		nd		**X** (P=0.325)	
	**Alternating Foot**	d	**<10y**	**10 - 55y**	**>55y**
		nd	**<10y**	**10 - 40y**	**>40y**
	**Alternating Hand**	d	**<25y**	**>25y**	
		nd	**<25y**	**>25y**	
	**Sequential Finger**	d	**X**		
		nd		**X** (P=0.335)	
