# Supplementary material for: Neuromotor functions across the lifespan: percentiles from 6 to 80 years
Source: Front Aging Neurosci. 2025 Jul 29;17:1543408. doi: 10.3389/fnagi.2025.1543408 (PMC12340781; doi:10.3389/fnagi.2025.1543408)
Supplement: Supplementary file 8 [file Data_Sheet_8.pdf]

Supplement e8: First derivative of the 50th centile curve (defined as the change over 2 months) with 95% confidence interval, for timed performance and standing long jump, separately for males and females. Peak performance is identified when the curve crosses the zero line.

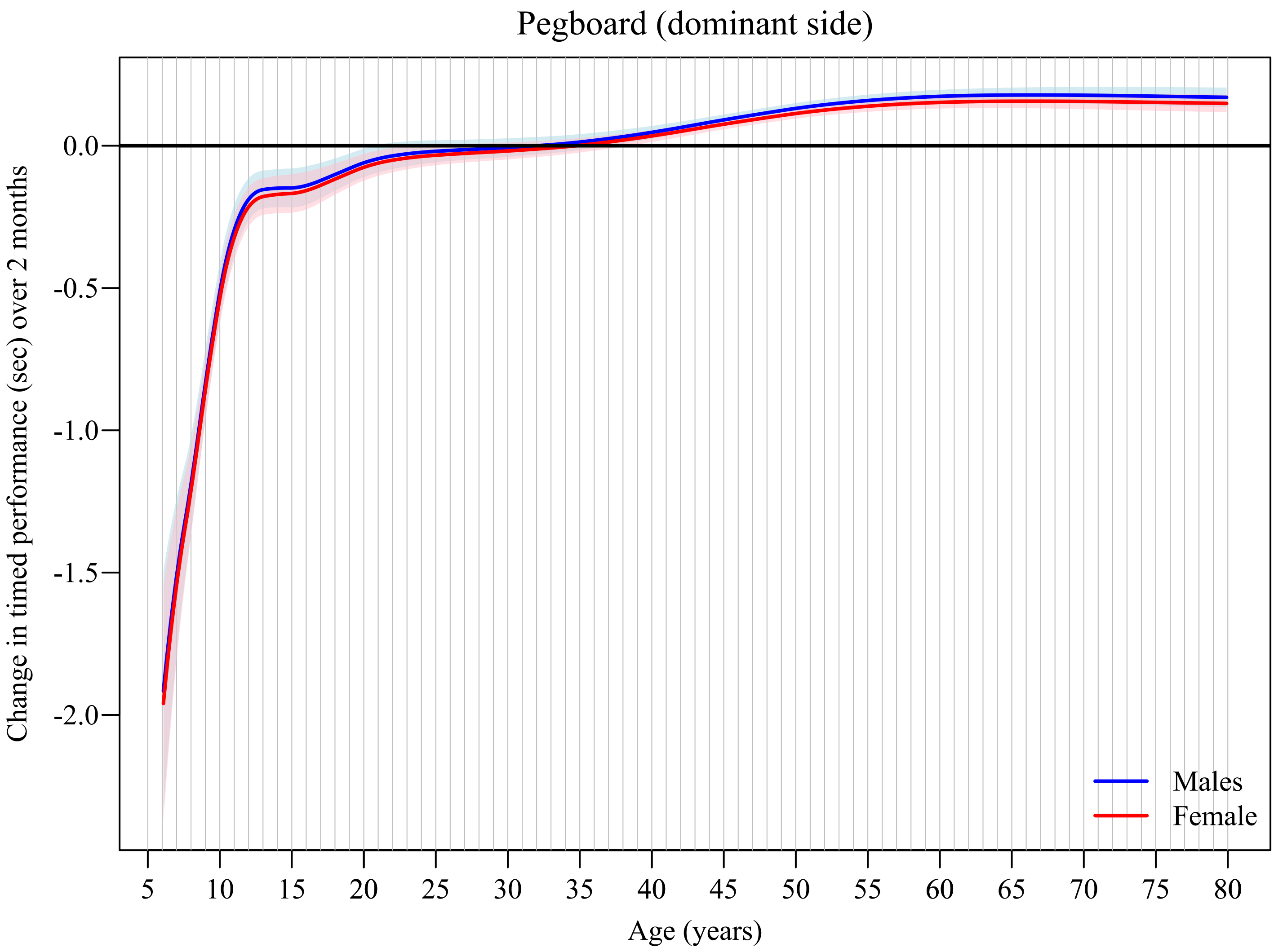
Pegboard (dominant side)
Change in timed performance (sec) over 2 months
0.0
-0.5
-1.0
-1.5
-2.0
5 10 15 20 25 30 35 40 45 50 55 60 65 70 75 80
Age (years)
Males
Female

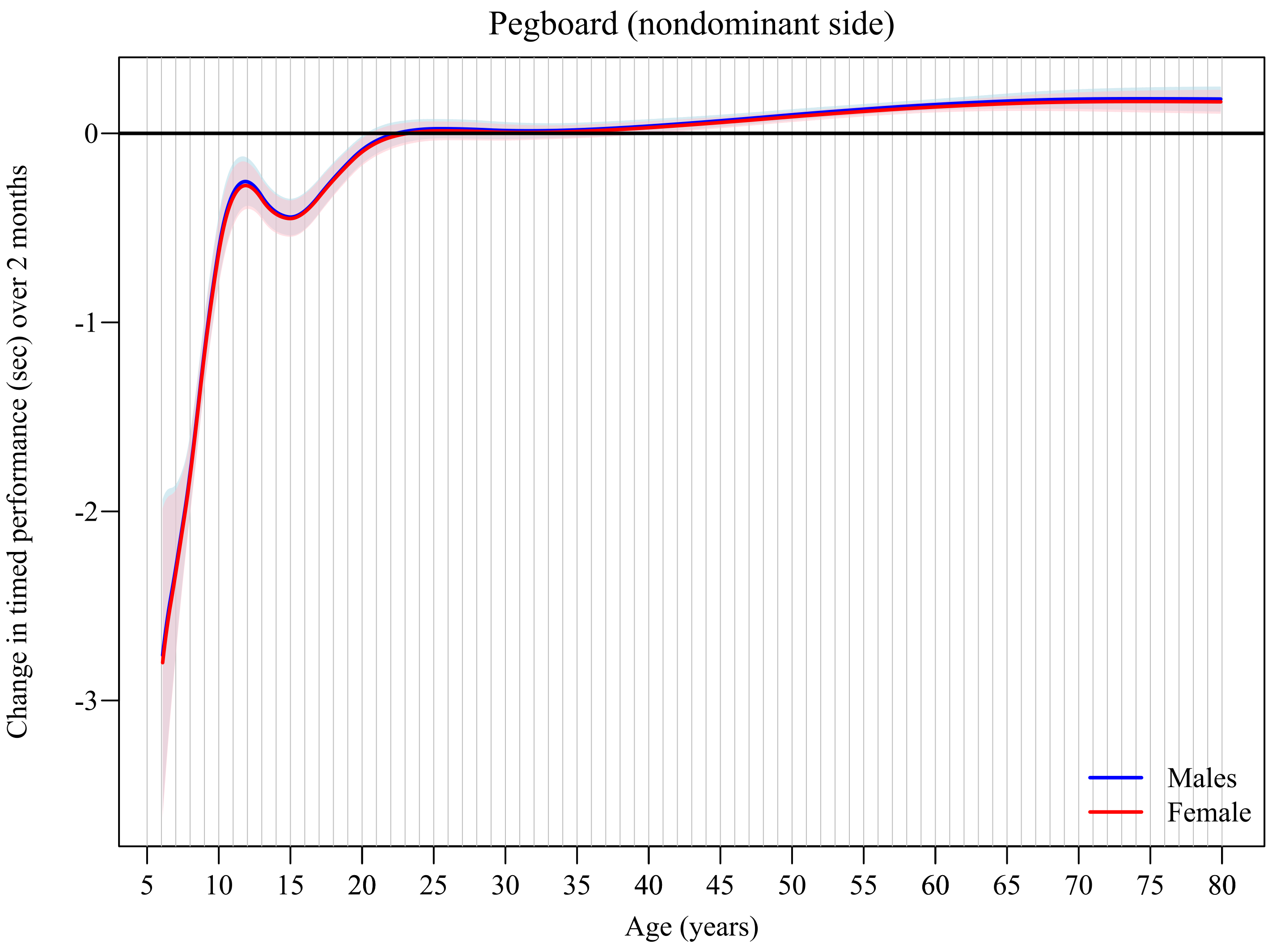
Pegboard (nondominant side)
Change in timed performance (sec) over 2 months
0
-1
-2
-3
5 10 15 20 25 30 35 40 45 50 55 60 65 70 75 80
Age (years)
Males
Female

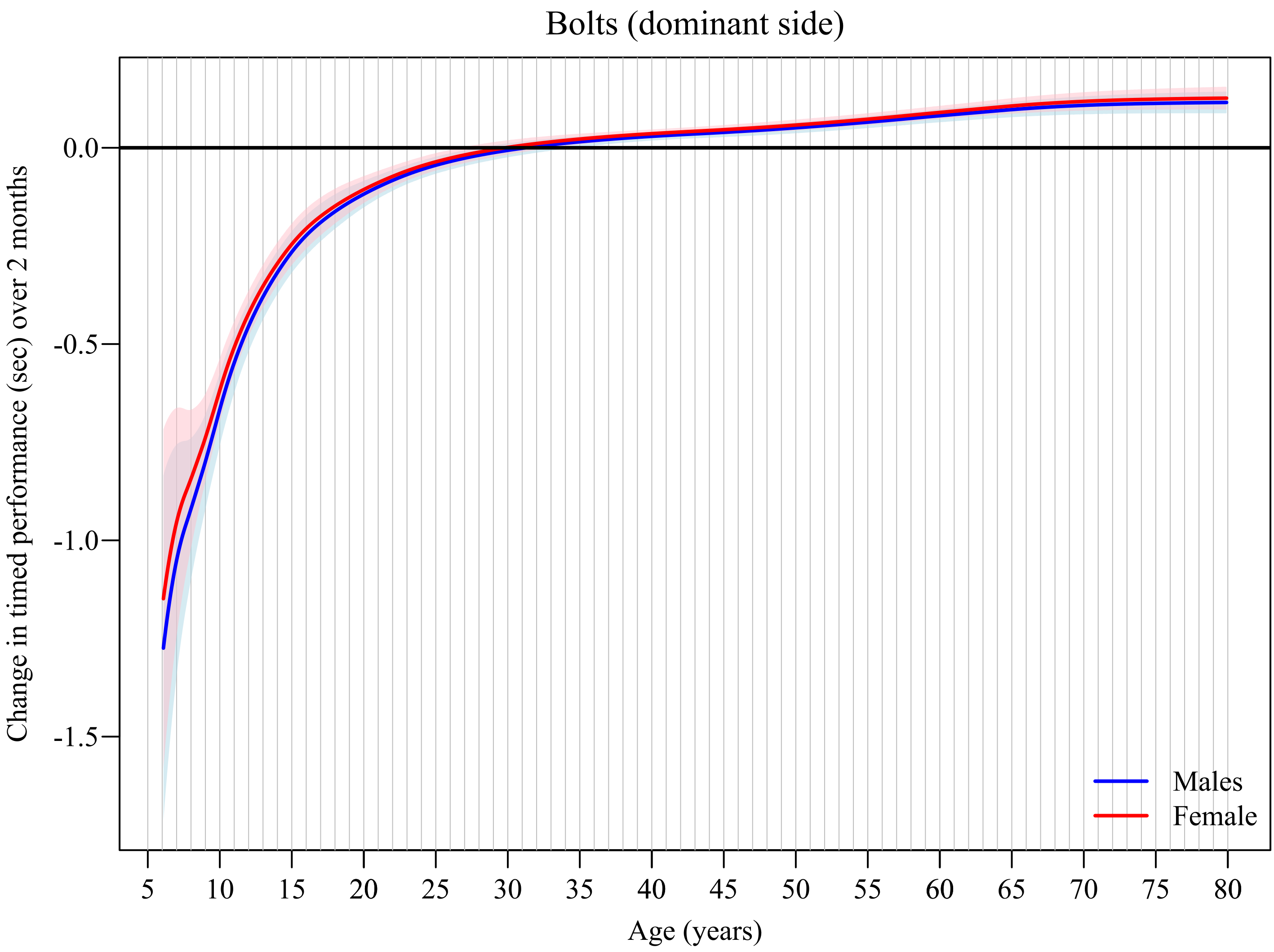
Bolts (dominant side)
Change in timed performance (sec) over 2 months
0.0
-0.5
-1.0
-1.5
5
10
15
20
25
30
35
40
45
50
55
60
65
70
75
80
Age (years)
Males
Female

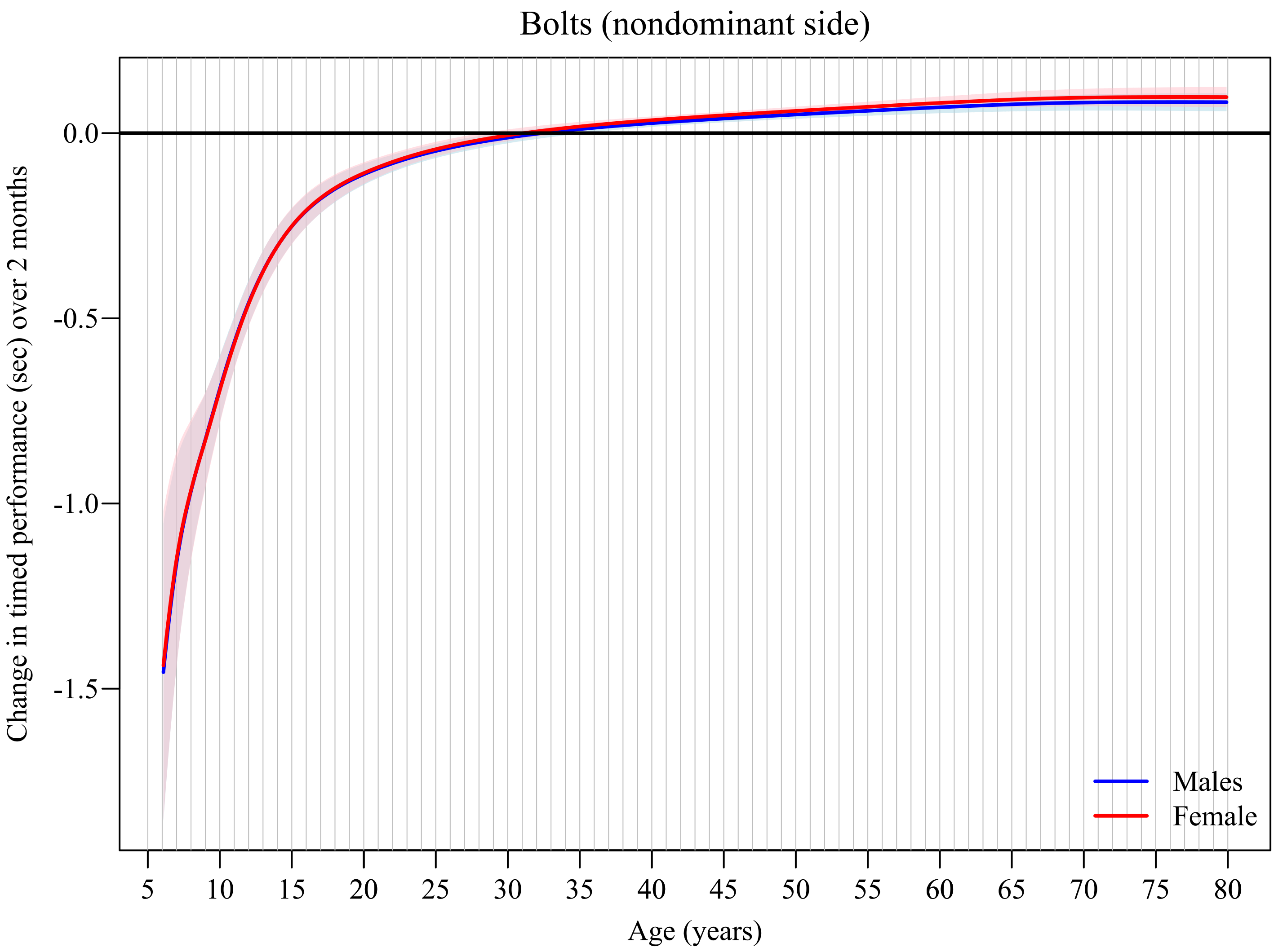
Bolts (nondominant side)
Change in timed performance (sec) over 2 months
0.0
-0.5
-1.0
-1.5
5
10
15
20
25
30
35
40
45
50
55
60
65
70
75
80
Age (years)
Males
Female

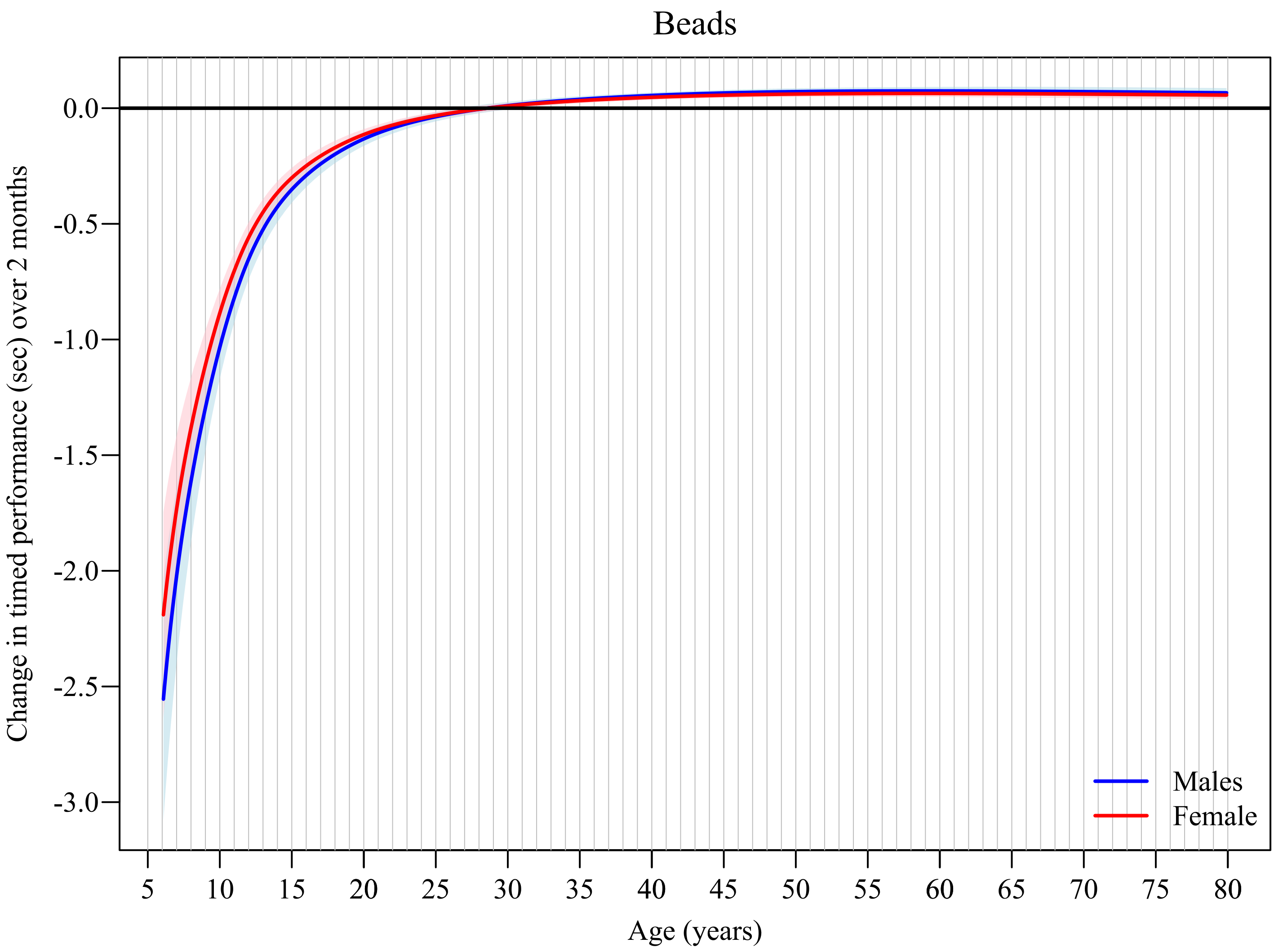
Beads
Change in timed performance (sec) over 2 months
0.0
-0.5
-1.0
-1.5
-2.0
-2.5
-3.0
5
10
15
20
25
30
35
40
45
50
55
60
65
70
75
80
Age (years)
Males
Female

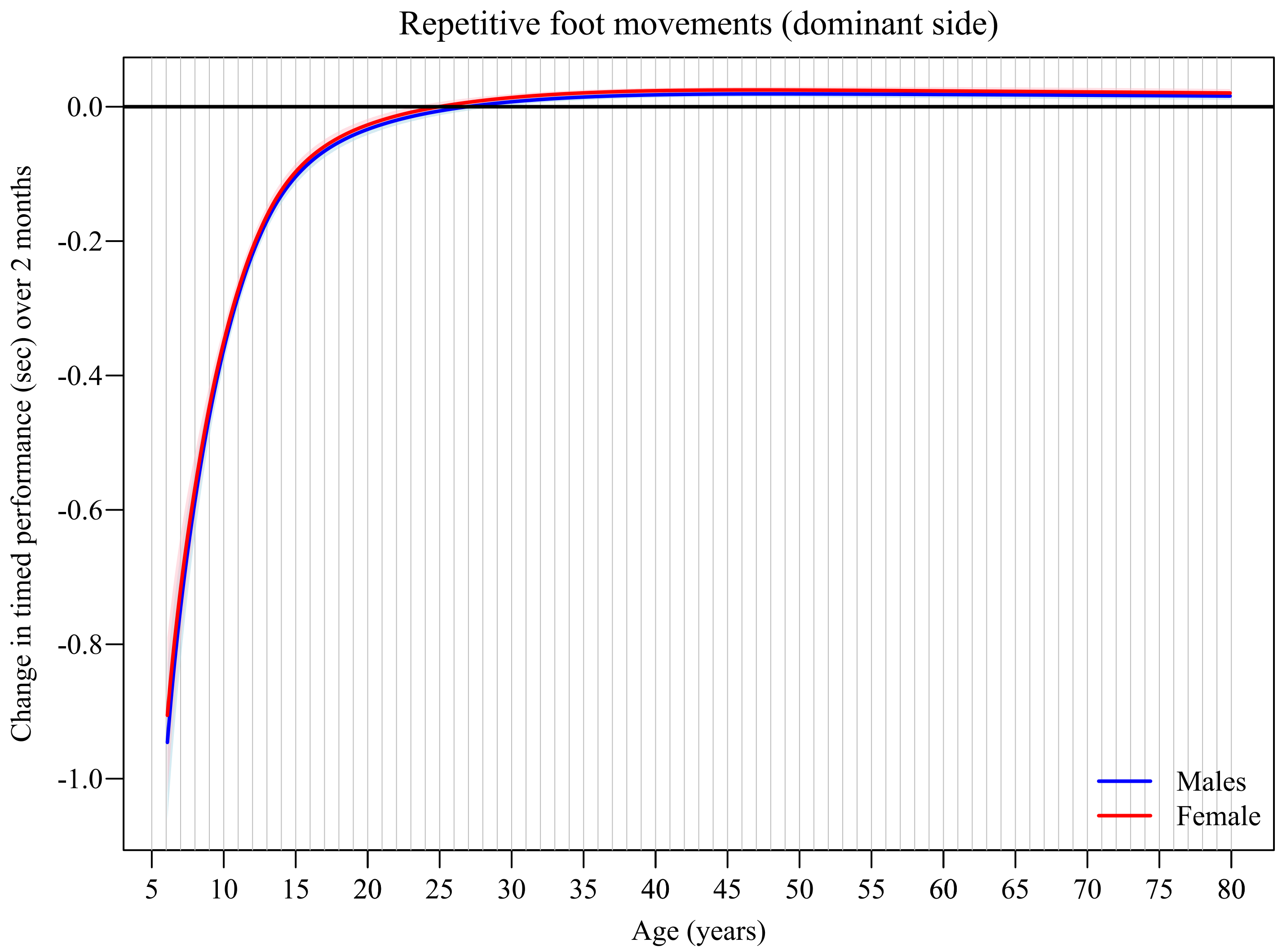
Repetitive foot movements (dominant side)
Change in timed performance (sec) over 2 months
0.0
-0.2
-0.4
-0.6
-0.8
-1.0
5
10
15
20
25
30
35
40
45
50
55
60
65
70
75
80
Age (years)
Males
Female

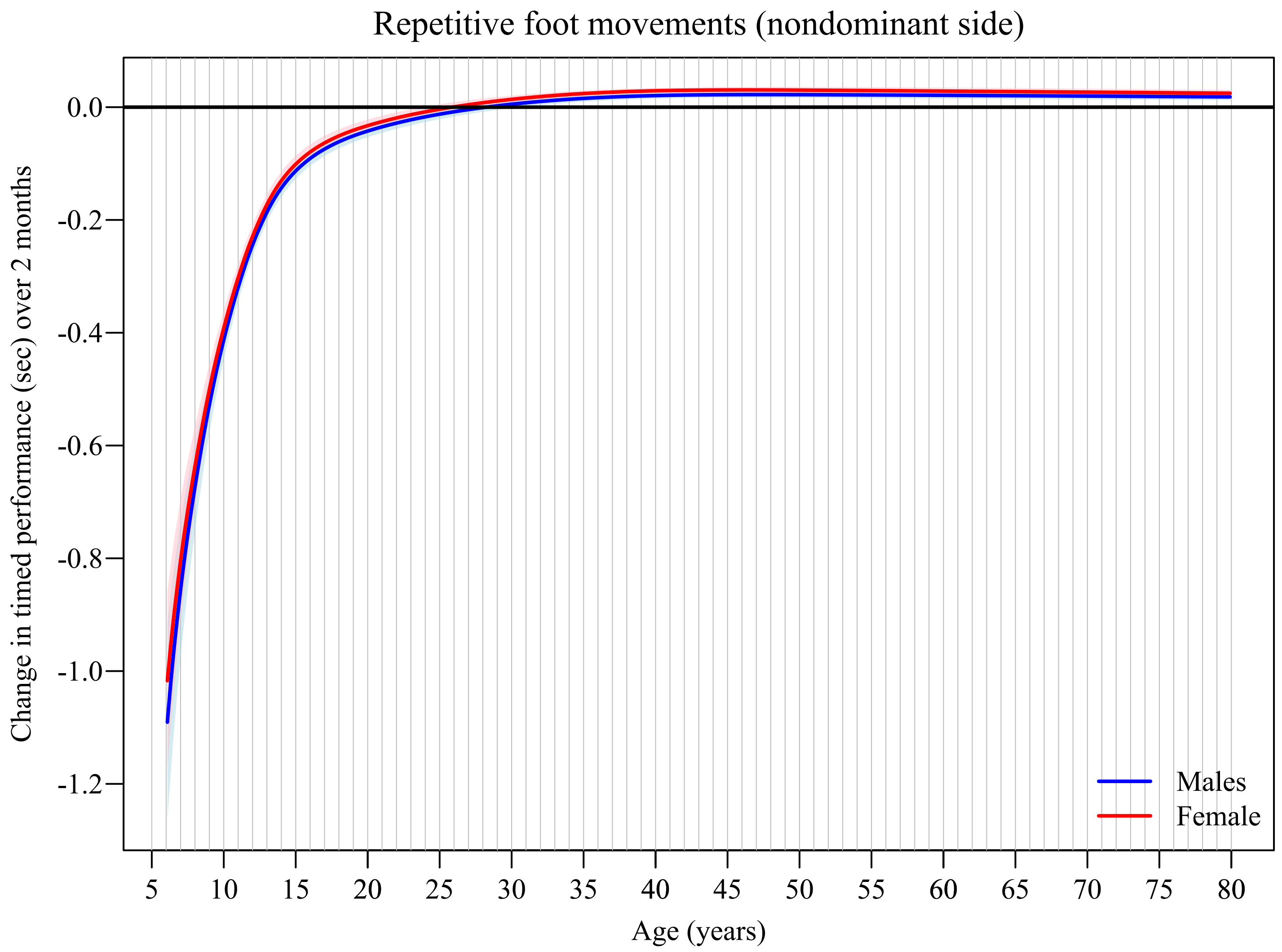
Repetitive foot movements (nondominant side)
Change in timed performance (sec) over 2 months
0.0
-0.2
-0.4
-0.6
-0.8
-1.0
-1.2
5
10
15
20
25
30
35
40
45
50
55
60
65
70
75
80
Age (years)
Males
Female

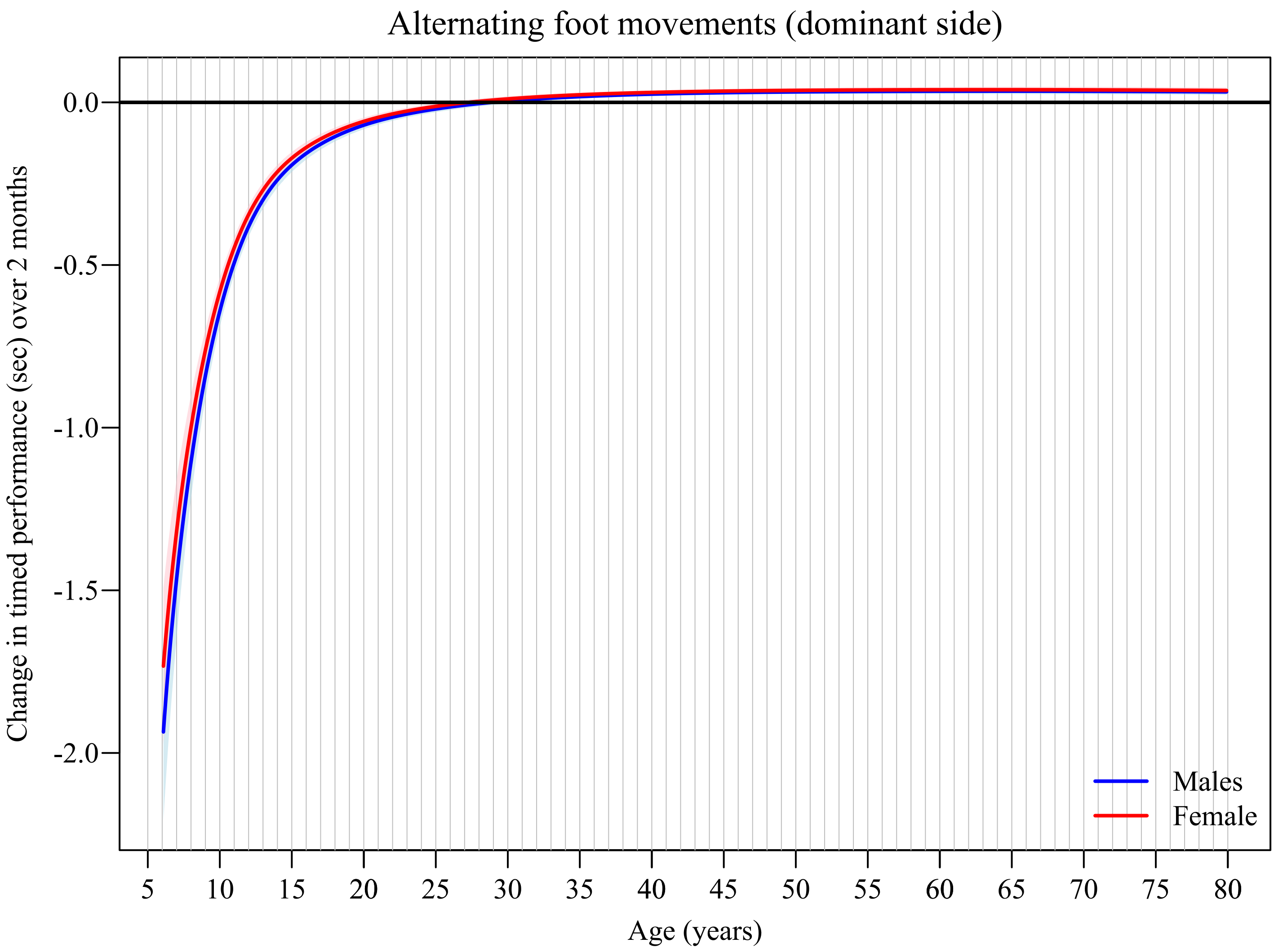
Alternating foot movements (dominant side)
Change in timed performance (sec) over 2 months
0.0
-0.5
-1.0
-1.5
-2.0
5
10
15
20
25
30
35
40
45
50
55
60
65
70
75
80
Age (years)
Males
Female

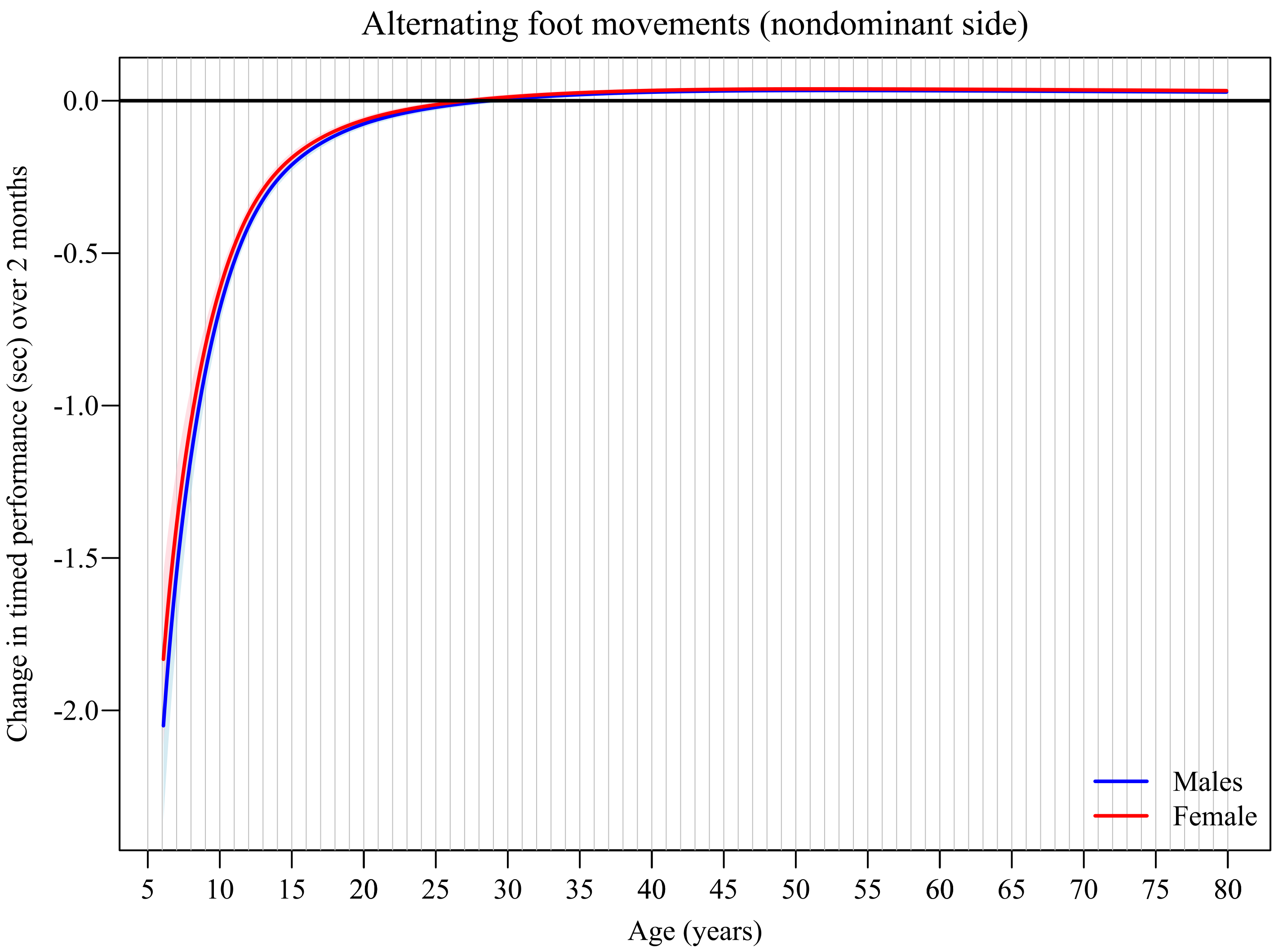
Alternating foot movements (nondominant side)
Change in timed performance (sec) over 2 months
0.0
-0.5
-1.0
-1.5
-2.0
5
10
15
20
25
30
35
40
45
50
55
60
65
70
75
80
Age (years)
Males
Female

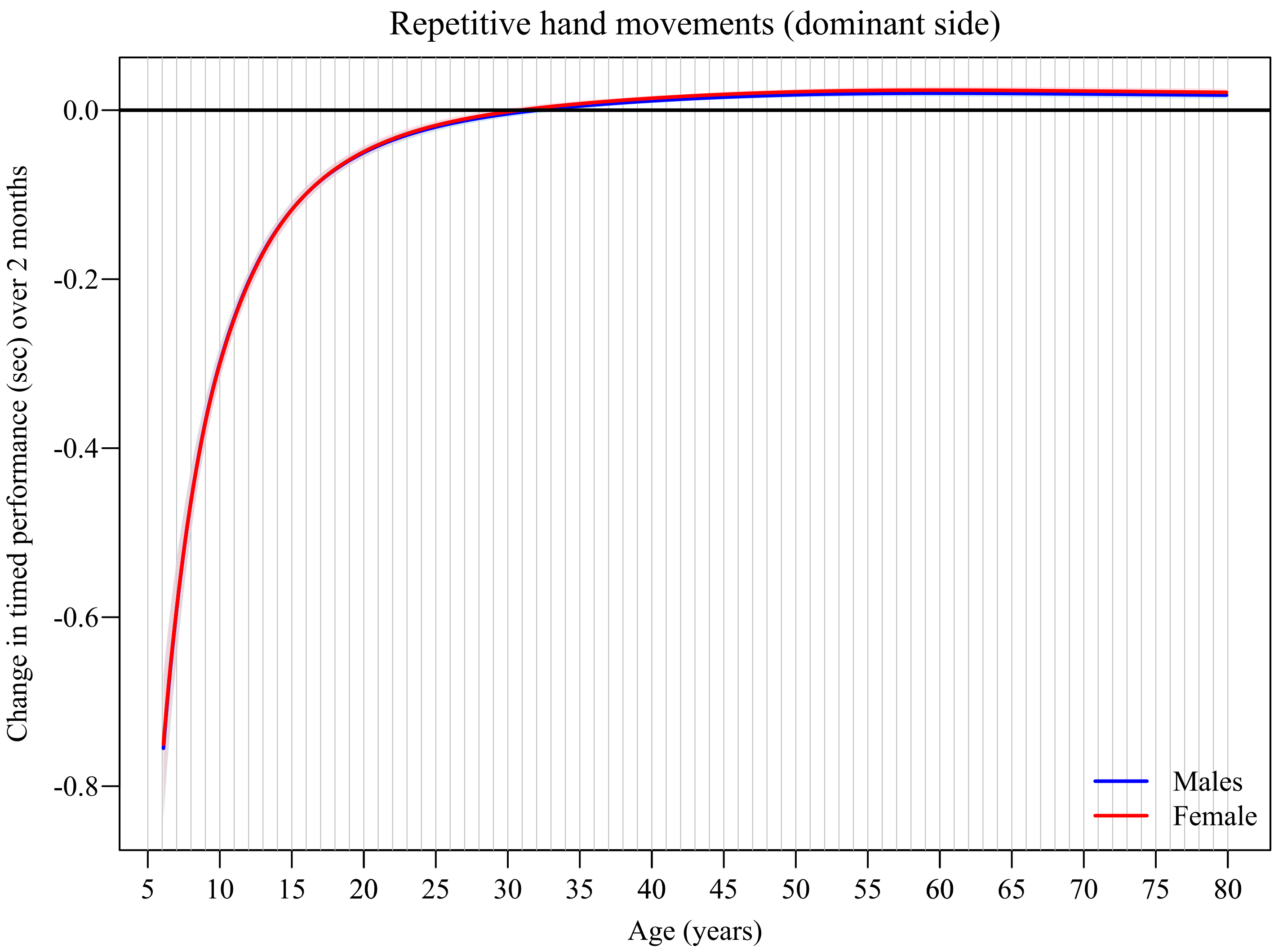
Repetitive hand movements (dominant side)
Change in timed performance (sec) over 2 months
0.0
-0.2
-0.4
-0.6
-0.8
5
10
15
20
25
30
35
40
45
50
55
60
65
70
75
80
Age (years)
Males
Female

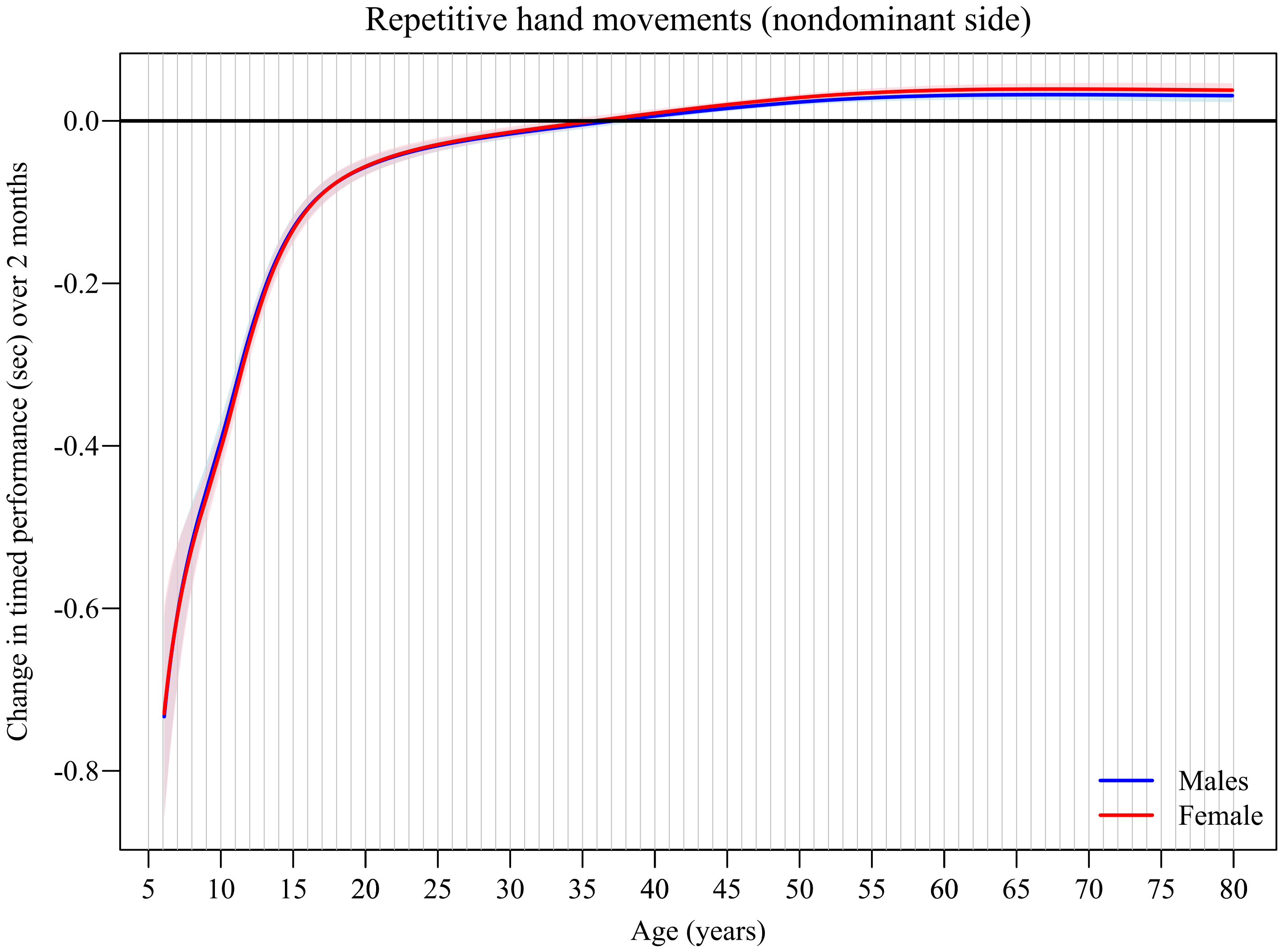
Repetitive hand movements (nondominant side)
Change in timed performance (sec) over 2 months
0.0
-0.2
-0.4
-0.6
-0.8
5
10
15
20
25
30
35
40
45
50
55
60
65
70
75
80
Age (years)
Males
Female

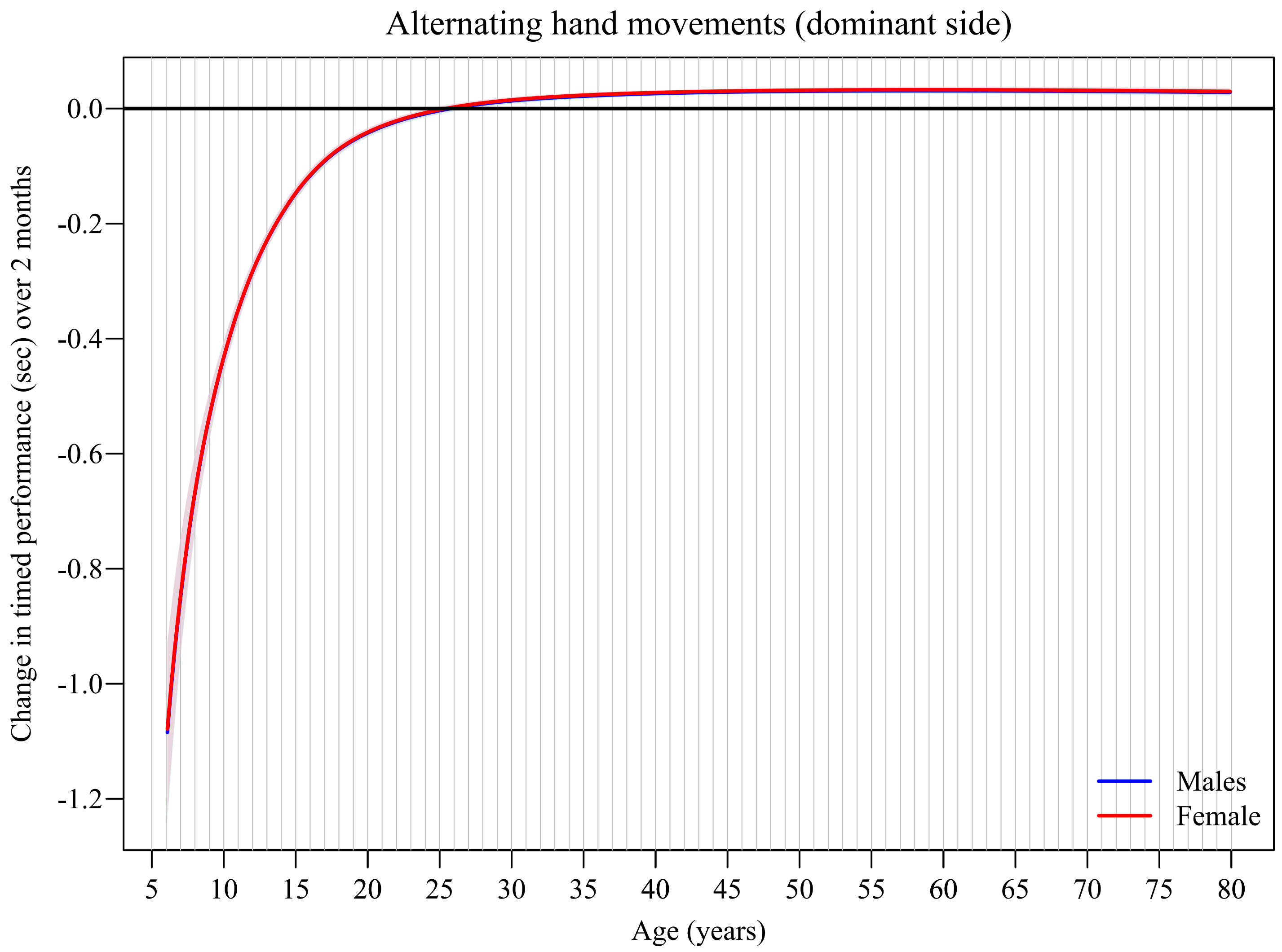
Alternating hand movements (dominant side)
Change in timed performance (sec) over 2 months
0.0
-0.2
-0.4
-0.6
-0.8
-1.0
-1.2
5
10
15
20
25
30
35
40
45
50
55
60
65
70
75
80
Age (years)
Males
Female

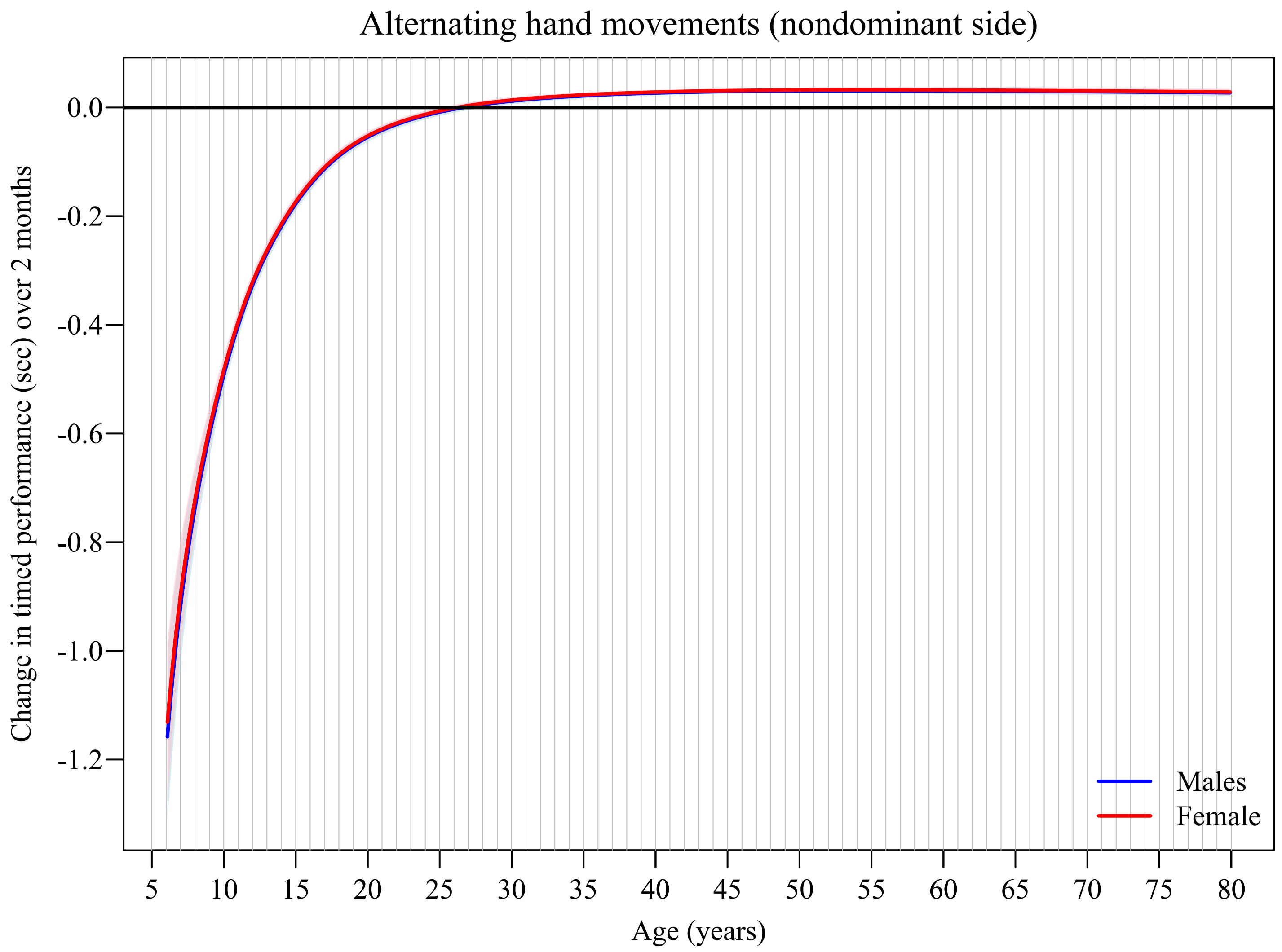
Alternating hand movements (nondominant side)
Change in timed performance (sec) over 2 months
0.0
-0.2
-0.4
-0.6
-0.8
-1.0
-1.2
5
10
15
20
25
30
35
40
45
50
55
60
65
70
75
80
Age (years)
Males
Female

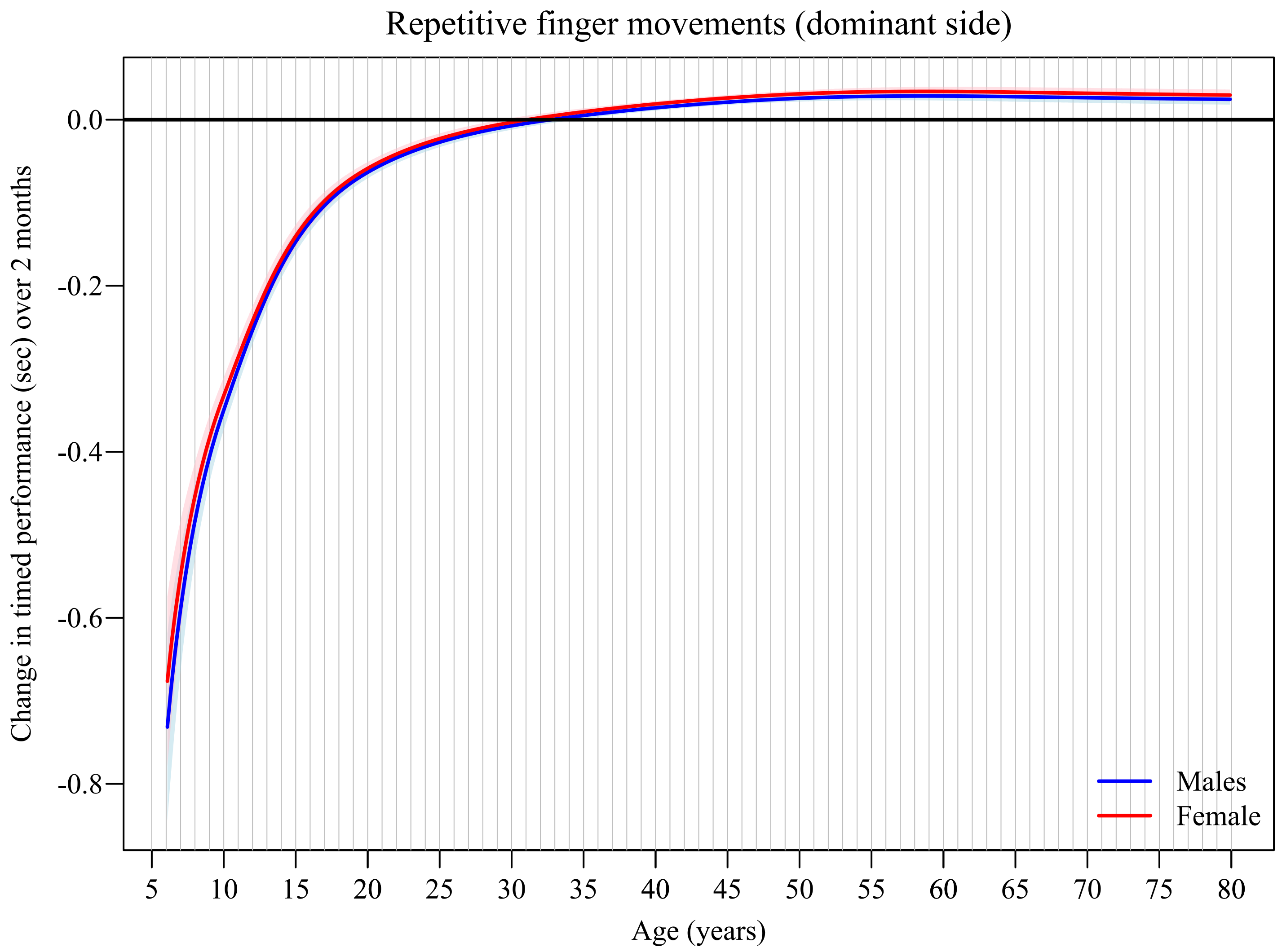
Repetitive finger movements (dominant side)
Change in timed performance (sec) over 2 months
0.0
-0.2
-0.4
-0.6
-0.8
Males
Female
5
10
15
20
25
30
35
40
45
50
55
60
65
70
75
80
Age (years)

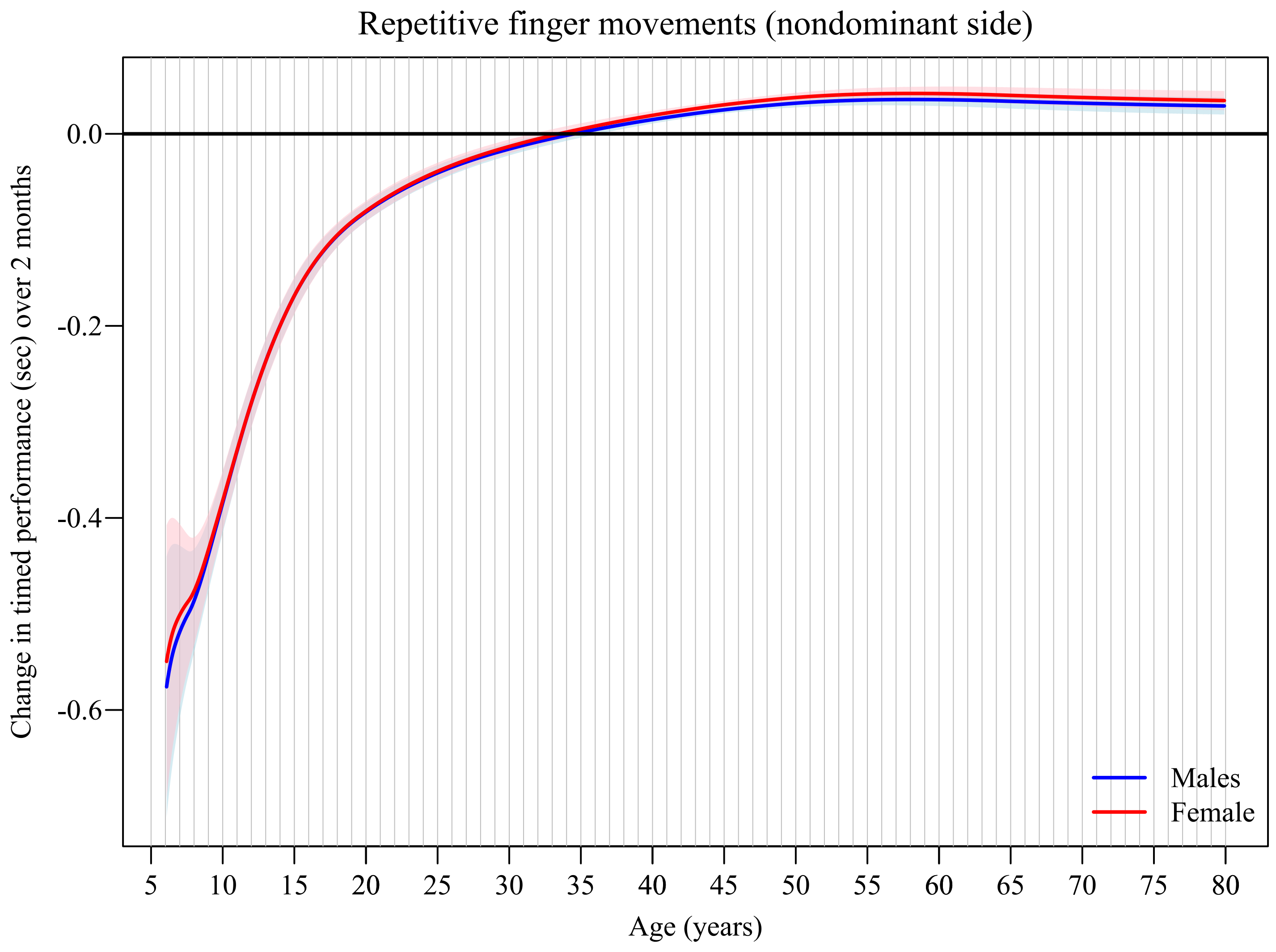
Repetitive finger movements (nondominant side)
Change in timed performance (sec) over 2 months
0.0
-0.2
-0.4
-0.6
Males
Female
5
10
15
20
25
30
35
40
45
50
55
60
65
70
75
80
Age (years)

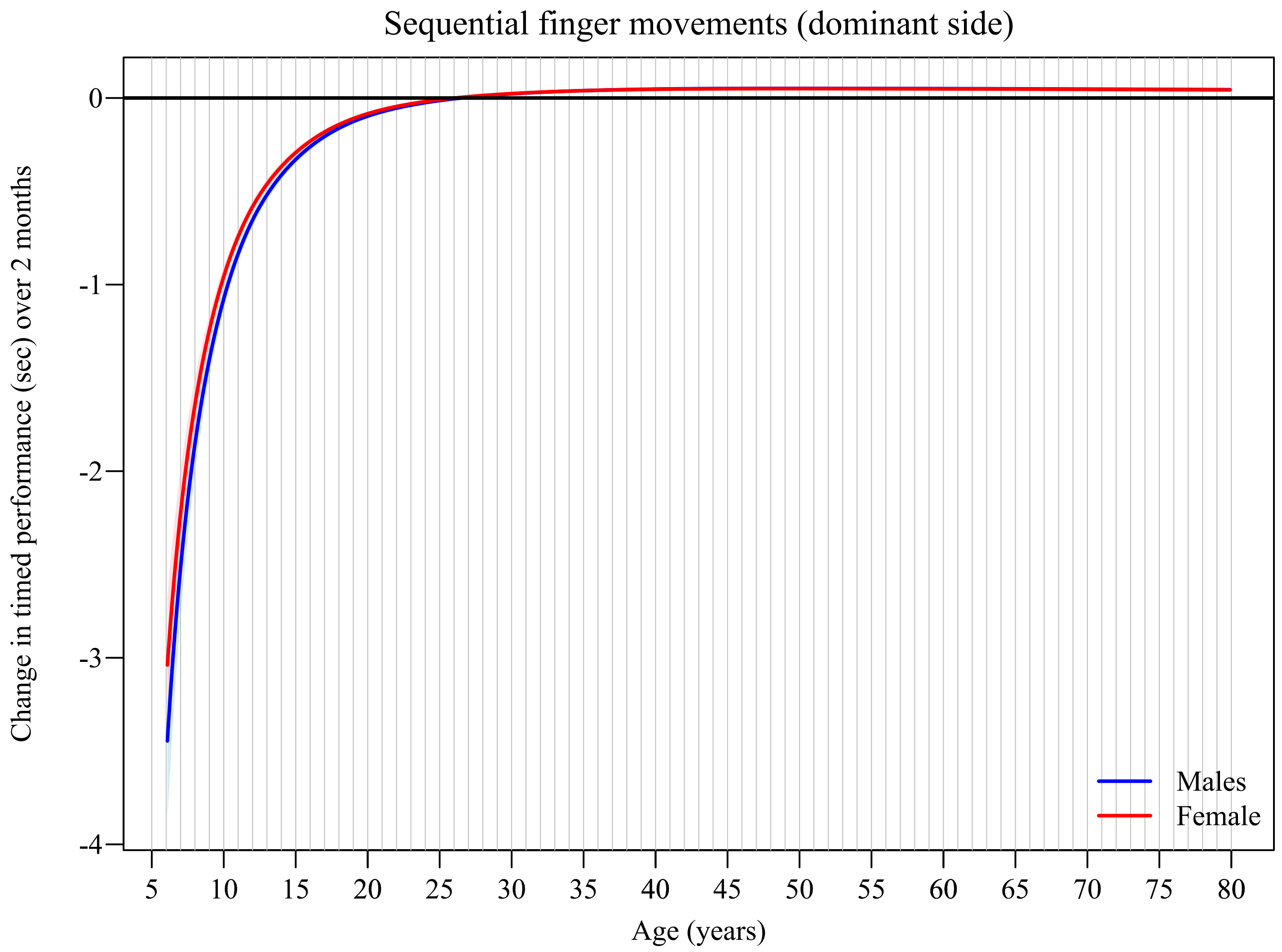

Sequential finger movements (dominant side)
Change in timed performance (sec) over 2 months
0
-1
-2
-3
-4
5
10
15
20
25
30
35
40
45
50
55
60
65
70
75
80
Age (years)
Males
Female

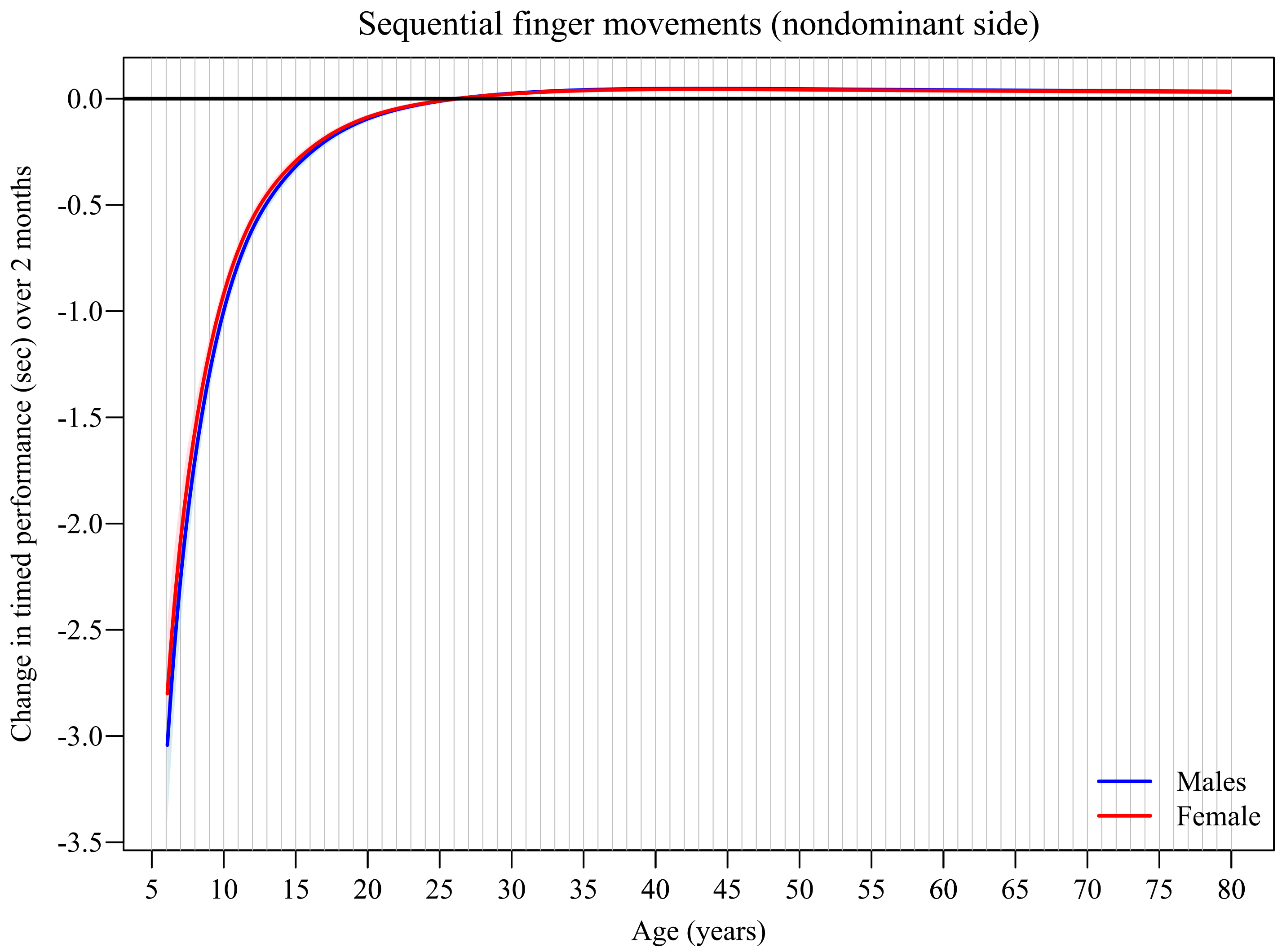

Sequential finger movements (nondominant side)
Change in timed performance (sec) over 2 months
0.0
-0.5
-1.0
-1.5
-2.0
-2.5
-3.0
-3.5
5
10
15
20
25
30
35
40
45
50
55
60
65
70
75
80
Age (years)
Males
Female

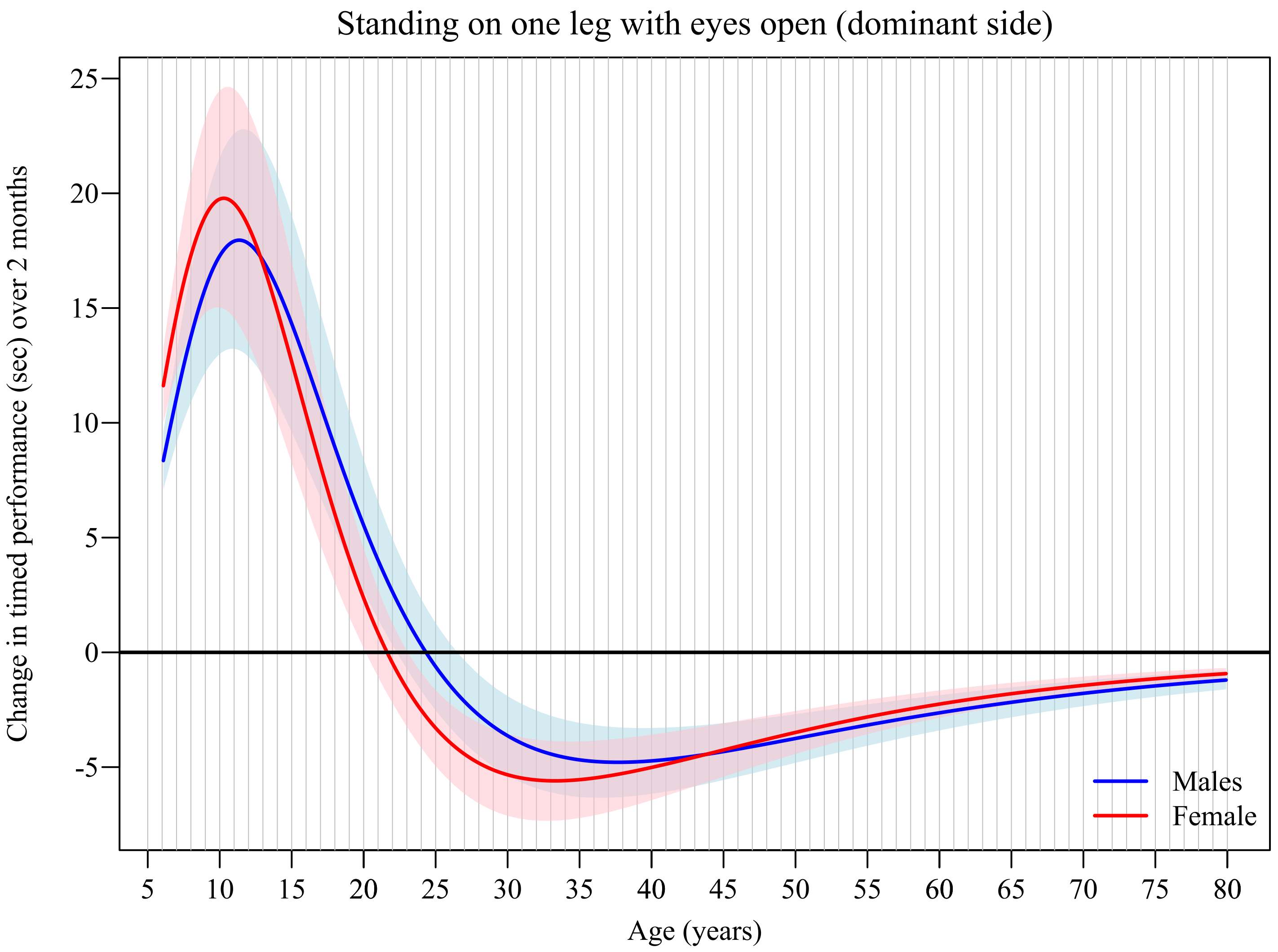

Standing on one leg with eyes open (dominant side)
Change in timed performance (sec) over 2 months
25
20
15
10
5
0
-5
5
10
15
20
25
30
35
40
45
50
55
60
65
70
75
80
Age (years)
Males
Female

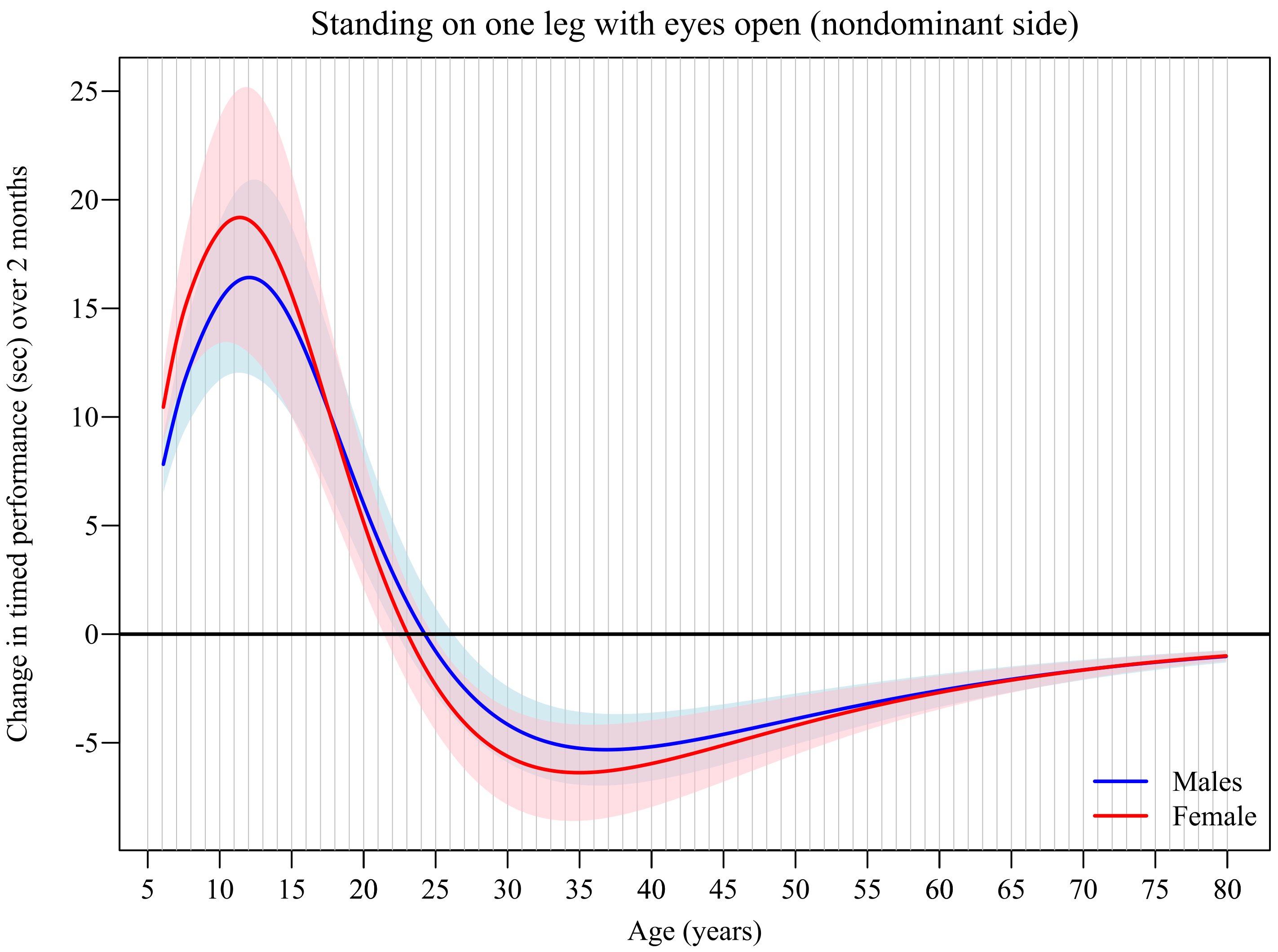

Standing on one leg with eyes open (nondominant side)
Change in timed performance (sec) over 2 months
25
20
15
10
5
0
-5
5
10
15
20
25
30
35
40
45
50
55
60
65
70
75
80
Age (years)
Males
Female

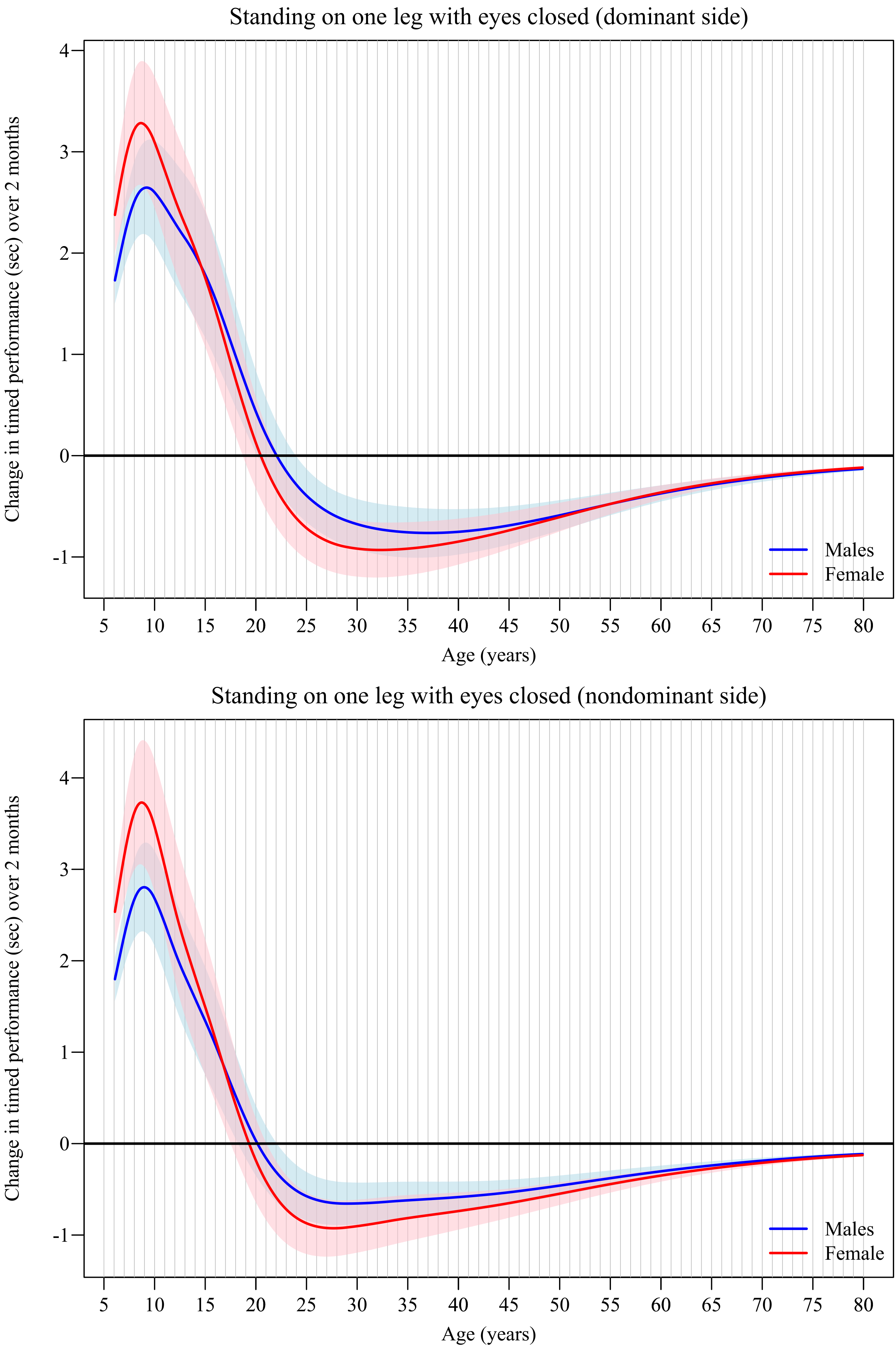
Standing on one leg with eyes closed (dominant side)
Change in timed performance (sec) over 2 months
Age (years)
Males
Female
4
3
2
1
0
-1
5
10
15
20
25
30
35
40
45
50
55
60
65
70
75
80
Standing on one leg with eyes closed (nondominant side)
Change in timed performance (sec) over 2 months
Age (years)
Males
Female

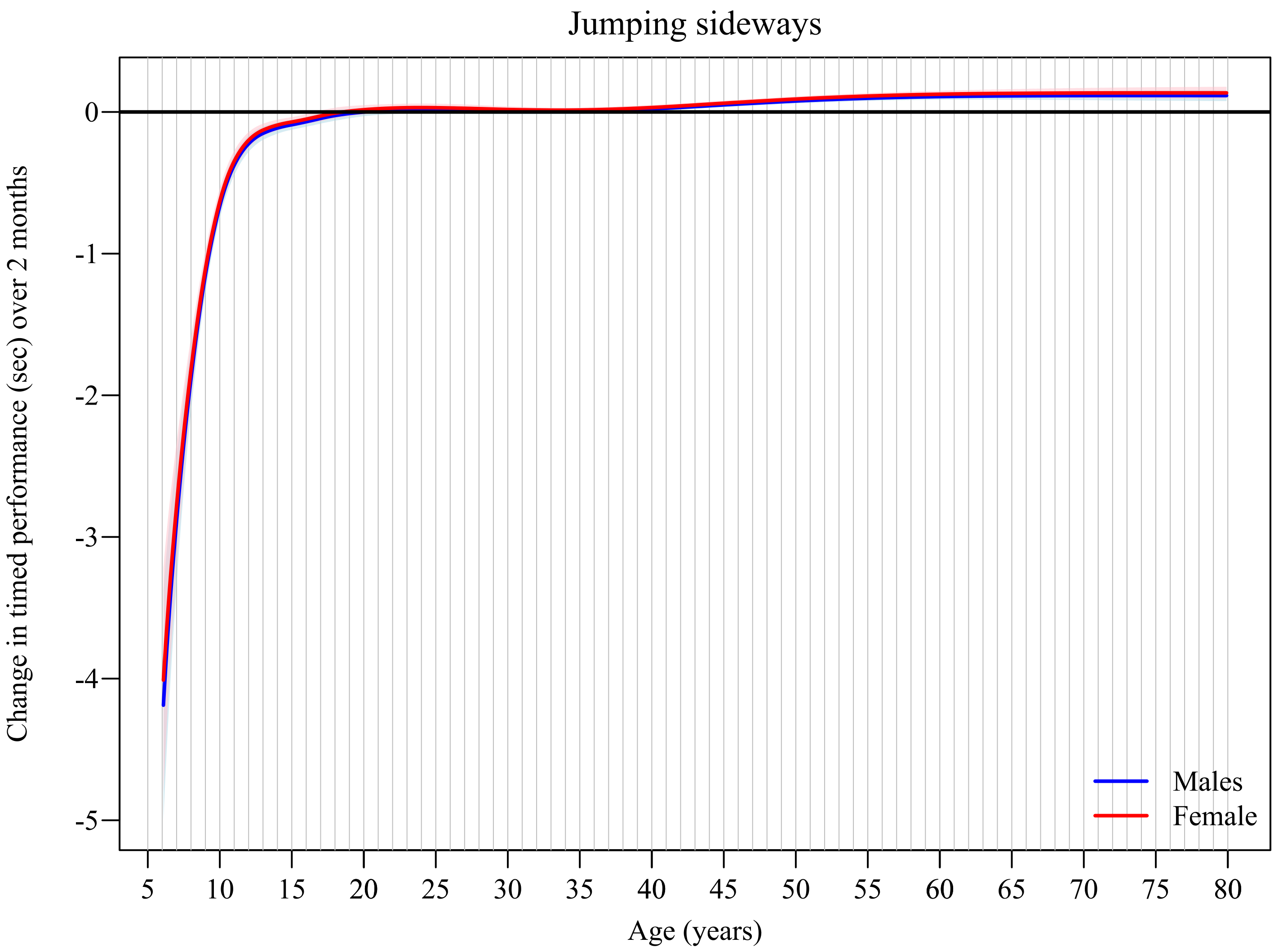

Jumping sideways
Change in timed performance (sec) over 2 months
0
-1
-2
-3
-4
-5
5 10 15 20 25 30 35 40 45 50 55 60 65 70 75 80
Age (years)
Males
Female

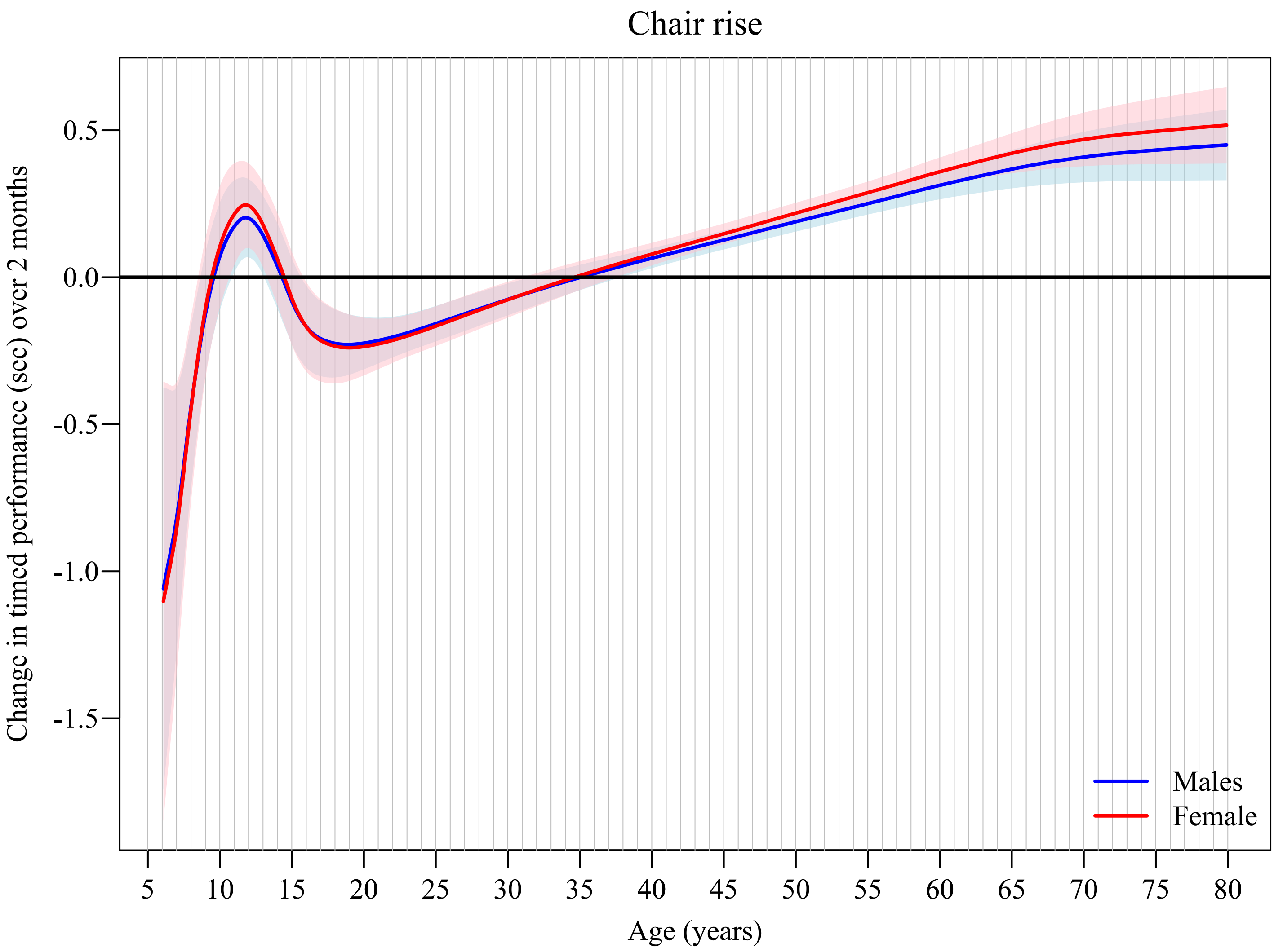

Chair rise
Change in timed performance (sec) over 2 months
0.5
0.0
-0.5
-1.0
-1.5
5 10 15 20 25 30 35 40 45 50 55 60 65 70 75 80
Age (years)
Males
Female

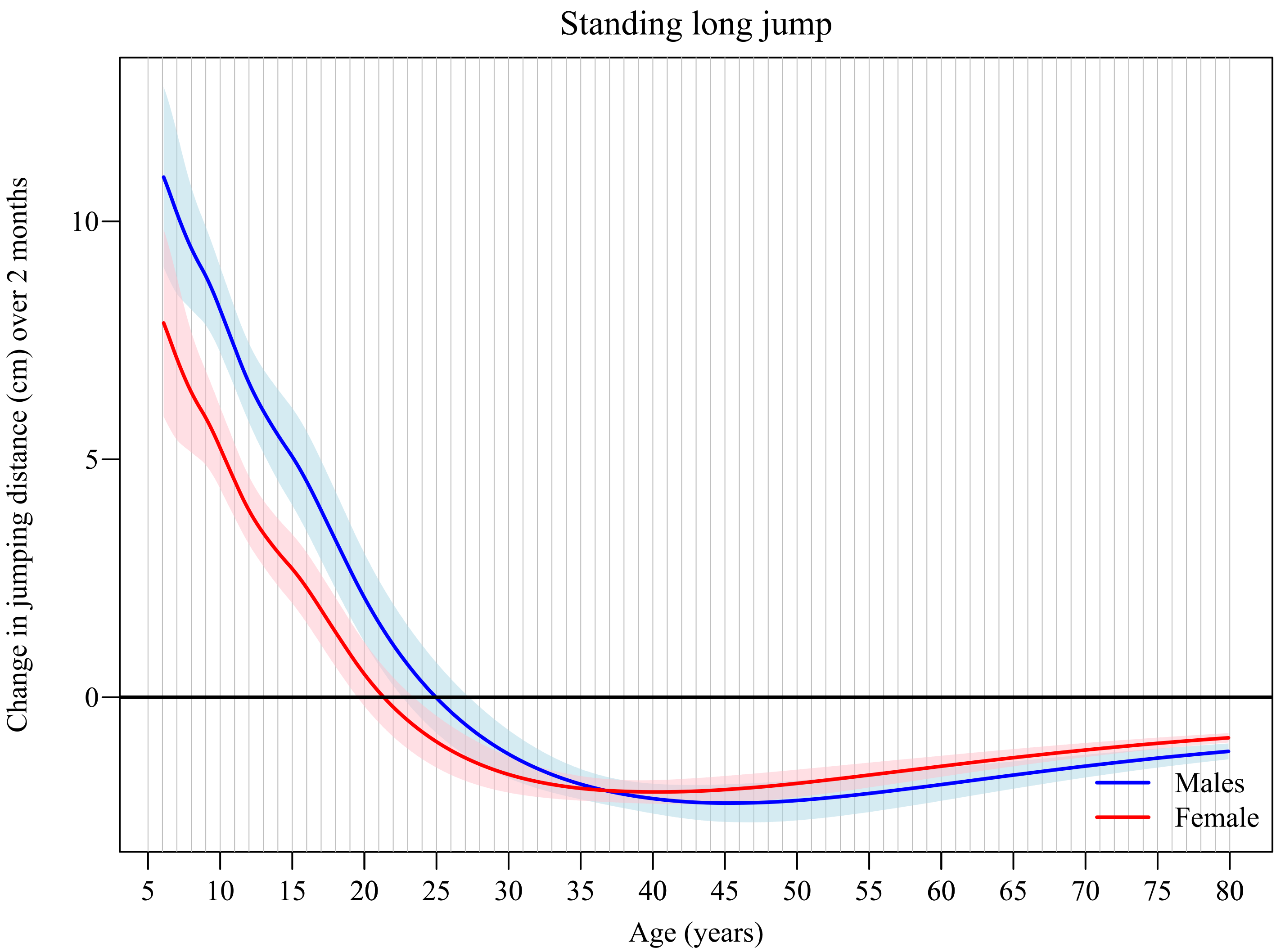
Standing long jump
Change in jumping distance (cm) over 2 months
10
5
0
5
10
15
20
25
30
35
40
45
50
55
60
65
70
75
80
Age (years)
Males
Female
